# Safety and Immunogenicity Study of Multiclade HIV-1 Adenoviral Vector Vaccine Alone or as Boost following a Multiclade HIV-1 DNA Vaccine in Africa

**DOI:** 10.1371/journal.pone.0012873

**Published:** 2010-09-21

**Authors:** Walter Jaoko, Etienne Karita, Kayitesi Kayitenkore, Gloria Omosa-Manyonyi, Susan Allen, Soe Than, Elizabeth M. Adams, Barney S. Graham, Richard A. Koup, Robert T. Bailer, Carol Smith, Len Dally, Bashir Farah, Omu Anzala, Claude M. Muvunyi, Jean Bizimana, Tony Tarragona-Fiol, Philip J. Bergin, Peter Hayes, Martin Ho, Kelley Loughran, Wendy Komaroff, Gwynneth Stevens, Helen Thomson, Mark J. Boaz, Josephine H. Cox, Claudia Schmidt, Jill Gilmour, Gary J. Nabel, Patricia Fast, Job Bwayo

**Affiliations:** 1 Kenya AIDS Vaccine Initiative (KAVI), Nairobi, Kenya; 2 Projet San Francisco (PSF), Rwanda-Zambia HIV Research Project, Kigali, Rwanda; 3 Rollins School of Public Health, Emory University, Atlanta, Georgia, United States of America; 4 International AIDS Vaccine Initiative (IAVI), New York, New York, United States of America; 5 Vaccine Clinical Research Branch (VCRB), Vaccine Research Program (VRP)/Division of AIDS (DAIDS)/National Institute of Allergy and Infectious Diseases (NIAID)/National Institutes of Health (NIH), Bethesda, Maryland, United States of America; 6 Vaccine Research Center (VRC)/NIAID/NIH, Bethesda, Maryland, United States of America; 7 The EMMES Corporation, Rockville, Maryland, United States of America; 8 IAVI Human Immunology Laboratory, Imperial College, London, United Kingdom; Queensland Institute of Medical Research, Australia

## Abstract

**Background:**

We conducted a double-blind, randomized, placebo-controlled Phase I study of a recombinant replication-defective adenovirus type 5 (rAd5) vector expressing HIV-1 Gag and Pol from subtype B and Env from subtypes A, B and C, given alone or as boost following a DNA plasmid vaccine expressing the same HIV-1 proteins plus Nef, in 114 healthy HIV-uninfected African adults.

**Methodology/Principal Findings:**

Volunteers were randomized to 4 groups receiving the rAd5 vaccine intramuscularly at dosage levels of 1×10^10^ or 1×10^11^ particle units (PU) either alone or as boost following 3 injections of the DNA vaccine given at 4 mg/dose intramuscularly by needle-free injection using Biojector® 2000. Safety and immunogenicity were evaluated for 12 months. Both vaccines were well-tolerated. Overall, 62% and 86% of vaccine recipients in the rAd5 alone and DNA prime - rAd5 boost groups, respectively, responded to the HIV-1 proteins by an interferon-gamma (IFN-γ) ELISPOT. The frequency of immune responses was independent of rAd5 dosage levels. The highest frequency of responses after rAd5 alone was detected at 6 weeks; after DNA prime - rAd5 boost, at 6 months (end of study). At baseline, neutralizing antibodies against Ad5 were present in 81% of volunteers; the distribution was similar across the 4 groups. Pre-existing immunity to Ad5 did not appear to have a significant impact on reactogenicity or immune response rates to HIV antigens by IFN-γ ELISPOT. Binding antibodies against Env were detected in up to 100% recipients of DNA prime - rAd5 boost. One volunteer acquired HIV infection after the study ended, two years after receipt of rAd5 alone.

**Conclusions/Significance:**

The HIV-1 rAd5 vaccine, either alone or as a boost following HIV-1 DNA vaccine, was well-tolerated and immunogenic in African adults. DNA priming increased the frequency and magnitude of cellular and humoral immune responses, but there was no effect of rAd5 dosage on immunogenicity endpoints.

**Trial Registration:**

ClinicalTrials.gov NCT00124007

## Introduction

In a Phase IIb/III community-based clinical trial in Thailand, prevention from HIV infection was demonstrated for the first time with a combination of ALVAC-HIV (canarypox vectored HIV vaccine) and AIDSVAX B/E (recombinant protein-based HIV vaccine) [1]. Vaccine efficacy was modest and there was no effect on viral load. Thus, the development of a safe and more efficacious preventive HIV vaccine remains a high public health priority.

A recombinant multiclade adenovirus type 5 (rAd5) vector-based vaccine expressing HIV-1 subtype B Gag and Pol and subtypes A, B and C Env (VRC HIV-1 rAd5), and a recombinant DNA vaccine encoding the same proteins plus subtype B Nef (VRC HIV-1 DNA), developed by the Vaccine Research Center (VRC) at the National Institute of Allergy and Infectious Diseases (NIAID) of the National Institutes of Health (NIH), have been evaluated previously either alone or in a DNA prime - rAd5 boost combination in healthy, HIV-uninfected volunteers; both vaccines were well-tolerated and immunogenic [2], [3], [4], [5], [6], [7]. To further develop these vaccines, three clinical studies were conducted simultaneously: i) the V001 study - presented here - sponsored by the International AIDS Vaccine Initiative (IAVI) in collaboration with the Division of AIDS (DAIDS)/NIAID/NIH, conducted in Kenya and Rwanda, ii) the HIV Vaccine Trials Network (HVTN) 204 study sponsored by DAIDS, NIAID, conducted in the US, Latin America, the Caribbean and South Africa and iii) the US Military HIV Research Program (USMHRP) sponsored RV172 study supported by DAIDS, NIAID, conducted in Kenya, Uganda and Tanzania [8]. The three trials were randomized, double-blind, placebo-controlled and tested the same vaccines in HIV-seronegative volunteers. RV172 enrolled 324 volunteers and used the same vaccination schedules as V001. HVTN 204 enrolled 480 volunteers and evaluated the DNA prime - rAd5 boost schedule only. The objectives of the studies were to evaluate the safety and immunogenicity of the two vaccines in preparation for future Phase IIb and efficacy trials. After publication of the STEP study results [9], [10] in November 2007, plans for larger trials using rAd5-vectored vaccines were suspended temporarily. The two vaccines are currently being tested in a Phase II safety and effectiveness trial in the US in HIV-uninfected, circumcised men who have sex with men and who are negative for neutralizing antibodies against Ad5 at baseline. (HVTN 505; http://clinicaltrials.gov/ct2/show/NCT00865566).

### Participants

Healthy HIV-uninfected adults aged 18–50 years were recruited at the Kenya AIDS Vaccine Initiative (KAVI), Nairobi, Kenya and at Project San Francisco (PSF), Kigali, Rwanda. Volunteers reported no increased risk for HIV (i.e., unprotected vaginal or anal sex with known HIV infected person; sex in exchange for money or drugs; a sexually transmitted infection within 6 months before vaccination). They were willing to undergo HIV testing and receive results. They agreed to use effective contraceptive methods for the duration of their participation if sexually active and not to become pregnant. Baseline serum neutralizing antibodies against Ad5 were measured, but not used as an eligibility criterion. All participants provided written informed consent.

### Ethics Statement

This study was a double-blind, randomized, placebo-controlled Phase I clinical trial (**Table 1**), approved by the appropriate local and international Independent Ethics Committee (IEC) or Institutional Review Board (IRB), and scientific and regulatory authorities as follows: in Kenya [Kenyatta National Hospital Ethics and Research Committee (KNHERC), the National Council of Science and Technology (NCST) and the Institutional Biosafety Committee (IBC)]; in Rwanda [The Rwanda National Ethics Committee, The Ministry of Health and The Institutional Biosafety Committee]; and in the USA (NIAID IRB and the Emory University IRB). The study was conducted according to International Conference on Harmonisation - Good Clinical Practice (ICH-GCP) and Good Clinical Laboratory Practice (GCLP) [11].

**Table 1:** Number of Volunteers Enrolled, Vaccination Schedule, Sample Collection and Assessment Time Points.

Group (N/n)^a^	Vaccines/Dosage Group^b^	W0	W2	W4	W6	W8	W10	W12	W24	W28	W30	W36	W48
**A** (13/5)	rAd5 10^10^	Sc,d,e ↓	S^e^	Sc,d,e	S^d^							S^d^	Sc,d.e
**B** (13/4)	rAd5 10^11^	Sc,d,e ↓	S^e^	Sc,d,e	S^d^							S^e^	Sc,d,e
**C** (29/11)	3DNA + rAd5 10^10^	Sc,d,e ↓		↓		↓	S d,e	S^d^	Sc,d,e ↓	Sc,d,e	Sd,e	S^d^	Sc,d,e
**D** (29/10)	3DNA + rAd5 10^11^	Sc,d,e ↓		↓		↓	S d,e	S^d^	Sc,d,e ↓	Sc,d,e	Sd,e	S^d^	Sc,d,e

a (N/n) Number of vaccine recipients/number of placebo recipients per group.

b Vaccines/Dosage Groups: - rAd5: VRC HIV-1 rAd5 at 1×10^10^ or 1×10^11^ PU - 3DNA: 3 doses of VRC HIV-1 DNA at 4 mg/dose given intramuscularly by needle-free injection using Biojector ® 2000.

↓indicates vaccination visit.

S: Sample collection and assessment time points (S):

c Serum neutralizing antibodies against Ad5.

d Vaccine-induced HIV-1 specific cellular immune responses.

e Vaccine-induced HIV-1 specific humoral immune responses.

### Interventions

The study vaccines were designed and constructed at the Vaccine Research Center (VRC), Bethesda, MD, USA and were described previously [3], [5].The recombinant, multiclade HIV-1 DNA vaccine (VRC-HIVDNA016-00-VP) is composed of six individual, closed, circular plasmids expressing clade B HIV-1 gag, pol, nef and clades A, B and C env. 50% of the mass is represented by gag, pol, nef and the other 50% by EnvA, Env B and EnvC (16.67% each). The recombinant, multiclade HIV-1 adenoviral vector (VRC-HIVADV014-00-VP) is composed of four replication-defective, serotype 5 adenoviral vectors in a 3∶1∶1∶1 ratio encoding HIV-1 Clade B Gag-Pol polyprotein and HIV-1 Env glycoproteins from clades A, B, and C. In the rAd5 vector, the Gag-Pol genes are present as fusion protein.

The rAd5 vaccine was given intramuscularly by needle injection at dosage levels of 1×10^10^ particle units (PU; low dosage, LD) or 1×10^11^ PU (high dosage, HD). The DNA vaccine was given at 4 mg/dose intramuscularly by needle-free injection (Biojector® 2000) (**Table 1**). Phosphate buffered saline was used as placebo for the DNA vaccine; final formulation buffer (VRC–DILUENT013-DIL-VP) was used as placebo for the rAd5 vaccine. Neither of the study vaccines can cause HIV infection.

### Objectives

The primary objective of the V001 study was to evaluate the safety and tolerability of a rAd5 vector expressing multiple HIV-1 proteins at two different dosage levels, given alone or as a boost following DNA plasmid vaccine in healthy HIV-uninfected adults in East Africa. The secondary objective was to evaluate the humoral and cellular immunogenicity of the vaccine at each dosage level.

## Materials and Methods

The protocol for this trial and supporting CONSORT checklist are available as supporting information; see Checklist S1 and Protocol S1.

### Safety monitoring

Study participants were monitored by interim medical history, and by physical and laboratory assessments. Local (pain, tenderness, erythema, induration, edema, papule, blister/vesicle, scab) and systemic signs and symptoms (headache, fever, chills, malaise and/or fatigue, myalgia, arthralgia, nausea, vomiting) were solicited for 3 days. Unsolicited adverse events (AEs) were recorded throughout the study, graded for severity (Grade 1 = mild, Grade 2 = moderate, Grade 3 = severe, Grade 4 = potentially life threatening) and classified by MedDRA (Medical Dictionary for Regulatory Activities). The AEs were assessed for relationship to study vaccines. There were 5 categories of relatedness: definitely, probably, possibly, probably not and not related. All safety data were reviewed quarterly by an independent Data and Safety Monitoring Board (DSMB). Protocol deviations were monitored throughout the trial.

### HIV testing

At screening, HIV testing was performed according to the respective national algorithms. Only HIV-seronegative individuals were enrolled. During the study, a protocol-specific algorithm was followed, using two different HIV-1 ELISA tests selected from Abbott Murex HIV Ag/Ab Combination Assay (Manufactured by Murex Biotech Limited, United Kingdom), Vironostika Uni-Form II Plus O (Manufactured by BioMerieux bv, Boxtel, The Netherlands) or Adaltis Detect HIV v2 (ADALTIS Inc. Montreal, Quebec Canada). If an ELISA test result was positive, a nucleic acid test (HIV RNA PCR, Roche Amplicor Standard Assay V1.5 manufactured by Roche Diagnostics GmbH, Mannheim, Germany) was performed to confirm or refute incident HIV infection in the presence of vaccine-induced antibodies. During the study, clinic staff was blinded to HIV test results. Volunteers who had a positive HIV ELISA test results due to vaccine-induced antibodies at the final study visit are being followed in a long-term follow up study until serological test results return to negative.

### Ad5 neutralizing antibody assay

Serum anti-Ad5 neutralizing titers were measured at baseline and designated time points throughout the study (**Table 1**) using previously described qualified assays [12], [13]. Testing of baseline sera for assessment of the impact of pre-existing Ad5 neutralizing titers was done at Crucell Holland BV (Netherlands), and pre- and post-vaccination Ad5 neutralizing titers were done at the NVITAL Laboratory (Gaithersburg, MD). Briefly, heat inactivated, serially diluted sera, rAd5 luciferase and 1×10^4^ A549 cells (human lung carcinoma) were incubated in 96-well plates. The plates were incubated for 24 hours and luciferase activity was quantitated in lysed cell supernatants in a luminometer. The 90% inhibition serum titer was determined to be the serum dilution that can be interpolated to have 10% of the maximum luciferase activity, as determined by the assay run without a serum sample. The cut-off for a negative Ad5 titer was <19 and <12 for the Crucell and NVITAL assays respectively. Study groups were stratified by baseline Ad5 neutralizing titer, <19, 19–200 and >200 (measured by Crucell assay), which correspond approximately to the strata used in the STEP trial analysis [9], [10]. The proportion of volunteers with any IFN-γ ELISPOT responses after HIV-1 rAd5 alone was compared to HIV-1 DNA prime - rAd5 boost. For immunogenicity assessments across multiple time points, the data were stratified with a cut-off of <12 using the NVITAL assay.

### Humoral immunogenicity assays

#### Vaccine-induced HIV-1 specific humoral immune response

An ELISA assay was used to delineate the antibody response to each individual antigen encoded within the vaccine, as previously described [3]. Testing was performed at the NVITAL laboratory (Gaithersburg, MD). End-point titration of serum was performed in 96-well Immulon2 (Dynex Technologies) plates coated with preparations of purified recombinant proteins as described [3]. Titers were determined by sequential incubation of antigen with antisera, followed by biotin-labeled anti-human IgG, IgA, or IgM, and Streptavidin conjugated with horseradish peroxidase and TMB (3, 5′, 5,5′-tetra-methylbenzidine) substrate. Titer was calculated as the most dilute serum concentration that gave an optical density reading of >0.2 above background and reported as reciprocal dilution.

#### HIV-1 neutralization

Neutralization was examined in Groups A and B (rAd5 alone) at baseline, at 2 and 4 weeks post vaccination and at final study visit (12 months after injection), and in Groups C and D (DNA prime – rAd5 boost) at baseline, 2 weeks after the 3^rd^ injection of the DNA vaccine, at week 24 immediately prior to the rAd5 boost, 4 weeks and 6 weeks after the rAd5 boost, and at final study visit (6 months after the boost). Neutralizing activity was assessed against HIV isolates representing easy, moderate and hard to neutralize HIV isolates from around the world. The panel contained two primary isolates from each of the following clades: A, B, C, D, circulating recombinant form CRF01/AE and 2 lab adapted clade B isolates.

Neutralization was evaluated using a single-cycle recombinant pseudotype virus assay by Monogram Biosciences, Inc. (San Francisco, CA) as described previously [14]. Briefly, recombinant viruses pseudotyped with test virus envelope proteins were incubated for 1 h at 37°C with serial 4-fold dilutions of heat-inactivated patient plasma samples. U87 cells that express CD4 plus CCR5 and CXCR4 were inoculated with the virus-plasma dilutions and the virus infectivity was determined 3 days post inoculation by measuring luciferase expression in the infected cells. Neutralizing activity was reported as the percent inhibition of virus infection at 4 dilutions as compared with controls containing no test plasma. Virus pseudotyped with a murine leukemia virus (MLV) env was used as a control for non-specific effects in the assay. The cut-off for positivity was defined as greater than 1.7X the MLV control and over 50% inhibition.

### Cellular immunogenicity assays

#### IFN-γ ELISPOT assay

Vaccine-induced HIV-1 specific T cell responses were assessed at time points shown in **Table 1** using a validated IFN-γ ELISPOT assay described previously [15], [16], [17]. At KAVI, fresh peripheral blood mononuclear cells (PBMC) were analyzed, whereas the IAVI Human Immunology Laboratory analyzed previously frozen PBMC from KAVI and PSF. Viable PBMC counted by Vi-Cell XR counter (Beckman Coulter, UK) were plated at 2×10^5^ per well and stimulated in quadruplicate overnight with vaccine-matched 15-mer peptides overlapping by 11 aminoacids (aa), applied in pools of 100–200 peptides depending upon the protein, and at a final concentration of 1.5–2 µg/mL. Peptide pools matching the vaccine were supplied by the VRC; one pool each for EnvA, EnvB, Gag and Nef and 2 pools for Pol. Responses to the EnvC pool were not assessed due to high background responses observed in baseline and placebo samples. A peptide pool consisting of 32 Influenza (Flu), Epstein Barr Virus (EBV) and Cytomegalovirus (CMV) peptides and PHA were used as controls [18], [19]. Definition of a positive response: 1) Spot Forming Cells per million (SFC/10^6^) PBMC more than 4 times the mean background SFC count and greater than 38–68 SFC/10^6^ cells (depending upon the peptide pool) above the background. The cut-off was determined by the level of non-specific response observed from at least 180 samples from unvaccinated individuals. The cut-offs for EnvA, EnvB, Gag, Nef and Pol pools 1 and 2 were 40, 51, 54, 68, 51 and 38, respectively; 2) coefficient of variation across the replicate wells <70%; 3) background: <55 SFC/10^6^ PBMC. A vaccine recipient with a positive response to at least one pool is defined as a responder.

#### Intracellular cytokine staining (ICS)

ICS was measured in 7 volunteers each of Groups C and D, and in 6 placebo recipients at only three visits; baseline, 2 weeks post DNA and 4 weeks post rAd5. PBMC were shipped to NVITAL for testing. The method used to detect CD4 and CD8 T cell responsiveness by secretion of Interleukin-2 (IL-2) and/or IFN-γ to HIV peptides via ICS was based upon previously published methods [5]. Optimized concentrations of peptide pools (the same peptide pools used for the IFN-γ ELISPOT assay, aside from EnvC, which was used in the ICS but not ELISPOT assays) and antibodies to CD28 and CD49D were added to a final total volume of 200 µL/well with a total of 5.0×10^5^–1.0×10^6^ viable cells/well. The plates were incubated for 6 hours in the presence of Brefeldin A. Following cell permeabilization, the cells were stained with optimized antibody concentrations for twenty minutes on ice. Antibodies of 4 colors were used to determine CD4 and CD8 T cell responses to HIV antigens including: CD3 (APC conjugated), CD4 (PerCP conjugated), CD8 (FITC conjugated), and a combination of antibodies to IL-2, and IFN-γ (both PE conjugated). Cells were analyzed with an LSRII flow cytometer (Becton Dickinson, San Diego, CA), and data analyzed with FlowJo software (Tree Star, Inc., San Carlos, CA). Analysis determined the percent of CD4 and CD8 populations with intracellular cytokine expression at levels above the background expression (anti-CD28/49D stimulation only). The definition of a positive ICS response for all peptide pools (CD4 and CD8) was 0.045%, except CD8 EnvC (0.07%) and CD8 GagB (0.058%).

#### Viral inhibition assay (VIA)

VIA was performed as described elsewhere [20], [21]. Briefly, PBMC were thawed, divided into two aliquots and expanded for 7 days with bi-specific CD3/CD8 or CD3/CD4 antibodies and IL-2 to generate target CD4 and effector CD8 T cells respectively, resulting in expansion and enrichment (>90% of the CD3 T cells in the culture) of the required CD4 or CD8 T cell population. Separate cultures were established containing target CD4 T cells alone infected with either vaccine-matched or mis-matched HIV-1 isolates at an MOI of 0.01, and virus-infected target cells co-cultured with autologous CD8 effector T cells from the baseline pre-vaccination blood draw or from each post vaccination time point to be assessed. To limit variation due to effects of the vaccine regimen on the CD4 target cells, a single population of target CD4 cells was generated for each individual; where available this cell population was generated from the baseline, pre-vaccination sample. Every 3–4 days, culture supernatant was removed and replaced with fresh media, and assessed for p24 content using a commercially available ELISA (Perkin Elmer, UK). CD8 T cell-mediated inhibition was expressed as log_10_ reduction in p24 content of day 13 CD8 and CD4 T cell co-cultures compared with CD4 T cells alone. Viruses used for assessment of inhibition were obtained from the National Institutes of Health AIDS Reference and Reagent Program (Gaithersburg, MD) and included the lab-adapted CXCR4-tropic clade B HIV-1-IIIB used as a reference strain with pre-defined criteria for positivity (>1.13 log_10_ units of inhibition) [21]. The primary isolates HIV-1-96ZM651 (clade C/R5-tropic) and HIV-1-98IN017 (C/X4) were also assessed.

### Sample size and Pause rules

The initial design called for 12 vaccine and 4 placebo recipients in each of the study arms (a total of 64 volunteers). The study was designed so that if there were no serious adverse events (SAE) related to the vaccine in 48 volunteers, the upper bound of 95% confidence interval was 0.06 for the probability that an SAE related to vaccine could have occurred. Following review of initial safety data of the candidate vaccines, Groups C and D were increased to 27 vaccine and 9 placebo recipients to better inform potential subsequent larger phase II studies. Thus, 104 volunteers were planned to provide enhanced precision of the observed point estimates of safety, tolerability and immunogenicity in volunteers who received the VRC DNA prime - rAd5 boost regimen. An over-enrollment of up to 10% was allowed, resulting in 114 volunteers overall.

The study stopping rules were to go into effect if there were one or more grade 4 adverse events that were judged as probably or definitely related to the study vaccine by the site clinician. If a grade 4 adverse event occurred that was possibly related to the study vaccine or a grade 3 adverse event was judged as definitely, probably or possibly related to the vaccine, the independent DSMB would be informed immediately and discuss whether or not the study should pause until more information could be collected.

### Interim analyses

Annual interim analyses were performed. Blinded summary tables and listings of adverse events, including solicited reactogenicity events, were presented to an independent DSMB.

### Randomization and Blinding

The randomization schedule was prepared by the statisticians at the Data Coordinating Center, The EMMES Corporation. The randomization list was sent to the site pharmacist of record for dispensing of vaccine and placebo in a double-blind fashion. Study site staff, volunteers, and laboratories remained blinded with respect to the allocation of placebo or vaccine.

### Statistical methods

Comparisons of reactogenicity and adverse events were made by assigning scores of 0, 1, 2 and 3 to severities of none, mild, moderate and severe. The Wilcoxon Rank sum test was used to compare 2 groups; the Kruskal-Wallis test was used to compare more than 2 groups. Fisher's exact test was used for 2×2 tables. These comparisons were based on the maximum severity per volunteer. Immunogenicity results were tested using Fisher's exact test (2 groups) or the Kruskal-Wallis test. All tests are 2-tailed; statistical significance is defined as a p-value of <0.05. Analyses were performed using SAS version 9.2, Cary, NC.

## Results

### Participant flow and Recruitment

**Figure 1:**
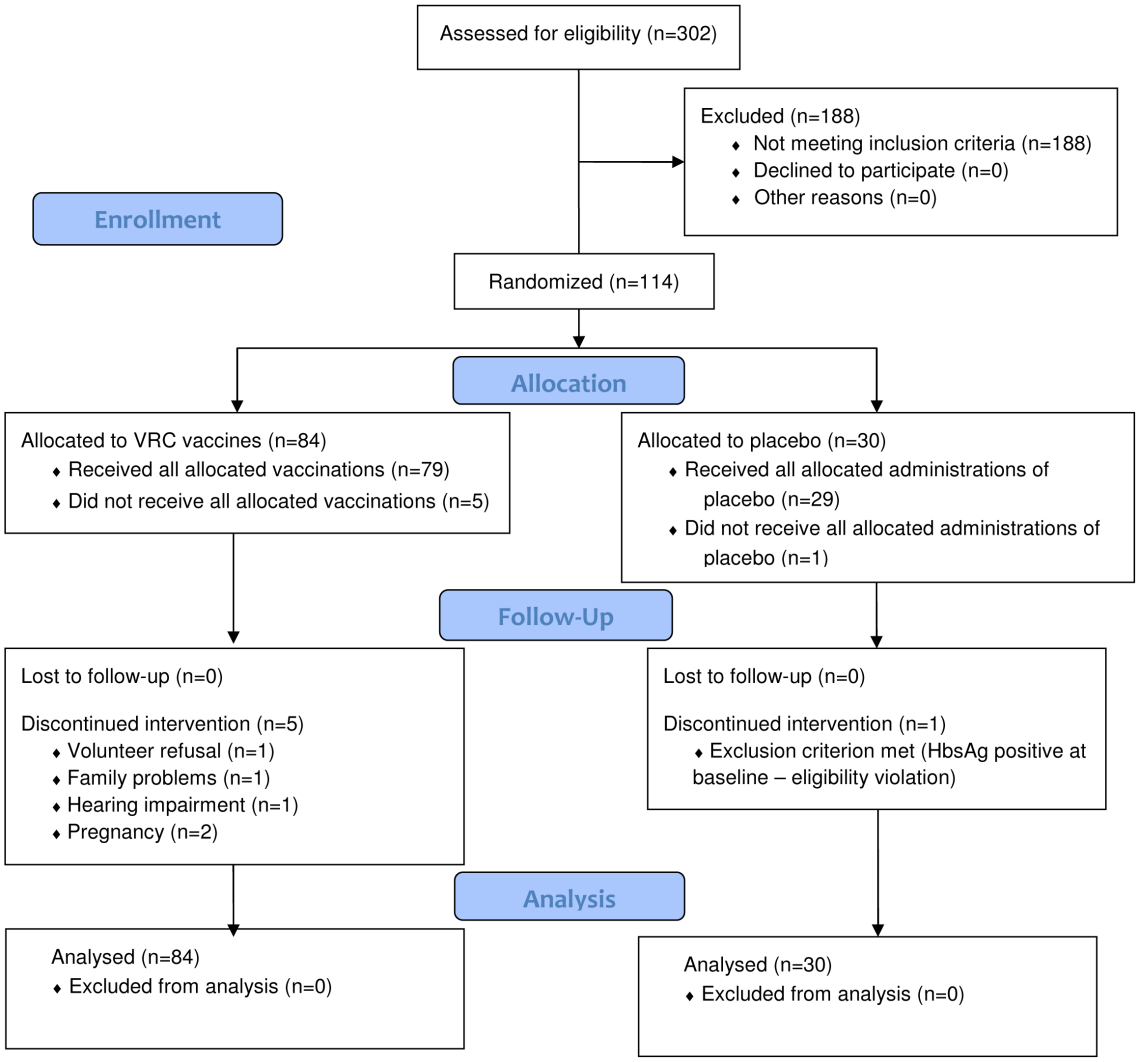
CONSORT Flow Diagram. Number of individuals assessed for eligibility, enrolled and randomized to study vaccine(s) and respective placebo, followed-up and analyzed.

As shown in Figure 1, 302 individuals were screened, of whom 114 volunteers (71 males, 43 females) were enrolled between November 2005 and May 2006. Table 2 shows the demographics by group assignment. Ninety-four percent of all volunteers completed the assigned administrations. No discontinuation was due to vaccine-related adverse events. Follow-up was completed in April 2007.

**Table 2:** Demographics, by Group Assignment.

Category	Placebo Recipients			Vaccine Recipients		
	Group A/B^a^ N = 9	Group C/D^b^ N = 21	Overall N = 30	Group A/B^a^ N = 26	Group C/D^b^ N = 58	Overall N = 84
**Gender**						
Male	6 (66.7%)	15 (71.4%)	21 (70.0%)	19 (73.1%)	31 (53.4%)	50 (59.5%)
Female	3 (33.3%)	6 (28.6%)^c^	9 (30.0%)	7 (26.9%)	27 (46.6%)^c^	34 (40.5%)
**Age (yrs)**						
Mean	28.5	28.4	28.4	27.5	26.3	26.7
Range	20.3–44.6	19.7–39.0	19.7–44.6	19.5–48.8	18.3–37.8	18.3–48.8
**Ad5 titers**						
# Non-Missing	7	21	28	26	57	83
Geometric Mean	489	338	370	341	288	303
Range	113–1819	16–3255	16–3255	16–4679	16–5000	16–5000
**Completed Vaccination**	9/9 (100%)	20/21 (95%)	29/30 (97%)	26/26 (100%)	53/58 (91%)	79/84 (94%)

a Groups A/B: rAd5 or Placebo alone.

b Groups C/D: DNA prime - rAd5 boost or Placebo - Placebo.

c In Groups C/D, the number of vaccinated women is not statistically different from female placebo recipients.

### Protocol Deviations

There were 59 minor protocol deviations, mainly procedural errors or study visits occurring outside the specified window period. There was one violation of an exclusion criterion: a volunteer, enrolled and randomized to Group C, received one injection of study vaccine prior to availability of the result of hepatitis B surface antigen (HbsAg), which was positive. Further vaccinations were discontinued. Individual unblinding at study end revealed that this volunteer was a placebo recipient. The interpretation of the data presented here is not affected by the protocol deviations.

### Vaccine Safety

#### Solicited events

**Figure 2:**
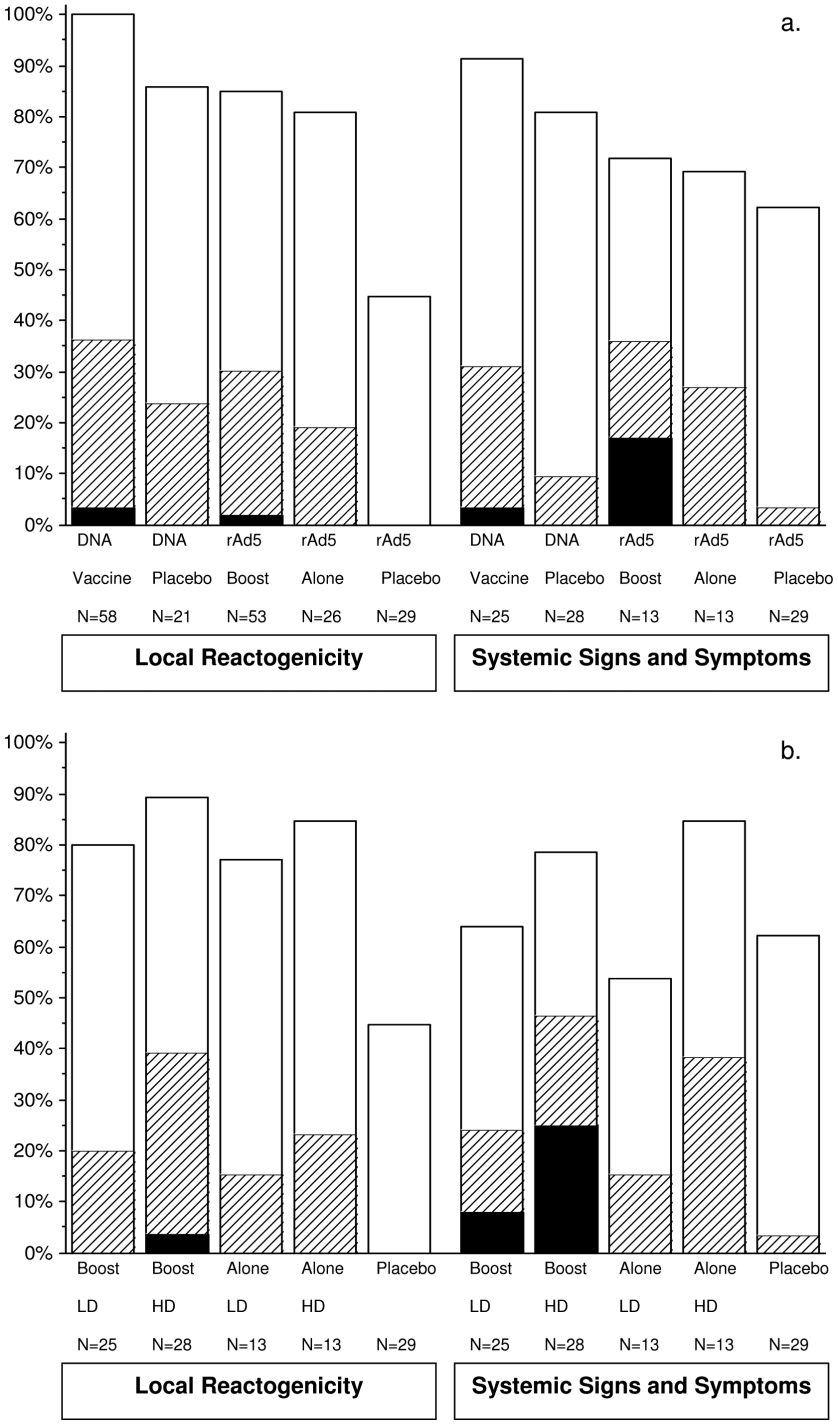
Solicited Local and Systemic Events. Figure 2a; Local reactogenicity and systemic signs and symptoms collected over 3 days post vaccination. Maximum severity of local reactions was significantly greater after rAd5 than after placebo (p = 0.006 and p<0.001 for rAd5 alone and rAd5 boost, respectively). For systemic signs and symptoms, the severity was significantly greater after DNA (p = 0.036) and after rAd5 boost (p = 0.028), than after the corresponding placebo. Figure 2b; Local reactogenicity and systemic events post rAd5 by dosage. Combining low dosage (LD) groups and high dosage (HD) groups, the maximum systemic reaction per volunteer post rAd5 was significantly higher (p = 0.025) in the HD group than in the LD group (41% versus 21%, respectively, were moderate or severe). Mild: open bars; Moderate: cross-hatched bars; and Severe: dark bars.

**Figure 2a** shows local and systemic reactogenicity to the individual vaccines and the respective placebo during the 3 days post-vaccination. **Figure 2b** shows local and systemic reactogenicity following rAd5 alone and rAd5 boost by rAd5 dosage.

#### Local Reactogenicity

Local reactions (pain, tenderness and vaccination site reactions/lesions) were reported by 92%, 85% and 100% of volunteers following rAd5 alone, rAd5 boost and DNA injections (all 3 combined), respectively, and by 44%, 45% and 86% following the respective placebo injections. The most common local reactions were mild (reported by 55.3% of all volunteers), followed by moderate (33.3%) and severe (2.6%). Two volunteers reported severe tenderness upon touch after the 1^st^ and 2^nd^ DNA vaccinations, respectively; a third volunteer reported severe pain and tenderness after the rAd5 boost. Two volunteers had moderate induration after the 1^st^ DNA vaccination, and two had moderate edema after DNA placebo and rA5 boost, respectively. There were no other moderate or greater local reactions. No papules, blisters/vesicles or scabs were observed.

Overall, the maximum severity per volunteer was significantly greater after rAd5 than after the corresponding placebo (p = 0.006 and p<0.001 in Groups A and B and Groups C and D, respectively). However, there was no significant difference between DNA and corresponding placebo (p = 0.137). The maximum severity per volunteer over all local reactions was not associated with dosage (p = 0.301 and p = 0.111 in Groups A and B and Groups C and D, respectively), and there was no significant difference between rAd5 alone or rAd5 boost (p = 0.716).

#### Systemic signs and symptoms

Systemic reactions were reported by 69%, 72% and 91% of volunteers following rAd5 alone, rAd5 boost and DNA injections (all 3 combined), respectively, and by 78%, 55% and 81% following the respective placebo injections. The most common systemic reactions were mild (reported by 49.1% of all volunteers), followed by moderate (25.4%) and severe (9.6%). Headache, malaise and/or fatigue were most commonly reported. Eleven vaccine recipients experienced severe events, lasting no more than 1–2 days. One volunteer reported severe headache, one severe malaise following the first DNA injection and 9 volunteers (2 from Group C, 7 from Group D) reported severe reactions following rAd5 boost: headache (n = 2); malaise (n = 1); myalgia (n = 1); elevated temperature (n = 2); arthralgia + malaise (n = 1); malaise + headache (n = 1); and malaise + myalgia + headache + arthralgia (n = 1). No severe or greater systemic symptoms were reported after rAd5 alone or in placebo recipients. No nausea or vomiting was reported.

Overall, the maximum severity per volunteer was not significantly different between rAd5 alone and placebo in Groups A and B (p = 0.566). However, in Groups C and D combined, the maximum severity was higher in the vaccines than the placebo group (p = 0.036 and p = 0.028 after any DNA injections and the rAd5 boost, respectively).

Comparing the two dosage levels of rAd5, the maximum severity per volunteer over all systemic reactions was not associated with dosage (p = 0.092 and p = 0.126 after rAd5 alone {Groups A versus B} and rAd5 boost {Group C versus D}, respectively); nor was there a significant difference between rAd5 alone (Groups A and B combined) or rAd5 boost (groups C and D combined) (p = 0.444). If the LD groups (A and C) are combined and the HD groups (B and D) are combined, then the maximum systemic reaction per volunteer post rAd5 is significantly higher (p = 0.025) in the HD group than in the LD group (41% versus 21%, respectively, were moderate or severe).

#### Reactogenicity by Ad5 neutralizing antibody titer at baseline

The severity of local reactions tended to be highest in subjects with baseline Ad5 neutralizing antibody titers to Ad5<19, lower in those with titers >200, and lowest in volunteers with titers between 19–200, but the differences were not statistically significant. Baseline Ad5 neutralizing antibody titers had no effect on systemic signs and symptoms. (**Figures S1 and S2**).

#### Unsolicited adverse events

During the 12-month observation period, 777 unsolicited AEs were reported, of which 344 occurred within 28 days after any injection. **Table 3** shows AEs by severity and dosage group. 271/344 (79%) were graded as mild, 66 (19%) as moderate and 7 (2%) as severe. Overall, there were no differences in the incidence or severity of unsolicited AEs, i.e., between rAd5 and placebo, rAd5 low and rAd5 high dosage, or rAd5 alone and rAd5 boost, whether within 28 days or at any time. Of 266 AEs occurring in vaccine recipients within 28 days after vaccination, 42 (15.8%) were judged possibly or probably related to study vaccines. The most frequent of these were laboratory abnormalities (see next section) and influenza-like syndromes. Similarly, of 78 AEs occurring in placebo recipients within 28 days after administration, 15 (19.2%) were judged related to study products before unblinding. These included neutropenia, leucopenia, urticarial rash and influenza-like syndrome. No epidemiological data are available on whether or not influenza virus was circulating in the community at the time these adverse events occurred. No blood samples were taken at the time of events that were compatible with adenoviral infections to look for serological evidence of infection with Ad5. Six events of urticarial rash were reported in 5 volunteers at PSF; all resolved spontaneously. Two events were moderate, one occurring 10 days after the 1^st^ DNA and the other 71 days after the rAd5 boost. Two events were mild, one occurring 48 days after the 3^rd^ DNA and the other 276 days after rAd5 alone. The remaining 2 events were mild and occurred in the same placebo volunteer (5 days after the 1^st^ DNA and on the day of the 3^rd^ DNA). All these events were described as typical urticarial rashes. Furthermore, in vaccine recipients the intervals between occurrence and the most recent vaccination did not clinically support “immediate hypersensitivity reaction” as a diagnosis. None of the rashes was accompanied by other allergic reactions, nor were any vaccinations discontinued as a result.

**Table 3:** Number of Unsolicited Adverse Events within 28 Days Post Any Vaccination, by Severity and Group.

Group	Treatment	Grade 1 Mild	Grade 2 Moderate	Grade 3 Severe	Total AEs
rAd5 Alone	Low Dosage	12 (80.0%)	3 (20.0%)	0 (0.0%)	15
	High Dosage	9 (90.0%)	1 (10.0%)	0 (0.0%)	10
	Any Dose	21 (84.0%)	4 (16.0%)	0 (0.0%)	25
	Placebo	8 (57.1%)	6 (42.9%)	0 (0.0%)	14
3DNA*	Vaccine	143 (75.7%)	41 (21.7%)	5 (2.6%)	189
	Placebo	45 (88.2%)	6 (11.8%)	0 (0.0%)	51
rAd5 Boost	Low Dosage	22 (91.7%)	2 (8.3%)	0 (0.0%)	24
	High Dosage	22 (78.6%)	5 (17.9%)	1 (3.6%)	28
	Any Dose	44 (84.6%)	7 (13.5%)	1 (1.9%)	52
	Placebo	10 (76.9%)	2 (15.4%)	1 (7.7%)	13

*AEs occurring within 28 days of any DNA or placebo injection (given at W 0, 4 and 8) combined.

#### Laboratory abnormalities

Laboratory abnormalities were mostly mild, with neutropenia (absolute counts) being the most frequent. Moderate neutropenia (750 to <1000 cells/µL) occurred in 6 (7.1%) of the 84 vaccine recipients and 3 (10%) of 30 placebo recipients. One severe (grade 3) neutropenia (740 cells/µL) was detected 2 weeks after the 2^nd^ DNA injection, and one grade 4 neutropenia (450 cells/µL) was detected in an asymptomatic volunteer 11 months after rAd5 alone. Two volunteers had a moderate decrease of hemoglobin (6 months after the 1^st^ DNA placebo and 3 months after rAd5 boost). There were 2 isolated, moderate ALT elevations, at 6 months after rAd5 alone and 3 months after rAd5 boost. There were no other moderate or greater laboratory abnormalities.

#### Serious Adverse Events

During the study, 6 serious adverse events (SAEs) occurred in 6 vaccine recipients. These events were cesarean section, complete abortion, phlebitis, discal hernia, pelvic inflammatory disease, and fracture of the middle finger. None of these events was considered related to study product.

#### Pregnancies

Three women became pregnant during the study. One had completed her vaccination regimen before getting pregnant and delivered a healthy baby. One woman, who had no history of spontaneous abortion, was found to be pregnant 4 weeks after the 1^st^ DNA injection and had a complete spontaneous abortion one month later. The third woman delivered a term male child by cesarean section due to cephalo-pelvic disproportion 41 weeks after the 3^rd^ DNA injection. No abnormalities were detected either at birth or at 14 weeks of age. However, at the age of 12 months the child was found to have a congenital malformation (a large arachnoidal cyst with agenesis of corpus callosum and a parieto-occipital calvarial defect) and underwent endoscopic fenestration and is now reaching age-appropriate developmental milestones. The abnormality was assessed as probably not related to study vaccine and was defined as “a potential relationship between study agent and the adverse event could exist (i.e., the possibility cannot be excluded), but the adverse event is most likely explained by causes other than the study agent”.

#### Incident HIV-infection

There was no intercurrent HIV infection during study follow up. One Group A volunteer acquired HIV about 2 years after receipt of rAd5 alone, i.e., one year after final study visit. At the time of diagnosis – six weeks after the estimated date of infection - the viral load was 929 HIV RNA copies/mL (2.97 logs). The baseline Ad5 neutralizing antibody titer was 830. This volunteer is currently being followed in a long-term follow up study specifically designed for HIV vaccine recipients from any IAVI - sponsored clinical trial who acquire HIV any time during or up to 2 years after the end of a study.

### Vaccine Immunogenicity

#### IFN-γ ELISPOT responses

The magnitude, response rate and breadth of HIV-1 specific T-cell responses in each group and the possible impact of pre-existing Ad5 antibodies on these responses were assessed using an IFN-γ ELISPOT assay.

#### Immune responses after rAd5 alone (Groups A and B)

Six weeks after administration of rAd5 1×10^10^ and rAd5 1×10^11^, 6/13 (46%) and 7/13 (54%) volunteers, respectively, had one or more positive responses by IFN-γ ELISPOT to any pool (**Table 4**). There were no responders among the placebo recipients. The response rate in vaccine recipients was similar in the two dosage groups (2-sided Fisher's exact test p-value>0.9), and significantly higher than in placebo recipients (1-sided Fisher's exact test p-value<0.001). At the 48-week time point (final study visit) there were 4/13 (31%) and 5/13 (38%) responders in Groups A and B, respectively, all but one of whom were also positive at 6 weeks.

**Table 4:** IFN-γ ELISPOT Response Rates at Single Post Vaccination Time Points.

			ELISPOT Responses^a^		
Group	Dose	Post Vaccination Time Point	Low Dosage	High Dosage	Total
**A and B**	**rAd5 Alone**	**Pre-Vaccination**	0/13 0% (0%–25%)	1/12 8% (0%–38%)	1/25 4% (0%–20%)
		**6 Weeks**	6/13 46% (19%–75%)	7/13 54% (25%–81%)	13/26 50% (30%–70%)
		**36 Weeks**	4/13 31% (9%–61%)	4/12 33% (10%–65%)	8/25 32% (15%–54%)
		**48 Weeks**	4/13 31% (9%–61%)	5/13 38% (14%–68%)	9/26 35% (17%–56%)
**C and D**	**3DNA Prime**	**Pre-Vaccination**	0/29 0% (0%–12%)	1/28 4% (0%–18%)	1/57 2% (0%–9%)
		**4 Weeks**	11/28 39% (22%–59%)	13/29 45% (26%–64%)	24/57 42% (29%–56%)
	**rAd5 Boost**	**6 Weeks** b	18/25 72% (51%–88%)	18/26 69% (48%–86%)	36/51 71% (56%–83%)
		**12 Weeks** b	18/27 67% (46%–83%)	18/27 67% (46%–83%)	36/54 67% (53%–79%)
		**24 Weeks** b	22/27 81% (62%–94%)	20/25 80% (59%–93%)	42/52 81% (67%–90%)

a Frequency (# Subjects) and percent (exact 95% confidence interval).

b Time interval given is post rAd5 boost.

#### Immune responses after DNA prime – rAd5 boost (Groups C and D)

At 4 weeks after the 3^rd^ dose of DNA, and prior to the rAd5 boost, 42% of vaccine recipients from both groups combined had one or more positive responses (**Table 4**). After the rAd5 boost, 72% and 69% of vaccine recipients had positive responses at 6 weeks, and 81% and 80% had positive responses at 6 months in Groups C and D respectively. Neither of these differences was statistically significant. The highest frequency of immune responses was observed at 24 weeks post rAd5 boost, which was the final follow up study visit. In both groups C and D the same number of volunteers, 25/29 (86%), had at least one positive response at one or more visits after the initial vaccination. Of the 36 volunteers with positive responses 6 weeks after the rAd5 boost, 33 (92%) were still positive at week 24, with responses to EnvA and EnvB being the most persistent (24/29 and 21/24, respectively, in the low and high dosage groups).

#### Response rates to rAd5 alone compared to DNA prime - rAd5 boost

**Figure 3:**
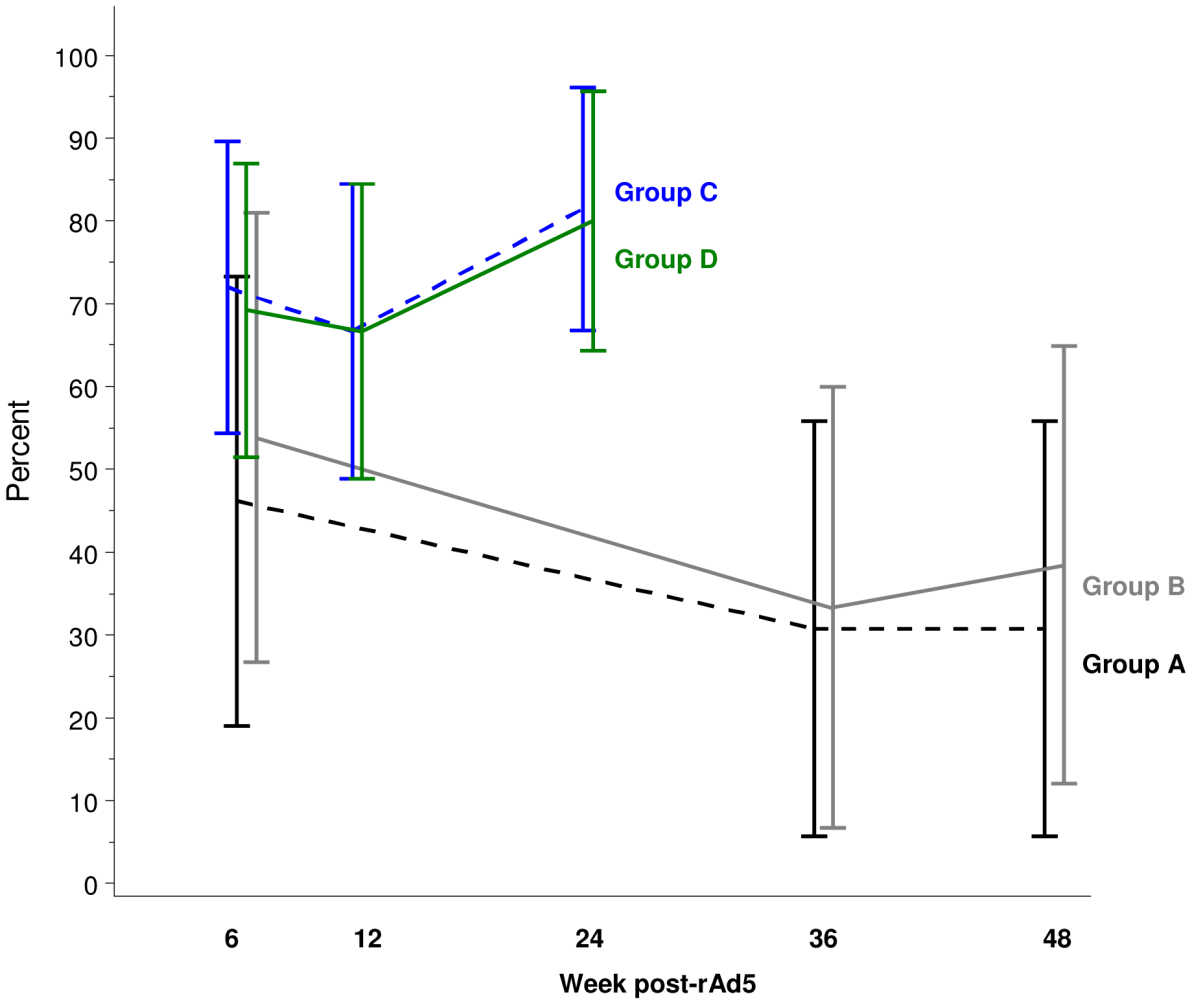
Impact of DNA prime on ELISPOT responses. Comparison of percent ELISPOT responders after rAd5 alone (Groups A and B) versus DNA prime - rAd5 boost over time (Groups C and D). Vertical lines represent 95% Confidence Intervals.

**Figure 3** and **Table 4** show that at 6 weeks post-vaccination with rAd5, there was no significant difference in the overall response rates between Groups A and B and Groups C and D (50% versus 71%,p = 0.0862). At the final study visit, which occurred 48 weeks after rAd5 alone and 24 weeks after rAd5 boost, the prime-boost group had a much higher response rates for IFN-γ ELISPOT (35% versus 81%, p<0.001, Fisher's exact 2-tailed: 9/26 versus 42/52). The proportions of volunteers with positive ELISPOT responses at 12 and 24 weeks post rAd5 boost are higher than at 36 and 48 weeks after rAd5 alone. However, it is unknown whether the high proportions in Groups C and D were maintained up to 36 and 48 weeks, or whether the proportions in Groups A and B at weeks 12 and 24 were higher than at week 36, since no blood samples were taken at those time points in the respective groups.

#### Magnitude of responses

**Figure 4:**
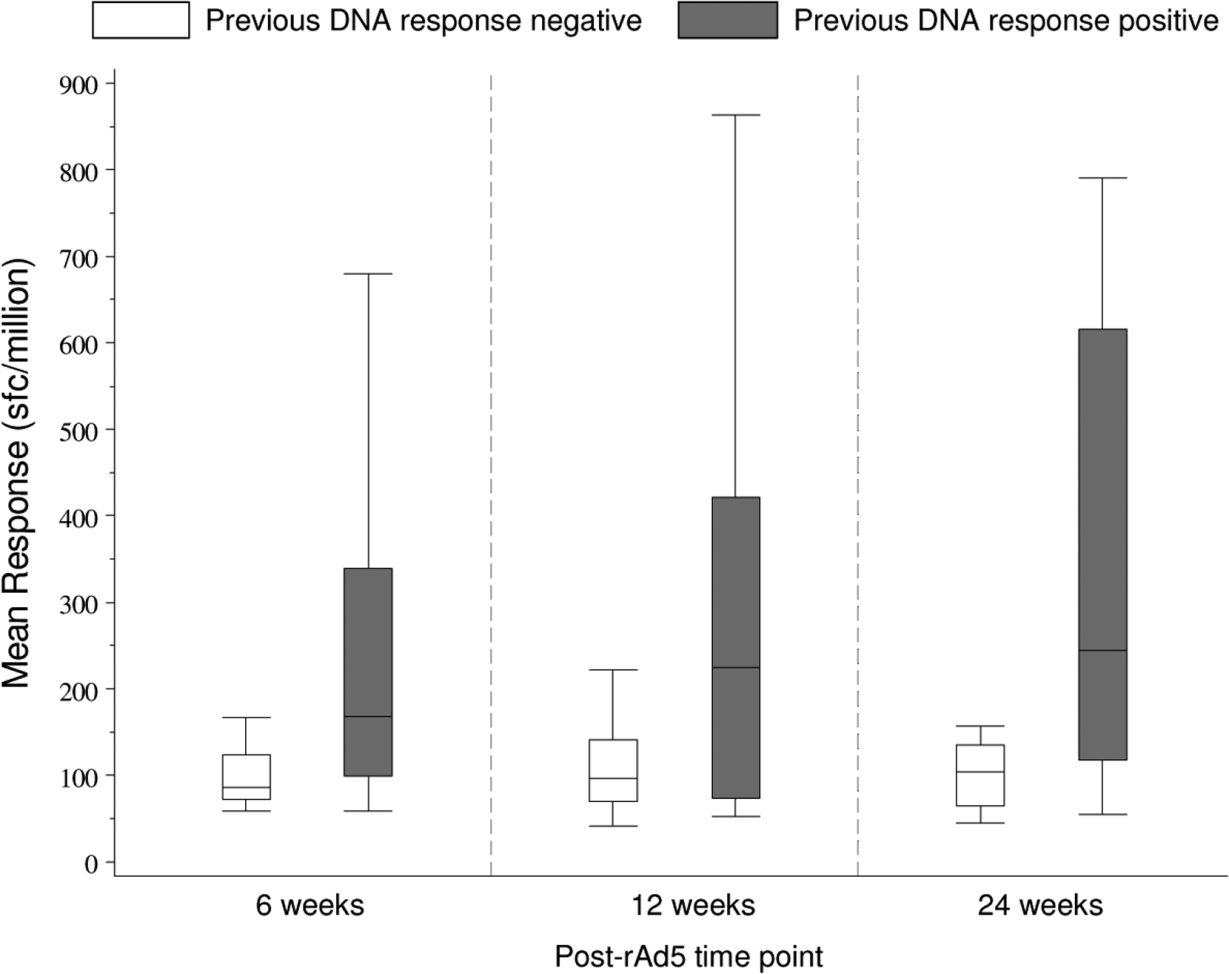
Impact of DNA prime on ELISPOT magnitude. Values plotted are the mean background-subtracted ELISPOT counts (Spot Forming Cells, SFC) over all positive peptide responses per volunteer at that visit. Shaded bars are subjects with any positive response at 4 weeks post 3^rd^ DNA, white boxes are those with no positive responses at this time point.

The median (range) background-subtracted number of SFC per 10^6^ PBMC from responders 6 weeks after vaccination with rAd5 alone is 85 (52–297) for the low and 78 (39–230) for the high dosage groups, respectively. Similarly, 6 weeks after the rAd5 boost the median (range) SFC counts are 91 (45–1420) and 105 (41–1707) per 10^6^ PBMC, respectively (see also below). The overall differences between dosage groups after rAd5 alone or rAd5 boost were not statistically significant. However, at each visit, the average magnitude of positive responses per volunteer after the rAd5 boost was statistically significantly greater in those who responded to the DNA vaccine compared to those who did not (**Figure 4**).

#### IFN-γ ELISPOT responses by antigen

**Figure 5:**
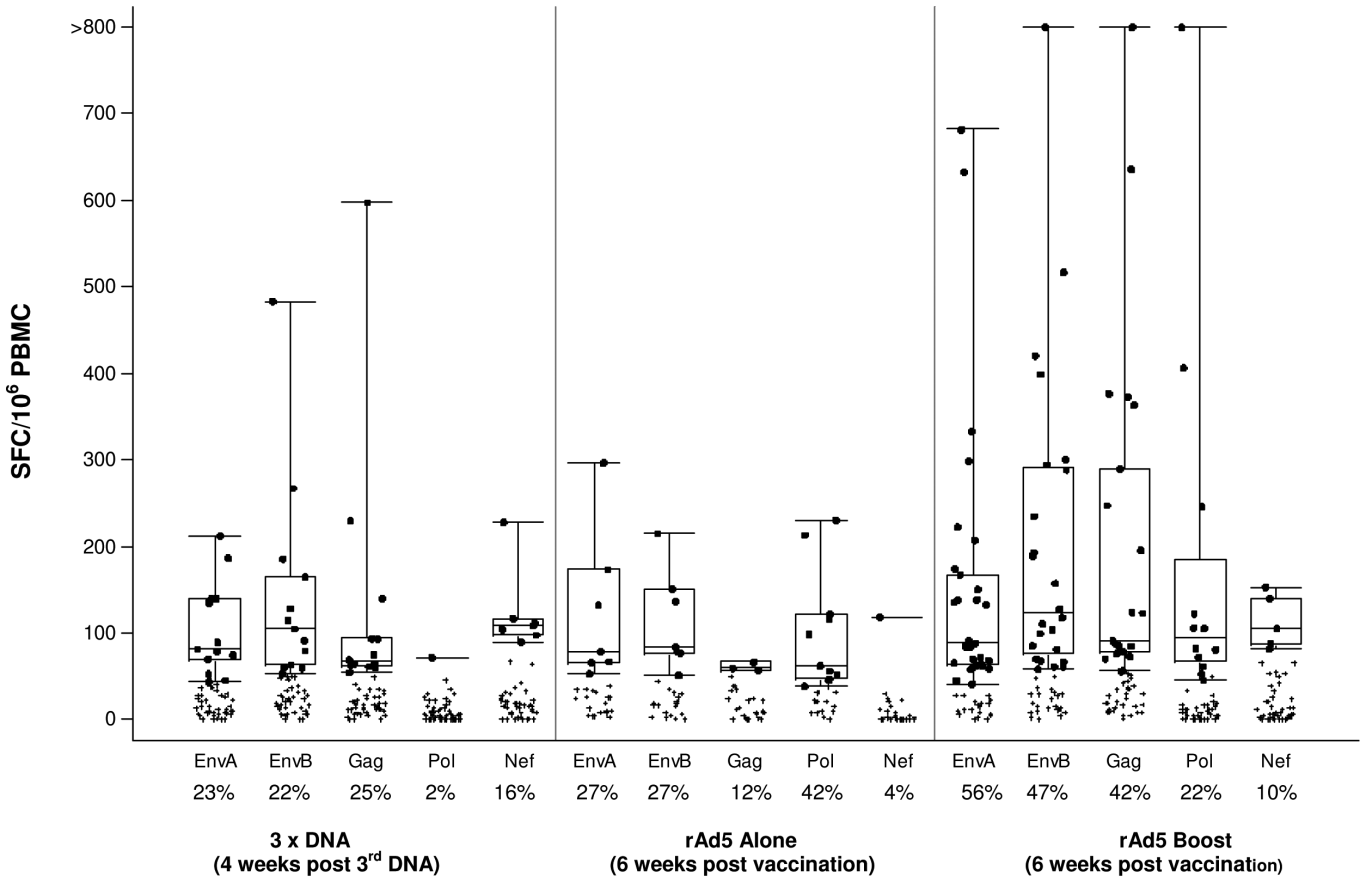
Magnitude of ELISPOT and % response rate. The magnitude of the response was measured by SFC per million peripheral blood mononuclear cells (SFC/m PBMC). The x-axis row one shows the peptide pool, row two the % response rate and row three the vaccine group. Black dots indicate positive responses, defined by background-subtracted values greater than the cutoff, more than 3 times mean background SFC count, and a coefficient of variation of not more than 70% amongst replicate wells. The rAd5 response rates correspond to both dose groups combined. The box plots summarize positive responses only (i.e., median, 1^st^ and 3^rd^ quartiles, minimum/maximum). The cut-offs for EnvA, EnvB, Gag, Nef, PolB1 and PolB2, as determined by the level of non-specific responses from at least 180 samples from unvaccinated individuals, are 40, 51, 54, 68, 51 and 38 respectively. The Pol response is the maximum of PolB1 and PolB2 and positive if either one is positive.

Six weeks after rAd5 alone there were no statistically significant differences between the low and high dosage groups in the proportions of volunteers responding to Pol, EnvA, EnvB or Gag. Similar results were obtained, including responses to Nef, six weeks after the rAd5 boost (**Table 5**). There was one false positive response to Nef in a Group B volunteer, who received a single dose of the rAd5 vaccine, which does not contain the Nef gene. Six weeks after rAd5 alone, the most frequent responses were to Pol followed by Env and then Gag, while 6 weeks after the rAd5 boost, the most frequently observed responses were to Env, followed by Gag and then Pol (**Figure 5**). The proportion of vaccine recipients with IFN-γ ELISPOT responses to at least 2 antigens was greater in those receiving the high versus low dosage of rAd5 alone (54% vs. 23% respectively, for Groups B and A, p = 0.226). After 3 injections of DNA, the proportion of vaccine recipients with responses to multiple antigens was 21% (Groups C and D combined). After rAd5 boost, 62% and 66% of DNA primed vaccinees in the LD and HD groups, respectively, had a positive response (**Figure 5**). Comparing LD rAd5 alone with LD rAd5 boost, the response rates were 23% versus 62% (p = 0.043), for HD rAd5 alone versus HD rAd5 boost, the response rates, respectively, were 54% and 66% (p = 0.510).

**Table 5:** Percent of Volunteers with Positive IFN-γ ELISPOT Response to Vaccine Antigens.

Group		Env A	Env B	Gag	Nef	Pol	Any
**A**	**6 Weeks Post rAd5 Alone**	23.1	23.1	7.7	0.0	38.5	46.2
**B**	**6 Weeks Post rAd5 Alone**	30.8	30.8	15.4	7.7	46.2	53.8
**C**	**4 Weeks Post 3DNA**	25.0	22.2	17.9	18.5	3.6	39.3
	**6 Weeks Post rAd5 Boost**	64.0	54.2	40.0	8.3	20.0	72.0
**D**	**4 Weeks Post 3DNA**	20.7	24.1	31.0	7.4	0.0	44.8
	**6 Weeks Post rAd5 Boost**	50.0	42.3	42.3	11.5	26.9	69.2
**Total**	**4 Weeks Post 3DNA**	**22.8**	**23.2**	**24.6**	**13.0**	**1.8**	**42.1**
	**6 Weeks Post rAd5 Alone**	**26.9**	**26.9**	**11.5**	**3.8**	**42.3**	**50.0**
	**6 Weeks Post rAd5 Boost**	**56.9**	**48.0**	**41.2**	**10.0**	**23.5**	**70.6**

DNA priming improved the proportion of vaccine recipients with any positive response at more than one time point after the rAd5 vaccination, with 31, 54, 69 and 64% in Groups A, B, C and D, respectively. The difference was not statistically significant (p = 0.122 between all four groups, and p = 0.054 for Groups A and B versus Groups C and D, i.e., 42% versus 67%, respectively).

**Figure S3** shows the frequency and distribution of peptide pool responses over time in each group which provides an idea of the breadth of the response to the vaccine regimens. Six pools are included in the analysis; Gag, Nef, Env A and B and two Pol pools. Responses of up to 4 pools were commonly observed across all the groups and over multiple time points.

#### Ad5 neutralizing titers and relationship to IFN-γ ELISPOT responses

The distribution of baseline Ad5 titers is similar across Groups A to D (p = 0.332). There was no significant difference in response rate to vaccination in any group of vaccine recipients depending on baseline anti-Ad5 titers. (**Figure S4**). In individuals with a baseline Ad5 titer >200, the response rate in participants vaccinated with DNA prime - rAd5 boost was higher (30/35; 86%) than in those receiving rAd5 alone (10/18; 56%: p = 0.0220). There was no effect of dosage. The numbers of volunteers with baseline Ad5 neutralizing titer <19 and 19–200 who responded were too small for comparisons. Four weeks after administration of rAd5, the Ad5 antibody titer was significantly increased in all groups. Titers in Group D were significantly higher than in Group C (p = 0.0056) (data not shown).

#### Intracellular cytokine staining (ICS)

Samples from 13 vaccine and 5 placebo recipients from groups C and D were assessed using an IL-2/IFN-γ ICS assay at 2 weeks post DNA prime and 4 weeks post Ad5 boost. The percent response rates for ICS are shown in **Table 6**. CD4 T cell responses to Env, Gag and Nef were detected after DNA prime and after Ad5 boost, but no Pol specific responses were seen. CD8 T cell responses to Env, Nef and Pol were detected after DNA prime - rAd5 boost, with fewer responders seen after the DNA priming. One placebo recipient had a false positive response to the EnvC pool. The magnitude of cytokine secretion was modest; HIV-specific CD4 T cell responses to any antigen were all <0.3% and CD8 T-cell responses all <0.32% except for one Pol response of 1.33%. Because of the small numbers of samples per group, it was not possible to determine whether there were differences in the frequencies and magnitude of CD4 versus CD8 T cell responses.

**Table 6:** ICS Responses Post DNA Prime - rAd5 Boost.

	Number of responders/total tested (% responders)					
	Baseline	2 weeks post 3DNA		4 weeks post rAd5		
Peptide Pool	V02	Placebo	3DNA	Placebo	Low Dosage	High Dosage
**CD4**						
**Any Env**	0/13 (0.0%)	**1/5 (20.0%)**	**8/13 (61.5%)**	0/5 (0.0%)	**3/6 (50.0%)**	**5/7 (71.4%)**
**Env A**	0/13 (0.0%)	0/5 (0.0%)	**6/13 (46.2%)**	0/5 (0.0%)	**3/6 (50.0%)**	**4/7 (57.1%)**
**Env B**	0/13 (0.0%)	0/5 (0.0%)	**4/13 (30.8%)**	0/5 (0.0%)	**1/6 (16.7%)**	**1/7 (14.3%)**
**Env C**	0/13 (0.0%)	**1/5 (20.0%)**	**7/13 (53.8%)**	0/5 (0.0%)	**1/6 (16.7%)**	**5/7 (71.4%)**
**Gag B**	0/13 (0.0%)	0/5 (0.0%)	**8/13 (61.5%)**	0/5 (0.0%)	**2/6 (33.3%)**	**2/7 (28.6%)**
* **Nef B**	0/11 (0.0%)	0/5 (0.0%)	**2/11 (18.2%)**	0/5 (0.0%)	**1/6 (16.7%)**	0/5 (0.0%)
**Any Pol**	0/13 (0.0%)	0/5 (0.0%)	0/13 (0.0%)	0/5 (0.0%)	0/6 (0.0%)	0/7 (0.0%)
**CD8**						
**Any Env**	0/13 (0.0%)	0/5 (0.0%)	**3/13 (23.1%)**	0/5 (0.0%)	**4/6 (66.7%)**	**3/7 (42.9%)**
**Env A**	0/13 (0.0%)	0/5 (0.0%)	**2/13 (15.4%)**	0/5 (0.0%)	**4/6 (66.7%)**	**3/7 (42.9%)**
**Env B**	0/13 (0.0%)	0/5 (0.0%)	**3/13 (23.1%)**	0/5 (0.0%)	**2/6 (33.3%)**	**3/7 (42.9%)**
**Env C**	0/13 (0.0%)	0/5 (0.0%)	0/13 (0.0%)	0/5 (0.0%)	0/6 (0.0%)	0/7 (0.0%)
**Gag B**	0/13 (0.0%)	0/5 (0.0%)	0/13 (0.0%)	0/5 (0.0%)	0/6 (0.0%)	**1/7 (14.3%)**
* **Nef B**	0/13 (0.0%)	0/5 (0.0%)	**2/13 (15.4%)**	0/5 (0.0%)	**1/6 (16.7%)**	0/7 (0.0%)
**Any Pol**	0/13 (0.0%)	0/5 (0.0%)	**1/13 (7.7%)**	0/5 (0.0%)	**1/6 (16.7%)**	**3/7 (42.9%)**

*Two volunteers had CD4 responses to Nef at all 3 visits. These Nef responses have been excluded.

#### Viral Inhibition Assay

**Figure 6:**
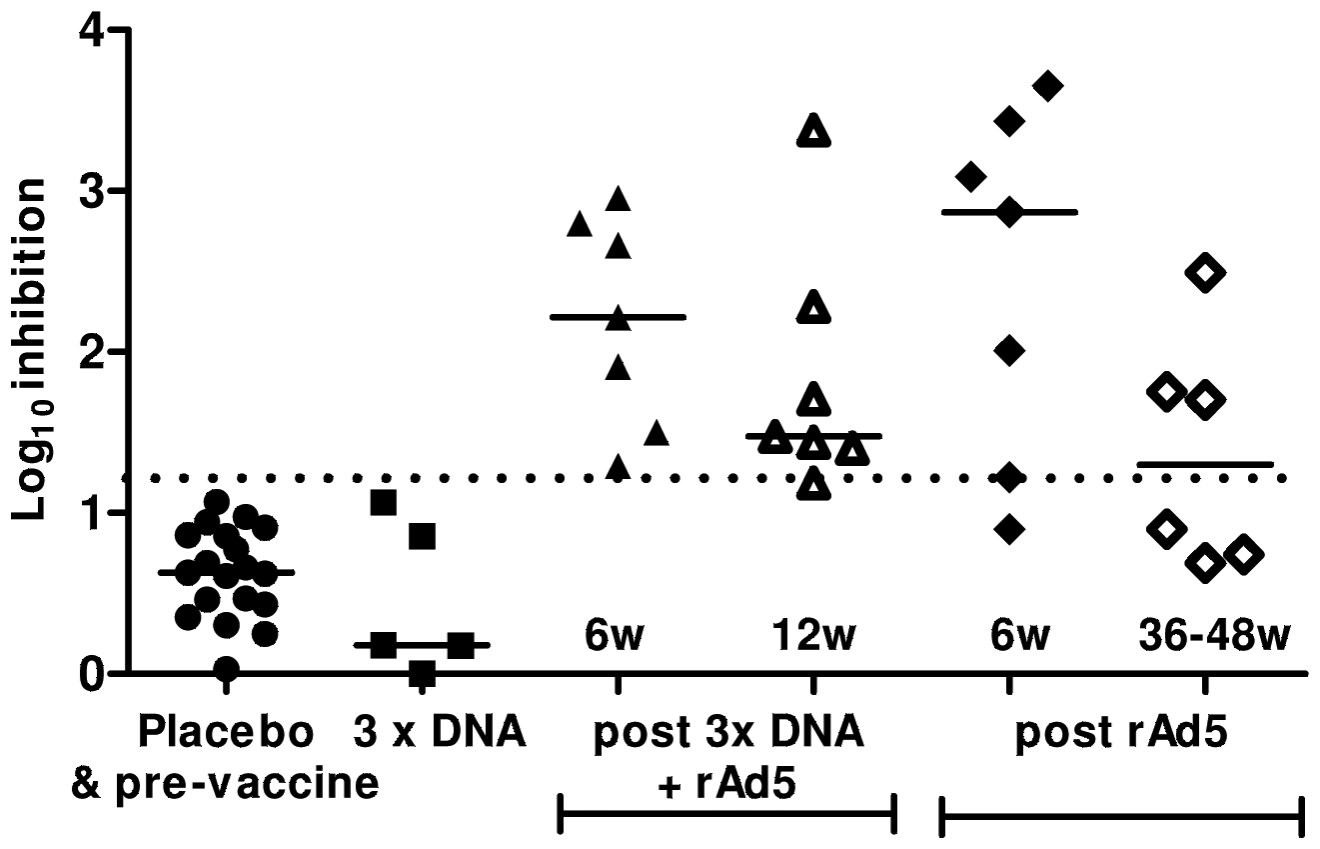
Induction of CD8 T cell-mediated inhibition of HIV-1-IIIB replication following vaccination with a DNA prime - rAd5 boost or rAd5 alone regimen. Viral inhibition was assessed in placebo recipients and prior to any vaccination (•); following DNA alone (▪); DNA prime - rAd5 boost at 6 and 12 weeks (▴and ▵respectively) and after rAd5 alone at 6 and between 36 to 48 weeks (♦ and ⋄ respectively). The 1.13 log_10_ inhibition value above which inhibition is considered HIV-1 specific is indicated by the hashed line. Medians for all groups are indicated (—).

A viral inhibition assay (VIA) has previously been optimized and qualified using PBMC from HIV-infected individuals with a range of viral loads (VL) and uninfected low risk volunteers. To determine the criteria for assay positivity, viral inhibition from 43 HIV–uninfected subjects was assessed. A cut-off of >1.13 log_10_ viral inhibition was established based on a median of 0.70 log_10_ inhibition of p24 release from HIV-1IIIB-infected CD4 T cells with >99% confidence. With a qualified viral inhibition assay, HIV seropositive individuals had a range of responses with a trend toward higher viral inhibition in those with lower viral loads. Treatment-naive HIV+ subjects with a plasma VL of <10 000/mL had median viral inhibition of 3.17 log_10_, whereas those with VL >10000 had median viral inhibition of 1.12 log_10_ [21]. To assess how well the VIA works for vaccine trials, seven samples from vaccinees who received DNA prime - rAd5 boost and one sample from a placebo recipient were tested and the results were recently published [21]. To extend these findings, viral inhibition was assessed in seven individuals at 6 weeks and between 36–48 weeks after a single dose of rAd5 and in a further 2 placebo individuals. The samples from vaccine recipients were selected based on a range of positive ELISPOT responses, from low (close to the cut-off) to high SFC counts, and placebo samples were randomly selected. The laboratory performing the assays remained blinded. **Figure 6** shows data from a total of 14 vaccine and 3 placebo recipients. All of the participants with an available pre-vaccination specimen (2 placebos and 10 vaccinees) classified as negative (<1.13 log_10_) for HIV-1_IIIB_ inhibition. At 4 weeks post-3^rd^ DNA vaccination, PBMC were available from 5 of the 7 vaccinees, and all were negative for inhibition. Six and 12 weeks post rAd5 boost, CD8 T-cells from all seven vaccinees efficiently inhibited HIV-1-IIIB (median 2.22 and 1.48 log_10_). At 6 weeks post rAd5 alone, 6 of the 7 vaccinees efficiently inhibited HIV-1_IIIB_ (median 2.87 log_10_). Between 36–48 weeks post rAd5 alone, viral inhibition was still present in 3 out of 6 vaccinees who had viral inhibition detected at week 6, one sample was unavailable for testing. There was no difference between VIA after Ad5 boost versus Ad5 alone at 6 weeks post-vaccination or any VIA post-Ad5 boost (n = 14) versus any post-Ad5 alone (n = 13) (p = 0.534 and p = 0.792 respectively, Wilcoxon test).

VIA activity was found in the presence of moderate to high Ad5 neutralization titers. At 4 weeks post rAd5, Ad5 neutralization titers were available from 13 of the 14 vaccinees, 9 of the 13 individuals had titers >8748 and 4 had titers >200 (1305–2667). No samples were available from individuals with Ad5 titers of <200.

#### HIV specific antibody responses

**Figure 7:**
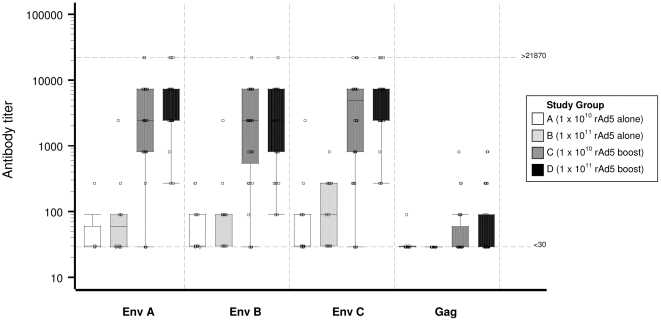
HIV antibody titers. Distribution of HIV-specific antibody titers at 1 month post rAd5 alone (Groups A and B) and rAd5 boost (Groups C and D) in vaccine recipients, by protein and treatment group. The Y-axis shows the antibody titer on a log scale with box plots showing the median, 1^st^ and 3^rd^ quartiles.

After rAd5 alone, antibodies to EnvA, EnvB and EnvC were detected in, respectively, 25%, 45% and 42% of volunteers in Group A and in 50%, 58% and 58% of volunteers in Group B. After the rAd5 boost, the response to each Env protein was 89% in Group C and 100% in Group D. There was no effect of dosage. Antibodies to Gag were detected after the LD and HD rAd5 boost in 25% and 31% of volunteers, respectively, compared to 8% and 0% after rAd5 alone, whereas antibodies to pol and nef were detected infrequently in all groups (data not shown). None of the placebo group had antibody responses to the HIV antigens. Antibody ELISA titers are shown in **Figure 7** post rAd5 for each of groups A–D. Comparison of ELISA titers between the 4 study groups was made separately within each of the 4 peptides Env A, Env B, Env C and Gag using the non-parametric Wilcoxon 2-sample test. Allowing for multiple comparisons, a p-value of less than 0.025 is required for an overall significance level. For Env A, B and C, the ELISA titers in groups C and D were significantly higher (p<.0001) than in groups A and B. There were no statistically significant differences between Gag titers. The median antibody ELISA titers after DNA alone were <30 and below the cut-off of the assay (data not shown).

#### HIV neutralizing activity

Only 2 individuals in Group D had moderate neutralizing activity against SF162 and no detectable neutralizing activity against 12 other HIV-1 isolates (data not shown). Similar HIV-1 binding ELISA and HIV neutralization activity was seen in individuals in a recently published study of the VRC DNA + Ad5 regimen [6]. No other samples showed neutralizing activity above cut-off.

#### HIV serology at the end of study

Twelve months after rAd5 alone and 6 months after the rAd5 boost, 10/13 (76%) and 48/57 (84%) vaccine recipients, respectively, had a positive HIV test result by at least one commercial HIV ELISA kit without being HIV-infected. Four months after 3 DNA injections and immediately prior to the rAd5 boost, only 6/57 (10%) vaccine recipients had a positive HIV test result. The Detect HIV v2 kit did not detect vaccine-induced antibodies in any vaccine recipient. 2/30 (7%) placebo recipients had a false positive HIV test result.

## Discussion

In this study, the VRC HIV-1 rAd5 vaccine was generally well-tolerated when given alone or as boost following the VRC HIV-1 DNA vaccine to healthy, HIV-seronegative African adults at low risk for HIV infection. The reactogenicity seen in this study was consistent with earlier phase I studies evaluating these products [2], [3], [4], [5].

The DNA prime - rAd5 boost vaccination schedule was highly immunogenic even in volunteers with neutralizing antibodies against Ad5 at baseline. IFN-γ ELISPOT responses were most frequent to Env and Gag epitopes and were maintained at least 6 months after the rAd5 boost. Overall, there was no dosage effect on the frequency of IFN-γ ELISPOT responses at 6 weeks after rAd5 alone or rAd5 boost.

Compared to rAd5 alone, DNA priming increased the frequency of IFN-γ ELISPOT response after the rAd5 boost, but the difference was not statistically significant 6 weeks after rAd5 administration. DNA priming also increased the magnitude and the durability of the T-cell responses and resulted in immunodominance of Env and Gag over Pol consistent with the results from the RV 172 study [8]. In our study, responses to Pol were highest after rAd5 alone. Contrary to our findings, responses to Env in RV 172 were not different between rAd5 alone and DNA prime – rAd5 boost groups [8]. These findings may be relevant for future antigen design of T cell based vaccines.

Vaccine-specific antibodies were detected to EnvA, EnvB and EnvC, and to Gag after DNA prime and rAd5 alone; after DNA prime - rAd5 boost, significantly higher magnitude of antibody titers and frequency of responders were detected. However, there was no dosage effect on frequency of antibody responses comparing Groups A and B and Groups C and D.

This study and others suggest that DNA priming may alter the impact of pre-existing Ad5 immunity on vaccine-induced immune responses and alter the quality and/or quantity of elicited T cell and antibody responses [22], [6].

A limited evaluation of ICS responses indicated that both CD4 and CD8 responses were elicited. Other studies have shown a predominant CD4 response after DNA alone, predominant CD8 response after rAd5 alone and a balanced response after DNA prime - rAd5 boost [3], [5], [6], [22].

Although it remains to be seen whether VIA activity is a true correlate of HIV protection, VIA activity has been shown to be associated with control of HIV [21], [23], [24]. Treatment-naive HIV+ subjects with a plasma viral load of <10 000/mL had median viral inhibition of 3.17 log_10_, whereas those with viral load >10000 had median viral inhibition of 1.12 log_10_ [21]. In this study, CD8 T cells from individuals vaccinated with DNA prime - rAd5 boost or rAd5 alone had a range of inhibition up to 3.7 log_10_ units. The difference in VIA activity between rAd5 alone and DNA prime - rAd5 boost was not statistically significant.

At the end of the study, most vaccine recipients tested positive on at least one commercial HIV antibody kit without being HIV-infected. Use of a commercial kit that is least likely to register vaccine-induced antibody as positive is advisable; a purpose-built kit such as HIV-SELECTEST, if eventually approved, may facilitate diagnostic HIV testing in vaccine recipients [25] within the study. In an observational follow up study of V001 volunteers, rapid HIV tests that incorporate only HIV-1 envelope proteins did not consistently detect vaccine-induced antibodies [26]. Therefore, cautious use of rapid HIV tests may be possible for long term follow up of HIV vaccine recipients, but volunteers must be warned that they may test falsely HIV positive for some time. Specialized testing services will be provided by these clinical sites until the false positive tests fade.

In Africa, the prevalence of pre-existing antibodies from natural exposure to adenovirus type 5 is at least 80% (IAVI unpublished data) compared to 30–60% in the US [27], [28]. This may be of concern: vaccine take may be compromised, leading to attenuation of immune responses to the rAd5 vector. In our study, the overall frequency of HIV-specific immune responses to the rAd5 vaccine was somewhat lower than reported in US volunteers, perhaps because of the higher Ad5 seroprevalence seen in the majority of our study participants [2], but there may be other reasons responsible for the differences observed, e.g., race, geographical area, population characteristics, nutritional status. Our findings are also consistent with the findings from the Merck Phase IIb (STEP) study, where pre-existing immunity reduced the immunogenicity of the MRK rAd5 gag-pol-nef vaccine [9]. However, it is important to note, that there are differences in properties and vaccination regimens between the VRC and the MRK vaccines and that results from the V001 and STEP study may not be comparable. With respect to the increased HIV incidence in uncircumcised male vaccinees with pre-existing immunity to Ad5 in the STEP study, a recent publication suggests that pre-existing serological immunity to Ad5 in itself does not appear to be associated with increased risk of HIV acquisition [29]. Uncircumcised status in males was a stronger predictor of HIV acquisition than pre-existing immunity to Ad5.

The V001 study was completed before the results from the STEP study (HVTN 502) became available; therefore, those results did not influence the conduct of the study [9], [10]. However, all former V001 participants were informed about the STEP study results, as were all IRBs/IECs and national regulatory agencies.

Currently, a focused Phase II study is evaluating the VRC HIV-1 DNA prime - rAd5 boost combination for its effect on early control of viral load in those study participants who become HIV-1 infected. The study is enrolling Ad5 seronegative and circumcised men who have sex with men in the USA (HVTN 505; http://clinicaltrials.gov/ct2/show/NCT00865566).

Recombinant adenovectors of serotypes other than type 5, such as rAd35 and rAd26, are also in early clinical trials as prophylactic vaccines for HIV and other diseases. The advantage of these vectors may be a lower seroprevalence [28], [30] in humans compared to Ad5. The effect of pre-existing immunity to these vectors on the immunogenicity of rAd vectored vaccines remains to be seen.

In addition, enormous efforts are being made to develop HIV vaccines capable of inducing neutralizing HIV antibodies and to design replicating viral vectors. While basic discovery and applied research are crucial for the development of a safe and efficacious HIV vaccine, it is important to continue to perform focused human clinical trials of different vaccine strategies to develop a highly effective and safe preventive HIV vaccine [31]. New functional T cell assays that allow determination of correlates of protection and/or predict vaccine efficacy are also urgently needed.

## Supporting Information

Figure S1 Local reactogenicity by Ad5 baseline titers. Ad5 neutralizing titers were stratified by values obtained prior to vaccination; <19, 19–200 and >200 as measured by the Crucell luciferase-based assay. N = numbers of individuals in each group.
(0.33 MB TIF)

Figure S2 Systemic reactogenicity by Ad5 baseline titers. Ad5 neutralizing titers were stratified by values obtained prior to vaccination; <19, 19–200 and >200 as measured by the Crucell luciferase-based assay. N = numbers of individuals in each group.
(0.22 MB TIF)

Figure S3 Percent of volunteers responding to HIV-peptide pools. The table shows the number of subjects per group at each time point that contributed ELISPOT data for the bar graph. Six pools are included in the analysis; Gag, Nef, Env A, Env B and two Pol pools. The numbers inside the bars represent percent of the responder frequencies.
(0.31 MB TIF)

Figure S4 Impact of Ad5 neutralizing antibody on IFN-γ ELISPOT responses. The bars show the cumulative proportion of vaccine recipients with positive ELISPOT responses in those with a baseline Ad5 titer >200 after the rAd5 boost in groups A–D. The p-values shown on the X-axis are based on Fisher's exact 2-tailed test.
(0.21 MB TIF)

Protocol S1 A Phase I Randomized, Placebo-Controlled, Double-Blind Trial to Evaluate the Safety and Immunogenicity of a Multiclade HIV-1 DNA Plasmid Vaccine Followed by Recombinant, Multiclade HIV-1 Adenoviral Vector Vaccine or the Multiclade HIV-1 Adenoviral Vector Vaccine Alone in Healthy Adult Volunteers not Infected with HIV
(5.64 MB PDF)

CONSORT Checklist S1 CONSORT Checklist
(0.22 MB DOC)
