# Supplementary figures and images for: Safety and Immunogenicity Study of Multiclade HIV-1 Adenoviral Vector Vaccine Alone or as Boost following a Multiclade HIV-1 DNA Vaccine in Africa
Source: PLoS One. 2010 Sep 21;5(9):e12873. doi: 10.1371/journal.pone.0012873 (PMC2943475; doi:10.1371/journal.pone.0012873)

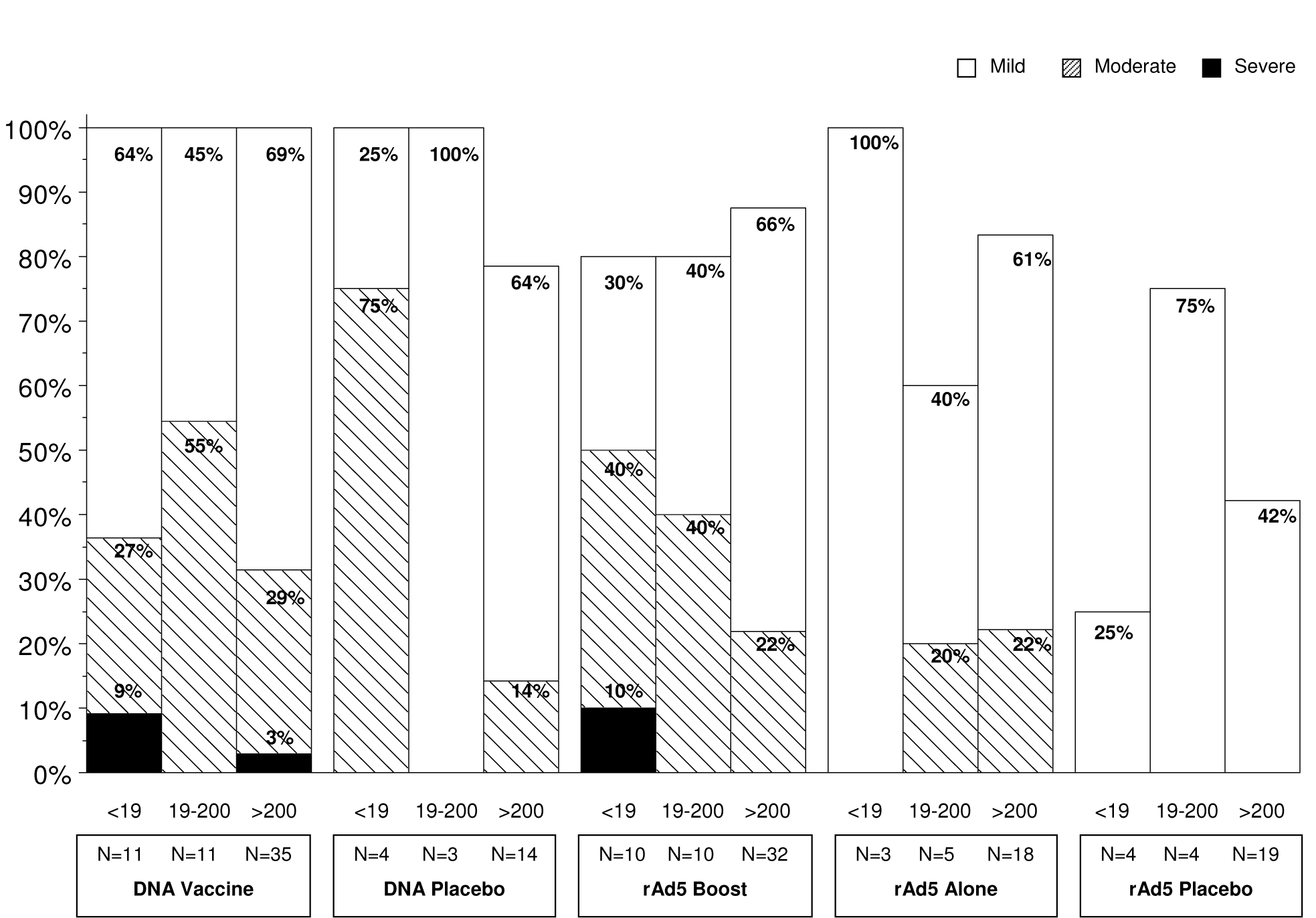

Supplement: Figure S1 — Local reactogenicity by Ad5 baseline titers. Ad5 neutralizing titers were stratified by values obtained prior to vaccination; <19, 19–200 and >200 as measured by the Crucell luciferase-based assay. N = numbers of individuals in each group. (0.33 MB TIF) [file pone.0012873.s001.tif]

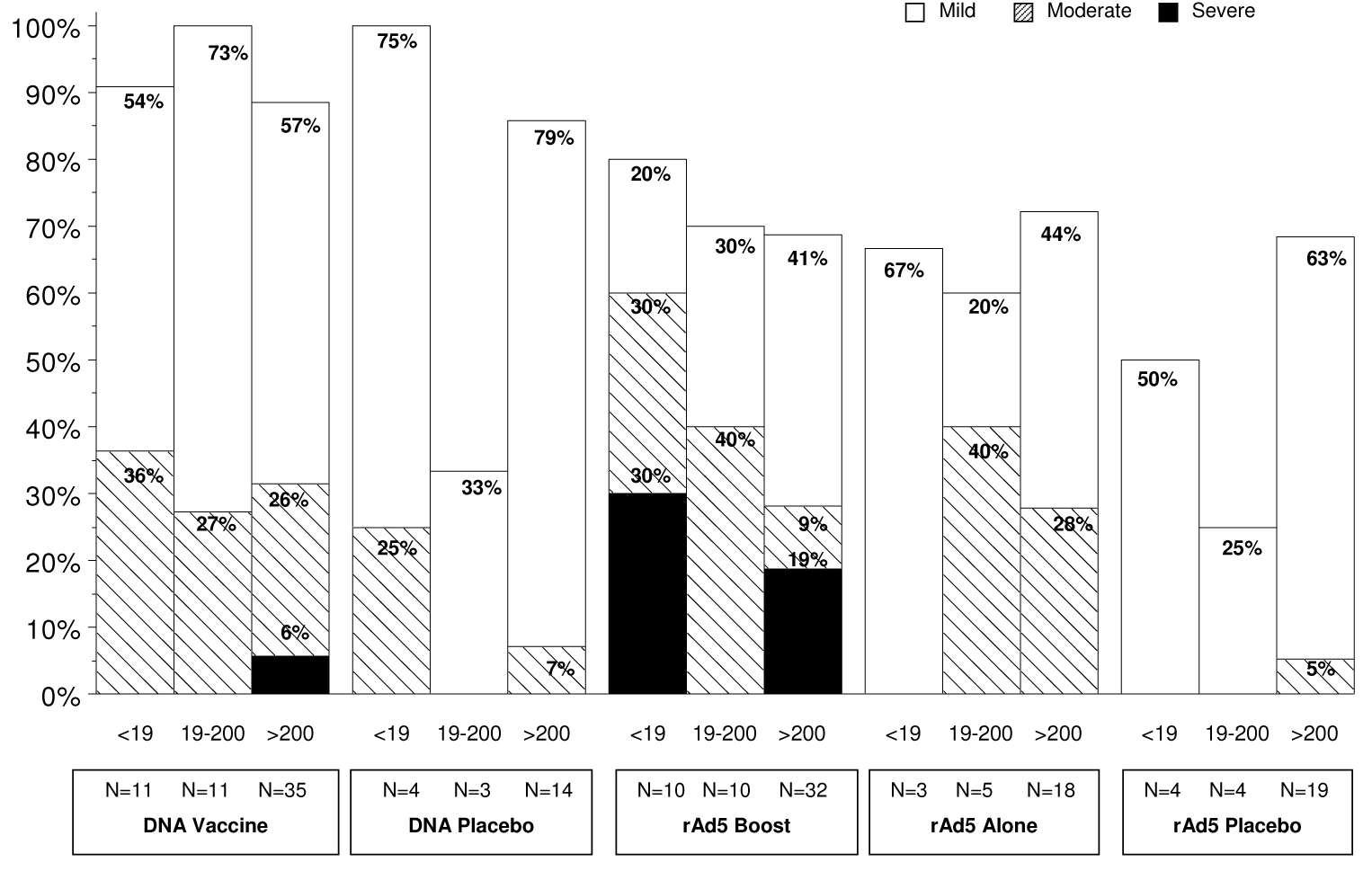

Supplement: Figure S2 — Systemic reactogenicity by Ad5 baseline titers. Ad5 neutralizing titers were stratified by values obtained prior to vaccination; <19, 19–200 and >200 as measured by the Crucell luciferase-based assay. N = numbers of individuals in each group. (0.22 MB TIF) [file pone.0012873.s002.tif]

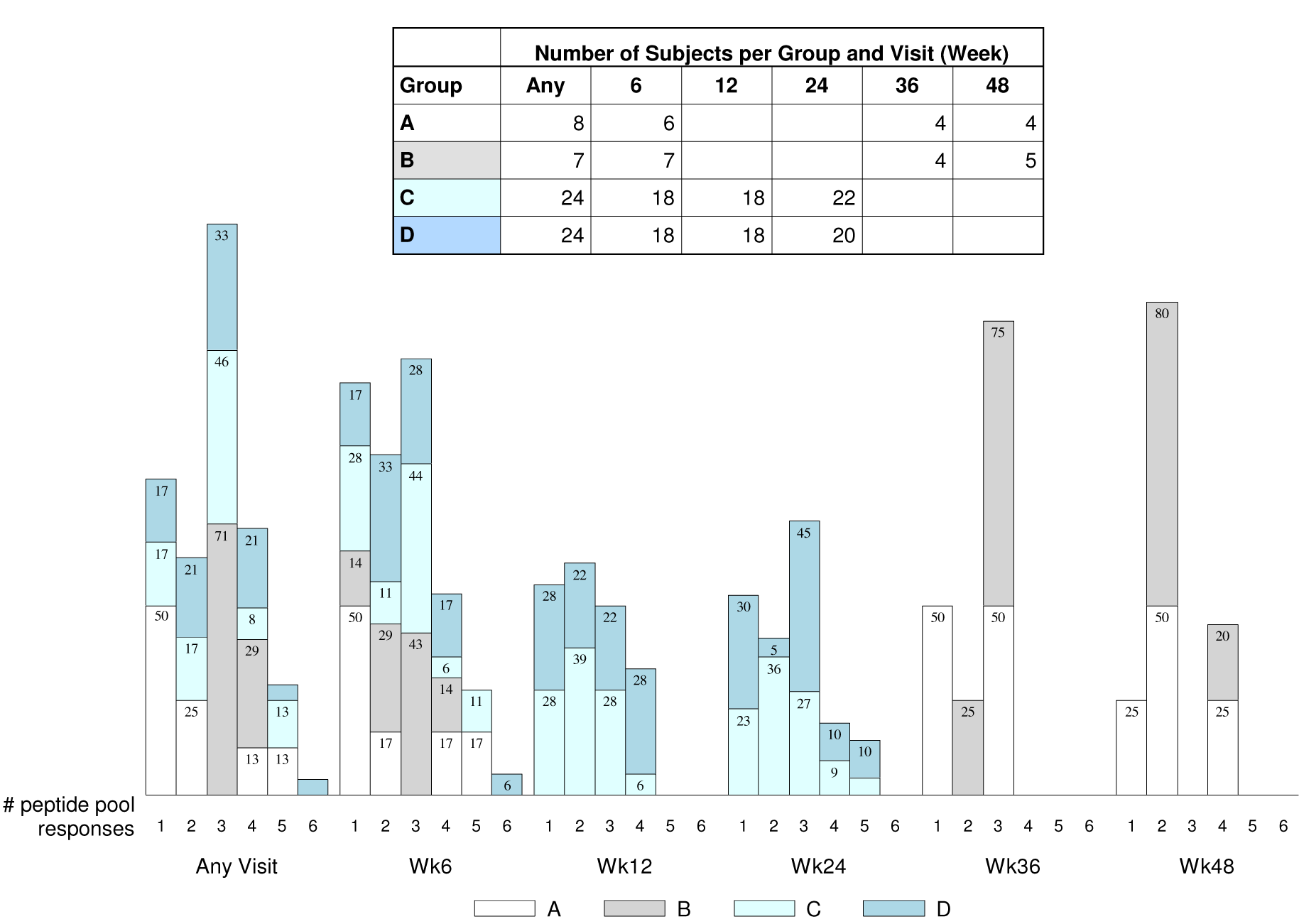

Supplement: Figure S3 — Percent of volunteers responding to HIV-peptide pools. The table shows the number of subjects per group at each time point that contributed ELISPOT data for the bar graph. Six pools are included in the analysis; Gag, Nef, Env A, Env B and two Pol pools. The numbers inside the bars represent percent of the responder frequencies. (0.31 MB TIF) [file pone.0012873.s003.tif]

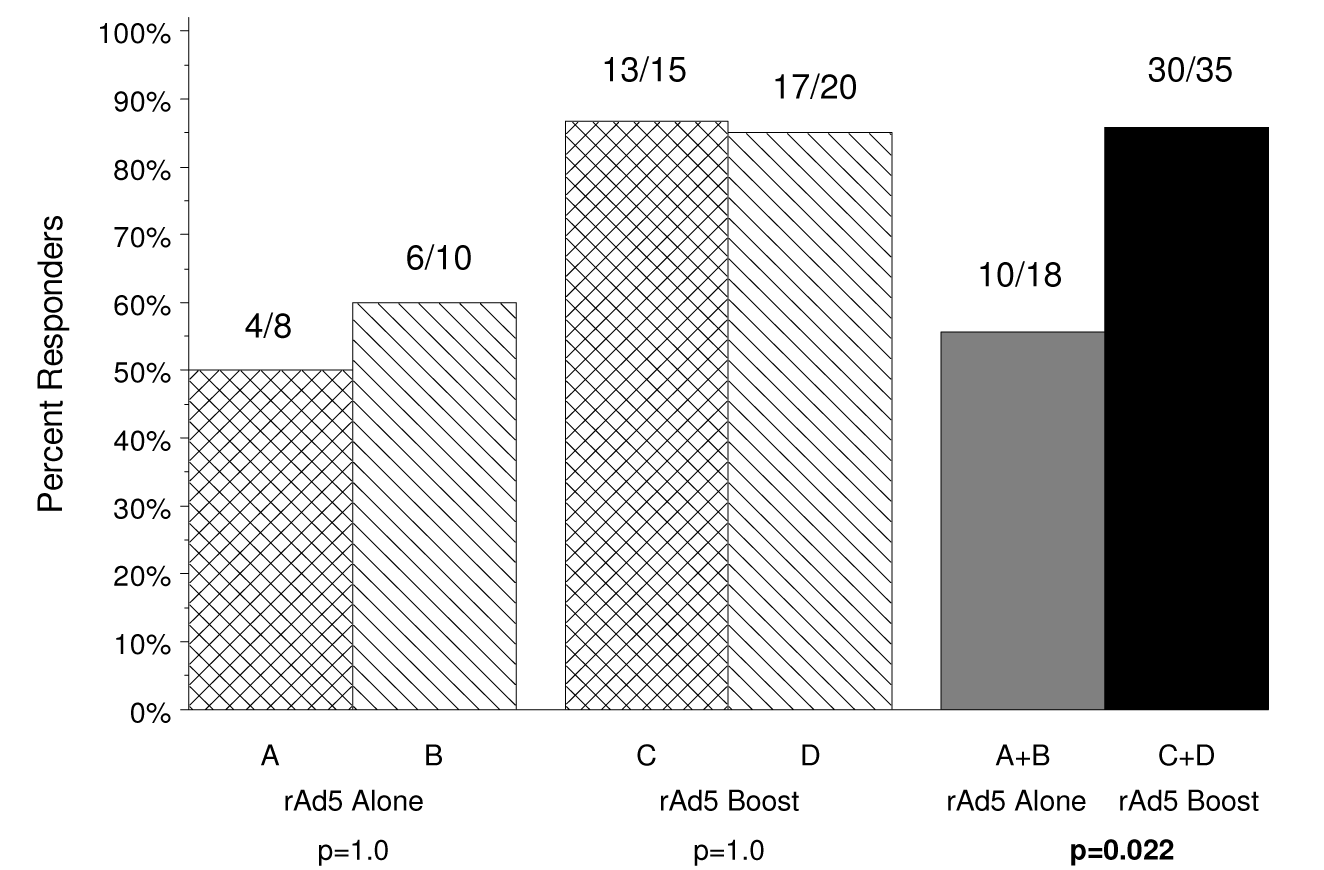

Supplement: Figure S4 — Impact of Ad5 neutralizing antibody on IFN-γ ELISPOT responses. The bars show the cumulative proportion of vaccine recipients with positive ELISPOT responses in those with a baseline Ad5 titer >200 after the rAd5 boost in groups A–D. The p-values shown on the X-axis are based on Fisher's exact 2-tailed test. (0.21 MB TIF) [file pone.0012873.s004.tif]
