# Supplementary material for: Safety and Immunogenicity Study of Multiclade HIV-1 Adenoviral Vector Vaccine Alone or as Boost following a Multiclade HIV-1 DNA Vaccine in Africa
Source: PLoS One. 2010 Sep 21;5(9):e12873. doi: 10.1371/journal.pone.0012873 (PMC2943475; doi:10.1371/journal.pone.0012873)

Letter of Amendment #3
Protocol IAVI V001
Version 1.0

Rationale for change:

IAVI and its collaborators desire to increase the sample sizes of V001 study arms C & D. The initial design called for 12 vaccinees and 4 placebo recipients in each of these arms; a number felt to be adequate for initial evaluation of a relatively new investigational agent not before evaluated in Africans. Based on the initial profile of the candidate vaccines and the abilities of sites to conduct V001, we would like to significantly increase the sizes of these two arms to better inform decision making regarding subsequent larger phase II studies of the VRC candidate in IAVI-sponsored African sites. An approximate doubling the sample sizes of arms C & D provides for enhanced precision of the observed point estimates of safety, tolerability and immunogenicity in volunteers after receiving the VRC prime boost vaccine regimen. The increases in each group contained herein are the maximum numbers that existing supplies of investigation products will accommodate.

Letter of Amendment # 3
Protocol IAVI V001
Version 1.0
Summary of Changes Overview

Amendment Change	Sections Affected by Amendment Change
Increase the total number of subjects to enroll in groups C and D	Synopsis: Study Design Table and Number of Volunteers sections<br>Section 8.2: Study Design<br>Section 8.2.2: Study Population<br>Section 11.1: Study Vaccine Regimen<br>Section 17.1: Sample Size<br>Section 17.2: Statistical Power and Analysis<br>Appendix D (Sample Informed Consent): Study Procedures<br>Appendix D (Sample Informed Consent): Injection Visits

Letter of Amendment #3
Protocol IAVI V001
Version 1.0

Summary of Changes

1. Synopsis

Study Design Table
Used to Read

Randomized, placebo-controlled, double-blind with respect to vaccine or placebo assignment

Eligible volunteers will be randomized as follows:

Total ~~64~~

Groups	Vaccine	N*	M 0	M 1	M 2	M 6
A	rAd5 10^{10}	12/4	rAd5/P			
B	rAd5 10^{11}	12/4	rAd5/P			
C	DNA+rAd5$10^{10}$	~~12/4~~	DNA/P	DNA/P	DNA/P	rAd5/P
D	DNA+rAd5$10^{11}$	~~12/4~~	DNA/P	DNA/P	DNA/P	rAd5/P

N* =Vaccine/placebo, M=Month, P= placebo

Now Reads
Randomized, placebo-controlled, double-blind with respect to vaccine or placebo assignment

Eligible volunteers will be randomized as follows:

Total **104**

Groups	Vaccine	N*	M 0	M 1	M 2	M 6
A	rAd5 10^{10}	12/4	rAd5/P			
B	rAd5 10^{11}	12/4	rAd5/P			
C	DNA+rAd5$10^{10}$	**27/9**	DNA/P	DNA/P	DNA/P	rAd5/P
D	DNA+rAd5$10^{11}$	**27/9**	DNA/P	DNA/P	DNA/P	rAd5/P

N* =Vaccine/placebo, M=Month, P= placebo

Letter of Amendment #3
Protocol IAVI V001
Version 1.0

Number of Volunteers
Used to read

~~64~~ volunteers (~~48~~ Vaccine/~~16~~ Placebo recipients) will be included in the study. An over-enrollment of up to 10% (approximately ~~6~~ additional volunteers) will be permitted in the study. 16 volunteers will be enrolled in ~~each group~~ and randomized in a 3:1 ratio of active vaccine to placebo.

Now reads

104 volunteers (**78** Vaccine/**26** Placebo recipients) will be included in the study. An over-erollment of up to 10% (approximately **10** additional volunteers) will be permitted in the study. 16 volunteers will be enrolled in **each of groups A and B whereas 36 volunteers will be enrolled in each of groups C and D** and randomized in a 3:1 ratio of active vaccine to placebo.

2. Section 8.2: Study Design

Table

Used to read
This study is a randomized, placebo-controlled, double-blind with respect to vaccine or placebo assignment. Eligible volunteers will be ~~randomized~~ as follows:

Total ~~64~~

Groups	Vaccine	N*	M 0	M 1	M 2	M 6
A	rAd5 10^{10}	12/4	rAd5/P			
B	rAd5 10^{11}	12/4	rAd5/P			
C	DNA+rAd5$10^{10}$	~~12/4~~	DNA/P	DNA/P	DNA/P	rAd5/P
D	DNA+rAd5$10^{11}$	~~12/4~~	DNA/P	DNA/P	DNA/P	rAd5/P

N* =Vaccine/placebo, M=Month, P= placebo

Letter of Amendment #3
Protocol IAVI V001
Version 1.0

Now reads

This study is a randomized, placebo-controlled, double-blind with respect to vaccine or placebo assignment. Eligible volunteers will be **assigned** as follows:

Total **104**

Groups	Vaccine	N*	M 0	M 1	M 2	M 6
A	rAd5 10^{10}	12/4	rAd5/P			
B	rAd5 10^{11}	12/4	rAd5/P			
C	DNA+rAd5$10^{10}$	**27/9**	DNA/P	DNA/P	DNA/P	rAd5/P
D	DNA+rAd5$10^{11}$	**27/9**	DNA/P	DNA/P	DNA/P	rAd5/P

N* =Vaccine/placebo, M=Month, P= placebo

3. Section 8.2.2: Study Population (last paragraph)

Used to read

Approximately ~~64~~ volunteers (~~48~~ Vaccine recipients, ~~16~~ placebo recipients), at least 30% females, who meet all eligibility criteria will be included in the study. An over-enrollment of up to 10% (approximately ~~6~~ additional volunteers) will be accepted in the study.

Now reads

Approximately **104** volunteers (**78** Vaccine recipients, **26** placebo recipients), at least 30% females, who meet all eligibility criteria will be included in the study. An over-enrollment of up to 10% (approximately **10** additional volunteers) will be accepted in the study.

Letter of Amendment #3
Protocol IAVI V001
Version 1.0

4. Section 11.1: Study Vaccine Regimen (Table)

Used to read

Group	N	Treatment	Priming			Boosting
			Injection Schedule in Months (*Days*)			
			0*(0)*	1*(28)*	2*(56)*	6*(168)*
A	12	*T-A*	rAd5 $1x10^{10}$ PU IM*			
	4	*C-A*	rAD5 Placebo 1ml IM*			
B	12	*T-B*	rAd5 $1x10^{11}$ PU IM*			
	4	*C-B*	rAd5 Placebo 1ml IM*			
C	~~12~~	*T-C*	DNA 4mg IM**	DNA 4mg IM**	DNA4mg IM**	rAD$1x10^{10}$ PU IM*
	4	*C-C*	DNA Placebo 1ml IM**	DNA Placebo 1ml IM**	DNA Placebo 1ml IM**	rAD Control 1ml IM**
D	~~12~~	*T-D*	DNA 4mg IM**	DNA 4mg IM**	DNA 4mg IM**	rAD $1x10^{11}$ PU IM*
	4	*C-D*	DNA Placebo 1ml IM**	DNA Placebo 1ml IM**	DNA Placebo 1ml IM**	rAD Control 1ml IM**

*Administered with needle and syringe as one 1 mL intramuscular (IM) injection in either deltoid.
**Administered by Biojector® as one 1 mL IM injection in either deltoid. Day 0 is defined as the day of first injection

Now reads

Group	N	Treatment	Priming			Boosting
			Injection Schedule in Months (*Days*)			
			0*(0)*	1*(28)*	2*(56)*	6*(168)*
A	12	*T-A*	rAd5 $1x10^{10}$ PU IM*			
	4	*C-A*	rAD5 Placebo 1ml IM*			
B	12	*T-B*	rAd5 $1x10^{11}$ PU IM*			
	4	*C-B*	rAd5 Placebo 1ml IM*			
C	**27**	*T-C*	DNA 4mg IM**	DNA 4mg IM**	DNA4mg IM**	rAD$1x10^{10}$ PU IM*
	**9**	*C-C*	DNA Placebo 1ml IM**	DNA Placebo 1ml IM**	DNA Placebo 1ml IM**	rAD Control 1ml IM**
D	**27**	*T-D*	DNA 4mg IM**	DNA 4mg IM**	DNA 4mg IM**	rAD $1x10^{11}$ PU IM*
	**9**	*C-D*	DNA Placebo 1ml IM**	DNA Placebo 1ml IM**	DNA Placebo 1ml IM**	rAD Control 1ml IM**

*Administered with needle and syringe as one 1 mL intramuscular (IM) injection in either deltoid.
**Administered by Biojector® as one 1 mL IM injection in either deltoid. Day 0 is defined as the day of first injection

Letter of Amendment #3
Protocol IAVI V001
Version 1.0

5. Section 17.1: Sample Size

Used to read
A total of ~~64~~ volunteers (~~48~~ Vaccine/~~16~~ placebo) will be entered into 4 groups scheduled to receive vaccine or placebo. Within each group, volunteers will be allocated in a 3:1 vaccine to placebo ratio and all will be followed for reactogenicity and systemic toxicity.

Now reads
A total of **104** volunteers (**78** Vaccine/**26** placebo) will be entered into 4 groups scheduled to receive vaccine or placebo. Within each group, volunteers will be allocated in a 3:1 vaccine to placebo ratio and all will be followed for reactogenicity and systemic toxicity.

6. Section 17.2: Statistical Power and Analysis

Used to read
For life-threatening adverse events related to investigational product: if none of the volunteers receiving the investigational product experiences such events (n=~~48~~) the 95% upper confidence bound for the rate of these adverse events in the population is 0.~~06~~.

Now reads
For life-threatening adverse events related to investigational product: if none of the volunteers receiving the investigational product experiences such events (n=**78**) the 95% upper confidence bound for the rate of these adverse events in this population is 0.**04.**

7. Appendix D (Sample Informed Consent): Study Procedures

Used to read
~~About 64~~ volunteers are expected to participate in this study at two sites: Projet San Francisco in Kigali, Rwanda and KAVI in Nairobi, Kenya.

Now reads
Approximately one hundred and four (104) volunteers are expected to participate in this study at two sites: Projet San Francisco in Kigali, Rwanda and KAVI in Nairobi, Kenya.

8. Appendix D (Sample Informed Consent): Injection Visits

Used to read
Injection Visits
You will be assigned by chance (like the flip of a coin) to one of four groups below:
• Group A: 12 volunteers will receive one injection of low dose rAd5 vaccine and 4 volunteers will receive one injection of placebo.

Letter of Amendment #3
Protocol IAVI V001
Version 1.0

• Group B: 12 volunteers will receive one injection of high dose rAd5 vaccine and 4 volunteers will receive one injection of placebo
• Group C: ~~12~~ volunteers will receive 3 injections of DNA vaccine followed by 1 injection of low dose rAd5 vaccine and 4 volunteers will receive 4 injections of placebo.
• Group D: ~~12~~ volunteers will receive 3 injections of DNA vaccine followed by 1 injection of high dose rAd5 vaccine and ~~4~~ volunteers will receive 4 injections of placebo

Now reads
Injection Visits
You will be assigned by chance (like the flip of a coin) to one of four groups below:
• Group A: 12 volunteers will receive one injection of low dose rAd5 vaccine and 4 volunteers will receive one injection of placebo.
• Group B: 12 volunteers will receive one injection of high dose rAd5 vaccine and 4 volunteers will receive one injection of placebo
• Group C: **27** volunteers will receive 3 injections of DNA vaccine followed by 1 injection of low dose rAd5 vaccine and **9** volunteers will receive 4 injections of placebo.
• Group D: **27** volunteers will receive 3 injections of DNA vaccine followed by 1 injection of high dose rAd5 vaccine and **9** volunteers will receive 4 injections of placebo

Protocol	V001 version 1.0 June 06, 2005
**Trial Master File**	Section : Protocol
**Subject**	Clarification post-vaccination reactogenicity assessment
**Site**	Kigali and KAVI
**Date**	26Oct2005

File note
Protocol V001 version 1.0 (June 6, 2005) specifies in section 9.3. Post-vaccination visits the following " on Days 3 and 14 after each vaccination the study personnel will review the memory card with the volunteer and record the information in the clinic chart,
The following procedures will be conducted at these visits:
.....

- assess local and systemic reactogenicity as well as any other adverse events"

Section 12.1.1 Local reactogenicity and 12.1.2. Systemic reactogenicity refers to the Schedule of Procedures (Appendix A and B) for the timepoints for the assessment of reactogenicity events

The Schedule of Procedures (Appendix A and B) specify that local and systemic reactogenicity assessment (pre-and post vaccination) will occur

- Appendix A (group C and D): day 0, day 3 and day 14, day 28, day 31, day 42, day 56, day 59 , day 70, day 84, day 168, day 171 and day 182
- Appendix B (group A and B): day 0, day 3 and day 14

After discussions between the three different networks (HVTN, USMHRP and IAVI) to ensure consistency in data collection, it was decided that the post-vaccination reactogenicity assessment would be done at day 3 post-vaccination only (and not on day 14).

Action:

In the next protocol amendment, the timepoints for post-vaccination reactogenicity will be corrected. In the meantime we will only perform post-vaccination reactogenicity assessment on day 3 post-vaccination.
The Schedule of Procedures (Appendix A and B) will be amended so that local and systemic reactogenicity assessment (pre-and post vaccination) will occur

- *Appendix A (group C and D): day 0, day 3, day 28, day 31, day 56, day 59 , day 168, day 171*
- *Appendix B (group A and B): day 0, day 3*

The administrative error in appendix A where study Month 6.5 (Day 182) has been wrongly identified as week 28 will be corrected to week 26.

This file note will be filed at the site trial master file and IAVI trial master file to record the above corrections.

Sponsor:

Signed: ______________________ Date: 26. Oct.'05

Dr. Soe Than
Senior Director, Medical Affairs, IAVI

Letter of Amendment 1
Protocol V001
Version 1.0
Summary of Changes Overview

Amendment Change	Sections Affected by Amendment Change
Add Professor Susan Allen as another PI	Page 1: Front Page
Add Confirmed Diagnosis of Hepatitis C in exclusion criteria	Section 8.2.4 Exclusion Criteria
HLA typing results may be available for volunteers upon their request	Section 12.3.1 Genetic testing
Correct ion of the figure in Appendix E	Appendix E: HIV testing algorithm

Summary of Changes

1. Front Page

Used to Read:
Principle Investigators
Professor Job Bwayo
KAVI

Dr. Etienne Karita
Projet San Francisco

Now Reads:
Principle Investigators
Professor Job Bwayo
KAVI

<u>Professor Susan Allen</u>
<u>Emory University</u>

Dr. Etienne Karita
Projet San Francisco

Rationale for change:
Addition of Dr. Susan Allen as another PI at Project San Francisco

2. Section 8.2.4: Exclusion Criteria #5

Used to Read:
Confirmed diagnosis of hepatitis B (surface antigen, HbsAg); or active or untreated syphilis (documented by exam or serology unless positive serology is due to remote

treated infection, or positive rapid plasma regain/venereal disease research laboratory (RPR/VDRL) test is not associated with positive Treponemal specific serology).

Now Reads:

Confirmed diagnosis of hepatitis B (surface antigen, HbsAg); Hepatitis C (HCV antibodies) or active or untreated syphilis (documented by exam or serology unless positive serology is due to remote treated infection, or positive rapid plasma regain/venereal disease research laboratory (RPR/VDRL) test is not associated with positive Treponemal specific serology).

Rationale for change: per suggestion from ethics committee

3. Section 12.3.1 Genetic Testing

Used to read:

Samples for HLA typing will be collected at the time point indicated in the Schedule of Procedures (Appendices A and B). HLA typing will be performed on samples for all participants vaccinated at a dosage level, provided T-cell responses are detected at that dosage level. The results will be kept confidential and are only for the purpose of characterizing the T-cell immune response. Volunteers ~~will not~~ receive the results of HLA typing.

Now reads:

Samples for HLA typing will be collected at the time point indicated in the Schedule of Procedures (Appendices A and B). HLA typing will be performed on samples for all participants vaccinated at a dosage level, provided T-cell responses are detected at that dosage level. The results will be kept confidential and are only for the purpose of characterizing the T-cell immune response. Volunteers will receive the results of HLA typing upon their request. In case the volunteer requests the HLA results, the data will be explained to the volunteer by the study physician.

Rationale for change: per suggestion from ethics committee

4. Appendix E: HIV testing algorithm

Previous Figure in Appendix E

Please see attached

Corrected figure for AppendixE

Please attached

Rationale for change:

To correctly depict the procedure outlined in Section 14.1, HIV Testing.

IAVI V001

**Protocol Title:**	A Phase I, Randomized, Placebo-Controlled, Double-Blind Trial to Evaluate the Safety and Immunogenicity of a Multiclade HIV-1 DNA Plasmid Vaccine Followed by Recombinant, Multiclade HIV-1 Adenoviral Vector Vaccine or the Multiclade HIV-1 Adenoviral Vector Vaccine Alone in Healthy Adult Volunteers not Infected with HIV
**Study Sites:**	KAVI- University of Nairobi<br>Nairobi, Kenya<br><br>Projet San Francisco<br>Kigali, Rwanda
**Principal Investigators:**	Professor Job Bwayo<br>KAVI<br><br>Dr. Etienne Karita<br>Projet San Francisco
**Protocol Number:**	IAVI V001
**Phase:**	Phase I
**Regulatory Investigational Product Number: (IND number)**	**BB-IND 12326**
**IND Holder**	Division of AIDS (DAIDS), NIAID, NIH, Departments of Health and Human Services (DHHS), Bethesda, Maryland, USA
**Sponsor:**	International AIDS Vaccine Initiative (IAVI)<br>110 William Street, 27th Floor<br>New York, NY 10038-3901
**Vaccine Provider**	Vaccine Research Center<br>National Institutes of Allergy and Infectious Diseases (NIAID)<br>40 Convent Drive<br>National Institutes of Health<br>Bethesda, MD 20892
**Date of Protocol**	Version 1.0 June 06, 2005

The **CONFIDENTIAL INFORMATION** in this document is provided to you as an Investigator, potential Investigator, or Consultant, for review by you, your staff, and applicable institutional review boards (IRBs) and/or independent ethics committees (IECs). It is understood that the information will not be disclosed to others, except to the extent necessary to obtain ethical and regulatory approval from the respective committee's agencies and informed consent from those persons to whom the Investigational Product may be administered.

SYNOPSIS

**TITLE:**	A Phase I, Randomized, Placebo-Controlled, Double-Blind Trial to Evaluate the Safety and Immunogenicity of a Multiclade HIV-1 DNA Plasmid Vaccine Followed by Recombinant, Multiclade HIV-1 Adenoviral Vector Vaccine or the Multiclade HIV-1 Adenoviral Vector Vaccine Alone in Healthy Adult Volunteers not Infected with HIV
**PROTOCOL NUMBER:**	IAVI V001
**Regulatory Investigational Product Number: (IND Number)**	**BBIND 12326**
**PHASE:**	Phase I
**SPONSOR:**	International AIDS Vaccine Initiative (IAVI)<br>110 William Street, 27th Floor<br>New York, New York 10038-3901
**OBJECTIVES:**	***Primary:***<br>• Evaluate the safety and tolerability of VRC HIV-1 recombinant adenovirus-5 vector (rAd5) vaccine at either 10^{10} particle units (PU) or 10^{11} PU in HIV-1 uninfected adults.<br>• Evaluate the safety and tolerability of three VRC HIV-1 DNA-six-plasmid vaccine doses at 4.0 mg/dose in HIV-1 uninfected adults.<br>• Evaluate the safety and tolerability of three VRC HIV-1 DNA-six-plasmid vaccine doses at 4.0 mg/dose boosted with VRC HIV-1 rAd5 vaccine at either 10^{10} PU or 10^{11} PU in HIV-1 uninfected adults.<br>***Secondary:***<br>• Evaluate the immunogenicity of rAd5 alone or DNA + rAd5 vs. placebo<br>• Evaluate the frequency and durability of cellular immune response.<br>• Characterize the magnitude and breadth of the vaccine-induced HIV specific T cell response as measured by ELISPOT and ICS assays.<br>• Evaluate the impact of pre-existing immunity to rAd5 on immunogenicity
**PRIMARY ENDPOINTS**	Safety and tolerability will be evaluated by monitoring participants for local and systemic adverse reactions after each injection and for 12 months after the first injection.<br>The following parameters will be assessed:<br>• Local reactogenicity signs and symptoms<br>• Systemic reactogenicity signs and symptoms<br>• Laboratory measures of safety<br>• Adverse and serious adverse experiences

SECONDARY ENDPOINTS

- The proportion of volunteers who have HIV-1 specific T- cell responses quantified by Intracellular cytokine staining (ICS; both CD4+ and CD8+) and ELISPOT and magnitude of the responses.
- The proportion of volunteers with HIV-1 specific antibodies and magnitude of the response
- Proportion of volunteers with an increase in antibodies to rAd5
- Impact of pre-existing immunity to rAd5 on immunogenicity

OTHER ENDPOINTS

Proportion of volunteers who test “false positive” on standard HIV testing algorithm

STUDY DESIGN TABLE

Randomized, placebo-controlled, double-blind with respect to vaccine or placebo assignment. Eligible volunteers will be randomized as follows:

Total 64

Groups	Vaccine	N*	M 0	M 1	M 2	M 6
A	rAd5 10^{10}	12/4	rAd5/P			
B	rAd5 10^{11}	12/4	rAd5/P			
C	DNA+rAd5$10^{10}$	12/4	DNA/P	DNA/P	DNA/P	rAd5/P
D	DNA+rAd5$10^{11}$	12/4	DNA/P	DNA/P	DNA/P	rAd5/P

N* =Vaccine/placebo, M=Month, P= placebo

METHODS:

See Schedule of Procedures; Appendices A and B

STUDY POPULATION:

Healthy male or female adults 18–50 years of age, who are willing to undergo HIV testing and who, in the opinion of the principal investigator or designee, understand the study and provide written informed consent. Female volunteers must be willing to use an effective method of contraception for the duration of their participation in the study.

Principal exclusion criteria include confirmed HIV-1 or HIV-2 infection, reported high-risk behavior for HIV infection, pregnancy and lactation, chronic disease, clinically significant abnormal laboratory values, recent vaccination(s) or receipt of a blood product, vaccine, or other investigational products, and previous severe local or systemic reaction(s) to any vaccination or history of severe allergic reactions.

<table>
<tr><td>NUMBER OF VOLUNTEERS:</td><td>64 volunteers (48 Vaccine/16 Placebo recipients) will be included in the study. An over-enrollment of up to 10% (approximately 6 additional volunteers) will be permitted in the study. 16 volunteers will be enrolled in each group and randomized in a 3:1 ratio of active vaccine to placebo.</td></tr>
<tr><td>DESCRIPTION OF INVESTIGATIONAL PRODUCT:</td><td><u>Vaccines</u>:

<u>DNA</u>: Recombinant, multiclade HIV-1 DNA plasmid vaccine (VRC-HIVDNA016-00-VP), composed of 6 closed circular plasmids which encode for HIV gag, HIV pol and HIV nef on separate plasmids along with plasmids encoding genes for clades A, B and C Env glycoproteins; 50% by mass are gag, pol, nef.

<u>rAd5:</u> Recombinant multiclade HIV-1 adenoviral vector (VRC-HIVADV014-00-VP), composed of 4 adenoviral vectors in a 3:1:1:1 ratio that encode the HIV-1 Gag/Pol polyprotein from clade B and HIV-1 Env glycoproteins from clades A, B, C respectively.

<u>Placebo for (VRC- HIVDNA016-00-VP) DNA Vaccine:</u> Phosphate-buffered saline (PBS): a clear, colorless, isotonic solution with a pH of 7.2, distributed in vials containing 2.4 mL.

<u>Placebo/Diluent for (VRC-HIVADV014-00-VP) rAd5 Vaccine:</u> VRC-DILUENT013-DIL-VP: Final Formulation Buffer, (FFB) is composed of sodium chloride, tris buffer, trehalose.$2H_2O$ (low endotoxin), magnesium chloride.$6H_2O$, monooleate (tween 80), and water for injection. It is a clear colorless solution with pH 7.2±0.2.

<table>
<tr><th>Investigational Product</th><th>Dose</th><th>Total Injected Volume (mL)</th><th>Route of Administration</th></tr>
<tr><td>rAd5</td><td>10^{10}</td><td>1.0</td><td>IM</td></tr>
<tr><td>rAd5</td><td>10^{11}</td><td>1.0</td><td>IM</td></tr>
<tr><td>DNA</td><td>4 mg</td><td>1.0</td><td>IM</td></tr>
<tr><td>Placebo (PBS)*</td><td>n/a</td><td>1.0</td><td>IM</td></tr>
<tr><td>Placebo (FFB)**</td><td>n/a</td><td>1.0</td><td>IM</td></tr>
</table>
* Placebo for DNA Vaccine, ** Placebo for rAd5 vaccine, IM = Intramuscular</td></tr>
<tr><td>BLINDING:</td><td>Study volunteers, site personnel and laboratory personnel, except the pharmacist, will be blinded with respect to the candidate vaccines and placebo within each arm, but not between groups A&B (groups receiving one injection of rAd5 or placebo only) vs. groups C&D (groups receiving 3 injections of DNA vaccine/placebo followed by rAd5/placebo).</td></tr>
</table>

**DURATION OF STUDY PARTICIPATION:**	Volunteers will be screened up to 6 weeks before the first study injection and will be followed for 12 months after the first study injection. It is anticipated that it will take approximately 6 months to enroll the study. The total duration of the study will be approximately 18 months.
**EVALUATION FOR INTERCURRENT HIV INFECTION:**	Volunteers will be clinically evaluated and tested for HIV-1 and HIV-2 antibodies at Screening, Day 0, before the initial study injection, at Months 2, 6 and 9, and at the end of the study using the protocol-defined algorithm. HIV testing at additional time points may be performed at the discretion of the volunteer and principal investigator or designee as medical or social circumstances arise. Test results will be interpreted according to a pre-determined HIV diagnostic algorithm to distinguish a true HIV infection from a vaccine-induced antibody response. The tests will be conducted at a laboratory not under the direction of the investigator in order to prevent unblinding.
**STATISTICAL CONSIDERATIONS:**	V001 is a randomized, phase I trial with at least 30% female volunteers. Pre-screening for Ad NAb will not occur; however Ad5 NAbs will be evaluated.<br><br>Study data will be identified only by volunteer ID number and will be collected in the centralized database. Interim analyses of grouped data will be carried out without unblinding the study to investigators or volunteers at time(s) to be determined by the Protocol Team. At the end of the study, a full analysis will be prepared according to a pre-specified statistical analysis plan.<br><br>Safety and tolerability will be tabulated by the proportion of volunteers who experience these events and the relationship to study vaccines. All clinical and routine laboratory data will be included in the safety analysis. Immunogenicity analyses will be performed in all volunteers who have received study injections. Volunteers will be classified as responders or non-responders and the magnitude of the immune responses will be compared for each vaccine group against placebo.

TABLE OF CONTENTS

SYNOPSIS **2**
ABBREVIATIONS **10**

SYNOPSIS 2
ABBREVIATIONS 10

1.0. CONTACT INFORMATION **12**
1.1 PRINCIPAL INVESTIGATOR CONTACT INFORMATION 12
1.2 SPONSOR CONTACT INFORMATION 12
1.3 DIADS CONTACT INFORMATION 13
2.0 SIGNATURE PAGE **14**
3.0 INTRODUCTION AND BACKGROUND INFORMATION **15**
3.0.1 KENYA 15
3.0.2 RWANDA 16
3.1 RATIONALE FOR MULTICLADE VACCINES FOR HIV-1 17
3.2 RATIONALE FOR DNA AND ADENOVIRUS-VECTORED VACCINES 18
3.2.1 DNA VACCINES 18
3.2.2 ADENOVIRUS-VECTORED VACCINES 19
3.2.3 EXPECTED DISTRIBUTION OF AD5 NEUTRALIZING ANTIBODY TITERS 21
3.2.4 RATIONALE FOR PRIME-BOOST VACCINE STRATEGIES 22
3.2.5 RATIONALE FOR CONDUCTING 3 SEPARATE PROTOCOLS 23
4.0 STUDY PRODUCT DESCRIPTIONS **24**
4.1 SOURCE AND CHARACTERISTICS OF THE HIV GENE INSERTS 24
4.2 DNA VACCINE PLASMIDS: VRC-HIVDNA016-00-VP 25
4.2.1 BIOJECTOR® 2000 NEEDLE-FREE INJECTION SYSTEM 25
4.3 RECOMBINANT ADENOVIRAL VECTORS: VRC-HIVADV014-00-VP 25
5.0 PRECLINICAL STUDIES **26**
5.1 SUMMARY OF PRECLINICAL SAFETY STUDIES: VRC DNA PLASMIDS 26
5.1.1 BIODISTRIBUTION OF VRC-HIVDNA006-00-VP 27
5.1.2 REPEAT-TOXICITY OF VRC-HIVDNA006-00-VP 27
5.2 SUMMARY OF PRECLINICAL SAFETY STUDIES: VRC RAD AND DNA PRIME FOLLOWED BY RAD BOOST 27
5.2.1 REPEAT-TOXICITY OF DNA PRIME/RAD5 BOOST HIV VACCINE REGIMEN 28
5.3 SUMMARY OF PRECLINICAL IMMUNOGENICITY STUDIES 29
6.0 CLINICAL STUDIES **31**
6.1 DNA PLASMID VACCINE TRIALS 31
6.1.1 PROTOCOL VRC 001 31
6.1.2 PROTOCOL VRC 004 32
6.1.3 PROTOCOL HVTN 044 33
6.1.4 PROTOCOL HVTN 052 33
6.1.5 PROTOCOL RV 156 34
6.1.6 PROTOCOL VRC 007 34
6.2 RECOMBINANT ADENOVIRAL VECTOR VACCINE TRIALS 35
6.2.1 PROTOCOL VRC 006 36
6.2.2 PROTOCOL HVTN 054 37
6.3 PRIME-BOOST TRIALS 37
6.3.1 PROTOCOL HVTN 057 37
6.3.2 PROTOCOL VRC 009 38
6.3.3 PROTOCOL VRC 008 38
6.3.4 PROTOCOL VRC 010 38

7.0 STUDY OBJECTIVES 38
7.1 PRIMARY OBJECTIVES 38
7.2 SECONDARY OBJECTIVES 39
8.0 STUDY ENDPOINTS AND STUDY DESIGN 39
8.1 STUDY ENDPOINTS 39
8.1.1 PRIMARY ENDPOINTS 39
8.1.2 SECONDARY ENDPOINTS 39
8.1.3 OTHER ENDPOINTS 39
8.2 STUDY DESIGN 40
8.2.1 DURATION OF THE STUDY 40
8.2.2 STUDY POPULATION 40
8.2.3 INCLUSION CRITERIA 41
8.2.4 EXCLUSION CRITERIA 42
8.2.5 RECRUITMENT OF VOLUNTEERS 43
9.0 STUDY VISITS 43
9.1 SCREENING VISIT 43
9.2 VACCINATION VISIT 44
9.3 POST-VACCINATION VISITS 44
9.4 ADDITIONAL FOLLOW-UP VISITS 45
9.5 UNSCHEDULED VISITS/CONTACT 45
9.6 FINAL VISIT/EARLY TERMINATION VISIT 45
10.0 STUDY PROCEDURES 46
10.1 PROTOCOL REGISTRATION 46
10.2 INFORMED CONSENT PROCESS 46
10.3 MEDICAL HISTORY AND PHYSICAL EXAMINATION 47
10.4 HIV RISK ASSESSMENT, HIV TESTING AND HIV-TEST COUNSELING 47
10.5 FAMILY PLANNING COUNSELING 47
10.6 BLOOD COLLECTION 47
10.7 REIMBURSEMENT 48
10.8 RANDOMIZATION AND BLINDING 48
10.9 UNBLINDING PROCEDURE FOR INDIVIDUAL VOLUNTEERS 48
11.0 INVESTIGATIONAL PRODUCT 49
11.1 STUDY VACCINE REGIMEN 49
11.2 STUDY PRODUCT FORMULATION AND PREPARATION 50
11.2.1 VRC-HIV ADV014-00-VP 1 X 10^{10} PU (MULTICLADE HIV-1 RECOMBINANT ADENOVIRAL VECTOR VACCINE, RAD5) 50
11.2.2 VRC-HIV ADV014-00-VP 1 X 10^{11} PU (MULTICLADE HIV-1 RECOMBINANT ADENOVIRAL VECTOR VACCINE, RAD5) 51
11.2.3 VRC-DILUENT013-DIL-VP (FINAL FORMULATION BUFFER, FFB, PLACEBO FOR RAD5) 51
11.2.4 VRC-HIVDNA016-00-VP (MULTICLADE HIV-1 DNA SIX PLASMID VACCINE) 51
11.2.5 PLACEBO FOR DNA VACCINE (PHOSPHATE BUFFERED SALINE, PBS) 52
11.2.6 LABELING PROCEDURES TO PRESERVE BLINDING 52
11.3 PROCEDURES TO PRESERVE BLINDING 52
11.4 STUDY PRODUCT ADMINISTRATION 53
11.4.1 VRC-HIVADV014-00-VP (RAD5 VECTOR) OR PLACEBO(LABELED AS VRC-DILUENT013-DIL-VP) 53
11.4.2 VRC-HIVDNA016-00-VP (DNA) VACCINE OR PLACEBO (PBS) 53
11.5 STUDY PRODUCT AQUISITION 54
11.6 PHARMACY RECORDS 54

11.7 STUDY PRODUCT ACCOUNTABILITY 54
11.7.1 DOCUMENTATION *54*
11.7.2 DISPOSITION *54*

12.0 ASSESSMENTS 54

12.1 SAFETY ASSESSMENTS 54
12.1.1 LOCAL REACTOGENICITY *55*
12.1.2 SYSTEMIC REACTOGENICITY *55*
12.1.3 OTHER ADVERSE EVENTS *55*
12.1.4 CONCOMITANT MEDICATIONS *55*
12.1.5 ROUTINE LABORATORY PARAMETERS *55*
12.1.6 SPECIFIC SCREENING TESTS: *56*
12.2 IMMUNOGENICITY ASSESSMENTS 56
12.2.1 ANTIBODY RESPONSES *56*
12.2.2 CELLULAR RESPONSES *56*
12.2.3 PBMC, SERUM AND PLASMA STORAGE *56*
12.3 OTHER ASSESSMENTS 57
12.3.1 GENETIC TESTING *57*
12.3.2 HIV TEST *57*
12.3.3 PREGNANCY TEST *57*

13.0 ADVERSE EVENTS 57

13.1 DEFINITIONS 57
13.2 RELATIONSHIP OF ADVERSE EVENT TO INVESTIGATIONAL PRODUCT 58
13.2.1 CATEGORIES OF RELATEDNESS *58*
13.2.2 INVESTIGATIONAL PRODUCTS TO BE CONSIDERED FOR ASSESSING RELATEDNESS *59*
13.3 GRADING SEVERITY OF ADVERSE EVENTS 59
13.4 EXPEDITED REPORTING OF ADVERSE EVENTS 59
13.4.1 ADVERSE EVENTS REQUIRING EXPEDITED REPORTING TO DAIDS *59*
13.4.2 ADDITIONAL ADVERSE EVENTS THAT SHOULD BE REPORTED FOR ANY STUDY/TRIAL REQUIRING EXPEDITED REPORTING TO DAIDS *59*
13.4.3 METHOD AND TIMEFRAME FOR REPORTING OF INDIVIDUAL ADVERSE EVENTS AS PER DAIDS MANUAL *60*
13.4.4 ADVERSE EVENT EXPEDITED REPORTING PERIODS *61*
13.5 CLINICAL MANAGEMENT OF ADVERSE EVENTS 61
13.6 PREGNANCY 62
13.7 INTERCURRENT HIV INFECTION 62

14.0 MANAGEMENT OF HIV ISSUES DURING AND FOLLOWING STUDY 62

14.1 HIV TESTING 62
14.2 SOCIAL DISCRIMINATION AS A RESULT OF AN ANTIBODY RESPONSE TO VACCINE 63
14.3 HIV INFECTION 63
14.3.1 COUNSELING *63*
14.3.2 REFERRAL FOR SUPPORT AND/OR CARE *64*

15.0 DISCONTINUATION OF VACCINATIONS AND/OR WITHDRAWAL FROM STUDY 64

15.1 DISCONTINUATION OF VACCINATIONS IN INDIVIDUAL VOLUNTEER 64
15.2 FOLLOW UP AFTER DISCONTINUATION OF FURTHER VACCINATIONS 64
15.3 WITHDRAWAL FROM THE STUDY (EARLY TERMINATION) 65
15.4 FOLLOW-UP WITHDRAWAL FROM THE STUDY (EARLY TERMINATION) 65

16.0 DATA HANDLING 65

16.1 DATA COLLECTION AND RECORD KEEPING AT THE STUDY SITE 65
16.2 DATA COLLECTION AND TRANSFER AT THE IAVI CORE LABORATORY 66
16.3 DATA ENTRY AT THE STUDY SITE 66
16.4 DATA ANALYSIS 66

17.0 STATISTICAL CONSIDERATIONS 66
17.1 SAMPLE SIZE 66
17.2 STATISTICAL POWER AND ANALYSIS 66
18.0 QUALITY CONTROL AND QUALITY ASSURANCE 67
19.0 DATA AND BIOLOGICAL MATERIAL 67
20.0 ADMINISTRATIVE STRUCTURE 68
20.1 DATA SAFETY MONITORING BOARD (DSMB) 68
20.1.1 CONTENT OF INTERIM REVIEW 68
20.1.2 CRITERIA FOR PAUSING THE STUDY 68
20.2 STUDY SUPERVISION 70
20.3 STUDY MONITORING 71
20.4 INVESTIGATOR'S RECORDS 71
21.0 INDEMNITY 71
22.0 PUBLICATION 71
23.0 ETHICAL CONSIDERATIONS 72
REFERENCES 73
APPENDIX A - SCHEDULE OF PROCEDURE FOR VOLUNTEERS RECEIVING DNA+RAD5 VACCINE (GROUPS C AND D) 80
APPENDIX B - SCHEDULE OF PROCEDURE FOR VOLUNTEERS RECEIVING RAD5 VACCINE ONLY (GROUPS A AND B) 81
APPENDIX C - ADVERSE EVENT GRADING TOXICITY TABLE 83
APPENDIX D: IAVI V001 SAMPLE CONSENT INFORMATION SHEET 103
APPENDIX E: HIV TESTING ALGORITHM 115

ABBREVIATIONS

Abbreviation	Term
Ad5	Adenovirus serotype 5
AE	Adverse Event
AIDS	Acquired Immunodeficiency Syndrome
ALT	Alanine-Aminotransferase
CMI	Cell Mediated Immunity
CMV	Cytomegalovirus
CRF	Case Report Form
CTA	Clinical Trial Agreement
CTL	Cytotoxic T Lymphocyte
DCC	Data Coordinating Centre
DSMB	Data Safety Monitoring Board
DNA	Deoxyribonucleic Acid
EAE	Expedited Adverse Event
ELISA	Enzyme Linked Immunosorbent Assay
GCP	Good Clinical Practice
HIV	Human Immunodeficiency Virus
HLA	Human Leukocyte Antigen
HVTN	HIV Vaccine Trials Network
IAVI	International AIDS Vaccine Initiative
ICH	International Conference on Harmonization
ICS	Intracellular cytokine staining
LTR	Long Terminal Repeat
PU	Particle Units
PBMC	Peripheral Blood Mononuclear Cells
PCR	Polymerase Chain Reaction
PSRT	Protocol Safety Review Team

**Abbreviation**	**Term**
RAE	Reportable Adverse Event
RNA	Ribonucleic Acid
SAE	Serious Adverse Event
SHIV	Simian Human Immunodeficiency Virus
SOP	Standard Operating Procedure
SOM	Study Operations Manual
STD	Sexually Transmitted Disease
USMHRP	United States Military HIV Research Program
VRC	Vaccine Research Center

1.0. CONTACT INFORMATION

1.1 Principal Investigator Contact Information

Professor Job Bwayo<br>Principal Investigator<br>KAVI - University of Nairobi<br>PO Box 19676<br>Nairobi<br>Kenya	Telephone: +254 2 719628<br>Fax: +254 2 726626<br>Mobile: +254 733 830043<br>E-mail: bwayo@kaviuon.org
Dr. Etienne Karita<br>Principal Investigator<br>Projet San Francisco<br>169 Rue de la Revolution<br>Kigali<br>Rwanda	Telephone: +250 503233<br>Mobile: +250 08307245<br>Fax: +250 518373<br>E-mail: ekarita@psfiavi.rw

1.2 Sponsor Contact Information

Trial Sponsor<br>International AIDS Vaccine Initiative (IAVI)<br>110 William Street, 27th Floor<br>New York, NY 10038-3901	Telephone: + 1- 212-847-1111<br>Fax: + 1- 212-847-1113
Medical Expert<br>Pat Fast, MD, PhD<br>International AIDS Vaccine Initiative (IAVI)<br>110 William Street, 27th Floor<br>New York, NY 10038-3901	Telephone: + 1- 212-847-1068<br>Fax: + 1- 212-847-1113<br>Mobile: + 1- 646-206-6611<br>E-mail: pfast@iavi.org
Medical Monitor, Head Quarters (HQ)<br>Soe Than, MD, PhD<br>International AIDS Vaccine Initiative (IAVI)<br>110 William Street, 27th Floor<br>New York, NY 10038-3901	Telephone: + 1- 212-847-1044<br>Fax: + 1- 212-847-1113<br>Mobile: + 1- 646-346-9334<br>E-mail: sthan@iavi.org
Medical Monitor, Eastern Africa<br>Chrispin Kambili, MD,<br>International AIDS Vaccine Initiative (IAVI)<br>IAVI Kenya Regional Office<br>The Rahimtulla Tower, Floor 16<br>Upper Hill Road, PO Box 340<br>KNH 00202, Nairobi, Kenya	Telephone: 254-20-2731463<br>Fax: 254-20-2731462<br>Mobile: 254-722-239961<br>E-mail: ckambili@iavi.org

Core Laboratory
Professor Frances Gotch
Department of Immunology,
Faculty of Medicine, Imperial College
Chelsea and Westminster Hospital
369 Fulham Road
London SW10 9NH

Telephone: +44 (0)20 8746 8257/8
Fax: +44 (0) 20 8746 5997
E-mail f.gotch@imperial.ac.uk

Jill Gilmour, PhD
Director, Clinical Immunology, Core Laboratory
International AIDS Vaccine Initiative (IAVI)
110 William Street, 27th Floor
New York, NY 10038-3901

Telephone: + 1- 212-847-1057
Fax: + 1- 212-847-1113
Mobile: + 1- 646-206-6650
E-mail jgilmour@iavi.org

Tony Tarragona, PhD
Lab Manager/Administrator
IAVI Core Lab
Imperial College London, Faculty of Medicine
St. Stephen's Centre, 5th Floor
Chelsea Campus
369 Fulham Road
London SW10 9NH

Telephone: + 44 (0) 20 8846 6300
Fax: + 44 (0) 7940 510090
Mobile: +44 (0) 20 8846 6301
E-mail t.tarragona@imperial.ac.uk

1.3 DIADS Contact Information

DAIDS Medical Officer
Alan Fix, MD
Vaccine Clinical Research Branch
6700 B Rockledge Drive, Room 5132
Bethesda, MD 20817

Telephone: + 1- 301-594-9953
Fax: + 1- 240-465-6262
Email: afix@niaid.nih.gov

Protocol Pharmacist
Ana Martinez, Pharm D
DAIDS Pharmacy Affairs Branch (PAB)
6700 Rockledge Drive, Room 4115
Bethesda, MD 20817

Telephone: + 1- 301-435-3734
Fax: + 1- 301-402-1506
E-mail: amartinez@mail.nih.gov

2.0 SIGNATURE PAGE

The signatures below constitute the approval of this protocol and the appendices and provide the necessary assurances that this study will be conducted in compliance with the protocol, GCP and the applicable regulatory requirement(s).

Sponsor:

Signed: ______________________________ Date: ______________

Patricia Fast, MD, PhD
Director, Medical Affairs, IAVI

Signed: ______________________________ Date: ______________

Medical Monitor
Soe Than, MD, PhD
Medical Project Director, IAVI

Signed Date:
Principal Investigator:

Signed: ______________________________ Date: ______________

Name (please print): ______________________________

Name of institution (please print): ______________________________

3.0 INTRODUCTION AND BACKGROUND INFORMATION

The ongoing worldwide epidemic of the human immunodeficiency virus type 1 (HIV-1) remains one of the major global health challenges. HIV-1 causes the acquired immunodeficiency syndrome (AIDS), which is responsible for tremendous human suffering and economic loss throughout the world. Currently, over 42 million people are living with HIV-1 infection [1]. Without treatment, it is likely that nearly all of these will die of AIDS in the next 2 decades.

Since 1996, potent new antiretroviral therapies, including combination regimens with protease inhibitors, have created the possibility that HIV-1 infection might become a chronic, manageable disease among individuals with access to these medications. In the US, AIDS deaths are down to 16,000 per year as a result of the new antiretrovirals [2]. However, for the developing world, where over 95% of the 5 million annual incident HIV-1 infections occur [1], it is unlikely that these drugs will be widely accessible, due to many logistical challenges associated with their use.

Globally, 14,000 new infections occur each day. More than 3 million AIDS deaths occur per year [1], and nearly 20 million have died since the HIV epidemic began [3]. AIDS has become the leading infectious disease killer and the fourth leading cause of death overall. In severely affected countries, life expectancy has fallen by more than 10 years [1]. AIDS is the leading killer in Africa, with over 28 million Africans living with HIV/AIDS. Sub-Saharan Africa has been affected most; in 7 Sub-Saharan African countries, over 20% of adults (aged 15-49) are living with HIV/AIDS [3]. For example, in Botswana, 38.8% of adults aged 15 to 49 are infected with HIV, while in South Africa, 24.8% of women in antenatal clinics are infected [3].

The need for better education, better treatment access, better prevention programs, and better prevention technologies is therefore clear. Specifically, the need for a safe, effective, and affordable HIV-1 vaccine is paramount [4,5]. The ideal HIV-1 vaccine for global use should meet several of the following criteria:

- proven safety in healthy HIV-uninfected persons
- induction of long-lasting HIV-specific cell-mediated and humoral immunity capable of conferring protection against HIV
- tolerability
- potential for production in sufficient quantity to meet global needs
- affordability
- stability during distribution and storage

3.0.1 Kenya

In Kenya, HIV prevalence in the adult population (ages 15-49 years) is estimated at 9.4%. Life expectancy in Kenya has declined from 59 years to 49 years in the last two decades because of AIDS. Along with the AIDS epidemic, tuberculosis incidence has increased nine-fold. Prenatal seroprevalence in Kisumu, located in Western Kenya, is estimated at over 35%. [Retrieved from www.iavi.org]

The Kenya AIDS Vaccine Initiative (KAVI) is a collaborative venture between the University of Nairobi, Medical Research Council (MRC) of the UK and IAVI. KAVI has carried out the following vaccine research work on the candidate vaccine (DNA-MVA) which was developed through this collaboration:

• Since Feb 2001 two phase-I trials of DNA.HIVA and MVA.HIVA with varying dose regimens have been completed at the Kenyatta National Hospital (KNH). Two additional trials are ongoing at KNH and expected to be completed in 2Q 2005. Selected DNA/MVA trials were conducted concurrently in Uganda and the UK and there is a quality assurance network between these sites. This experience has shown that work carried out in African sites is of the same level of high scientific quality as work done in a developed country and with experience, enrollment rates have greatly increased.

• In the Kangemi slums of Nairobi KAVI is developing a cohort of individuals at high risk of HIV infection for participation in several feasibility protocols . So far more than 1300 individuals have been registered as being interested in participating in possible trials. IAVI feasibility study, protocol D will collect the blood samples from volunteers to observe the lab normal ranges of the population of the area. KAVI is also participating in other feasibility studies; protocols A, B and C, which will collect the information on prevalence and incidence of HIV infection and will study acute infection of HIV in infected subjects. .

In Kilifi in the Coast Province IAVI is working with the KEMRI-Coast Geographic Medical Research Centre to establish a suitable cohort to determine HIV incidence and prepare for efficacy trials. In addition community sensitizations, upgrading of voluntary testing and counseling (VCT) services and a large cross-sectional survey are being initiated.

IAVI is also working in Kenya and the region to create a supportive environment for trials and use of an eventual vaccine by:

• Briefing national leaders and soliciting their support for the vaccine development process

• Working with policy makers to develop national AIDS vaccine plans and other supportive policy measures

• National level outreach to key constituencies, including medical professionals, NGO's and religious leaders.

• Educating the general public in the communities surrounding the trial sites so as to reduce stigma about HIV/AIDS and participation in AIDS vaccine trials

• Encouraging informed coverage by media of HIV/AIDS in general and vaccine trials in particular.

3.0.2 Rwanda

In Rwanda, more than 370,00 people--almost 13 percent of the adult population--are living with HIV. About 180,000 of them have developed AIDS. HIV prevalence rates in urban areas in Rwanda range from 32 percent in low-risk groups to 56 percent in high-risk groups. Overall HIV prevalence in rural populations is estimated at 10 percent. AIDS claimed 36,000 lives in Rwanda in 1997 and AIDS is one of the three leading causes of death. By 2005, the crude death rate will be 40 percent higher due to AIDS than it was in 1990. Life expectancy has been reduced from 54 to 42 years in Rwanda as a result of AIDS. [Retrieved from www.iavi.org]

The Kigali site in Rwanda, a partnership between IAVI and Project San Francisco (PSF), an NGO led by Dr Susan Allen from Emory University, has the capacity at present to run a clinical trial. There are clinical facilities on-site and also a Clinical Safety Diagnostic laboratory and Immunology laboratory - both of which are also fully operational. All equipment is present on-site, and the local PI - Dr Etienne Karita, study physicians, nurses and laboratory team also on site.

Projet San Francisco has an extensive experience in VCT and HIV research since 1986. As of November, 2004, more than 700 HIV discordant couples and 80 HIV concordant negative couples are being followed in studies of HIV epidemiology and natural history.

This site will shortly begin work on one of the IAVI feasibility protocols, protocols C and has already started enrolling protocol D. Protocol C will involve collecting serum and PBMC samples from individuals recently infected with HIV - in order to study the early immune response to the virus, the nature of local circulating strains, and likelihood of cross-reactivity with HIV vaccine induced responses. Protocol D will test 200 to 400 individuals of each gender at each site who are not infected with HIV for laboratory tests that are performed to assess enrollment in HIV clinical trials. The volunteers will be representative of potential participants in an efficacy trial. The mean and standard deviation for each test will be used to select relevant inclusion and exclusion criteria and provide reference ranges in order to interpret adverse events. The site is also trained and ready for phase I/II studies. The site is also engaged in other studies; Heterosexual transmission study, Family Planning study and “Promote Demand/enhance supply” study utilizing influence network agents to recruit couples for CVCT

3.1 Rationale for multiclade vaccines for HIV-1

HIV has an enormous potential to generate genetically diverse variants because of the high error rate of reverse transcription, large viral burden, high replication rate, and pressure from immune defenses and anti-retroviral treatment. A recent analysis of genomic sequences from different regions in the world has identified at least 9 major subtypes (A, B, C, D, F, G, H, J and K) and dozens of recombinant forms [6]. Genetic diversity occurs within the individual patient as well with as much as 10% difference noted in the variable viral envelope sequences within a single infected patient [7-9]. A major challenge to designing HIV vaccines is to identify and target viral structures that are the critical determinants for protective humoral and cellular responses across the widest possible range of diversity. Genetic and antigenic variation is a particular problem for generating neutralizing antibody responses against HIV. The envelope glycoprotein is the most variable among the HIV genes, and the antibody response elicited tends to be type-specific. In contrast, CD8+ T cells can recognize epitopes from internal structural and regulatory proteins in addition to the envelope glycoprotein, and these frequently occur within more highly conserved regions [10]. For this reason, CD8+ T-cell responses may be more broadly cross-reactive than many antibody responses.

While the immunological relevance of HIV genetic diversity is not fully understood, studies of HIV vaccine volunteers and infected patients suggest that a successful global HIV vaccine program will need to protect against the diverse strains and subtypes predominating in the target populations, as has been the case for influenza. In a report from Merck & Co., Inc., the frequency of T cell immune responses recognizing clade A and clade C determinants appeared lower than to clade B in volunteers with a positive response to recombinant adenoviral-Gag (clade B) vaccine; among the 13 vaccinees with PBMC reactive to clade B peptides, 77% had PBMC recognizing pools of either clade A (10/13) or C (10/13) Gag peptides [11]. Lacking a thorough understanding of protective targets over the broad array of circulating HIV strains, a globally successful vaccine will most likely need to include many HIV antigens to represent the full range of variants that will need to be controlled. An analysis of HIV genetic variation and CTL epitopes based on data in the Los Alamos database has concluded that there may be an advantage in using a consensus sequence or an isolate from within each clade for capturing the largest number of relevant T cell epitopes in the vaccine immunogen [12,13]. The use of multivalent vaccines containing a defined mixture of immunogens from a number of prevalent subtypes is a feasible approach to achieve broadly protective HIV vaccines. The World Health Organization / UNAIDS HIV Vaccine Advisory Committee has recommended that candidate HIV

vaccines be designed based upon the strains prevalent in the country in which trials are to be conducted [14]. This approach is the foundation for the design of VRC-HIVDNA016-00-VP and VRC-HIVADV014-00-VP, which incorporate HIV *gag,* and *pol* (and *nef* in VRC-HIVDNA016-00-VP), genes from clade B as well as more diverse *env* genes from clades A, B, and C, which together represent the viral subtypes responsible for about 90% of new HIV infections in the world [15].

3.2 Rationale for DNA and adenovirus-vectored vaccines

3.2.1 DNA Vaccines

DNA vaccines have been tested in animal models and clinical trials for a variety of different pathogens, including influenza [16], malaria [17,18], and hepatitis B [19], in addition to HIV-1. Typically, these studies have shown that the vaccines are safe and well-tolerated, and there has been no evidence of induction of antinuclear or anti-double stranded DNA antibodies. However, overall immunogenicity has been generally disappointing and various strategies to improve the immunogenicity profile have been a major area of research over the last several years. Optimization strategies include studies of dose escalation and vaccination route comparison, and the published results so far suggest that DNA vaccines can be delivered safely at single doses ranging from 250 µg to 5 mg of DNA, with one trial reaching a cumulative dose of 20 mg after multiple vaccinations [11]. Also, it is postulated that the lack of potent immunogenicity in humans and nonhuman primates is a technical issue that can be overcome by experimentation with improved expression and/or enhanced delivery systems. Gene expression can be augmented by stronger gene enhancers and by improved translational efficiency of foreign DNA in mammalian cells through modifications to human codon sequences [20,21]. In addition, new DNA formulations given with cytokine adjuvants may improve immunogenicity [22-24].

Many novel HIV-specific DNA constructs have been tested in animal models, and the key research now focuses on immunogenicity and efficacy in nonhuman primates and humans [25]. Protection from SHIV challenge has been demonstrated after DNA vaccination alone, without a heterologous boost, when given in combination with a plasmid IL-2/Ig adjuvant [26] while several studies show protection in prime-boost experiments (see Section 3.2.4).

The first series of human clinical trials of an anti-HIV DNA vaccine involved 2 different vaccines, one expressing Env and Rev proteins, and the other expressing Gag and Pol proteins, which were developed by Apollon and Wyeth Lederle Vaccines, Inc. Two studies were carried out with the Env/Rev vaccine in HIV-infected subjects with CD4 counts >500. The Env/Rev vaccine was also tested in a small phase I study in HIV-negative subjects at the NIH Clinical Center. Shortly thereafter, the Gag/Pol DNA vaccine was tested in the NIAID-supported AIDS Vaccine Evaluation Group (AVEG 031), also in HIV-negative participants. In all these studies, the plasmid was administered with bupivacaine to enhance DNA uptake, gene expression, and immune responses. Doses ranged in these studies from 30 mcg to 3 mg, and vaccines were generally administered intramuscularly up to 4 times over a period of 6 months. These studies provided extensive safety data, including long-term follow-up for at least 2.5 years, and revealed no significant clinical or laboratory findings in any of the subjects [30, 31]. Sporadic low level T lymphocyte immune responses were seen in these studies, but the conclusions were generally limited by the small number of subjects per group [27].

Subsequently, a number of clinical trials with DNA vaccines have been conducted by the HVTN, Merck Vaccines, the International AIDS Vaccine Initiative (IAVI) and the Australian-New South Wales group. In most of the studies, the DNA vaccine was or will be administered as prime in prime-boost regimens with a variety of booster vaccines [28-31]. To date, the HVTN has tested

DNA vaccines developed by Emory University, Epimmune Inc., and Chiron Corp. in small phase I trials. The cumulative experience with all these vaccines reflects the findings in the non-HIV DNA vaccine studies, i.e., they are safe and well-tolerated, but induce only modest B- and T-cell stimulation when given alone [28-31].

The Vaccine Research Center, NIAID, NIH in collaboration with the Division of AIDS (DAIDS), NIAID, NIH has previously sponsored two intramural clinical trials with two different HIV-1 DNA vaccines. These are a single plasmid clade B Gag-Pol vaccine (VRC 4302) and a multiclade Gag-Pol-Nef and Env, 4-plasmid vaccine (VRC-HIVDNA009-00-VP). A total of 65 subjects have received these DNA vaccines in doses ranging from 0.5-8 mg, and the safety data to date indicates that these DNA vaccines are well tolerated and safe in healthy volunteers. The cellular immunogenicity of VRC-HIVDNA009-00-VP through week 12, as measured by intracellular cytokine staining (ICS) and enzyme-linked immunospot (ELISpot), show good CD4+ responses to the Env immunogens, but responses to the Gag, Pol and Nef are weak to absent. CD8+ responses are less pronounced than CD4+. In addition, VRC-HIVDNA009-00-VP is also now undergoing further evaluation in two other Phase I clinical trials conducted by the HVTN, HVTN 052, which is fully enrolled and evaluating two- versus three-injections of the multiclade DNA vaccine, VRC-HIVDNA009-00-VP, in 120 participants, and HVTN 044, is evaluating VRC-HIVDNA009-00-VP in combination with escalating doses of a plasmid IL-2/Ig adjuvant in 70 participants. Safety data from the studies again indicates that the DNA vaccines are well-tolerated, while immunogenicity results from the HVTN studies are currently pending. See Section 6 for a more complete presentation of the clinical data.

The rationale for the development of the current 6-plasmid vaccine, VRC-HIVDNA016-00-VP, derives from the poor immunogenicity of the Gag, Pol and Nef components of the 4-plasmid vaccine in the intramural study. The new generation vaccine separates the gag, pol and nef genes into separate plasmids, rather than having one plasmid that produces a fusion protein immunogen. In addition to splitting the genes into separate plasmids there are two other changes in the plasmid construction. These are: 1) a change in the promoter (to CMV/R) incorporated into these plasmids and 2) a 68 amino acid addition to the gag gene in the VRC 4401 (Gag protein only) plasmid as compared to the VRC 4306 (Gag-Pol-Nef fusion protein) plasmid that was in VRC-HIVDNA009-00-VP. The CMV/R promoter consists of the translational enhancer region of the CMV immediate early region 1 enhancer substituted with the 5'-untranslated HTLV-1 R-U5 region of the human T-cell leukemia virus type 1 (HTLV-1) long terminal repeat (LTR) to optimize gene expression further.

Preclinical data in monkeys with VRC-HIVDNA016-00-VP indicate that the separate gag, pol and nef plasmids generate more consistent and stronger immune responses to the Gag, Pol and Nef immunogens than were produced by the fusion protein immunogen. The clades A, B and C env plasmids in VRC-HIVDNA016-00-VP are identical to the three env plasmids in VRC-HIVDNA009-00-VP except that the promoter CMV/R has been used rather than the previous CMV promoter. Therefore the vaccine is expected to elicit immune responses against several proteins from a variety of HIV-1 strains and incorporates the safety features of previous HIV-1 vaccines, while potentially improving the immunogenicity.

3.2.2 Adenovirus-vectored vaccines

Replication-competent adenovirus vaccines in oral form have been administered to millions of military personnel, and over 42,000 have participated in well-controlled clinical trials to evaluate the safety and efficacy of these agents. These studies are well summarized in the literature [32-36] and have shown these vaccines to be safe and highly effective in preventing acute respiratory disease in recruits. The potential risk for live adenovirus to cause serious infection in

immunocompromised hosts and potential concerns for oncogenic potential of some serotypes have led to the development of replication-defective adenoviral vectors as vaccines and gene therapy vectors.

Adenoviral vectors incorporating a variety of expressed proteins have been used for gene therapy studies in many different human diseases, including cancer, cystic fibrosis, and cardiovascular disease, and have been delivered via several routes of administration including aerosol, intradermal, intramyocardial, intravenous, intrapleural and intratumoral [37,37-50]. Hundreds of human subjects have taken part in studies to evaluate adenoviral vectors as gene therapy agents, with many of these evaluating vectors based upon adenovirus type 5. Data from these and other studies found that side effects of adenoviral vectors were minor, local, or absent in most cases where the agents were administered intradermally or intramuscularly, with no significant vector-induced toxicities. One study assessed ten clinical trials for the safety parameters [51] and risk factors [48] of low ($<10^9$ particle units, PU)- and intermediate (10^9-10^{10} PU) – dose adenoviral vectors, delivered by various routes (nasal, bronchial, percutaneous injection into solid tumor, intradermal, epicardial injection of myocardium, and injection of skeletal muscle) to 90 individuals and 12 controls for treatment of a variety of conditions (cystic fibrosis, colon cancer metastases, severe coronary artery disease, and peripheral vascular disease). Local administration of these doses of adenoviral vectors appeared to be well tolerated. The major adverse events seemed to be primarily associated with characteristics of the study population (age, co-morbid conditions) and/or trial procedures (surgery) rather than dose, route of administration, expressed transgene, or number of administrations.

In the context of a large body of clinical research experience with adenovirus-based vectors, the death of a teenager in a gene therapy trial at the University of Pennsylvania prompted extensive reviews of safety data from both human and animal studies of these agents, many at the direction of the NIH Recombinant DNA Advisory Committee (RAC) and the Food and Drug Administration (FDA). The 18 year-old patient, suffering from ornithine transcarbamylase deficiency (OTCD), died after receiving a dose of 3.8×10^{13} PU of an E1/E4–deleted serotype 5 adenoviral vector directly into the hepatic artery [52]. An NIH report summarizing a review of clinical data from the case concluded that the participant's death was most likely due to a systemic adenoviral vector-induced shock syndrome, caused by a cytokine cascade that led to disseminated intravascular coagulation, acute respiratory distress syndrome, and multiorgan failure. Post-mortem bone marrow biopsy revealed red cell aplasia. The data suggested that the high dose of adenoviral vector delivered directly to the liver quickly saturated available receptors for the vector in that organ, leading to systemic dissemination which induced the fatal immune response [53].

In the University of Pennsylvania study, 19 OTCD patients received doses ranging from 1.86×10^{11} to 3.8×10^{13} E1/E4–deleted adenoviral vector particles infused directly into the hepatic circulation. The trial was halted after the death described above. Essentially all study subjects experienced one or more of the following: fevers, myalgias, nausea, and occasional emesis. Nearly all subjects showed a mild and transient thrombocytopenia without consistent abnormalities in coagulation, and higher dose levels were associated with subsequent abnormal liver function studies [52]. Similar results have been observed in animal studies [54-59] with the implication that systemic administration of adenoviral vectors might cause liver abnormalities and fever and might be associated with these symptoms more often than in patients treated via other routes of administration. Close clinical monitoring to detect such symptoms will be carried out in the present study and the adenoviral vectors will be administered by intramuscular injection, with a maximum study dose of 10^{10} PU.

Significant preclinical and clinical evidence also exists indicating that immune responses against HIV and other pathogens can be induced by direct gene transfer of immunogen-expressing genes via recombinant adenoviral vectors. Studies in non-human primates have shown that replication-incompetent serotype 5 (Ad5) adenoviral vectors can generate cellular immune responses against several viruses including HIV-1, SIV and Ebola [60-64]. Strong cellular immune responses were demonstrated using an IFN-γ ELISpot assay in baboons immunized with 10^{11} particles of replication-incompetent Ad5, with HIV-1 Gag-specific T-cells as high as 0.2% of circulating lymphocytes [61]. Rhesus macaques immunized with SIV Gag-based Ad5 vectors showed potent cytotoxic T lymphocyte (CTL) responses that correlated with protection (reduced CD4 loss, contained acute and chronic viremia, and reduced morbidity and mortality when challenged with a pathogenic strain of SIV) [60].

Preexisting vector immunity appears to attenuate immunologic responses to Ad5 vaccines in animal studies, but evidence exists that this attenuation may be overcome by increasing vaccine doses or using a prime-boost approach or both. In a mouse model, preexisting immunity to Ad5 resulted in markedly reduced cytotoxic T-cell responses to Ebola virus glycoprotein following immunization with an adenoviral vector expressing the Ebola antigen, compared with mice without prior vector immunity. Priming the mice with DNA encoding the Ebola antigen largely reversed this attenuation of cellular immune response to the glycoprotein after boosting with the adenoviral vector. Humoral immune responses to the Ebola antigen were also significantly reduced in mice with prior immunity to the adenoviral vector, but were not significantly increased by DNA priming [65]. Macaque studies done by Merck with their SIV Gag-Ad5 vaccine also indicate that immune responses are attenuated by preexisting immunity to Ad5, and that increasing the dose of the vaccine may overcome preexisting immunity [11,66].

Reports from Phase I dose escalation clinical trials of recombinant adenovirus type 5 (Ad5) vector vaccines developed by Merck & Co., Inc. and encoding clade B HIV-1 Gag, which examined doses ranging from 10^8 to 10^{11} PU per injection, described the vaccines as well tolerated [11,66]. These studies found moderate and sporadic injection site reactions, as well as sporadic fever with malaise, chills, and body aches, apparently more common at higher doses of the vaccine [11,66]. All of these adverse events were self-limited, and typically resolved within 48 hours. Preexisting immunity to Ad5 appeared to be associated with differences in side effects and immunogenicity. Local and systemic reactions were more common in participants with low Ad5 neutralizing antibody titers at baseline, and were attenuated following a booster dose of the adenoviral vector [66]. These studies demonstrated ELISpot responses 4 weeks following the second injection in 43%- 91% of participants, depending upon dose level administered and preexisting Ad5 neutralizing antibody titer. The frequency of responses to the Ad5 HIV-1 Gag vaccine appeared to be influenced by preexisting humoral immunity to Ad5 at all dose levels. The beneficial immunogenic effect of increasing the vaccine dose appeared most marked in the high (>1:200) titer preexisting Ad5 neutralizing antibody group, but the number of subjects was small [66].

3.2.3 Expected distribution of Ad5 neutralizing antibody titers

Extensive population data on the global distribution of neutralizing antibodies (NAb) to Ad5 are limited, but appears to be widespread in the regions where it circulates, and is often acquired at an early age [67]. Most of the data comes from studies done in the United States or other developed countries, and suggest that at baseline roughly one third of adults in developed countries will be seronegative (titer <1:10 - 1:20) for Ad5-neutralizing antibodies, and roughly one third will have titers above the 1:200 range [11,49,66,68,69]. Considerable variation exists among studies, however, with some reporting detectable neutralizing antibodies to Ad5 in as few as one third of US subjects [70,71].

An analysis of reports to WHO between 1967 and 1976 found no difference between Northern and Southern Hemispheres in the relative incidence of Ad5 among reported adenovirus infections [72]. However, recent studies in sub-Saharan Africa have found the seroprevalence of neutralizing antibodies to Ad5 to be approximately 80-90% [69,73]. Merck, WRAIR, the HVTN and the VRC have recently collected, or are in the process of collecting seroprevalence data on neutralizing titers to Ad5 in many countries that have a high incidence of HIV infection. Preliminary studies suggest that the average titer in the VRC assay is approximately 3-fold higher than the average titer in the Merck assay. The significant prevalence of Ad5 immunity in these countries necessitates clinical investigation of the effect of prior immunity on the immunogenicity of type 5 adenoviral vector vaccines, and the development of strategies to overcome this effect, before large-scale clinical trials of such HIV vaccines are undertaken.

Ad5 neutralizing antibody titers tested by Merck & Co., Inc. [74]

	<18 (%)	18-200 (%)	>200 (%)
Brazil (n=183)	8.2	29.5	62.3
Botswana (n=63)	8.8	26.5	64.7
Cameroon (n=225)	8.9	28.9	62.2
Malawi (n=49)	12.2	32.7	55.1
South Africa (n=182)	10.4	21.4	68.2
Thailand (n=1006)	6.0	13.7	80.3
US (n=779)	38.8	25.4	35.8

Ad5 neutralizing antibody titers tested by the VRC

	<12 (%)	12-500 (%)	>500 (%)
India (n=100)	19.8	29.2	51.0
Tanzania (n=53)	5.7	43.4	50.9
Uganda (n=110)	19.1	30.9	50.0
US (n=85)	50.6	10.6	38.8

3.2.4 Rationale for prime-boost vaccine strategies

Prime-boost regimens have shown promise in non-human primate models of HIV infection. Such regimens have the potential for raising high levels of immune responses. DNA vaccine priming followed by a recombinant viral vector boost with a modified vaccinia Ankara (rMVA) [4] or replication-deficient Ad5 [5] have been shown to attenuate a pathogenic SHIV infection in rhesus macaques, most likely by the generation of a $CD8^+$ CTL response. Merck Research Laboratories has published preclinical studies of priming for HIV-1 specific immunity using an adjuvant-formulated DNA vaccine followed with Ad5 vaccine boost. This generates levels of T-cell immune response that are comparable to those in naive animals receiving multiple high doses of Ad5 HIV-1 vaccines [6].

A prime-boost regimen has also shown promise in a preclinical model for prevention of Ebola virus infection in a study sponsored by the VRC, NIH and the Special Pathogens Branch, CDC. Cynomolgus macaques immunized with a combination DNA plasmid (a mixture of four DNA plasmids encoding glycoproteins from three Ebola strains and nucleoprotein from one strain) and boosted with a replication-deficient adenoviral vector encoding the glycoprotein resisted lethal viral challenge [7].

These preclinical studies suggest that DNA plasmid vaccine priming, followed by replication-defective adenoviral vector vaccine boost, as part of a carefully crafted vaccine strategy, can elicit potent and protective T-cell immune responses in animal models.

3.2.5 Rationale for conducting 3 separate protocols

This study, IAVI-001, and two others,RV 172 and HVTN 204, are conducted with the Vaccine Research Center (VRC), U.S. National Institute of Allergy and Infectious Diseases (NIAID) of the U.S. National Institutes of Health (NIH) to determine whether their six-plasmid multiclade HIV-1 DNA plasmid vaccine boosted by a multiclade HIV-1 recombinant adenovirus-5 vector (rAd5) vaccine should be carried forward into efficacy trials.

These three studies are being conducted by three independent trial networks and each network consists of non-overlapping national and international partnerships to execute vaccine clinical trials. The main objective of these three trials taken together is to attain safety and immunogenicity data sufficient to make a decision regarding efficacy testing of the vaccines. Phase II safety data that support the safety of this prime-boost vaccine approach as well as immunogenicity data that show distinct improvement, relative to products currently in Phase III clinical trials, in terms of breadth or quantitative immune responses, are needed to move into an efficacy trial.

All three networks are part of the Partnership for AIDS Vaccine Evaluation (PAVE). PAVE is a consortium of organizations that are actively involved in HIV vaccine clinical research and development and consists of the DAIDS, VRC, NIAID-sponsored HVTN and USMHRP, the U.S. Centers for Disease Control and Prevention (CDC), and IAVI.

The HIV Vaccine Trials Network (HVTN), the U.S. Military HIV Research Program (USMHRP), and the International AIDS Vaccine Initiative (IAVI), working closely with international host site investigators from each one of the countries to implement the studies, wrote HVTN 204, RV 172 and IAVI-V001. The combined study data will constitute the primary end of Phase II data set for safety and immunogenicity of this prime-boost Investigational New Drug (IND). It is crucial that all three trials, although independent from one another in implementation, are developed with sufficient harmonization to guarantee compatibility of the respective data sets.

All three trials will be conducted in accordance with current Good Clinical Practice (cGCP), International Conference on Harmonization (ICH) guidelines and the revised U.S. Code of Federal Regulations—21 and 45 CFRs. Copies of all the above documents and any other information and/or guidelines that are applicable for the safe and ethical conduct of the study will be available at each clinical site. The FDA will be the U.S. regulatory agency granting the IND application for the three studies. All national regulations for approval, implementation, and reporting will be followed in Kenya and Rwanda for V001.

The three organizations worked with the vaccine developer, VRC, as well as with the studies' sponsor, the Division of AIDS (DAIDS) NIAID-NIH, to harmonize all three trials during the development phase of the protocols. This harmonization was done to guarantee that the three trials will collect the same set of information from each study population using standardized terms and definitions, so that the data can be merged and used to evaluate this vaccination approach for efficacy testing. Although the populations will be different given the diverse geographic locations and associated variations in socio-economic and medical infrastructure, virtually identical inclusion and exclusion criteria will be applied among the three trials.

Furthermore, the harmonization of the three trials will continue through implementation period of the studies with continued communication and meetings, as needed, to discuss any elements of one trial that may affect and/or trigger modification of all three trials. Although safety management of each trial will be independently followed for each protocol, there will be a continued and open exchange of safety information channeled through the common DAIDS Medical Officer for all three trials and the vaccine developer. In addition, members of each network will be available to participate in other network's protocol safety reviews teams (PSRTs).

The three studies together, RV 172, HVTN 204, and IAVI-V001, are powered to detect small differences in safety and tolerability (between vaccine and placebo); and RV 172 and HVTN 204, the true Phase II studies, are powered to detect small differences in immunogenicity between the vaccine and placebo arms.

4.0 STUDY PRODUCT DESCRIPTIONS

4.1 Source and characteristics of the HIV gene inserts

The VRC DNA-HIV vaccine (VRC-HIVDNA016-00-VP) and VRC Ad5-HIV vaccine (VRC-HIVADV014-00-VP) contain largely matched HIV gene inserts, but they are not identical. The Gag and Pol proteins in both vaccines exhibit highly conserved domains. In the Ad5 vaccine they are present as a fusion protein, whereas they are expressed by separate plasmids in the DNA-HIV vaccine. In addition, the DNA-HIV vaccine includes a plasmid that encodes for Nef, whereas Nef is not included in the Ad5-HIV vaccine mixture. The clade A, B and C Env's are synthetic versions of modified, truncated envelope glycoproteins (gp145 in the DNA-HIV vaccine and a slightly shorter gp140 in the Ad5-HIV vaccine).

Gag
The synthetic *gag* gene in both vaccines is from HIV-1 clade B strain HXB2. In order to construct the fusion Gag-Pol expressed by the Ad5-HIV vaccine, a sequence encoding a 68 base pair amino acid sequence was deleted from the *gag* gene. No additional amino acid modifications were made to the *gag* gene in either vaccine.

Pol
The synthetic *pol* gene for both vaccines is from HIV-1 clade B strain NL4-3, and common mutations were introduced in the synthetic protease and reverse transcriptase genes. The protease modification prevents processing of the *pol* gene product, and reduces the potential for functional protease, reverse transcriptase and integrase enzymatic activity. In addition, the *pol* gene in the Ad5-HIV vaccine (VRC-HIVADV014-00-VP) is nonfunctional, because it is present as a fusion protein with the *gag* gene.

Nef
The DNA-HIV vaccine contains a plasmid that encodes for Nef from HIV-1 clade B strain NY5/BRU (LAV-1) recombinant clone pNL4-3. Nef is an accessory protein against which a vigorous T-cell response is mounted in natural infection. Two amino acids in the myristylation site in the HIV-1 *nef* gene were deleted to abrogate MHC class I and CD4+ down-regulation by the Nef protein [38, 39]. There is no *nef* gene included in the rAd5 mixture.

Env A, B and C
The sequences used to create the DNA plasmids encoding Env are derived from three HIV-1 CCR5-tropic strains of virus. These genes have been truncated and modified to improve immunogenicity, which has been demonstrated in mice [75] and monkeys [19]. The clade A Env protein sequence is from strain 92rw020. The clade B Env protein sequence is from strain HXB2 (X4-tropic), which was engineered to replace the region encoding HIV-1 envelope polyprotein amino acids 275 to 361 from X4gp160/h with the corresponding region from the BaL strain (CCR5-tropic). The V1 and V2 loops have been deleted from the clade B *env* gene in the Ad5-HIV vaccine (VRC-HIVADV014-00-VP) to improve stability and yield of the vector in the producer cell line. The clade C Env protein sequence is from strain 97ZA012.

4.2 DNA vaccine plasmids: VRC-HIVDNA016-00-VP

VRC-HIVDNA016-00-VP is composed of 6 closed, circular DNA plasmids that are each 16.67% (by weight) of the vaccine and are designed to express clade B HIV-1 Gag, Pol and Nef, and HIV-1 Env glycoprotein from clade A, clade B, and clade C. The DNA expression vectors are similar to those used for other candidate vaccines currently undergoing evaluation in clinical studies by the VRC and HVTN. The plasmid and host *E. coli* strain used in the production of the vaccine are characterized in accordance with the relevant sections of the “Points to Consider in the Production and Testing of New Drugs and Biologicals Produced by Recombinant DNA Technology” (1985), the “Supplement: Nucleic Acid Characterization and Genetic Stability” (1992), “Points to Consider in Human Somatic Cell Therapy and Gene Therapy” (1991, 1998), and “Points to Consider on Plasmid DNA Vaccines for Preventive Infectious Disease Indications” (1996).

4.2.1 Biojector® 2000 Needle-free Injection System
The plasmid DNA vaccine and the PBS placebo will be delivered by the intramuscular route, as this optimally elicits cellular immune responses in animal models [26]. The Biojector® 2000 Needle-Free Injection System will be used for these intramuscular injections. The Biojector® uses sterile, single-use syringes that deliver the study material intramuscularly using a compressed carbon dioxide cartridge. The study agent is expelled under pressure through a micro-orifice at high velocity in a fraction of a second.

The Biojector® 2000 Needle-Free Injection System has been approved by the US Food and Drug Administration (FDA) for parenteral administration of medications and immunizations. *In vivo* testing of this system by Bioject, Inc. (Bedminster, New Jersey, USA) has demonstrated effective immune responses with no associated serious adverse experiences. It has been shown to enhance the antibody response to hepatitis A vaccine in humans [76,77] and to HIV DNA vaccines in guinea pigs and rabbits (verbal communication, John Shiver). In studies to evaluate the potential for DNA integration in guinea pigs, it was shown that the extrachromosomal DNA copy number in skin and muscle at 6 weeks was several hundred–fold higher after Biojector® delivery than after needle delivery of plasmid [78]. This suggests the potential for more prolonged expression of the transgene, without integration. The Biojector® also has been shown to improve the transgene specific T-cell responses to malaria DNA vaccines compared to needle injection in studies in Rhesus macaques and humans [79-82]. A comparative study in humans, VRC008, will commence in Q1 2005 to further investigate the potential benefit that the Biojector® may contribute to immunogenicity.

4.3 Recombinant adenoviral vectors: VRC-HIVADV014-00-VP
The recombinant adenoviral vector product VRC-HIVADV014-00-VP (rAd5) is a replication-deficient, combination vaccine containing a mixture of four recombinant serotype 5 adenoviral

vectors. The process for constructing the four VRC-HIVADV014-00-VP recombinant adenoviral vectors is based upon a rapid vector construction system (AdFAST™, GenVec, Inc.), which generates four adenoviral vectors, each expressing one of the four HIV antigens gp140(A), gp140(B)dv12, gp140(C) and GagPol(B) driven by the cytomegalovirus (CMV) immediate-early promoter. To construct the adenoviral vector, the HIV-1 DNA sequence was subcloned using standard recombinant DNA techniques into an expression cassette in an E1-shuttle plasmid. Manufacturing is based upon production in a proprietary cell line (293-ORF6), yielding adenoviral vectors that are replication deficient. The vectors are purified using CsCl centrifugation. The product is formulated as a sterile liquid injectable dosage form for intramuscular injection.

The GV11 adenoviral backbone was chosen to reduce the risk of replication-competent adenovirus (RCA) generation during clinical production. The GV11 backbone contains deletions of two essential regions, E1 and E4, as well as a partial E3 deletion that render the vaccine product replication-deficient. The generation of RCA would require two independent recombination events in a single adenovirus genome, predicted to be an extremely rare event [83]. The Ad_{GV} (HIV).11D vectors contain HIV-1 antigen open reading frame (ORF) expression cassettes inserted to replace the deleted adenovirus E1 gene region. The other deleted adenovirus regions have been replaced with a transcriptionally inert spacer element (T1S1) that enhances production of the adenoviral vectors [84].

The 293-ORF6 cell line used to propagate these E1, E4 and partial E3 deleted vectors was developed at GenVec, Inc. These cells were constructed by stably transforming 293 cells (which are of human embryonic kidney origin) with an inducible E4-ORF6 expression cassette. This enables the cells to efficiently complement the E1-, E4-, and partial E3-deleted adenoviral vectors, provide increased transgene capacity and greatly reduce the potential to generate replication-competent adenovirus. The particular clone that has given rise to the cell line is the A232 clone. The multiclade adenoviral vector vaccine product, VRC-HIVADV014-00-VP, will be a 3:1:1:1 ratio of the adenoviral vectors that encode for HIV-1 Gag/Pol polyprotein from clade B and HIV-1 Env glycoproteins from clades A, B, and C, respectively. Final product meeting all test specifications will be released for use in the proposed clinical study. Vials will be filled to 1.2 mL volume with 1 $x10^{10}$ particle units/mL.

5.0 PRECLINICAL STUDIES

5.1 Summary of preclinical safety studies: VRC DNA plasmids

Prior to preparing the Investigational New Drug application (IND) for the 6-plasmid HIV-1 DNA vaccine, VRC-HIVDNA016-00-VP, a request was sent to the FDA for guidance and clarification regarding preclinical safety testing. The response received from the FDA was that the preclinical safety testing done on similar multi-component DNA plasmid products should be sufficient to support the IND for VRC-HIVDNA016-00-VP. The FDA advised that the amount of product administered to study subjects should not exceed that which had been administered in previous DNA vaccine studies (i.e., three vaccinations at the 8 mg dose level).

Preclinical toxicology studies have not been conducted with VRC-HIVDNA016-00-VP. In this section a brief summary of preclinical biodistribution and toxicology studies with a similar 6-plasmid DNA vaccine (VRC-HIVDNA006-00-VP) is provided. Given the high degree of homology for the DNA plasmids, the FDA concurred that preclinical testing of a single combination clade A/B/C product (VRC-HIVDNA006-00-VP) would preclude the necessity for

evaluating very similar products to assess toxicological effects, biodistribution, and potential integration. VRC-HIVDNA006-00-VP was not used in a clinical trial; however, the preclinical toxicology and biodistribution studies conducted with this vaccine were used to support the clinical testing of a similar 4-plasmid vaccine, VRC-HIVDNA009-00-VP, and a newer 6-plasmid vaccine, VRC-HIVDNA0016-00-VP which showed a more promising immunogenicity profile in preclinical studies.

TherImmune Research Corporation (Gaithersburg, MD) conducted preclinical safety studies under Good Laboratory Practices (GLP) using a DNA plasmid vaccine VRC-HIVDNA006-00-VP, including a single-dose biodistribution and a repeat-dose toxicity study, using intramuscular injections delivered by a needleless injection system, conducted in New Zealand White rabbits.

5.1.1 Biodistribution of VRC-HIVDNA006-00-VP

In the biodistribution study, evaluation on Day 8 showed that the highest signals were found in the tissues at or adjacent to the injection site. The magnitude of positive signal produced from Day 8 tissues were greatly diminished at each subsequent time point (Day 30 and Day 60), indicating clearance of the test article. All animals survived until the scheduled sacrifice, and no obvious difference in the biodistribution pattern was observed between female and male animals. The biodistribution studies are summarized in more detail in the Investigator's Brochures for the DNA plasmid vaccines VRC-HIVDNA009-00-VP and VRC-HIVDNA016-00-VP.

5.1.2 Repeat-toxicity of VRC-HIVDNA006-00-VP

The toxicity test revealed no apparent test-related alterations in clinical pathology parameters. Although there were some changes observed, they followed no obvious pattern. Therefore, the changes were attributed to individual animal variation in small sample-sized groups. While some organ weight parameters were affected, these alterations could not be verified in the clinical pathology or histology data and therefore were considered incidental.

More detail on the toxicology studies done with VRC-HIVDNA006-00-VP is provided in the Investigator's Brochures for the DNA plasmid vaccines VRC-HIVDNA009-00-VP and VRC-HIVDNA016-00-VP.

5.2 Summary of preclinical safety studies: VRC rAd and DNA prime followed by rAd boost

The Investigator's Brochures provides more extensive information about the preclinical safety studies for the DNA plasmid vaccine and rAd5 vaccine administered as single agents; the FDA did not require that additional biodistribution studies be performed on the combination regimen.

Gene Logic, Inc., Gaithersburg, MD, (formerly TherImmune Research Corporation) conducted a repeated-dose toxicology study of the 4-plasmid DNA vaccine (VRC-HIVDNA009-00-VP) in combination with the rAd5 vaccine (VRC-HIVADV014-00-VP) boost in New Zealand White rabbits under GLP. The DNA was delivered intramuscularly using a Biojector® device and the rAd5 vaccine was delivered intramuscularly via needle and syringe. This study determined effects of vaccination on mortality, clinical observations, body weights and changes, food consumption, ophthalmology, immunogenicity, organ weights and ratios, gross & histopathology, clinical chemistries, hematology, and coagulation parameters. More detail on the toxicology studies performed with rAd5 vaccine administered alone, and as part of a prime-boost combination is provided in the Investigator's Brochure for the rAd5 vaccine VRC-HIVADV014-00-VP.

5.2.1 Repeat-toxicity of DNA prime/rAd5 boost HIV vaccine regimen

DNA vaccine and PSB control were administered 4 times (study day 1, 22, 43, and 64), and adenoviral vector vaccine and diluent control were administered twice (study day 85 and 106). In this study, all animals survived to sacrifice and necropsy. In the adenoviral vector alone group, no treatment-related observations were made with regard to morbidity/clinical observations, body weights and changes, and ophthalmology. No prime-boost treatment-related observations were made with regard to morbidity/clinical observations and ophthalmology. However, possible prime-boost treatment effects were seen with body weights and changes, particularly in treated females. Differences began to be noted as early as study day 36, but became statistically significantly different from control females on study days 71, 78, 92, 99, and 108 for body weights and days 85-92 for body weight changes in prime-boost treated females. These animals continued to gain weight over the course of the study, but did not gain as much weight as the controls.

Additionally, minimal erythema was seen at the injection sites in a couple of treated males and one control female after the second adenoviral vector injection when given alone. In contrast, in the prime-boost regimen, vaccination with the DNA prime resulted in Draize observations of minimal to moderate edema and erythema in a few treated animals, increasing in frequency and severity with repeated dosing. This was a result of the combination of injection with the Biojector® and the active vaccination, as these observations also occurred in the control animals but to a lower amount and lesser degree. These findings were consistent with toxicology studies performed with the DNA vaccination alone. Boost (adenoviral vector delivered by needle and syringe) injections did not increase the frequency or severity of the Draize observations seen at earlier time points (after priming doses).

Clear treatment-related (adenoviral vector alone and prime-boost) observations were seen in gross and histopathology at the injection sites and in the histopathological findings of inflammation in the perineural tissue of the sciatic nerve (near the injection site). These latter lesions consisted of chronic inflammatory cells (small macrophages and lymphocytes) in the connective tissue around the sciatic nerve and in adjacent lymphatics and blood capillaries. This inflammation was likely the result of the distal injection sites with drainage toward proximal lymph nodes. The injection site reactions were less frequent and less severe in the recovery sacrifice animals than in the immediate sacrifice animals for both the adenoviral vector alone and the prime-boost regimens, demonstrating the reversibility of the injection site reactions.

Fever was seen in the 24 hours subsequent to the initial, but not second, adenoviral vector vaccination (adenoviral vector only arm), and was more striking in treated males than females. These fevers resolved by 48 hours. Likewise, fever was seen in treated males and females in the 24 hours subsequent to the initial, but only in the first 3 hours after the second adenoviral vector boost (only in treated females), in the prime-boost treated animals. These fevers resolved by 48 hours after the initial and 24 hours after the second (treated females only) adenoviral vector boost.

Food consumption in the rabbits was less in the 24 hours (adenoviral vector alone and prime-boost) to 48 hours (prime-boost) following each adenoviral vector vaccination, but resolved, and did not result in differences in body weights or changes in males or females inoculated with adenoviral vector alone or treated males in the prime-boost regimen. However, it did result in the noted body weight changes in treated females in the prime-boost regimen (although these differences began prior to exposure to adenoviral vector as discussed above).

There were many other observations, particularly in clinical chemistries and hematology parameters, which were unclear in their relationship to treatment because they either remained within the normal range for the species and laboratory (even though there were statistically significant differences from matched control animals on study) or they were outside the normal range and different from the control animals on study but were not consistent between genders or across time points. None of these findings correlated with clinical observations or gross or histopathological findings. Of note among these, however, was the finding of statistically significant (from matched controls on study) elevated triglycerides on the second day subsequent to the initial adenoviral vector inoculation in both treated males (mean was 1.5 times the upper limit of normal - ULN) and females (mean was >1 but <1.5 time the ULN) receiving adenoviral vector vaccination alone and on the day subsequent to the initial adenoviral vector boost in treated males (mean was between 3-4 times the ULN), but not treated females, receiving the prime-boost regimen.

5.3 Summary of preclinical immunogenicity studies

Although there are no animal models of HIV-1 infection that is highly predictive of what will be seen with vaccination in human clinical trials, immunogenicity studies are typically carried out in mice and monkeys, and challenge studies are often carried out in macaques with the SIV analogues of the candidate HIV-1 vaccines. As shown in the table below, preclinical immunogenicity studies were conducted in mice, rabbits and non-human primates with several of the VRC-HIV DNA and Ad5-vectored vaccines at the VRC and Beth Israel Deaconess Medical Center, Harvard Medical School (Boston, MA). The immunogenicity studies in mice and rabbits, as well as the non-human primate studies VRC-02-035 and VRC-03-060 were conducted using materials from the same exact bulk lots as those intended for clinical trials and formulated exactly as clinical trial material. In summary, the studies shown below in Table 5.3 indicate that the previous generation of VRC DNA and Ad5 vaccines was able to induce partial protection from challenge in the SHIV model system, and that the improvements made with the current generation of both VRC candidate HIV vaccines (VRC-HIVDNA-016-00-VP and VRC-HIVADV-014-00-VP) can reasonably be expected to induce immune responses of greater magnitude and breadth than those from the earlier constructs. More detail on these studies can be found in the Investigator’s Brochure.

Table 5.3: Pre-clinical Immunogenicity Studies with VRC DNA and Ad5 vaccines

Test System	Route	Dose	Treatments per Animal	Treatment Period	Study Duration	Conclusions	References
Mouse	i.m.	rAd: 1 x 10^{11}PU	1	3 wks	4 wks	VRC-HIVADV014-00-VP immunization elicited humoral and cellular immune responses in mice.	Summary in VRC-HIVADV014-00-VP IB Section 5.3.1
Mouse	i.m.	DNA: VRC-HIVDNA016-00-VP, 50µg	1	0 day	3 wks	Vaccination with the CMV/R plasmid encoding gag-pol-nef fusion protein (contained in VRC-HIVDNA016-00-VP vaccine product) elicits higher HIV-1specific cellular immune responses in mice than the unmodified 1012 plasmid encoding the same fusion protein (contained in VRC-HIVDNA009-00-VP vaccine product).	Summary in VRC-HIVDNA016-00-VP IB Section 4.2.1
Rabbit	i.m.	DNA: VRC-HIVDNA009-00-VP, 4 mg<br><br>rAd: 1 x 10^{11} PU	6	106 days	119 days	Immunization VRC-HIVDNA009-00-VP DNA prime followed by VRC-HIVADV014-00-VP boost elicited humoral immune responses in rabbits.	Gene Logic (formerly TherImmune) Repeated Dose Toxicity Study #1195-114 and Summary in VRC-HIVADV014-00-VP IB Section 5.3.2
Rhesus macaques	i.m.	DNA: (SIV)<br>10 mg<br><br>rAd: 2 x 10^{12} PU	3 DNA + 1 rAd	0, 4, 8 (DNA) + 26 wks (rAd)	64 wks	Immunization with SIVmac239 *gag-pol-nef* + (89.6P or HXB2 BaL *env* DNA prime/rAd (no *nef*) boost confers partial protection against SHIV-89.6 challenge. Matched (89.6P) or mismatched (HXB2 BaL) Env immunogens conferred better immunity than vaccination without Env.	Study ASP-015<br>Letvin, et al. Virology (2004) 78:7490-7
Rhesus macaques	i.m.	DNA: (SIV)<br>9 mg<br><br>rAd: 1 x 10^{12} PU	3 DNA + 1 rAd	0, 4, 8 wks (DNA) + 26 wks (rAd)	42 wks	Immunization with a four component multiclade vaccine resulted in broader responses without loss of immunogenicity to any component as compared with the vaccines consisting of plasmids and rAd expressing SIV Gag/Pol-(Nef) and HIV-1 Env from a single clade.	Study VRC-026A<br>Summary in VRC-HIVADV014-00-VP IB Section 5.4.1
Cynomolgus macaques	i.m.	DNA: 4-plasmid VRC-HIVDNA009-00-VP or 6-plasmid HIV-DNA016-00-VP, 8 mg<br><br>rAd:VRC-HIVADV014-00-VP 1 x 10^{11} PU	3 DNA<br>1 rAd	0, 4, 8 wks (DNA) + 38 wks (4-plasmid rAd boost) or 24 wks (6-plasmid rAd boost)	58 wks	Cynomolgus macaques receiving DNA prime/rAd boost immunization with the 6-plasmid DNA vaccine that expresses HIV-1 Gag, Pol, Nef and clade A, B and C Env (VRC-HIVDNA016-00-VP), and boosted with rAd expressing HIV-1 Gag/Pol and 3 Env, elicited cellular immune responses to all vaccine antigens. The 6-plasmid prime appeared t o be more immunogenic than the 4-plasmid DNA vaccine prime.	Study VRC-02-035<br>Summary in VRC-HIVDNA016-00-VP IB Section 2.3.2

IB = Investigator Brochure
rAd = VRC-HIVADV014-00-VP.
Administered intramuscularly by needle and syringe (rAd) or Biojector 2000® (DNA)
PU = particle unit

6.0 CLINICAL STUDIES

6.1 DNA plasmid vaccine trials

Human trials conducted with other plasmid DNA vaccines using similar clinical products have revealed no significant toxicity, autoimmunity, or other adverse reactions [14,17,27]. The status of completed and ongoing Phase I VRC, HVTN, and USMHRP studies with HIV-1 plasmid DNA vaccines developed by the VRC are summarized in Table 6.1 and briefly described below:

Table 6.1 Multiclade HIV DNA vaccine experience in uninfected participants as of May 2005

HIV DNA vaccine formulation	Study	Dose (mg)	# active doses planned (participants in active arm)	# active doses to date (participants in active arm)	Comment
**VRC-HIVDNA009-00-VP**<br>4 plasmids (multiclade):<br>clade B *gag-pol-nef,*<br>clade A *env*,<br>clade B *env*,<br>clade C *env*.	VRC 004	2.0<br>4.0<br>8.0	15 (5)<br>60 (20)<br>45 (15)	15 (5)<br>60 (20)<br>44 (15)	Vaccinations completed and study unblinded; also included 10 placebo participants (29 placebo injections)
	HVTN 052	4.0	300 (120)	≥292 (120)	Of 540 blinded doses (300 active and 240 placebo) planned, 8 total were not given (still blinded).
	RV 156	4.0	45 (15)	~30 (15)	First enrollment January 2005; also includes 15 placebo participants (45 placebo injections)
**VRC-HIVDNA016-00-VP**<br>6 plasmids (multiclade):<br>clade B *gag,*<br>clade B *pol*,<br>clade B *nef*,<br>clade A *env*,<br>clade B *env*,<br>clade C *env*.	VRC 007	4.0	45 (15)	44 (15)	Vaccinations completed; open label; no placebos.
	VRC 008	4.0	120 (40)	0	Project initiation May 2005
Summary: A dose range of 2.0 mg to 8.0 mg was evaluated for the 4-plasmid DNA vaccine. The majority of experience is with the 4.0 mg dose. This dose range was well tolerated.<br>Phase I studies (uninfected participants) include plans for 630 vaccine injections in 230 participants.<br>As of May 5, 2005 190 uninfected participants who have received one or more vaccine injections..<br>Note: Experience with a clade B single plasmid vaccine, with the 4-plasmid DNA vaccine in combination with an IL2/Ig adjuvant, and with the 4-plasmid DNA vaccine in HIV-infected participants is not shown. These studies together include more than 50 vaccinees to date.					

6.1.1 Protocol VRC 001

The VRC completed a Phase I, randomized, controlled, double-blinded dose escalation study, VRC 001 (BB-IND 9782), to evaluate safety, tolerability, dose and immune response of an HIV plasmid DNA vaccine expressing a clade B Gag-Pol fusion protein (pGag (del fs) PolΔPRΔRTΔIN/h; VRC-4302), which is similar in composition to VRC-HIVDNA009-00-VP and VRC-HIVDNA016-00-VP. Each of three groups of seven healthy, HIV-negative volunteers received a constant dose of the vaccine (5 people) or a phosphate buffered saline (PBS) control (2 people) by intramuscular inoculation. Once safety was established, successive groups received a higher dose. Study groups received three immunizations containing 0.5 mg (Group

1), 1.5 mg (Group 2), or 4.0 mg (Group 3) of the DNA vaccine or placebo. All 21 participants received all planned injections, which were well tolerated. All participants completed the study. There were no serious adverse events attributed to the study agent.

6.1.2 Protocol VRC 004

Protocol VRC-004 (03-I-0022) is a Phase I randomized, controlled, double-blinded dose escalation study to evaluate safety, tolerability, dose and immune response of a multiclade HIV plasmid DNA vaccine identified as VRC-HIVDNA009-00-VP. This study opened to accrual in November 2002 and was fully enrolled with 50 healthy HIV-negative participants in August 2003. Forty participants received vaccine injections and 10 participants received placebo injections. The three-injection schedule (administered at Weeks 0, 4 and 8), was completed in 5 of 5 participants randomized to 2 mg vaccine injections, 20 of 20 participants randomized to 4 mg vaccine injections, 14 of 15 participants randomized to 8 mg vaccine injections and 9 of 10 participants randomized to placebo injections. The unblinded final study results indicate that the vaccine injections were as well tolerated as the placebo injections.

In the vaccine groups, there were three adverse events possibly related to vaccine that required expedited reporting to the IND sponsor. These were a grade 3 asymptomatic neutropenia with onset 27 days after 3rd vaccination (4 mg group), a grade 3 urticaria with onset 4 days after 3rd vaccination (4 mg group) and a grade 2 maculopapular rash with onset 27 days after 2nd vaccination (8 mg group). All resolved without sequelae. Other factors in the occurrence of the urticaria include concomitant bladder infection, yeast infection and multiple antibiotics. The rash resulted in discontinuation from the vaccination schedule after the 2nd injection and it was clinically consistent with either a drug eruption or a viral exanthem. The diary cards indicate that vaccine injections were well tolerated. No participants reported severe symptoms on diary cards. Most participants (80-100% per group), including placebo recipients, reported at least one local symptom (pain/tenderness, induration or erythema) at some point in the 7 days after an injection. Most participants (70-80% per group), including placebo recipients, also reported at least one systemic symptom in the 7 days after an injection. No vaccine recipients reported fever. Chills and nausea were infrequent in all participants (0-20% per dose group). Headache and myalgia were reported in 20-50% per dose group. Malaise was the most common systemic symptom, occurring in 50-60% of vaccinees and 40% of placebo recipients at least once in the 7 days following a study injection. During the study the most frequently recorded laboratory adverse events included asymptomatic hyperglycemia and hypoglycemia. The unblinded data show that placebo recipients had higher incidence of both hyperglycemia and hypoglycemia and these data support the clinical impression that variations in blood glucose are unrelated to study vaccinations.

Preliminary immunogenicity data through Week 12 from the VRC 004 study, when sorted by treatment assignment indicate that $CD4^+$ responses were detected in nearly 100% of recipients at all dose levels. $CD8^+$ responses were detected in nearly half. The greatest responses (in frequency and magnitude) were generally observed as directed against Env. The immunogenicity response to the Gag, Pol and Nef are weak to absent. There is a trend to greater responses in the 4 mg and 8 mg dose compared to the 2 mg dose, although not statistically significant. There was a statistically significant increase in the response after 3 injections compared to 2 injections at both the 4 mg and 8 mg dose levels, although there is no way to determine if this was due to the 3rd injection or simply a maturation of the response following the 2nd injection. Definitive responses are first detectable with the 4 mg and 8 mg dose at the 6-week time point (2 weeks after the second injection). When compared with the 8 mg dose, the 4 mg dose offers the combination of a good safety profile, greater ease of administration, and approximately equivalent cellular immunogenicity.

Fourteen of 35 vaccinees in the 4 mg and 8 mg groups had a positive ELISA at one or more points between Week 8 and Week 52 when tested by a commercial HIV antibody test. The optical density (O.D.) of the ELISA results usually decreased over time; six participants were ELISA positive by the commercial assay at Week 52.

Some VRC 004 participants may be offered the opportunity to enroll in a separate study (VRC 009) in which they could receive a single boost injection of VRC-HIVADV014-00-VP, a VRC recombinant adenoviral vector vaccine, at 1×10^{10} PU. That study will provide safety data of the adenoviral vector vaccine as a booster vaccine and will complement the immunogenicity data obtained in HVTN 057.

6.1.3 Protocol HVTN 044

Protocol HVTN 044 (BB-IND 10914) is a placebo-controlled Phase I study to evaluate the safety and immunogenicity of the 4 mg dose of the 4 plasmid multiclade VRC-HIVDNA009-00-VP vaccine administered in combination with escalating doses (0.1, 0.5, 1.5 and 4.0 mg) of a plasmid cytokine adjuvant VRC-ADJDNA004-IL2-VP (IL-2/Ig) in 70 HIV-negative participants. For this study, the multiclade DNA vaccine is being administered using the Biojector apparatus. The study opened for enrollment in December 2003, but is still accruing due to the multiple built-in safety pauses for the dose escalation of the adjuvant. As of March 2005, 54 out of 70 participants have been enrolled into this trial, 38 of which could have received the multiclade VRC-HIVDNA009-00-VP vaccine. All enrolled participants have received the first injection, 39 have received the 2nd injection, 31 have received the 3rd injection, and 17 have received all 4 injections.

Local reactions to the study vaccine, including mild or moderate pain and/or tenderness at the injection site, were reported by 47 participants (81% of these were mild in severity). Mild erythema and/or induration were reported by 21 participants. Systemic reactogenicity symptoms (i.e., malaise, myalgia, headache, nausea, vomiting, chills or arthralgia) were experienced by 34 participants. The vast majority (79%) of these were mild in severity. There was one report of severe (grade 3) malaise which is discussed in greater detail below.

There were two grade 3 events possibly related to vaccine that required expedited reporting to the IND sponsor. One participant developed a grade 3 decrease in CD4 count that was considered possibly related to the study vaccine. This participant was enrolled in the study with a preexisting grade 2 CD4 lymphopenia, and is currently being worked up for a possible diagnosis of sarcoidosis. If confirmed, sarcoidosis would be the likely contributor to the low CD4 counts. The HVTN Safety Monitoring Board reviewed this event at the time of its occurrence in an unblinded fashion and determined that it was safe to continue the study. Another participant developed grade 3 severe malaise 1 day after the 3rd vaccination. These symptoms resolved by day 3 and further vaccinations were discontinued for this participant. This event was also reviewed in an unblinded fashion by the HVTN Safety Monitoring Board, which determined that it was safe to proceed with the study.

6.1.4 Protocol HVTN 052

The HVTN is conducting a Phase IB study, HVTN 052 (BB-IND 10681), to evaluate the safety and immunogenicity of the 4-plasmid VRC-HIVDNA-009-00-VP in a two- versus a three-injection regimen in 180 participants (120 vaccine/60 control). The schedule being compared is 0, 4 and 8 weeks versus 0 and 8 weeks, and all injections are given by Biojector. The study opened for enrollment in December 2003 and accrual was completed on October 19, 2004. As

of May 2005, all 180 participants are beyond Week 8; 8 of the 540 planned injections were not administered. The study remains blinded.

Local reactions of pain and/or tenderness at the injection site were reported by 158 (88%) of the participants. Mild erythema and/or induration were reported by 36% of the participants. All local reactions were mild or moderate, except in one participant, who experienced an episode of severe injection site pain that started 30 minutes after the first vaccination. The pain was mild by the following day and resolved by day 4.

Sixty percent of the participants experienced mild or moderate symptoms of systemic reactogenicity (malaise, myalgia, headache, nausea, vomiting, chills or arthralgia), the vast majority of which (83%) were mild.

As of February 2005, an unblinded review of HVTN 052 safety data by the HVTN Safety Monitoring Board indicated that there were no significant differences in AEs or SAEs across treatment groups. As of March 2005, there have been three adverse events that required expedited reporting to the IND sponsor, excluding those ultimately deemed not related. One participant experienced severe injection site pain that was mild by the day after vaccination as discussed above. A 41-year-old male participant developed an increased glucose (261 mg/dl) at visit 5, 13 days after the 2nd vaccination. All other laboratory values were within normal limits at this visit. The participant had a family history of diabetes mellitus and a BMI of 35.7 and reported eating a very large breakfast, as well as soda and cookies, prior to the blood draw. This event was ultimately attributed to a new diagnosis of diabetes mellitus and determined unlikely to be related to vaccination. One 44-year-old male participant developed grade 3 platelet elevation. The participant had pre-enrollment platelet counts that met the inclusion criterion but were greater than the site's upper limit of normal. He is asymptomatic and continues protocol visits, during which the elevations have persisted.

6.1.5 Protocol RV 156

RV 156 is a Phase I clinical trial to evaluate the safety and immunogenicity of the multiclade HIV-1 DNA plasmid vaccine, VRC-HIVDNA009-00-VP, in uninfected adult volunteers in Uganda. The study has enrolled 30 participants, half of whom have received three 4 mg doses of the 4-plasmid DNA vaccine and half of whom received placebo. Enrollment started in January 2005, and it is anticipated that enrollment will be completed in June 2005. As of March 25, 2005, no notable vaccine-associated adverse events had been identified.

6.1.6 Protocol VRC 007

VRC 007 (04-I-0254) is the first Phase I study of the 6-plasmid DNA vaccine, VRC-HIVDNA016-00-VP. This open-label study enrolled 15 participants between August 17, 2004 and October 28, 2004. Fourteen of the 15 participants received 3 intramuscular injections of a 4 mg dose of vaccine administered by Biojector; one participant was lost to follow-up after two vaccinations. The last study vaccination was administered on December 22, 2004. This summary represents interim results through May 4, 2005. No participants reported fever following vaccination. Reactogenicity, as reported on 43 diary cards, was none to mild except that two participants reported moderate injection site pain and one participant reported moderate nausea and malaise. The only adverse event requiring expedited reporting to the IND sponsor was a grade 3 generalized urticaria. The subject had reported starting an antihistamine about 2 weeks after first vaccination but reported at that time that the reason was latex allergy. While being screened for the rollover booster study, VRC 010, it was learned that the subject had experienced generalized urticaria around the time of the second vaccination when the supply of antihistamine ran out. As of May 2005 the subject has chronic urticaria that are well controlled

by antihistamine. Evaluation is ongoing. The etiology is unknown but at this time the chronic urticaria is assessed as possibly related to study vaccine. To date, there have been two moderate (grade 2) adverse events possibly attributed to vaccine. These were intermittent dizziness of 2 days duration beginning 13 days after the second vaccination in one participant (this participant received the third vaccination without recurrence of symptoms) and asymptomatic hypoglycemia in another participant, first noted at the follow-up visit that was 14 days after the third vaccination. The last safety evaluation of the participant lost to follow-up was by telephone one day after the second vaccination; at that time the participant reported no side effects from the vaccination. The last study visit for all participants is projected to occur in June 2005.

An unexpected local injection site reaction for this DNA vaccine has been observed. Mild cutaneous lesions (0.5-1.0 cm diameter) at the vaccination site occurred after 4 of 44 (9%) vaccinations administered; these occurred in 3 of 15 (20%) participants. Participants were routinely asked to call if they experience any unusual problem after study vaccinations. The vaccination site cutaneous lesions did not alarm participants enough to prompt them to contact the VRC Clinic prior to their next regularly-scheduled visit. In retrospect, three participants reported that they experienced skin lesions that started as a small papule or vesicle within 3 days after vaccination. After a few days the papule or vesicle unroofed and a scab formed. There was surrounding mild erythema and mild induration. After the scab came off, the skin healed without treatment. None of the cutaneous lesions were associated with pustular exudates, fever, rash or urticaria. They did not appear to be either a local infection or allergic reaction.

The first three cutaneous lesions were discovered at the first post-vaccination clinic visit (days 14 ± 3 Day); at that time they were largely resolved. The fourth cutaneous lesion, was examined in the clinic while still in an active stage, and it was biopsied at post-vaccination day 6. This biopsy demonstrated a microscopic subcutaneous and dermal perivascular lymphocytic infiltrate. The infiltrate was composed almost exclusively of CD3 positive cells, including both $CD4^+$ and $CD8^+$ T cells. There were rare eosinophils present and rare giant cells noted. The process appeared to be primarily a subcutaneous and dermal response to vaccination with cutaneous manifestations.

The reason these reactions have been seen in VRC 007 and not studies evaluating other DNA vaccines delivered intramuscularly by Biojector is not known. Whether these reactions correlate with the strength of the vaccine-induced immune response is also not yet known. Six of the 14 subjects remaining on study have had a vaccine-induced positive HIV ELISA by a commercial test at one or more timepoint; this includes all three subjects who had a cutaneous lesion. Preliminary immunogenicity data from VRC 007 (6-plasmid product) suggests the Env-specific T cell responses are similar to those seen in VRC 004 (4-plasmid product), and now Gag- and Nef-specific responses are also present.

6.2 Recombinant adenoviral vector vaccine trials

The status of completed and ongoing Phase I VRC and HVTN studies with the HIV-1 adenoviral vector vaccine developed by the VRC are summarized in Table 6.2 and briefly described below:

Table 6.2 Multiclade HIV adenoviral vaccine, VRC-HIVADV014-00-VP, experience in uninfected participants as of May 2005

Study	Single dose (PU)	# active doses planned	# active doses to date	Comment
VRC 006	10^9<br>10^{10}<br>10^{11}	10<br>10<br>10	10<br>10<br>10	Vaccinations completed; also included 6 placebo participants (6 placebo injections)
VRC 008	10^9<br>10^{10}	20<br>20	0<br>0	Project initiation May 2005
VRC 009	10^{10}	≤ 32	8	First enrolled January 28, 2005.
VRC 010	10^{10}	≤ 14	1	Project initiation May 2005
HVTN 054	10^{10}<br>10^{11}	20<br>20	≤5<br>0	Project initiation April 2005
HVTN 057	10^{10}	60	60	Boosting of HVTN 052 (4-plasmid DNA x2 or x3); includes 10 placebo subjects; Ad5Ab not a factor in eligibility or randomization; 70 participants enrolled in the study (still blinded)
Summary: A dose range of 10^9 PU to 10^{11} PU was evaluated. The majority of the experience is with the 10^{10} PU dose. This dose range was well tolerated. The experience to date includes 84 injections of the 10^{10} PU dose and 10 injections of the 10^{11} PU dose.<br><br>Note: Preliminary VRC 006 data suggests that at least some subjects with pre-existing adenovirus serotype 5 antibody (Ad5Ab) show an immune response to the vaccine.				

6.2.1 Protocol VRC 006

The Vaccine Research Center (VRC) is conducting VRC 006 (04-I-0172), "A Phase I Clinical Trial to Evaluate the Safety and Immunogenicity of a Recombinant Multiclade HIV-1 Adenoviral Vector Vaccine, VRC-HIVADV014-00-VP, in Uninfected Adult Volunteers." This is a randomized, placebo-controlled, double-blinded, dose escalation study to examine safety, tolerability and immune response following a single injection of VRC-HIVADV014-00-VP at a dose of 10^9 PU, 10^{10} PU, or 10^{11} PU. Each group includes 12 participants (10 vaccine; 2 placebo). VRC 006 was initiated on July 19, 2004 and the study completed enrollment of 36 participants on November 10, 2004. The NIAID Intramural Data and Safety Monitoring Board (DSMB) reviewed the preliminary safety data through 14 days of follow-up prior to each dose escalation. The preliminary data indicate that the vaccine appears to be safe for healthy participants at the three dose levels evaluated. The 10^9 and 10^{10} PU dose levels are associated with less reactogenicity than the 10^{11} PU dose level. In both the 10^9 and 10^{10} PU dose groups the local and systemic parameters recorded on the 5-day diary card were none to mild in severity and none of the participants experienced fever. In the 10^{11} PU dose group, four participants reported fever on Day 1 (3 mild and 1 moderate in severity). Each of the four participants with fever also reported moderate headache on Day 1 and three of these participants also reported at least one other moderate systemic parameter (malaise, myalgia, chills). Two participants without fever reported at least one moderate systemic symptom (malaise, myalgia, nausea). One participant in the 10^{11} PU dose group reported moderate injection site pain; injection site reactogenicity was otherwise none or mild.

As of April 27, 2005, there has been one grade 4 (potentially life-threatening) and three grade 2 (moderate) adverse events that are possibly related to vaccination. The study remains blinded to vaccine vs. placebo injection assignments. The grade 4 adverse event was a seizure that occurred 64 days after study injection in a healthy participant in the 10^{11} PU dose group who had a history of a single seizure three years prior to study enrollment. Following the review of past medical records and test results and given the history of a prior seizure and the timing of the event more than 2 months after study injection, the seizure was assessed as unrelated to study agent. The grade 2 adverse events possibly related to study agent include: 1) asymptomatic neutropenia noted 21 days after study injection in a participant known to sometimes have asymptomatic low neutrophil counts prior to enrollment; 2) diarrhea (duration one day) in a different participant on the third day after study injection and 3) steatohepatitis (fatty liver) diagnosed after extensive evaluation to identify the cause of a persistent grade 1 ALT (alanine aminotransferase) elevation that was noted starting 25 days after the study vaccination in a clinically asymptomatic participant. A hepatology consultant reported an impression that the condition likely existed prior to study enrollment. Contributing factors to the persistent grade 1 ALT may be alcohol consumption and recent weight gain. A diagnosis of steatohepatitis is overall considered to be a grade 2 condition, but the liver function tests remained at grade 1 severity for about 5 months and then was within normal range at last study visit; a repeat ultrasound showed fatty liver was still present.

Although more reactogenicity has been observed with the 10^{11} PU dose, it appears to be a well-tolerated dose and analgesic/antipyretic nonprescription medications may be self administered for relief of the short-term symptoms. A protocol-specified interim immunogenicity analysis is in progress to compare the placebo and three dosage groups. The blinded immunogenicity data suggest a dose effect with increasing immune response at higher doses. The number of participants with vaccine-induced ELISA at study week 12 by commercial HIV-antibody assay increases from 3 in the 10^{9} PU group, to 6 in the 10^{10} PU group, and to 9 in the 10^{11} PU group among the 12 participants (two placebo and ten vaccine recipients). Preliminary immunogenicity data from VRC 006 suggests the majority of vaccinees develop both CD4+ and CD8+ Env, Gag, and Pol specific T cell responses.

6.2.2 Protocol HVTN 054

HVTN 054 is the second Phase I study of the rAd vaccine, VRC-HIVADV014, as a single agent in uninfected adult participants. This randomized, placebo-controlled, dose escalation study was submitted to BB-IND 11661 in December 2004 and opened accrual in April 2005. It is designed to enroll two groups of 24 participants with low Ad5 neutralizing antibody titer (<1:12) that will be randomized to rAd or placebo in a 5:1 ratio. The first group of vaccinees will receive 10^{10} PU rAd and the second group will receive 10^{11} PU rAd.

6.3 Prime-boost trials

6.3.1 Protocol HVTN 057

HVTN 057 (BB-IND 11894) is the first Phase I study to administer the adenoviral vector vaccine, VRC-HIVADV014-00-VP, as a booster vaccination. In this blinded Phase I study a single boost at 10^{10} PU (or a placebo) is administered to participants who completed the injection regimen with VRC-HIVDNA009-00-VP or placebo in HVTN 052. The rAd boost is given at an interval of 6-9 months after the participant's first injection in HVTN 052. The first participant was enrolled on November 22, 2004 and on April 20, 2005 the last enrollment and study injection was completed. As of May 13, 2005 the still blinded reactogenicity results indicated local pain and/or tenderness reported by 55 (78.6%) participants, with maximal severity moderate,

reported for 3 (4.2%) participants. Erythema and/or induration no greater than 25 cm^2 was reported for 10 (14.3%) participants, of whom 7 (10.0%) reported the erythema and/or induration as <10 cm^2. Systemic reactogenicity did not exceed moderate: 18 (25.7%) reported mild symptoms and 6 (8.5%) reported moderate symptoms. The most commonly reported mild symptoms were malaise and/or fatigue in 20 (28.5%) participants, myalgia in 15 (21.4%) participants, and headache in 10 (14.2%) participants. The most commonly reported moderate symptoms were headache in 7 (10.0%) participants, malaise and/or fatigue in 6 (8.5%) participants, and myalgia in 5 (7.5%) participants. Six (8.5%) participants reported grade 1 fever, none of which exceeded 38.5 ^{o}C.

6.3.2 Protocol VRC 009

VRC 009 is the second Phase I study of rAd as a booster vaccine. It is an open label study designed to enroll subjects who completed three vaccinations with 4 mg or 8 mg of VRC-HIVDNA009-00-VP in VRC 004 (03-I-0022) to receive a 10^{10} PU rAd booster vaccination. The first enrollment into this study occurred January 28, 2005 and as of May 5, 2005 eight subjects were enrolled; four from the 4 mg group and four from the 8 mg group in VRC 004. The mean boost interval to date is 91 weeks [range 79-104 weeks] from first DNA prime vaccination. There have been no serious adverse events. All 8 subjects had mild pain at the injection site and 5 of 8 subjects had at least one mild or moderate symptom (malaise, myalgia, headache or chills). Seven of the 8 subjects have reached the first HIV ELISA testing timepoint and all have shown a vaccine-induced antibody by the commercial ELISA test method.

6.3.3 Protocol VRC 008

VRC 008 is a Phase I randomized study to examine safety and tolerability and immune response to a prime-boost vaccination schedule. The schedule includes three doses of the 6-plasmid multiclade DNA-HIV vaccine, VRC-HIVDNA016-00-VP, at weeks 0, 4 and 8, followed by one dose of the multiclade HIV adenoviral vector vaccine, VRC-HIVADV014-00-VP, booster at week 24. Forty participants, half with high (>1:500) and half with low (≤1:500) adenovirus serotype 5 neutralizing antibody titers, will be randomized in a 1:1 ratio to receive the DNA vaccinations by either needle and syringe or by Biojector and also randomized in a 1:1 ratio to receive the booster vaccination with either 10^{10} PU or 10^{11} PU of the adenoviral vector vaccine. The booster dose is blinded, but there are no placebo injections. This study opened to accrual in May 2005.

6.3.4 Protocol VRC 010

VRC 008 and VRC 010 (for VRC 007 rollover subjects) together (BB-IND 12326) will provide the Phase I safety and immunogenicity data for the prime-boost regimen that uses the 6-plasmid DNA vaccine, VRC-HIVDNA016-00-VP for the priming vaccinations and VRC-HIVADV014-00-VP for the booster vaccination. The first rAd booster injection (10^{10} PU) of a subject primed with the 6-plasmid DNA vaccine occurred on May 4, 2005 when the first subject was enrolled into VRC 010.

7.0 STUDY OBJECTIVES

7.1 Primary Objectives

- Evaluate the safety and tolerability of VRC HIV-1 recombinant adenovirus-5 vector (rAd5) vaccine at either 10^{10} particle units (PU) or 10^{11} PU in HIV-1 uninfected adults.
- Evaluate the safety and tolerability of three VRC HIV-1 DNA-six-plasmid vaccine doses at 4.0 mg/dose in HIV-1 uninfected adults.

- Evaluate the safety and tolerability of three VRC HIV-1 DNA-six-plasmid vaccine doses at 4.0 mg/dose boosted with VRC HIV-1 rAd5 vaccine at either 10^{10} PU or 10^{11} PU in HIV-1 uninfected adults.

7.2 Secondary Objectives

- Evaluate the immunogenicity of rAd5 alone or DNA + rAd5 vs. placebo
- Evaluate the frequency and durability of cellular immune response.
- Characterize the magnitude and breadth of the vaccine-induced HIV specific T cell response as measured by ELISPOT and ICS assays.

8.0 STUDY ENDPOINTS AND STUDY DESIGN

8.1 Study Endpoints

8.1.1 Primary Endpoints

Safety and tolerability will be evaluated by monitoring participants for local and systemic adverse reactions after each injection and for 12 months after the first injection.
The following parameters will be assessed:

- Local reactogenicity signs and symptoms
- Systemic reactogenicity signs and symptoms
- Laboratory measures of safety
- Adverse and serious adverse experiences

8.1.2 Secondary Endpoints

- The proportion of volunteers who have HIV-1 specific T- cell responses quantified by Intracellular cytokine staining (ICS; both CD4+ and CD8+) and ELISPOT and magnitude of the responses.
- The proportion of volunteers with HIV-1 specific antibodies and magnitude of the response
- Proportion of volunteers with increase in antibodies to rAd5
- Impact of pre-existing immunity to rAd5 on immunogenicity

8.1.3 Other endpoints

Proportion of volunteers who test “false positive” on standard HIV testing algorithm

8.2 Study Design

This study is a randomized, placebo-controlled, double-blind with respect to vaccine or placebo assignment. Eligible volunteers will be randomized as follows:

Total 64

Groups	Vaccine	N*	M 0	M 1	M 2	M 6
A	rAd5 10^{10}	12/4	rAd5/P			
B	rAd5 10^{11}	12/4	rAd5/P			
C	DNA+rAd5$10^{10}$	12/4	DNA/P	DNA/P	DNA/P	rAd5/P
D	DNA+rAd5$10^{11}$	12/4	DNA/P	DNA/P	DNA/P	rAd5/P

N* =Vaccine/placebo, M=Month, P= placebo

Safety and tolerability evaluations will be performed at baseline, injection + 30 minutes, Days 3 and14 after each vaccination, and at months 3 (only for rAd5 only arm), 6, 9 and 12.

In DNA+rAd5 arms immune responses will be evaluated at baseline and at 2 and 4 weeks after the third dose of DNA vaccination, (months 2.5 and 3.0), at month 6 prior to the rAd5 boost and at 2, 4 and 6 weeks after rAd5 vaccination (at months 7.0 and 7.5) and at months 9 and 12. Similarly, in rAd5 only arms, immune responses will be evaluated at baseline and at 4 and 6 weeks after rAd5 vaccination (months 1 and 1.5), and at months 9 and 12 post vaccination.

Samples collected at selected time points described below will be analyzed by IAVI Core laboratories

- PBMC and serum collected at base line
- PBMC and serum from 4 weeks after 3rd vaccination with DNA
- PBMC and serum from 6 weeks after rAd5 10^{10} and 10^{11} in all groups
- PBMC and serum from M 9 and M12

Specimens from the following time points will be analyzed by VRC lab.

- PBMC and serum collected at base line
- PBMC and serum from 2 weeks after 3rd vaccination with DNA
- PBMC and serum from 4 weeks after rAd5 10^{10} and 10^{11} in all groups
- PBMC and serum from M6 and M12

8.2.1 Duration of the Study

Volunteers will be screened up to 6 weeks before vaccination and will be followed for 12 months after the first study vaccination. It is anticipated that it will take approximately 6 months to enroll the study. The total duration of the study will be approximately 18 months.

8.2.2 Study Population

The study population consists of healthy male or female adults aged 18-50 years who are willing to undergo HIV testing and who, in the opinion of the investigator or designee, understand the

study and can provide written informed consent. Female volunteers must be willing to use an effective method of contraception for the duration of their participation in the study.

Principle exclusion criteria include confirmed HIV-1 or HIV-2 infection; reported high-risk behavior for HIV infection; pregnancy and lactation; chronic disease; clinically significant abnormal laboratory values; recent vaccination(s) or receipt of blood product or investigational product, vaccine or other investigational products; and previous severe local or systemic reactogenicity to vaccination or history of severe allergic reactions.

Approximately 64 volunteers (48 Vaccine recipients, 16 placebo recipients), at least 30% females, who meet all eligibility criteria will be included in the study. An over-enrollment of up to 10% (approximately 6 additional volunteers) will be accepted in the study.

8.2.3 Inclusion Criteria

Healthy males and females, as assessed by a medical history, physical exam, and laboratory tests;

1. At least 18 years of age on the day of screening and no greater than 50 years (not yet reached 51st birthday) on the day of first study vaccination;
2. Willing to comply with the requirements of the protocol and available for follow-up for the planned duration of the study ;
3. In the opinion of the Principal Investigator or designee, understand the information provided. Written informed consent must be given before any study-related procedures are performed;
4. Willing to undergo HIV Testing, HIV counseling and receive HIV Test results;
5. If sexually active female of child bearing potential (not menopausal or anatomically sterile), using or agree to use an effective method of contraception (hormonal contraceptive; diaphragm; Intra Uterine Device (IUD); condoms; anatomical sterility in self or partner) for the duration of their participation in the study. All female volunteers must be willing to undergo urine pregnancy tests at time points as indicated in the Schedule of Procedures (Appendices A and B)
6. Laboratory parameters as follows:
   - Hemoglobin within the institutional normal range and greater than 11.0 g/dL for women and 12.5 g/dL for men.
   - White blood cell count (WBC): 3,300 – 12,000 cells/mm^3 (in the absence of clinical or pathological etiology).
   - Total lymphocyte count greater than 800 cells/mm^3
   - Absolute neutrophil count within the institutional normal range and greater than 1000 cells/mm^3
   - Platelets = 125,000 – 550,000 cells/mm^3.
   - ALT (SGPT) ≤ 1.25 x ULN.
   - Serum creatinine ≤ ULN.
   - Normal urinalysis defined as dipstick with negative glucose, negative or trace protein, and negative or trace hemoglobin (blood), unless values outside of this range is determined to be clinically insignificant by the site principal investigator

8.2.4 Exclusion Criteria

1. Confirmed HIV-1 or HIV-2 infection

2. Reported high-risk behavior for HIV infection defined as:
   Within 6 months before vaccination, the volunteer has:
   - Had unprotected vaginal or anal sex with a known HIV infected person or a casual partner (i.e. no continuing established relationship).
   - Engaged in sex work for money or drugs.
   - Substance abuse/use injection drugs.
   - Acquired a sexually transmitted disease (STD) (e.g. gonorrhea, chlamydia, syphilis, trichomonas vaginalis, and symptomatic herpes genitalis).
   - Having a high-risk partner either currently or within the previous 6 months.

3. Any clinically significant abnormality on history or examination including history of immunodeficiency or autoimmune disease; use of systemic corticosteroids, immunosuppressive, antiviral, anticancer, or other medications considered significant by the investigator within the previous 6 months;

4. Any clinically significant acute or chronic medical condition that is considered progressive or in the opinion of the investigator would make the volunteer unsuitable for the study.

5. Confirmed diagnosis of hepatitis B (surface antigen, HbsAg); or active or untreated syphilis (documented by exam or serology unless positive serology is due to remote treated infection, or positive rapid plasma regain/venereal disease research laboratory (RPR/VDRL) test is not associated with positive Treponemal specific serology).

6. If female, pregnant or planning a pregnancy at any time throughout the duration their participation in the trial; or lactating.

7. Receipt of live attenuated vaccine within the previous 30 days or planned receipt within 60 days after vaccination with investigational product or receipt of other medically indicated subunit or killed vaccine within the previous 14 days or planned receipt within 14 days after vaccination with investigational product;

8. Receipt of blood transfusion or blood products within the previous 120 days or immunoglobulin within the previous 60 days;

9. Participation in another clinical study of an investigational product currently, within the previous 3 months or expected participation during this study;

10. Receipt of another investigational HIV vaccine at any time;

11. History of severe local or systemic reactogenicity to vaccines or history of severe allergic reactions;

12. Major psychiatric illness including any history of schizophrenia or severe psychosis, bipolar disorder requiring therapy, suicide attempt or ideation in the previous 3 years;

13. Hypertension that is not well controlled by medication or blood pressure ≥ 150/100 (either or both values).

8.2.5 Recruitment of Volunteers

Healthy adult male and female (at least 30 %) volunteers may be recruited through information presented in community organizations, hospitals, colleges, other institutions and/or advertisements to the general public. This information will contain contact details.

If other recruitment strategies are used, the sponsor needs to be informed during the recruitment process it is important to ensure full counseling and full informed consent.

9.0 STUDY VISITS

9.1 Screening Visit

During Screening, site personnel will perform the following procedures:

- Provide and/or review the Informed Consent Document and answer any questions about the study prior to obtaining written informed consent.
- Obtain written informed consent prior to conducting any study procedures.

If the volunteer agrees to participate, site personnel will:

- Provide a screening questionnaire to the volunteer for completion (if questionnaire is used)
- Perform an HIV risk assessment.
- Perform a complete medical history (including concomitant medication)
- Perform a general physical examination including height, weight, vital signs (pulse, respiratory rate, blood pressure and temperature), examination of skin, respiratory, cardiovascular systems and abdomen, and an assessment of cervical and axillary lymph nodes etc.
- Conduct Pre- HIV test counseling
- Collect blood and urine specimens for all tests as indicated in the Schedule of Procedures (Appendices A and B). Perform a pregnancy test for all female volunteers.
- Post-HIV Test Counseling will be performed as the results become available or at the vaccination visit.

Screening laboratory test(s) may be repeated once at the discretion of the principal investigator or designee to investigate any isolated abnormalities.

If the screening visit occurs more than 42 days prior to the date of vaccination, all screening procedures must be repeated. The complete medical history may be replaced by an interim medical history and the Volunteer Information Sheet should be reviewed.

If a volunteer has signed the informed consent form but does not meet the eligibility criteria, the reason for exclusion should be entered into the log. All signed informed consent forms must be kept in a secured area.

9.2 Vaccination Visit

Prior to the first vaccination, site personnel will:

- Answer any questions about the study
- Review interim medical history (including concomitant medications)
- Review screening safety laboratory data.
- Review the Informed Consent Document with volunteers.
- Perform a directed physical examination including vital signs (pulse, respiratory rate, blood pressure and temperature), an assessment of cervical and axillary lymph nodes and any further examination indicated by history or observation).
- Conduct pre HIV-test counseling.
- Collect blood and urine specimens for all tests as indicated in the Schedule of Procedures (Appendices A and B). Perform a pregnancy test for all female volunteers and obtain results prior to vaccination.
- Baseline assessment of the site of vaccination and any systemic symptoms

The volunteer will be assigned an allocation number according to the instructions specified in the Study Operations Manual.

The Investigational Product will be administered as specified in Section 11.3 Administration.

Site personnel will closely observe volunteers for at least 30 minutes after vaccination for any acute reactogenicity. At the end of the observation period site personnel will:

- Record vital signs (pulse, respiratory rate, blood pressure and temperature)
- Assess any local and systemic reactogenicity
- Assess any other adverse events.

For subsequent vaccination visits, site personnel will perform the same procedures as above with the following exceptions:

- Review the routine safety laboratory parameters from the previous visit prior to each vaccination. If a volunteer has an abnormal laboratory value that is known at the time of vaccination, follow the specified guidelines (see section 13.4)
- Conduct pre HIV-test counseling if an HIV Test is required (see Appendices A and B) and/or provide post-test counseling if the results of a prior HIV test are being provided to the volunteer.

9.3 Post-Vaccination Visits

The volunteer will be provided a memory card to record any reactogenicity events occurred within 3 days after first vaccination. The volunteer will be asked to return to the clinic on Days 3 and 14 after each vaccination. The study personnel will review the memory card with the volunteer and record the information in the clinic chart.

The following procedures will be conducted at these visits:

- Review of interim medical history and use of concomitant medications.

- If symptoms are present, perform a symptom-directed physical examination.
- Assess local and systemic reactogenicity as well as any other adverse events.
- Collection of blood and urine specimens for all tests as indicated in the Schedule of Procedures (Appendices A and B).

9.4 Additional Follow-up Visits

Assessments and procedures will be performed according to the Schedule of Procedures (Appendices A and B). Post HIV-test counseling will be performed as the HIV-test results become available.

In case of adverse event(s), the volunteer will be assessed and followed up by the clinical team. Supplemental visit(s) for further investigation can be planned at the discretion of the principal investigator or designee. Supplemental visit(s) may be recommended if clinically indicated or to clarify observations.

9.5 Unscheduled Visits/Contact

Unscheduled visits/contacts are visits/contacts which are not described in the Schedule of Procedures (Appendices A and B). They may be performed at any time during the study.

Unscheduled visits may occur:

- For administrative reasons, for example, the volunteer may have questions for study staff or may need to re-schedule a follow-up visit.
- To obtain laboratory test results from a previous visit.
- In the event that a volunteer presents to the study site after having missed a scheduled study visit outside a scheduled visit window.
- For other reasons as requested by the volunteer or site investigator.

All unscheduled visits will be documented in the volunteers' study records and on applicable source documents.

9.6 Final Visit/Early Termination Visit

Assessments and procedures will be performed according to the Schedule of Procedures (Appendices A and B).

Site personnel will:

- Review any adverse events and concomitant medications
- Perform a general physical examination including weight, vital signs (pulse, respiratory rate, blood pressure and temperature), examination of skin, respiratory, cardiovascular and abdominal systems, and an assessment of cervical and axillary lymph nodes.
- Assess any local and systemic reactogenicity
- Collect blood and urine specimens for tests as specified in the Schedule of Procedures (Appendices A and B).

10.0 STUDY PROCEDURES

10.1 Protocol Registration

Prior to implementation of this protocol, the protocol and consent form must be approved by the applicable institutional review board/ethics committee (IRB/EC) and must be submitted to the institutional biosafety committee (IBC). The protocol and subsequent protocol amendment(s) must be registered and approved by the DAIDS RCC Protocol Registration Office. The approval letter from the IBC must be submitted to the RCC at the time of the initial protocol registration. Protocol registration must occur before any subjects can be enrolled in this study.

10.2 Informed Consent Process

A sample informed consent document (Appendix D) is provided by the sponsor to the site. An informed consent document will be made site-specific, submitted and approved by the IEC/ERB before it can be used at the site.

Volunteer Information Sheet
A qualified member of the site personnel will obtain informed consent by reviewing the Volunteer Information Sheet.

The following study specific elements are included:

1. That it is unknown whether or not the vaccine(s) will protect against HIV infection or disease

2. That it may be possible that the vaccinated volunteer will develop antibodies against HIV following vaccination, which may produce a positive result in a routine HIV Antibody Test, and that provisions have been made to distinguish between response to vaccine and HIV infection during and after the study. In case the volunteer has a positive result in a routine HIV Antibody Test, he/she will be followed until the result is no longer positive

3. That the sexually active volunteer should use a reliable form of contraception from screening until completion of the study.

4. That placebo will be administered in this study and volunteer may receive placebo throughout the study.

Informed Consent Form
All volunteers will give their written informed consent to participate in the study on the basis of appropriate information and with adequate time to consider this information and ask questions.

The volunteer's consent to participate must be obtained by him/her signing or marking, and dating the informed consent form witnessed by a member of the study team. The members of the site personnel who are involved in conducting the informed consent discussions must also sign and date the Informed Consent Form. However, in all cases a physician must be involved in the discussions and should also sign the Informed Consent Form. The time the Informed Consent Form has been signed should be recorded.

If the volunteer is functionally illiterate, the consent document must be read to them in the language that they best understand in the presence of an independent literate observer not affiliated with the study, who will also sign and date the consent form as an independent witness.

The signed/marked and dated informed consent document must remain at the study site. A copy of the signed and dated informed consent form will be offered to the volunteer to take home if the volunteer is willing to receive the consent form. Those volunteers who do not wish to take a copy will be required to document that they declined to do so.

Family members, sexual partner(s) or spouse(s) will be offered education and counseling regarding a volunteer's participation in the study ONLY with the written consent of the participating volunteer.

The study site is responsible for developing study informed consent form for local use, based on the template provided in Appendix D of this protocol, which describes the purpose of the study, the procedures to be followed, and the risks and benefits of participation, in accordance with all applicable regulations. The study site also is responsible for translating the consent form into local languages and verifying the accuracy of the translation by performing an independent back-translation.

10.3 Medical History and Physical Examination

At screening, a comprehensive medical history will be collected including details of any previous vaccinations and reaction to vaccinations, history of sexually transmitted diseases, contraceptive practices, and history of epilepsy.

A general physical examination includes the following: weight, height, vital signs, and examination of skin, respiratory, cardio-vascular, central nervous and abdominal systems as well as an assessment of cervical and axillary lymph nodes.

At each study visit, an interim medical history and symptom directed physical examination will be performed. A directed physical examination will include vital signs, examination of vaccination site and any further examination indicated by history or observation

10.4 HIV Risk Assessment, HIV Testing and HIV-test Counseling

Site personnel will assess volunteers for past and current risk of HIV infection.

Additionally, site personnel will perform pre-HIV test counselling (prior to collecting blood for an HIV test) and post-HIV test counselling (when HIV test results are available) according to the Schedule of Procedures (Appendices A and B). For more information on HIV testing and HIV-test counselling, see Section 14.0.

10.5 Family Planning Counseling

Site personnel will counsel male and female volunteers about the importance of preventing pregnancies and use of condoms as well as other effective family planning methods. Volunteers will be referred to a family planning clinic if a contraceptive prescription is required.

The family planning counseling will be performed at time points according to the Schedule of Procedures (Appendices A and B).

10.6 Blood Collection

Up to 107 mL of blood will be collected at each visit (ranging from 7-107 mL), usually from the antecubital fossa, according to the Schedule of Procedures (Appendices A and B).

All specimens will be handled according to the procedures specified in the Study Operations Manual. All safety tests will be conducted at the site laboratories (KAVI and PSF) with the exception of PCR for confirmatory HIV testing which will be done by CLS Laboratories, South Africa. Immunology testing will be conducted at the site lab (KAVI only), IAVI Core Laboratory and the Vaccine Research Center. Samples will be shipped to external lab according to the shipping SOP. (Refer to the Study Operations Manual for the timepoints of sample shipment to IAVI and VRC labs)

In the event of an abnormal laboratory value, volunteers may be asked to have an additional sample collected at the discretion of the principal investigator or designee.

10.7 Reimbursement

Volunteers will be reimbursed for their time, effort and for costs to cover their travel expenses to the study site and any inconvenience caused due to study participation. Reimbursement will be made after the completion of each study visit. Site specific reimbursement amounts will be documented in the site-specific Volunteer Information Sheet approved by the IRB/EC.

10.8 Randomization and Blinding

Volunteers will be identified by a unique volunteer identification number.

The randomization schedule will be prepared by the statisticians at the Data Coordinating Center (DCC) prior to the start of the study. Volunteers will be assigned a specific allocation number. An unblinding list will be provided to the site where pharmacist only will have access information about individual allocation by the DCC.

This is a double-blind study. Site personnel (investigator and clinical personnel monitoring the safety and laboratory assay results) and volunteers will be blinded with respect to the allocation of Investigational Product (placebo or active vaccine).

Blinding will not apply to the group assignment (A, B or C, D) nor to the assignment of dosage levels (10^{10} or 10^{11} PU for rAd5 dosage).

Volunteers will be informed about their assignment to active vaccine or placebo once the data analysis is completed.

10.9 Unblinding Procedure for Individual Volunteers

Unblinding of an individual volunteer may be indicated in the event of a medical emergency where the clinical management/medical treatment of the volunteer would be altered by knowledge of the group assignment allocation of investigational product.

The unblinded information should be restricted only to a small group of individuals involved in clinical management/medical treatment of the volunteer (e.g. treating physician) and maintain the blind for those responsible for the study assessments.

The reasons for unblinding should be documented and the Data Coordinating Center should be notified. The procedures and contact numbers for unblinding are outlined in the Study Operations Manual.

11.0 INVESTIGATIONAL PRODUCT

The pharmacists should consult the *Pharmacy Guidelines and Instructions for DAIDS Clinical Trials Networks* manual for standard pharmacy operations procedures. The study vaccine regimen is shown in Section 11.1. See Investigator's Brochure for further information about study products.

11.1 Study vaccine regimen

- **rAd5 1 x 10^{10}** PU/ mL: VRC-HIV ADV014-00-VP (HIV-1 recombinant adenovirus-5 vaccine, rAd5 vaccine)
- **rAd5 1 x 10^{11}** PU/ mL: VRC-HIV ADV014-00-VP (HIV-1 recombinant adenovirus vaccine, rAd5 vaccine)
- **rAd5 Placebo**: VRC-DILUENT013-DIL-VP
- **DNA**: VRC-HIVDNA016-00-VP (Multiclade HIV-1 DNA six plasmid vaccine, DNA Vaccine)
- **DNA Placebo**: Phosphate-buffered saline (PBS)

Group	N	Treatment	Priming			Boosting
			Injection Schedule in Months *(Days)*			
			0*(0)*	1*(28)*	2*(56)*	6*(168)*
	12	*T-A*	**rAd5 1 x 10^{10}** PU IM*			
**A**	4	*C-A*	**rAD5 Placebo** 1 mL IM*			
	12	*T-B*	**rAd5 1 x 10^{11}** PU IM*			
**B**	4	*C-B*	**rAd5 Placebo** 1 mL IM*			
	12	*T-C*	**DNA** 4 mg IM**	**DNA** 4 mg IM**	**DNA** 4 mg IM**	**rAD 1 x 10^{10}** PU IM*
**C**	4	*C-C*	**DNA Placebo** 1 mL IM**	**DNA Placebo** 1 mL IM**	**DNA Placebo** 1 mL IM**	**rAD Control** 1 mL IM*
	12	*T-D*	**DNA** 4 mg IM**	**DNA** 4 mg IM**	**DNA** 4 mg IM**	**rAD 1 x 10^{11}** PU IM*
**D**	4	*C-D*	**DNA Placebo** 1 mL IM**	**DNA Placebo** 1 mL IM**	**DNA Placebo** 1 mL IM**	**rAD Control** 1 mL IM*

*Administered with needle and syringe as one 1 mL intramuscular (IM) injection in either deltoid.
**Administered by Biojector® as one 1 mL IM injection in either deltoid.
Day 0 is defined as the day of first injection

Group A

T-A VRC-HIV ADV014-00-VP (rAd5) 1 x 10^{10} PU administered as 1 mL IM in either deltoid at Day 0.

C-A: Placebo for VRC-HIV ADV014-00-VP (rAd5) (labeled as VRC-DILUENT013-DIL-VP) 1 mL IM in either deltoid at Day 0.

Group B

T-B: VRC-HIV ADV014-00-VP (rAd5) 1 x 10^{11} PU administered as 1 mL IM in either deltoid at Day 0.

C-B: Placebo for VRC-HIV ADV014-00-VP (rAd5) (labeled as VRC-DILUENT013-DIL-VP) 1 mL IM in either deltoid at Day 0.

Group C

T-C: VRC-HIVDNA016-00-VP (DNA) 4 mg administered as 1 mL IM (via Biojector®) in either deltoid at Day 0, Day 28 +/- 7, and Day 56 +/- 7.
AND
VRC-HIV ADV014-00-VP (rAd5) 1 x 10^{10} PU administered as 1 mL IM in either deltoid at Day 168 +/- 7.

C-C: Placebo for VRC-HIVDNA016-00-VP (DNA): (phosphate buffered saline, PBS) 1 mL IM (via Biojector®) in either deltoid at Day 0, Day 28+/- 7 and Day 56 +/- 7.
AND
Placebo for VRC-HIV ADV014-00-VP (rAd5) (labeled as VRC-DILUENT013-DIL-VP) 1 mL IM in either deltoid at Day 168 +/- 7.

Group D

T-D: VRC-HIVDNA016-00-VP (DNA) 4 mg administered as 1 mL IM (via Biojector®) in either deltoid at Day 0, Day 28 +/- 7 and Day 56 +/- 7.
AND
VRC-HIV ADV014-00-VP (rAd5) 1 x 10^{11} PU administered as 1 mL IM in either deltoid at Day 168 +/- 7.

C-D: Placebo for VRC-HIVDNA016-00-VP (DNA): (phosphate buffered saline, PBS) 1 mL IM (via Biojector®) in either deltoid at Day 0, Day 28 +/- 7 and Day 56 +/- 7.
AND
Placebo for VRC-HIV ADV014-00-VP (rAd4) (labeled as VRC-DILUENT013-DIL-VP) 1 mL IM in either deltoid at Day 168 +/- 7.

11.2 Study product formulation and preparation

See the Investigator's Brochure for further information about study products.

11.2.1 VRC-HIV ADV014-00-VP 1 x 10^{10} PU (multiclade HIV-1 recombinant adenoviral vector vaccine, rAd5)

VRC-HIVADV014-00-VP is manufactured by GenVec Incorporated at a contract manufacturer, Molecular Medicine. The vaccine is supplied as a 1 x 10^{10} PU/mL solution in a 3 mL sterile glass vial containing 1.3 mL of a clear, colorless, sterile, isotonic solution. Although the vial label notes a storage temperature of -10º C to -25º C, the product may be stored at temperatures as low as -30º C. Once thawed, vials should not be frozen or reused.

To prepare, remove 1 vial of VRC-HIVADV014-00-VP 1 x 10^{10} PU/mL from the freezer and allow to equilibrate to room temperature. Using aseptic technique, withdraw 1 mL of the study product into a syringe. Each syringe should be labeled as directed in the V001 Study Operations Manual. The study product must be administered within 4 hours of removal from the freezer.

Any unused portion of entered vials and expired pre-filled syringes should be disposed of in a biohazard container and incinerated or autoclaved.

11.2.2 VRC-HIV ADV014-00-VP 1 x 10^{11} PU (multiclade HIV-1 recombinant adenoviral vector vaccine, rAd5)

VRC-HIVADV014-00-VP is manufactured by GenVec Incorporated at a contract manufacturer, Molecular Medicine. The vaccine is supplied as a 1 x 10^{11} PU/mL solution in a 3 mL sterile glass vial containing 1.3 mL of a clear, colorless, sterile, isotonic solution. Although the vial label notes a storage temperature of -10º C to -25º C, the product may be stored at temperatures as low as -30º C. Once thawed, vials should not be frozen or reused.

To prepare, remove 1 vial of VRC-HIVADV014-00-VP 1 x 10^{11} PU/mL from the freezer and allow to equilibrate to room temperature. Using aseptic technique, withdraw 1 mL of the study product into a syringe. Each syringe should be labeled as directed in the V001 Study Operations Manual. The study product must be administered within 4 hours of removal from the freezer.

Any unused portion of entered vials and expired pre-filled syringes should be disposed of in a biohazard container and incinerated or autoclaved.

11.2.3 VRC-DILUENT013-DIL-VP (Final Formulation Buffer, FFB, Placebo for rAd5)

Final Formulation Buffer (FFB) provided by Gen Vec Incorporated for use as the placebo. It is composed of sodium chloride, Tris buffer, trehalose ●2H20 (low endotoxin), magnesium chloride ●6H20, monoleate (Tween 80) and water for injection (WFI). It is provided as a 1.2 mL clear, colorless, isotonic solution with a pH of 7.2. It should be refrigerated at 2-8 º C.

To prepare, remove 1 vial of VRC-DILENT013-DIL-VP (placebo control, FFB) from the refrigerator and allow to equilibrate to room temperature. Using aseptic technique, withdraw 1 mL of the study agent into a syringe. Each syringe should be labeled as directed in the V001 Study Operations Manual. The study product must be administered within 4 hours of removal from the refrigerator.

Any unused portion of entered vials and expired pre-filled syringes should be disposed of in a biohazard container and incinerated or autoclaved.

11.2.4 VRC-HIVDNA016-00-VP (multiclade HIV-1 DNA six plasmid vaccine)

VRC-HIVDNA016-00-VP is manufactured by Vical Incorporated (San Diego, CA). The product is formulated in phosphate buffered saline (PBS), pH 7.2. The vaccine is provided as a 4 mg/mL solution in 2 mL single use glass vials containing 1.3 mL of a clear, colorless, sterile, isotonic solution. The product must be stored frozen at -10º C to -70º C. Once thawed, vials should not be re-frozen after thawing.

To prepare, remove 1 vial of DNA 4 mg/mL from the freezer and allow to equilibrate to room temperature. Swirl the contents gently. Using aseptic technique, aseptically withdraw 1 mL of

the DNA (4 mg/mL) from the vial into the Biojector® 2000 syringe and cap the syringe. Each syringe should be labeled as directed in the V001 Study Operations Manual. The injection should be given as soon as possible after preparation.

Any unused portion of entered vials and expired pre-filled syringes should be disposed of in a biohazard container and incinerated or autoclaved.

11.2.5 Placebo for DNA vaccine (phosphate buffered saline, PBS)

Phosphate-buffered saline (PBS) is manufactured by Bell-Moore Labs for use as the placebo for DNA vaccine. It is a clear, colorless, isotonic solution with a PH of 7.2. It is provided in 3 mL vials containing 2.4 mL of PBS. The product must be stored at controlled room temperature (20 to 25º C with excursions permitted between 15 and 30º C).

To prepare, remove 1 vial of Placebo (PBS) and swirl the contents gently. Using aseptic technique, aseptically withdraw 1 mL of the PBS from the vial into the Biojector® 2000 syringe and cap the syringe. Each syringe should be labeled as directed in the V001 Study Operations Manual. The injection should be given as soon as possible after preparation.

Any unused portion of entered vials and expired pre-filled syringes should be disposed of in a biohazard container and incinerated or autoclaved.

11.2.6 Labeling procedures to preserve blinding

The pharmacist will prepare all doses for administration and dispense to the clinic. In order to preserve blinding, the pharmacist will place a yellow overlay on the syringe containing rAd vaccine (VRC-HIV ADV014-00-VP 1 x 10^{10} PU and VRC-HIV ADV014-00-VP 1 x 10^{11}) or rAd5 placebo.

11.3 Procedures to preserve blinding

The study statistician will provide randomization lists to the Pharmacist of Record at each site. Site pharmacists will be responsible for maintaining randomization materials in a confidential and secure manner. Additionally, study assignments for each participant will be contained in individual sealed envelopes labeled only with the randomization number. *These envelopes will be securely stored by the site pharmacist in a locked file cabinet in a limited access area to be available in case of emergency. Only the site pharmacist will have a key to the locked file cabinet.*

Participants will be randomized to receive either the investigational vaccine or placebo. The participant, the clinical staff, and the Principal Investigator will be blinded to treatment allocation *of study product (vaccine or placebo).* The pharmacist with primary responsibility for drug dispensing maintains the randomization code and completes assignments of participants according to the randomization allocation.

The study pharmacists are responsible for preparing a Biojector® 2000 syringe with the DNA vaccine dose or DNA placebo, or the regular syringe with needle for the rAd5 vaccine or rAd5 placebo indicated by the participant’s randomization assignment and labeling with the participant identification.

The blind will be broken only if, in the opinion of the Principal Investigator, immediate unblinding is necessitated by an acute safety concern. The individual assignment envelopes will be used for this purpose. Unblinding of participants may occur only after all participants have had their last study visit. Blinding will be limited to whether the participant receives the test vaccine or

placebo; however, researchers and participants will know to what treatment group the participant is assigned.

11.4 Study Product administration

All injections should be administered in either deltoid. It is not necessary to use the same deltoid at each visit. *The preferred site of first administration is the deltoid muscle of the non-dominant upper arm.*

A 1 mL injection of DNA vaccine or placebo will be administered IM using the Biojector® 2000. A syringe with a 21 gauge needle will be used to administer rAD vaccine or rAD control.

The study vaccine will be prepared by the study pharmacist and administered by the clinic nursing staff.

11.4.1 VRC-HIVADV014-00-VP (rAd5 vector) or Placebo(labeled as VRC-DILUENT013-DIL-VP)

A 1 mL injection of VRC-HIVADV014-00-VP(rAd5)1 x 10^{10} PU , or VRC-HIVADV014-00-VP(rAd5) 1 x 10^{11} PU or Placebo (FFB) will be administered into either deltoid muscle using a syringe with a 21 gauge needle with a length of 1 or 1-1/2 inches (depending on the subject's arm size).

When preparing a dose in a syringe and administering the dose, consideration should be given to the volume of solution that may remain in the needle after the dose is administered. The pharmacy and clinic staffs are encouraged to work together to administer the dose specified in the protocol.

11.4.2 VRC-HIVDNA016-00-VP (DNA) Vaccine or Placebo (PBS)

A 1 mL injection of VRC-HIVDNA016-00-VP (DNA) Vaccine or Placebo (PBS) will be administered IM using the Biojector® 2000.

For the Biojector® 2000 Needle-Free Injection Management System, the specifications below should be observed.

The Biojector® 2000 Needle-Free Injection Management System

- This system will be used as directed by the company
- Neither the material being injected nor injection site skin preparation requires deviation from standard procedures
- The injection site is disinfected and the area allowed to dry completely
- The skin around the injection site is held firmly while the syringe is placed against the injection site at a 90º angle
- The actuator is pressed and the material is released into the muscle
- Continue to hold firmly for 3 seconds
- After the injection, the site is covered with a sterile covering and pressure applied with 3 fingers for 1 minute.

Biojector® 2000 utilizes sterile, single-use syringes for variable dose, up to 1.0 mL, medication administration. The study agent is delivered under pressure by a compressed CO_2 gas cartridge that is stored inside the Biojector® 2000. When the Biojector® 2000's actuator is depressed, CO_2 is released, causing the plunger to push the study agent out of the sterile syringe through the skin and into the underlying tissue. The study agent is expelled through a micro-orifice at high velocity in a fraction of a second to pierce the skin. The CO_2 does not come

in contact with the injectate and the syringe design prevents any back splatter or contamination of the device by tissue from the participant.

Testing of the Biojector® 2000 for administration of vaccines has demonstrated effective immune responses and although its use is associated with some self-limited pain, redness or swelling at the injection site, this method of administration is well tolerated, and offers the advantage of eliminating needle stick accidents in the clinic.

This system has U.S. Food and Drug Administration (FDA) clearance for delivering intramuscular injections of vaccine.

11.5 Study Product Aquisition

Study product will be provided by the Vaccine research Center (VRC), National Institute of Allergy and Infectious Diseases. The Biojector 2000® and the Biojector 2000® syringes will be purchased from Bioject Inc. Study pharmacists can obtain study products (and Biojector 2000® syringes) from the DAIDS Clinical Research Products Management Center (CRPMC) by following the ordering procedures given in the *Pharmacy Guidelines and Instructions for DAIDS Clinical Trials Networks* manual.

11.6 Pharmacy Records

The pharmacist is required to maintain complete records of all study products received from the CRPMC and subsequently dispensed. The procedures are included in the sections on Study Product Control and Study Product Dispensing in the *Pharmacy Guidelines and Instructions for DAIDS Clinical Trials Networks* manual.

11.7 Study Product Accountability

11.7.1 Documentation

The study pharmacist will be responsible for maintaining an accurate record of the randomization codes, inventory, and an accountability record of vaccine supplies for this study. The study pharmacist will also be responsible for insuring the security of these documents.

11.7.2 Disposition

After the study is completed or terminated, the site will receive instruction regarding the final disposition of any remaining study products. Partially used vials will not be administered together participants or used for *in vitro* experimental studies.

All unused study products must be returned to the CRPMC after the study is completed or terminated unless otherwise instructed by the CRPMC.

12.0 ASSESSMENTS

12.1 Safety Assessments

Data on local and systemic reactogenicity will be collected by structured interview, using specific questions. Data on other adverse events will be collected with open-ended questions. All data will be recorded on the appropriate source documents.

12.1.1 Local reactogenicity

The presence of local reactogenicity will be assessed at the time points specified in the Schedule of Procedures (Appendices A and B).

Local reactogenicity (pain, tenderness, erythema) will be assessed and graded using Appendix C, DAIDS AE Grading Toxicity Table, as a guideline.

12.1.2 Systemic reactogenicity

The presence of systemic reactogenicity will be assessed at the time points specified in the Schedule of Procedures (Appendices A and B).

Vital signs (pulse, respiratory rate, blood pressure and temperature) will be measured by site personnel prior to vaccination and at least 30 minutes post-vaccination.

Systemic reactogenicity will be assessed and graded using Appendix C, DAIDS AE Grading Toxicity Table as a guideline.

12.1.3 Other adverse events

Occurrence of other adverse events (including Serious Adverse Events) will be collected following an open-ended question to volunteers on the time points according to the Schedule of Procedures (Appendices A and B). The adverse events will be graded using Appendix C DAIDS AE Gading Toxicity Table as a guideline.

For more information regarding adverse events refer to Section 13.0, Adverse Events.

12.1.4 Concomitant Medications

During the study, information regarding concomitant medications and reasons for their use will be solicited from the study volunteers at each visit and recorded.

Concomitant receipt of investigational products, including other HIV vaccines is prohibited during the study.

If clinically indicated, non-live vaccines (non-HIV) may be given up to 14 days before study vaccination(s) or after post-vaccination blood draw (i.e. 2 weeks after study vaccinations).

Live-attenuated vaccines (non-HIV) may be given 60 days before study vaccination(s) or after the post-vaccination blood draw. However, the study vaccination(s) should not be given if there are any continuing symptoms from recently administered non-HIV vaccines. In this situation, the principal investigator should consult with the IAVI Medical Monitor before administering the next study vaccination.

12.1.5 Routine laboratory parameters

The table below shows the laboratory parameters that will be measured routinely. These will include hematology, clinical chemistry, immunological assays and urinalysis. The samples for these tests will be collected at the time points indicated in the Schedule of Procedures (Appendices A and B).

Laboratory Parameters

Laboratory Parameter	Test
Hematology	Full blood count (hemoglobin, erythrocytes, leucocytes platelets)<br>Differential count, absolute neutrophil count, absolute lymphocyte count, Monocytes, Granulocytes, Eosinophils),
Clinical Chemistry	Liver function test: alanine transferase (ALT)<br>Creatinine
Immunology	CD4 and CD8 T cells (percentage and absolute count)
Urinalysis	Dipstick test for protein, blood glucose, ketones, esterase (leukocytes), nitrite.<br>If abnormalities (blood, protein, leucocytes) are found on dipstick test then further test will be performed (e.g. microscopy, culture)

12.1.6 Specific screening tests:

Volunteers will be screened to exclude the following diseases:

- Hepatitis B: positive for hepatitis B surface antigen (HbsAg)
- Syphilis: confirmed diagnosis of active/untreated syphilis (documented by exam or serology unless positive serology is due to remote treated infection, or positive rapid plasma reagin/venereal disease research laboratory (RPR/VDRL) test is not associated with positive Treponemal specific serology).

12.2 Immunogenicity Assessments

12.2.1 Antibody Responses

- Antibodies against HIV protein will be measured according to time points as indicated for binding and neutralization assays on the Schedule of Procedures (Appendices A and B).

- Serum antibody against Ad5 may be examined as specified in Schedule of Procedures (Appendices A and B)

12.2.2 Cellular Responses

Immunogenicity assays, including ELISPOT and intracellular cytokine staining (ICS) will be used to measure in-vitro T cell responses following stimulation by HIV-specific antigen peptides. This will be conducted by validated assays at various timepoints at the site laboratory (KAVI only), IAVI Core Laboratory and/or VRC lab as indicated in the Schedule of Procedures (Appendices A and B).

Further studies may be carried out to characterize the nature of any observed vaccine induced immune responses. These would include using peptide pools a) to determine the specific epitopes recognized and b) from different HIV-subtypes. Selected T cell responses may be further characterized for HLA restriction, their ability to proliferate (IAVI Core Lab) and additional markers on the responding cells, such as markers for activation or homing to mucosal tissues.

12.2.3 PBMC, Serum and Plasma Storage

Samples of cryopreserved PBMC, plasma and serum will be taken at time points as indicated in the Schedule of Procedures (Appendices A and B) for purposes of standardization, quality control and for future assays related to HIV vaccine research and development. These samples will be archived and only a code will identify the samples.

For the immunogenicity assessments, the laboratory personnel will be trained as necessary by the sponsor and provided with a written procedure manual. The sponsor will also provide specific instructions on reagents.

The samples described above will be shipped routinely from each site to the IAVI Core Laboratory and VRC lab. The sites will keep approximately $2x10^7$ PBMC samples to conduct some assays at the site e.g., ELISPOT

12.3 Other Assessments

12.3.1 Genetic Testing

Samples for HLA typing will be collected at the time point indicated in the Schedule of Procedures (Appendices A and B). HLA typing will be performed on samples for all participants vaccinated at a dosage level, provided T-cell responses are detected at that dosage level. The results will be kept confidential and are only for the purpose of characterizing the T-cell immune response. Volunteers will not receive the results of HLA typing.

Samples may also be tested for single nucleotide polymorphism (SNP) or microsatellite analysis using either arrays or targeted approaches. These approaches may evolve as the techniques become more sophisticated.

12.3.2 HIV Test

Samples will be tested at the time points indicated in the Schedule of Procedures (Appendices A and B). Further information is specified in Section 14.1 HIV Testing.

12.3.3 Pregnancy Test

A urine pregnancy test for all female volunteers will be performed by measurement of Human Chorionic Gonadotrophin (βhCG) at the time points indicated in the Schedule of Procedures (Appendices A and B).

The results of the pregnancy test must be negative prior to vaccination.

13.0 ADVERSE EVENTS

13.1 Definitions

- An adverse event (AE) is any untoward medical occurrence in a patient or clinical investigation subject administered a pharmaceutical product and which does not necessarily have a casual relationship with this treatment. An AE can therefore be any unfavorable and unintended sign (including an abnormal laboratory finding), symptom, or disease temporally associated with the use of a medicinal (investigational) product, whether or not related to the medicinal (investigational) product. (ICH E6) (Synonym: Adverse Experience).

- A serious adverse event (SAE) is any untoward medical occurrence that at any dose results in death, is life-threatening, requires inpatient hospitalization or prolongation of existing hospitalization, results in persistent or significant disability/incapacity, or is a congenital anomaly/birth defect. This includes important medical events that may not be immediately life-threatening or result in death or hospitalization but may jeopardize the

patient or may require intervention to prevent one of the outcomes listed in the definition above. (ICH E6 and E2A)

- A suspected adverse drug reaction (SADR) is an adverse event that could potentially have a causal relationship to the investigational product (definitely, probably, possibly, probably not related, or for deaths, pending).

- Expected events are based on events previously observed, *not* events that might be anticipated from the pharmacological properties of the investigational product (ICH E2A).

- Unexpected events are those events whose nature or severity (intensity) is not consistent with those included in the package insert/summary of the investigational products that have been approved by the U.S. FDA or in the Investigator's Brochure (ICH E2A).

13.2 Relationship of Adverse Event to Investigational Product

The study physician makes the site's final assessment of the causal association based upon the temporal relationship to administration of the investigational product (s), the pharmacology of the investigational product(s), and his/her clinical judgment. The terms used in DAIDS studies/trials to assess relationship of an event to investigational product are:

13.2.1 Categories of Relatedness

1. **Definitely Related**. The adverse event and administration of investigational product are related in time, and a direct association can be demonstrated.

2. **Probably Related**. The adverse event and administration of study agent are reasonably related in time, and the adverse event is more likely explained by the investigational product than other cause.

3. **Possibly Related**. The adverse event and administration of study agent are reasonably related in time, and the adverse event can be explained equally well by causes other than investigational product.

4. **Probably Not Related**. A potential relationship between study agent and the adverse event could exist (i.e., the possibility cannot be excluded), but the adverse event is most likely explained by causes other than the investigational product.

5. **Not Related**. The adverse event is clearly explained by another cause not related to the investigational product.

6. **Pending.** Pending may be used as a temporary relationship assessment only for death and only if data necessary to determine relationship to study agent are being collected. The site is required to submit a final assessment within 3 business days after reporting the death. If no final assessment is made within 3 business days after the date of submission, the event will be assessed as possibly related to investigational product. Any additional information received at a later time, including an autopsy report, should be submitted as a Follow-up Report.

13.2.2 Investigational Products to be Considered for Assessing Relatedness
The investigational products that must be considered in determining relationships of AEs requiring expedited reporting to DAIDS are:

- VRC-HIVDNA016-00-VP vaccine/ placebo
- VRC-HIVADV014-00-VP vaccine/ placebo
- Phosphate buffered saline (PBS)
- VRC-DILUENT013-DIL-VP Final Formulation Buffer (FFB).

13.3 Grading Severity of Adverse Events

The Division of AIDS Table for Grading the Severity of Adult and Pediatric Adverse Events (DAIDS AE Grading Table, **Appendix C**), *Version 1.0, December 2004* must be used and is available on the RCC website at http://rcc.tech-res-intl.com/

13.4 Expedited Reporting of Adverse Events

13.4.1 Adverse Events Requiring Expedited Reporting to DAIDS
This study uses the Standard Level of expedited AE reporting as defined in the DAIDS EAE Manual. At this level, report all adverse events following any exposure to investigational product that:

1. Result in death regardless of relationship to investigational product.
2. Are congenital anomalies, birth defects, or fetal losses regardless of relationship to investigational product.
3. Result in persistent or significant disabilities or incapacities regardless of relationship to investigational product.
4. Are a suspected adverse drug reaction, i.e., definitely, probably, possibly, and probably not related, to an investigational product that requires or prolongs existing hospitalization.
5. Are a suspected adverse drug reaction, i.e., definitely, probably, possibly, and probably not related, to an investigational product that requires intervention to prevent significant/permanent disability or death.
6. Are a suspected adverse drug reaction, i.e. definitely, probably, possibly, and probably not related to an investigational product that is life-threatening (including all Grade 4 adverse events)
7. "Life threatening" refers to an adverse event that at occurrence represented an immediate risk of death to the subject. An experience which may have caused death had it occurred in a more severe form is not considered life threatening. Similarly, a hospital admission for an elective procedure is not considered a Serious Adverse event.

13.4.2 Additional Adverse Events that Should be Reported for Any Study/Trial Requiring Expedited Reporting to DAIDS
In addition to the reporting requirements described above, sites should report any of the following adverse events on an expedited basis:

1. Suspected adverse drug reactions, i.e., definitely, probably, possibly, and probably not related to an investigational product, that **do not meet the protocol-required reporting criteria**, but the Investigator believes are of sufficient concern to be reported on an expedited basis to DAIDS. This includes adverse events that, based

upon appropriate medical judgment, may jeopardize the subject and may require medical or surgical intervention to prevent a serious adverse event. Examples of such events are intensive treatment in an emergency room or at home for allergic bronchospasm or blood dyscrasias or convulsions that do not result in hospitalization.

2. Unexpected, serious suspected adverse drug reactions, i.e., definitely, probably, possibly, and probably not related to an investigational product, that occur at any time **after the protocol-defined expedited reporting period** if the study staff become aware of its occurrence. These events include deaths, permanent disabilities, congenital anomalies, hospitalizations, and life-threatening clinical events. (Do not report Grade 4 laboratory values unless associated with a life-threatening clinical event.)
3. Serious adverse events that are not related to an investigational product, but could be associated with **study participation or procedure** (e.g., pulmonary embolism secondary to an intravenous catheter placed for administration of investigational product).
4. HIV Infection if volunteer becomes infected during the course of the study.

13.4.3 Method and Timeframe for Reporting of Individual Adverse Events as Per DAIDS Manual

Information on adverse events is collected by a clinical staff member, entered onto CRF pages and transmitted to the data center. These data are reviewed on an ongoing basis by the study coordinator and the principal investigator and study safety team in regular basis. Additional participants could include co-investigators and senior clinical research nursing staff and data management. The site will send the CRF pages to data center (DC) and those data will be available in tabular forms weekly for review by the IAVI Medical Monitor and PSRT. Safety calls of the PSRT review will be conducted regularly, and ad hoc calls may be convened if a PSRT member has safety concern after his/her weekly review. PSRT calls may be attended by a representative of the RV 172 and HVTN 204 teams and Division of AIDS. A quorum of four of the Safety Team will be required for study modification. And, as per the study IND holder, DAIDS, study modifications must be approved by the internal DAIDS Processes for Study Amendments and/or Letters of Amendments. Additionally, any LoA of study amendments must also be approved before implementation by IRBs/ECs.

The expedited adverse event (EAE) reporting requirements and definitions for this study and the methods for expedited reporting of adverse events (AEs) to the DAIDS Regulatory Compliance Center (RCC) Safety Office are defined in “The Manual for Expedited Reporting of Adverse Events to DAIDS” (DAIDS EAE Manual) **dated May 6, 2004**. The DAIDS EAE Manual is available on the RCC website (see below).

All information requested on the DAIDS Expedited Adverse Event Reporting Form must be provided and the form submitted to the DAIDS RCC Safety Office. This form can be found at the web site for the DAIDS RCC Safety Office. IAVI will coordinate with the site for EAE reporting to DAIDS RCC Safety Office on time. For questions or other communication, please note the following:

Website:	http://rcc.tech-res-intl.com
Office Phone*:	1-800-537-9979 (U.S. only) or +1-301-897-1709

Office Fax*:	1-800-275-7619 (U.S. only) or +1-301-897-1710
Office Email:	RCCSafetyOffice@tech-res.com
Office Hours:	Monday through Friday, 8:30 AM to 5:00 PM (U.S. Eastern Time)
Mailing Address:	DAIDS Safety Office 6500 Rock Spring Drive Suite 650 Bethesda, MD 20817

*Office phone and fax are accessible 24 hours per day.

The time frame for expedited reporting of individual adverse events begins when the site recognizes that an event fulfills the criteria outlined in the manual for expedited reporting to DAIDS. Sites must submit adverse events requiring expedited reporting to the DAIDS RCC Safety Office as soon as possible, **but no later than 3 business days**, after the site's recognition that the event fulfills the criteria for expedited reporting. The IND holder is responsible for submitting IND safety reports to the FDA, as necessary, per 21 CFR 312.32. DAIDS submits IND safety reports as soon as possible, but no later than 15 days after initial receipt of the information.

13.4.4 Adverse Event Expedited Reporting Periods

AEs must be reported on an expedited basis at the Standard Level during the Protocol-defined EAE Reporting Period, which is the entire study duration for an individual subject (from study enrollment until study completion or discontinuation of the subject from study participation for any reason).

After the end of the Protocol-defined EAE Reporting Period stated above, sites must report serious, unexpected, clinical suspected adverse drug reactions if the study site staff becomes aware of the event on a passive basis, i.e., from publicly available information.

13.5 Clinical Management of Adverse Events

Adverse events (AEs) will be managed in accordance with good medical practices by the clinical study team who will assess and treat the volunteer as appropriate, including referral. All volunteers experiencing adverse events, regardless of severity, will be followed until satisfactory resolution, return to baseline, or until the toxicity is presumed to be irreversible. If at the end of the study, an adverse event (including clinically significant lab abnormality) which is considered possibly, probably or definitely related to the investigational product is unresolved, follow-up will continue until resolution if possible and the volunteer will be referred. If any treatment/medical care is required as a result of the harm caused by the investigational product or study procedures, this will be provided free of charge.

If a volunteer has an abnormal laboratory value that is known at the time of study injection(s), the following guidelines apply:

- For a mild laboratory abnormality, volunteers can be vaccinated only if the abnormality is judged to be not clinically significant by the principal investigator or designee,
- For a moderate laboratory abnormality, the laboratory test must be repeated and the event determined to be resolved in the opinion of the principal investigator or designee prior to the study injection(s)

- For a severe laboratory abnormality, even if resolved, the IAVI Medical Monitor must be consulted before making a decision to vaccinate (for further information see Section 15.0)

- For a life-threatening laboratory abnormality even if resolved, the IAVI Medical Monitor must be consulted before making a decision to vaccinate (for further information see Section 15.0)

13.6 Pregnancy

Although not considered an adverse event, if a female volunteer becomes pregnant during the study, it is the responsibility of the Principal Investigator or designee to report the pregnancy promptly to IAVI using the designated case report forms. However, serious complications of pregnancy that meet SAE criteria (e.g. eclampsia, spontaneous abortion, etc.) should be reported as an SAE. For follow up on a pregnancy refer to Section 15.2.

If a female volunteer becomes pregnant during the study, vaccinations will be discontinued and the volunteer will be followed for safety information until the end of pregnancy or study completion, whichever occurs last. Approximately 2-4 weeks after delivery, the baby will be examined by a physician to assess its health status and the results will be reported to IAVI.

13.7 Intercurrent HIV Infection

HIV infection cannot be caused by the investigational product. If a volunteer is found to be HIV-infected through exposure in the community, study vaccinations must be discontinued and the volunteer followed according to procedures described in Section 15.2.

Intercurrent HIV infection in study volunteers, although not considered an SAE, must be reported as specified in Section 13.4. Furthermore, serious medical conditions associated with the HIV infection that meet SAE criteria specified in this Protocol (e.g. sepsis, PCP pneumonia) should also be reported as an SAE as specified in Section 13.4.

14.0 MANAGEMENT OF HIV ISSUES DURING AND FOLLOWING STUDY

14.1 HIV Testing

Volunteers will be tested for HIV-1 and HIV-2 antibodies as indicated in the Schedule of Procedures (Appendix A and B) or as needed, if medical or concern about exposure arise.

HIV testing will be conducted by performing two standard HIV1/2 ELISAs at each required timepoint.. In the case of discrepant ELISA results and to confirm HIV infection, nucleic-acid-based assay(s) (e.g. HIV RNA by PCR) will be performed on specimens with positive or indeterminate results. The results will be interpreted according to a pre-defined testing algorithm (see Appendix E, HIV Testing Algorithm):

1. If the results of both ELISAs are negative, this will be reported as “Not infected with HIV-1 or HIV-2”.
2. If the results of the two ELISAs are positive, confirmatory testing using nucleic-acid-based assay (e.g. HIV RNA by PCR or other) will be performed. If the results indicate the presence of HIV,, all the tests will be repeated using a new specimen. The

repeatedly positive results of nucleic-acid-based assay will be reported as "HIV infected".

3. If the results of the two ELISA are discrepant, the testing will be repeated using the same specimen. If the repeated results remain discrepant, confirmatory testing using nucleic-acid-based assay (e.g. HIV RNA by PCR or other) will be performed. If the results indicate a presence of detectable HIV the tests will be repeated using a new specimen. The repeatedly positive results of nucleic-acid-based assay will be reported as "HIV infected". If the results indicate absence of detectable HIV, the results will be reported as "indeterminate" and all tests (ELISA and nucleic-based assay) repeated in 4 weeks. Additional tests may be performed to differentiate HIV-1 from HIV-2 infection.

Study injections will be discontinued for volunteers with confirmed HIV infection and they will be followed for additional 12 months to obtain viral load and CD4 counts and other clinical information. They will be also referred for care and management according to the national guidelines in Kenya and Rwanda, respectively (as specified in the section 14.3.2).

Volunteers who have a positive HIV-antibody test(s) as a result of vaccine-induced HIV antibodies rather than a true HIV infection (false positive HIV test) will have their test result reported as "Not infected with HIV-1 or HIV-2" (to prevent unblinding of volunteer and staff) and will be followed up until the test becomes negative. Volunteers who are not infected with HIV but have vaccine-induced false positive HIV test at the end of the study will be offered a continuing follow-up test, every 6 months, until the test becomes negative.

Should a volunteer require an HIV test outside the study for personal reasons or if medical or social circumstances arise, it is recommended that the volunteer contacts the site personnel first. The HIV test can be performed at the study site as above. Written information regarding the HIV status will be provided to volunteers upon request. Written evidence of HIV status (HIV-infected or Not infected with HIV) will be provided to study volunteers upon request.
All volunteers will receive HIV prevention counseling and pre-HIV-test and post-HIV-test counseling as specified in the Study Operations Manual.

14.2 Social Discrimination as a Result of an Antibody Response to Vaccine

The aim is to minimize the possibility of social discrimination in volunteers (if any) who develop vaccine-induced HIV antibodies and test positive on a diagnostic HIV antibody test. Appropriate diagnostic HIV testing and certification will be provided both during and after the study as needed. In addition, sites will have the option of offering the volunteer a card stating that they are participating in a research study.

14.3 HIV infection

Volunteers who are found to be HIV infected at screening and volunteers who acquire HIV infection during the study will be provided the following:

14.3.1 Counseling

The volunteer will be counseled by the study counselors. The counseling process will assist the volunteer with the following issues:

- Psychological and social implications of HIV infection
- Who to inform and what to say
- Implications for sexual partners

- Implications for child-bearing
- Avoidance of transmission to others in future

14.3.2 Referral for Support and/or Care

Volunteers will be referred to a patient support center or institution of their choice for a full discussion of the clinical aspects of HIV infection. Referral will be made to a designated physician or center for discussion of options of treatment of HIV-infection.

For those individuals who become HIV infected after enrollment in the study, antiretroviral therapy will be provided when clinically indicated according to accepted treatment guidelines.

HIV-infected pregnant women will be referred for prenatal care and to a program for the Prevention of Mother to Child Transmission (PMTCT). The pregnant volunteer will be followed according to timeline as specified in this protocol.

15.0 DISCONTINUATION OF VACCINATIONS AND/OR WITHDRAWAL FROM STUDY

15.1 Discontinuation of Vaccinations in Individual Volunteer

Any volunteer discontinuing from further vaccinations or being considered for discontinuation of vaccinations will be discussed with the sponsor. Volunteers will be discontinued from further vaccination for any of the following reasons:

1. Pregnancy
2. Intercurrent HIV Infection
3. Use of systemic corticosteroids, immunosuppressive, antiviral, anticancer, or other medications.
4. A disease or condition or an adverse event that may develop, regardless of relationship to the investigational product, if the principal investigator or designee is of the opinion that further study vaccinations will jeopardize the safety of the volunteer.
5. Any abnormal laboratory parameters considered clinically significant by the PI and IAVI Medical Monitor.
6. Receipt of live attenuated vaccine within the previous 60 days (live attenuated flu vaccine within 14 days) or planned receipt within 60 days after vaccination with investigational product or receipt of other vaccine within the previous 14 days or planned receipt within 14 days after vaccination with investigational product
7. A severe local reactogenicity involving the major part of the injected arm circumference.
8. Anaphylaxis; bronchospasm; laryngeal edema; convulsions or encephalopathy following study vaccinations.
9. Life threatening adverse event following study vaccinations unless not related to the investigational product and fully resolved.
10. Any immediate hypersensitivity reaction judged to be definitely related to the investigational product.
11. Volunteer request to discontinue further vaccination.
12. Participating in another clinical study of an investigational product

15.2 Follow Up After Discontinuation of Further Vaccinations

Volunteers, in whom study vaccinations are discontinued due to adverse events, will be followed until the adverse event resolves or stabilizes or up to the end of the study, whichever comes last. These volunteers will not be replaced.

Follow-up of HIV infected individuals who have received investigational product will be determined by the Primary Investigator and the IAVI Medical Monitor.

Follow-up of pregnant volunteers will be done as specified in this protocol.

15.3 Withdrawal from the Study (Early Termination)

Volunteers may be withdrawn from the study permanently for the following reasons:

1. Volunteers may withdraw from the study at any time if they wish, for any reason.
2. The principal investigator or designee has reason to believe that the volunteer is not complying with the protocol.
3. If the US FDA, national regulatory authorities in Kenya or Rwanda, NIH (DAIDS and VRC), IAVI or IRB/EC decides to terminate or suspend the study.

15.4 Follow-up Withdrawal from the Study (Early Termination)

If the volunteer withdraws from the study, all termination visit procedures will be performed according to the Schedule of Procedures (Appendices A and B) where possible. Every effort will be made to determine and document the reason for withdrawal from the study.

16.0 DATA HANDLING

16.1 Data Collection and Record Keeping at the Study Site

Data Collection: All study data will be collected by the clinical study staff using source documents and entered onto the appropriate case report forms (CRFs). CRFs will be provided by IAVI and should be handled in accordance with the instructions from IAVI. All study data must be verifiable to the source documentation. A file will be held for each volunteer at the clinic(s) containing all the CRFs. Source documentation will be available for review to ensure that the collected data are consistent with the CRFs.

All CRFs and laboratory reports will be reviewed by the clinical team, who will ensure that they are accurate and complete.

CRFs, source documents and other supporting documents will be kept in a secure location and remain separate from volunteer identification information (name, address, etc.) to ensure confidentiality.

Standard GCP practices will be followed to ensure accurate, reliable and consistent data collection.

Source documents include but are not limited to:

- Signed Informed Consent Documents
- Dates of visits including dates of vaccinations
- Documentation of any existing conditions or past conditions relevant to eligibility
- Reported laboratory results
- All adverse events
- Concomitant medications
- Local and systemic reactogenicity

16.2 Data Collection and Transfer at the IAVI Core Laboratory

Data generated at the IAVI Core laboratory will be transferred directly to the Data Coordinating Center.

16.3 Data Entry at the Study Site

The data collected at the site will be

- Entered onto the CRFs by the site personnel. To provide for real time assessment of safety, data should be entered as soon as reasonably feasible (e.g. within one week) of a visit.
- If immunogenicity testing is performed locally, immunogenicity results will be transferred within 2 weeks of the assay being performed
- Safety review tables will be updated weekly and available for review by the Protocol Safety Team (DAIDS, VRC, IAVI and investigators).

16.4 Data Analysis

The data analysis plan will be developed and agreed upon by the sponsor and the principal investigator prior to unblinding of the study. The statistician at the Data Coordinating Center in collaboration with the principal investigator and the sponsor will create tables according to this data analysis plan. The DCC will conduct the data analysis and will provide interim and final study reports for the sponsor, principal investigator, and the DSMB as appropriate.

In order to efficiently promote the development of the vaccine candidate, immunogenicity data taken one month after the Ad5 boost time point will be analyzed along with the cumulative safety data and adenovirus serologies for all volunteers, by study group (vaccine vs. placebo). Should the Sponsor and collaborators determine that analysis of the immunogenicity data needs to be performed prior to availability of data for the entire targeted enrollment, that analysis will be performed according to a modified SAP (Statistical Analysis Plan).

An official SAP for the analysis of the combined data from the three studies (HVTN-204, RV-172 and IAVI-V001) will be developed by NIAID in collaboration with the three networks (HVTN, USMHRP and IAVI).

17.0 STATISTICAL CONSIDERATIONS

17.1 Sample Size

A total of 64 volunteers (48 Vaccine/16 placebo) will be entered into 4 groups scheduled to receive vaccine or placebo. Within each group, volunteers will be allocated in a 3:1 vaccine to placebo ratio and all will be followed for reactogenicity and systemic toxicity.

17.2 Statistical Power and Analysis

Safety and Tolerability:
The rate of local and systemic reactogenicity will be assessed in each group.

The rate of Serious Adverse Events related to the investigational product will be used as one measure of the safety of the investigational product. Adverse events that may be temporarily incapacitating (for example, loss or cancellation of work or social activities), which could make

an investigational product impractical for large scale use if they occur in more than a small proportion of cases, will also be assessed.

All adverse events will be reported, grouped as to whether or not they qualify as SAEs, their severity assessment, and their relationship to the Investigational Product (as judged by the investigator).
For life-threatening adverse events related to investigational product: if none of the volunteers receiving the investigational product experiences such events (n=48) the 95% upper confidence bound for the rate of these adverse events in the population is 0.06

Immunogenicity:
Cellular immune responses will be analyzed using binomial methods to examine for the presence or absence of HIV specific T-cell responses quantified by ELISPOT and intracellular cytokine staining (ICS). Assays will be performed using the SOPs and standard reagents of IAVI Core Laboratory and VRC lab for all volunteers.

Presence or absence of antibodies to HIV gene will be also analyzed. Assays will be performed in a similar fashion in all volunteers.

Response rates will be determined and compared for the dosage groups who receive the investigational product. Separately, the volunteers receiving placebo will be compared with the investigational product groups. Because of the small sample sizes and multiple epitopes, the results will be primarily descriptive.

Based on the previous experience with IAVI Phase 1 investigational product studies, it is expected that the amount of missing, unused or spurious data will be insignificant. Unused and spurious data will be listed separately and excluded from the statistical analysis. Missing data will be excluded from the statistical analysis.

18.0 QUALITY CONTROL AND QUALITY ASSURANCE

To ensure the quality and reliability of the data gathered and the ethical conduct of this study, a Study Operations Manual has been developed.

Regular monitoring will be performed according to ICH-GCP as indicated in section 20.3.

An independent audit of the study may be performed at the discretion of the sponsor.

By signing the protocol, the principal investigators, agree to facilitate study related monitoring, audits, IRB/IEC review and regulatory inspection(s) and direct access to source documents. Such information will be treated as strictly confidential and under no circumstances be made publicly available.

19.0 DATA AND BIOLOGICAL MATERIAL

All data and biological material collected through the study shall be managed in accordance with the Clinical Trial Agreements (CTA) between NIAID and IAVI and between IAVI and the sites. Distribution and use of that data will be conducted by agreement of all parties.

The computerized raw data generated will be held by the DCC on behalf of the sponsor. The study site will also hold the final data files and tables generated for the purpose of analysis.

20.0 ADMINISTRATIVE STRUCTURE

The principal investigator will be responsible for all aspects of the study at the study site.

20.1 Data Safety Monitoring Board (DSMB)

The DSMB will oversee the progress of the study. The DSMB will consist of independent clinicians/scientists/statisticians and/or ethicists who are not involved in the study and each participating country will be represented. Investigators responsible for the clinical care of volunteers or representative of the sponsor may not be members of the DSMB.

However, the DSMB may invite the principal investigator or designee and a sponsor representative to an open session of the meeting to provide information on study conduct, present data or to respond to questions.

The review of study data by the DSMB will take place regularly or may be specifically requested.

20.1.1 Content of Interim Review

The DSMB will be asked to review the following data:

- All severe and life threatening clinical adverse events/reactogenicity judged by the principal investigator or designee to be possibly, probably or definitely related to the investigational product, or

- All severe and life threatening laboratory adverse events confirmed on retest and judged by the principal investigator or designee to be possibly, probably, or definitely related to investigational product.

- All Serious Adverse Events, independent of relationship to the investigational product.

20.1.2 Criteria for Pausing the Study

Site Principal Investigators (PI), co-investigators and the entire sites' clinical research teams will exercise due diligence in ascertaining, accurately recording, promptly reporting, and actively acting on all adverse events (AEs). Adverse event case report forms (CRFs) will be completed daily and transmitted to the data center.

The site PI or designee will monitor study data including all adverse events (AEs) and laboratory data on a daily basis and will make determinations regarding the severity of adverse events and their relation to study agent. The site PI or medically qualified designee will identify any adverse event(s) requiring expedited reporting as per DAIDS procedures (See Section 13.4) or which may invoke study pause rules. The IAVI Medical Monitor will be notified by e-mail or telephone of any Reportable Adverse Events (RAE) or events triggering pause rules, and in turn will forward the information to other members of the Protocol Safety Review Team (PSRT). If a Grade 3 or 4 RAE is identified, the chairman of the PSRT will convene a teleconference as noted below (Table 20.1.2) to review the event and determine appropriate responses. Site investigators will complete a RAE form providing a full description of the event to facilitate its

discussion by the PSRT. These RAE forms will be faxed or emailed ONLY to members of the PSRT.

The PSRT will review aggregate safety data reports (available electronically) every week. Specific concerns will be resolved by teleconference. In case of one or more events that trigger an immediate review, as noted in Table 20.1.2, the IAVI Medical Monitor (HQ) or designee as PSRT chairman, will promptly convene an ad hoc teleconference to assess and advise regarding volunteer safety and potential study pause or termination, if necessary.

If a Grade 4 adverse event arises which is probably or definitely related to study treatment (See Section 13.2.1), all immunizations will be immediately paused pending review by the PSRT (See Table 20.1.2).

Clinic site teams at all study sites will be trained in recognition of post-injection reactogenicity events and AEs and the requirement for prompt action following certain events (See Table 20.1.2). The PSRT will assist all sites with this responsibility on the weekly data reviews and ad hoc as needed. The PSRT will include the following:

- IAVI Medical Monitor (HQ) or designee
- Site PIs or designees
- IAVI Medical Monitor (East Africa)
- DAIDS Medical Officer
- The VRC Representative

Additional PSRT participants may include, as needed, the following:

- A representative from USMHRP and a representative HVTN
- Co-investigators and site senior clinical research nursing staff
- Laboratory directors
- Data management and regulatory staff

PSRT members will be responsible for notifying appropriate individuals and boards or offices within their institutions and/or networks. DAIDS will be responsible for safety reporting to the U.S. FDA. IAVI will be responsible for providing copies to the study sites of any submitted DAIDS--FDA Safety Reports as well as the outcome of all PSRT--FDA pause decisions prompted by events defined in the Table 9. The site PIs will notify their Ethics Committee and national regulatory authorities as appropriate.

Adverse events that may trigger a pause rule must be reported in accordance with the pause rule table depicted below (Table 20.1.2), and in accordance with DAIDS EAE reporting guidelines utilizing the DAIDS EAE reporting form for submission. Sites will initially contact the IAVI Medical Monitor at HQ who will notify DAIDS Regulatory Compliance Center (RCC) Safety Office if the event meets EAE reporting requirements. RCC will provide IAVI with a copy of all the other sites' expedited adverse event and FDA safety reports. IAVI will be responsible for forwarding these reports to the clinical site. The sites will then forward to their respective local ethics and IRBs.

The process of notification and reporting will generally be fulfilled as follows:

- RCC will be responsible for distribution of adverse event and FDA safety reports to DAIDS Pharmacy Affairs Branch (PAB), DAIDS Vaccine Clinical Research Branch (VCRB), VRC, IAVI and U.S. FDA.

- IAVI will be responsible for distribution to the clinical sites.
- Site PIs will be responsible for ensuring that all reporting required by country specific authorities, including the IRB/IBC/EC as appropriate, is completed in a timely fashion.
- If an RAE is also an EAE, the sites' clinical teams will use DAIDS EAE form to notify both the PSRT and RCC via IAVI Medical Monitor at HQ.

TABLE 20.1.2 Adverse Event Notification and Immunization Pausing Rules

**Relatedness**	**Post-Injection Reactogenicity (PIR) and Study Treatment Related Adverse Events (AEs)**	**Action[1,4]**	**Criterion for Pausing or Pausing Consideration**
**Grade 4**<br>Probably or Definitely Related	"Verified"[5] abnormal laboratory values, local or systemic PIR, or other study treatment related AEs (if fever, it must persist for ≥ 48 hours)	Immediate (Concurrent with observation or report)[2]	≥ 1 Volunteer
**Grade 4**<br>Possibly related<br>**Grade 3**<br>Definitely, Probably or Possibly Related	"Verified" abnormal laboratory values, fever for ≥ 48 hours, vomiting, erythema, induration or other clinical study treatment related AEs (except subjective events)	Expedite[3]	≥ 1 Volunteer

[1]**Reporting** requirements for immunization pause consideration include certain defined local and systemic AEs that occur in the immediate post-injection period (within 72 hours of immunization), e.g. injection site- erythema, induration, pain, and tenderness or fever, vomiting, malaise, fatigue, headache, chills, nausea, myalgia and arthralgia. These AEs are termed post-injection reactogenicity (PIR). Other clinical or laboratory abnormalities that are possibly, probably or definitely related study treatment AEs and will also be reported, per above guidelines
[2] **Immediate Notification:** Site PI notifies the IAVI Medical Monitor immediately upon observation of the AE. The IAVI Medical Monitor notifies the, PSRT, DAIDS PAB, DAIDS VCRB, VRC, RCC and the other site immediately (within a few hours after the site observes, or is notified of, the AE). The RCC will process the AE forms but will not be involved in discussions regarding safety pauses. If AE is deemed probably or definitely related to study treatment, all study immunizations are held until further notice from the PSRT. If AE is deemed possibly related, immunization pause does not occur automatically; only after PSRT discussion and agreement. The PSRT will convene within one business day to review immediate safety reports. DAIDS notifies FDA of the PSRT decision and actions taken. Resumption of study immunizations requires concurrence of the FDA. IAVI notifies the clinical trial site of the outcome of the PSRT/FDA review. The sites notify their IRBs and regulatory authorities as appropriate.
[3] **Expedited Notification**: Site notifies the IAVI Medical Monitor immediately upon observation of the AE. The IAVI Medical Monitor notifies the PSRT, DAIDS PAB, DAIDS VCRB, VRC, RCC and other site within 24 hours of observation or notification of the AE. The RCC will process the AE forms but will not be involved in discussions regarding safety pauses. The PSRT convenes by teleconference and/or email within 48 hours of notification to determine if study immunizations should be held pending further review. In instances, in which this reporting/review timeline is not met, all study immunizations are to be held until the PSRT review and determination occurs.DAIDS notifies FDA of the PSRT decision. PSRT may also solicit the advice of FDA through the DAIDS Regulatory Affairs Branch. IAVI notifies the clinical trial site of the outcome of the PSRT/FDA review. The sites notify their Ethics Committee and regulatory authorities as appropriate.
[4] **Follow-Up and Resolution:** All AEs are followed until resolution or documented establishment of chronicity.
[5] **If no evidence of disease** is present other than the abnormal laboratory value, the test must be repeated (entailing blood re-draw) at least one time in order to be considered "verified". The verification period will be a maximum of 36 hours after initial awareness of the abnormal laboratory value. (Note that if any sign or symptom is present, it should be reported prior to receiving verification.)

20.2 Study Supervision

The Data Safety Monitoring Board (DSMB) and the IAVI Director of Medical Affairs will be provided progress report(s) of this study. Close cooperation between members of the DSMB

will be necessary address safety issues in a timely manner and assure consistent documentation and effective information sharing. Rates of accrual, retention, and other parameters relevant to the site's performance will be regularly and closely monitored by the study team.

20.3 Study Monitoring

On-site monitoring will be conducted to ensure that the study is conducted in compliance with human subjects and other research regulations and guidelines, recorded and reported in accordance with the protocol, is consistent with locally accepted HIV counseling practices, standard operating procedures, Good Clinical Practice (GCP) and applicable regulatory requirements. The study will be monitored by independent monitors.

The monitor will confirm the quality and accuracy of data at the site by validation against the source documents such as clinical records and against the database where applicable. The investigators and volunteers, by giving consent, agree that the monitor may inspect study facilities and source documents (e.g. informed consent forms, clinic and laboratory records, other source documents) as well as observe the performance of study procedures. Such information will be treated as strictly confidential and will under no circumstances be made publicly available.

The monitor will adhere to Good Clinical Practice guidelines. The principal investigator will permit inspection of the facilities and all study related documentation by authorized representatives of IAVI, and government and regulatory authorities relevant to this study. Representatives of DAIDS may accompany the IAVI monitors to co-monitor, at mutually agreeable times.

20.4 Investigator's Records

Study records include administrative documentation, including reports and correspondence relating to the study and documentation related to each volunteer screened for and/or enrolled in the study, including informed consent forms, case report forms, and all other source documents. The investigator will maintain and store, in a secure manner, complete, accurate, and current study records for a minimum of 2 years after marketing application approval or the study is discontinued and applicable national and local health authorities are notified. IAVI will notify the principal investigator of these events.

21.0 INDEMNITY

The sponsor and institution are responsible to have appropriate liability insurance. For research related injuries and/or medical problems determined to result from receiving the investigational product, treatment including necessary emergency treatment and proper follow-up care will be made available to the volunteer free of charge at the expense of the sponsor.

22.0 PUBLICATION

A primary manuscript describing safety and immune responses in this trial will be prepared promptly after the data analysis is available, based on the data compiled by the IAVI statistical centre. Authors will be representatives of each trial site, the statistical center, the laboratories

and IAVI, subject to the generally accepted criteria of contributions to the design, work, analysis and writing of the study. Precedence will be given to authors from the site enrolling the greatest number of volunteers. Manuscripts will be reviewed by representatives of each participating group as specified in the CTA. The process of preparation for publication should follow the agreement described in CTA between IAVI and NIAID and IAVI and the site.

23.0 ETHICAL CONSIDERATIONS

The principal investigator will ensure that the study is conducted in compliance with the protocol, standard operating procedures in accordance with guidelines laid down by the International Conference on Harmonisation for Good Clinical Practice in clinical studies and applicable regulatory requirements.

REFERENCES

1. UNAIDS, WHO. AIDS epidemic update. **2002**;UNAIDS/02.46E:1-36.

2. Centers for Disease Control and Prevention. HIV/AIDS surveillance report. **2001**;1-48.

3. UNAIDS. Report on the global HIV/AIDS epidemic. **2002**;UNAIDS/02.26E.

4. Fauci AS. The AIDS epidemic--considerations for the 21st century. N Engl J Med **1999**;341:1046-50.

5. Varmus H, Nathanson N. Science and the control of AIDS. Science **1998**;280:1815.

6. McCutchan FE. Understanding the genetic diversity of HIV-1. AIDS **2000**;14 Suppl 3:S31-S44.

7. Kuiken CL, Lukashov VV, Baan E, Dekker J, Leunissen JA, Goudsmit J. Evidence for limited within-person evolution of the V3 domain of the HIV-1 envelope in the amsterdam population. AIDS **1996**;10:31-7.

8. Moore JP, Parren PW, Burton DR. Genetic subtypes, humoral immunity, and human immunodeficiency virus type 1 vaccine development. J Virol **2001**;75:5721-9.

9. Shankarappa R, Margolick JB, Gange SJ, et al. Consistent viral evolutionary changes associated with the progression of human immunodeficiency virus type 1 infection. J Virol **1999**;73:10489-502.

10. Nixon DF, Townsend AR, Elvin JG, Rizza CR, Gallwey J, McMichael AJ. HIV-1 gag-specific cytotoxic T lymphocytes defined with recombinant vaccinia virus and synthetic peptides. Nature **1988**;336:484-7.

11. Emini, E. A. A potential HIV-1 vaccine using a replication-defective adenoviral vaccine vector. 9th conference of retroviruses and opportunistic infections. Seattle, **2002**.

12. Korber B, Brander C, Haynes B, Koup R, Kuiken C, Moore JP, Watkins DI. HIV molecular immunology 2000. Los Alamos, NM: Los Alamos National Laboratory, Theoretical Biology and Biophysics, **2000**.

13. Gaschen B, Taylor J, Yusim K, et al. Diversity considerations in HIV-1 vaccine selection. Science **2002**;296:2354-60.

14. Approaches to the development of broadly protective HIV vaccines: challenges posed by the genetic, biological and antigenic variability of HIV-1: Report from a meeting of the WHO-UNAIDS Vaccine Advisory Committee Geneva, 21-23 February 2000. AIDS **2001**;15:W1-W25.

15. Osmanov S, Pattou C, Walker N, Schwardlander B, Esparza J. Estimated global distribution and regional spread of HIV-1 genetic subtypes in the year 2000. J Acquir Immune Defic Syndr **2002**;29:184-90.

16. Charo J, Lindencrona JA, Carlson LM, Hinkula J, Kiessling R. Protective efficacy of a DNA influenza virus vaccine is markedly increased by the coadministration of a Schiff base-forming drug. J Virol **2004**;78:11321-6.

17. Wang R, Doolan DL, Le TP, et al. Induction of antigen-specific cytotoxic T lymphocytes in humans by a malaria DNA vaccine. Science **1998**;282:476-80.

18. Le TP, Coonan KM, Hedstrom RC, et al. Safety, tolerability and humoral immune responses after intramuscular administration of a malaria DNA vaccine to healthy adult volunteers. Vaccine **2000**;18:1893-901.

19. Tacket CO, Roy MJ, Widera G, Swain WF, Broome S, Edelman R. Phase 1 safety and immune response studies of a DNA vaccine encoding hepatitis B surface antigen delivered by a gene delivery device. Vaccine **1999**;17:2826-9.

20. Kotsopoulou E, Kim VN, Kingsman AJ, Kingsman SM, Mitrophanous KA. A Rev-independent human immunodeficiency virus type 1 (HIV-1)-based vector that exploits a codon-optimized HIV-1 gag-pol gene. J Virol **2000**;74:4839-52.

21. zur Megede J, Chen MC, Doe B, et al. Increased expression and immunogenicity of sequence-modified human immunodeficiency virus type 1 gag gene. J Virol **2000**;74:2628-35.

22. Kim JJ, Yang JS, VanCott TC, et al. Modulation of antigen-specific humoral responses in rhesus macaques by using cytokine cDNAs as DNA vaccine adjuvants. J Virol **2000**;74:3427-9.

23. Sedegah M, Weiss W, Sacci JB, Jr., et al. Improving protective immunity induced by DNA-based immunization: priming with antigen and GM-CSF-encoding plasmid DNA and boosting with antigen-expressing recombinant poxvirus. J Immunol **2000**;164:5905-12.

24. Boyer JD, Kim J, Ugen K, et al. HIV-1 DNA vaccines and chemokines. Vaccine **1999**;17 Suppl 2:S53-S64.

25. Mascola JR, Nabel GJ. Vaccines for the prevention of HIV-1 disease. Curr Opin Immunol **2001**;13:489-95.

26. Barouch DH, Santra S, Schmitz JE, et al. Control of viremia and prevention of clinical AIDS in rhesus monkeys by cytokine-augmented DNA vaccination. Science **2000**;290:486-92.

27. Boyer JD, Cohen AD, Vogt S, et al. Vaccination of seronegative volunteers with a human immunodeficiency virus type 1 env/rev DNA vaccine induces antigen-specific proliferation and lymphocyte production of beta-chemokines. J Infect Dis **2000**;181:476-83.

28. Mwau M, Cebere I, Sutton J, et al. A human immunodeficiency virus 1 (HIV-1) clade A vaccine in clinical trials: stimulation of HIV-specific T-cell responses by DNA and recombinant modified vaccinia virus Ankara (MVA) vaccines in humans. J Gen Virol **2004**;85:911-9.

29. Isaacs, R. Impact of pre-existing immunity on the immunogenicity of adenovirus serotype 5-based vaccines. AIDS Vaccine 2004. Lausanne, Switzerland, **2004**.

30. Shiver, J. Development of an anti-HIV-1 vaccine based on replication defective adenovirus. Keystone Symposium on HIV Vaccine Development. Whistler, British Columbia, Canada, **2004**.

31. DeRose, R., Dale, C. J., Stratov, I., Chea, S., Purcell, D., Ramshaw, I. A., Thomson, S., Boyle, D. B., Coupar, B., Ramsay, A. J., French, R., Law, M., Emery, S., Cooper, D. A., Kent, S. J. Efficacy of DNA and fowlpox prime/boost vaccines for HIV-1 and SHIV. World AIDS Conference. Bangkok, Thailand, **2004**.

32. Plotkin S, Orenstein WA. Vaccines. Philadelphia: W.B. Saunders Co., **1999**;1230.

33. Schwartz AR, Togo Y, Hornick RB. Clinical evaluation of live, oral types 1, 2, and 5 adenovirus vaccines. Am Rev Respir Dis **1974**;109:233-9.

34. Rosenbaum MJ, Poudstone JW, Edwards EA. Acceptability and antigenicity of adjuvant soluble sub-unit adenovirus vaccines in navy recruits. Mil Med **1976**;141:383-8.

35. Rosenbaum MJ, Edwards EA, Hoeffler DF CAPT. Recent experiences with live adenovirus vaccines in navy recruits. Mil Med **1975**;140:251-7.

36. Gaydos CA, Gaydos JC. Adenovirus vaccines in the U.S. military. Mil Med **1995**;160:300-4.

37. Harvey BG, Leopold PL, Hackett NR, et al. Airway epithelial CFTR mRNA expression in cystic fibrosis patients after repetitive administration of a recombinant adenovirus. J Clin Invest **1999**;104:1245-55.

38. Bellon G, Michel-Calemard L, Thouvenot D, et al. Aerosol administration of a recombinant adenovirus expressing CFTR to cystic fibrosis patients: a phase I clinical trial. Hum Gene Ther **1997**;8:15-25.

39. Hay JG, McElvaney NG, Herena J, Crystal RG. Modification of nasal epithelial potential differences of individuals with cystic fibrosis consequent to local administration of a normal CFTR cDNA adenovirus gene transfer vector. Hum Gene Ther **1995**;6:1487-96.

40. Harvey BG, Worgall S, Ely S, Leopold PL, Crystal RG. Cellular immune responses of healthy individuals to intradermal administration of an E1-E3- adenovirus gene transfer vector. Hum Gene Ther **1999**;10:2823-37.

41. Rosengart TK, Lee LY, Patel SR, et al. Six-month assessment of a phase I trial of angiogenic gene therapy for the treatment of coronary artery disease using direct intramyocardial administration of an adenovirus vector expressing the VEGF121 cDNA. Ann Surg **1999**;230:466-70.

42. Rosengart TK, Lee LY, Patel SR, et al. Angiogenesis gene therapy: phase I assessment of direct intramyocardial administration of an adenovirus vector expressing VEGF121

cDNA to individuals with clinically significant severe coronary artery disease. Circulation **1999**;100:468-74.

43. Sterman DH, Treat J, Litzky LA, et al. Adenovirus-mediated herpes simplex virus thymidine kinase/ganciclovir gene therapy in patients with localized malignancy: results of a phase I clinical trial in malignant mesothelioma. Hum Gene Ther **1998**;9:1083-92.

44. Clayman GL, Frank DK, Bruso PA, Goepfert H. Adenovirus-mediated wild-type p53 gene transfer as a surgical adjuvant in advanced head and neck cancers. Clin Cancer Res **1999**;5:1715-22.

45. Swisher SG, Roth JA, Nemunaitis J, et al. Adenovirus-mediated p53 gene transfer in advanced non-small-cell lung cancer. J Natl Cancer Inst **1999**;91:763-71.

46. Gahery-Segard H, Molinier-Frenkel V, Le Boulaire C, et al. Phase I trial of recombinant adenovirus gene transfer in lung cancer. Longitudinal study of the immune responses to transgene and viral products. J Clin Invest **1997**;100:2218-26.

47. Tursz T, Cesne AL, Baldeyrou P, et al. Phase I study of a recombinant adenovirus-mediated gene transfer in lung cancer patients. J Natl Cancer Inst **1996**;88:1857-63.

48. Harvey BG, Maroni J, O'Donoghue KA, et al. Safety of local delivery of low- and intermediate-dose adenovirus gene transfer vectors to individuals with a spectrum of morbid conditions. Hum Gene Ther **2002**;13:15-63.

49. Harvey BG, Hackett NR, El Sawy T, et al. Variability of human systemic humoral immune responses to adenovirus gene transfer vectors administered to different organs. J Virol **1999**;73:6729-42.

50. Rajagopalan S, Trachtenberg J, Mohler E, et al. Phase I study of direct administration of a replication deficient adenovirus vector containing the vascular endothelial growth factor cDNA (CI-1023) to patients with claudication. Am J Cardiol **2002**;90:512-6.

51. Crystal RG, Harvey BG, Wisnivesky JP, et al. Analysis of risk factors for local delivery of low- and intermediate-dose adenovirus gene transfer vectors to individuals with a spectrum of comorbid conditions. Hum Gene Ther **2002**;13:65-100.

52. Raper SE, Yudkoff M, Chirmule N, et al. A pilot study of in vivo liver-directed gene transfer with an adenoviral vector in partial ornithine transcarbamylase deficiency. Hum Gene Ther **2002**;13:163-75.

53. Assessment of adenoviral vector safety and toxicity: report of the National Institutes of Health Recombinant DNA Advisory Committee. Hum Gene Ther **2002**;13:3-13.

54. Varnavski AN, Zhang Y, Schnell M, et al. Preexisting immunity to adenovirus in rhesus monkeys fails to prevent vector-induced toxicity. J Virol **2002**;76:5711-9.

55. Yang Y, Wilson JM. Clearance of adenovirus-infected hepatocytes by MHC class I-restricted CD4+ CTLs in vivo. J Immunol **1995**;155:2564-70.

56. Yang Y, Nunes FA, Berencsi K, Furth EE, Gonczol E, Wilson JM. Cellular immunity to viral antigens limits E1-deleted adenoviruses for gene therapy. Proc Natl Acad Sci U S A **1994**;91:4407-11.

57. Nunes FA, Furth EE, Wilson JM, Raper SE. Gene transfer into the liver of nonhuman primates with E1-deleted recombinant adenoviral vectors: safety of readministration. Hum Gene Ther **1999**;10:2515-26.

58. Lozier JN, Csako G, Mondoro TH, et al. Toxicity of a first-generation adenoviral vector in rhesus macaques. Hum Gene Ther **2002**;13:113-24.

59. Morral N, O'Neal WK, Rice K, et al. Lethal toxicity, severe endothelial injury, and a threshold effect with high doses of an adenoviral vector in baboons. Hum Gene Ther **2002**;13:143-54.

60. Fu TM, et al. Replication-incompetent recombinant adenovirus vector expressing SIV Gag elicits robust and effective cellular immune responses in rhesus macaques. AIDS Vaccine 2001, **2001**.

61. Tang A, et al. Evaluation of the immunogenicity of HIV-1 vaccine in baboons. AIDS Vaccine 2001, **2001**.

62. Casimiro D, et al. Preclinical evaluation of gene-delivery-based HIV-1 vaccine in nonhuman primates. AIDS Vaccine 2001, **2001**.

63. Shiver JW, Fu TM, Chen L, et al. Replication-incompetent adenoviral vaccine vector elicits effective anti-immunodeficiency-virus immunity. Nature **2002**;415:331-5.

64. Sullivan NJ, Sanchez A, Rollin PE, Yang ZY, Nabel GJ. Development of a preventive vaccine for Ebola virus infection in primates. Nature **2000**;408:605-9.

65. Yang ZY, Wyatt LS, Kong WP, Moodie Z, Moss B, Nabel GJ. Overcoming immunity to a viral vaccine by DNA priming before vector boosting. J Virol **2003**;77:799-803.

66. Emini, E. A. Ongoing Development and Evaluation of a Potential HIV-1 Vaccine Using a Replication-Defective Adenoviral Vector. Keystone Conference on HIV Vaccines. Banff, **2003**.

67. Chirmule N, Propert K, Magosin S, Qian Y, Qian R, Wilson J. Immune responses to adenovirus and adeno-associated virus in humans. Gene Ther **1999**;6:1574-83.

68. Hofmann C, Loser P, Cichon G, Arnold W, Both GW, Strauss M. Ovine adenovirus vectors overcome preexisting humoral immunity against human adenoviruses in vivo. J Virol **1999**;73:6930-6.

69. Kostense, S, Vogel, S., Lemckert, A., Ophorst, O., Koudstaal, W., Sprangers, M., Goudsmit, J., Havenga, M. Adenovirus serotype 35-based vaccination in the absence or presence of serotype 5 specific immunity. Keystone Conference on HIV Vaccines. Banff, **2003**.

70. Vogels R, Zuijdgeest D, van Rijnsoever R, et al. Replication-deficient human adenovirus type 35 vectors for gene transfer and vaccination: efficient human cell infection and bypass of preexisting adenovirus immunity. J Virol **2003**;77:8263-71.

71. Seshidhar RP, Ganesh S, Limbach MP, et al. Development of adenovirus serotype 35 as a gene transfer vector. Virology **2003**;311:384-93.

72. Schmitz H, Wigand R, Heinrich W. Worldwide epidemiology of human adenovirus infections. Am J Epidemiol **1983**;117:455-66.

73. Nwanegbo E, Gao W, Gambotto A, Rowe D, Robbins P, Sun H, Whittle H, Vardas E. Prevalence of neutralizing antibody to adenoviral serotype 5 and 35 in the Gambia, South Africa, and United States adult populations. AIDS Vaccine 2003. New York, **2003**.

74. Mast TC, Gupta S, Nikas A, Kierstead L, Kallas EG, Mbewe B, Pitisuttithum P, Schecter M, Vardas E, Wolfe N, Coplan P, Aste-Amezaga M, Shiver J, Emini EA, Straus WL. International epidemiology of human preexisting anti-adenovirus type-5 (Ad5) antibodies in areas representative of potential HIV vaccine trial sites. AIDS Vaccine 2003. Bangkok, Thailand, **2004**.

75. Chakrabarti BK, Kong WP, Wu BY, et al. Modifications of the human immunodeficiency virus envelope glycoprotein enhance immunogenicity for genetic immunization. J Virol **2002**;76:5357-68.

76. Williams J, Fox-Leyva L, Christensen C, et al. Hepatitis A vaccine administration: comparison between jet-injector and needle injection. Vaccine **2000**;18:1939-43.

77. Hoke CH, Jr., Egan JE, Sjogren MH, et al. Administration of hepatitis A vaccine to a military population by needle and jet injector and with hepatitis B vaccine. J Infect Dis **1995**;171 Suppl 1:S53-S60.

78. Manam S, Ledwith BJ, Barnum AB, et al. Plasmid DNA vaccines: tissue distribution and effects of DNA sequence, adjuvants and delivery method on integration into host DNA. Intervirology **2000**;43:273-81.

79. Wang R, Epstein J, Baraceros FM, et al. Induction of CD4(+) T cell-dependent CD8(+) type 1 responses in humans by a malaria DNA vaccine. Proc Natl Acad Sci U S A **2001**;98:10817-22.

80. Rogers WO, Baird JK, Kumar A, et al. Multistage multiantigen heterologous prime boost vaccine for Plasmodium knowlesi malaria provides partial protection in rhesus macaques. Infect Immun **2001**;69:5565-72.

81. Epstein JE, Gorak EJ, Charoenvit Y, et al. Safety, tolerability, and lack of antibody responses after administration of a PfCSP DNA malaria vaccine via needle or needle-free jet injection, and comparison of intramuscular and combination intramuscular/intradermal routes. Hum Gene Ther **2002**;13:1551-60.

82. Rogers WO, Weiss WR, Kumar A, et al. Protection of rhesus macaques against lethal Plasmodium knowlesi malaria by a heterologous DNA priming and poxvirus boosting immunization regimen. Infect Immun **2002**;70:4329-35.

83. Seth P. Adenoviruses: basic biology to gene therapy. Austin, TX: RG Landes Co., **1999**.

84. Brough DE, Lizonova A, Hsu C, Kulesa VA, Kovesdi I. A gene transfer vector-cell line system for complete functional complementation of adenovirus early regions E1 and E4. J Virol **1996**;70:6497-501.

APPENDIX A - SCHEDULE OF PROCEDURE FOR VOLUNTEERS RECEIVING DNA+rAd5 VACCINE (GROUPS C AND D)

	**Screen**																	**Final Visit/ET**
Study Month		**M0**			**M1**			**M2**		**M2.5**	**M3**	**M6**		**M6.5**	**M7**	**M7.5**	**M9**	**M12**
Study Week		W0		W2	W4		W6	W8		W10	W12	W24		W28	W28	W30	W36	W48
Study Day		0	3	14	28	31	42	56	59	70	84	168	171	182	196	210	252	336
Visit Windows (Days)		-42	±1	±2	±3	±1	±2	±3	±1	±2	±3	±3	±1	±2	±3	±2	±14	±14
Investigational Product/Placebo		X			X			X				X						
Local and Systemic Reactogenicity Assessment (pre-and post vaccination)		XX*	X	X	XX*	X	X	XX*	X	X	X	XX*	X	X				
Informed Consent	X																	
Screening Questionnaire	X																	
Medical History (including concomitant medications)	X	X	X	X	X	X	X	X	X	X	X	X	X	X	X		X	X
General Physical Exam	X																	X
Directed Physical Exam		X	X	X	X	X	X	X	X	X	X	X	X	X	X		X	
Adverse Events / Serious Adverse Events		X	X	X	X	X	X	X	X	X	X	X	X	X	X		X	X
Vital Signs (pre-and post vaccination)		X X*			XX*			XX*				XX*						
Hep BsAg, Syphilis	X																	
Hematology	X	X		X			X			X				X			X	X
Clinical Chemistry (+ serum storage)	X	X		X			X			X				X			X	X
Immunology (CD4, CD8)	X	X																
Urine dipstick	X						X							X				
HIV test Pre-/Post HIV-test Counseling	X	X						X				X					X	X
Family planning counselling	X	X									X	X					X	X
Pregnancy Test (all female volunteers)	X	X			X			X				X					X	X
PBMCs for Cellular immunogenicity assays (ELISPOT assay and ICS), Plasma + PBMC storage		X								X	X	X			X	X	X	X
Anti-rAd5 Antibody		X										X			X			X
HIV Antibody		X								X		X			X	X		X
HLA Typing**				X														
Total Blood Volume for Visit (mL)***	12/12	102/82		17/17			7/7			67/67	100/50	65/65		7/7	65/65	100/70	107/67	102/102
Cumulative Blood Volume (mL)***	12/12	114/94		131/111			138/118			205/185	305/235	370/300		377/307	422/372	522/422	629/509	751/611

ET = Early Termination * Post vaccination assessment will be done at 30 minutes post vaccination. ** To be drawn at either Week 2 or Week 6. ***Blood volumes expressed as volume collected at KAVI/volume collected at Kigali.

APPENDIX B - SCHEDULE OF PROCEDURE FOR VOLUNTEERS RECEIVING rAd5 VACCINE ONLY (GROUPS A AND B)

	**Screen**									**Final Visit/ET**
Study Month		**M0**			**M1**	**M1.5**	**M3**	**M6**	**M9**	**M12**
Study Week		W0		W2	W4	W6	W12	W24	W36	W48
Study Day		0	3	14	28	42	84	168	252	336
Visit Windows (Days)		-42	±1	±2	±3	±1	±3	±7	±14	±14
Investigational Product/Placebo		X								
Local and Systemic Reactogenicity Assessment (pre-and post vaccination)		XX*	X	X						
Informed Consent	X									
Screening Questionnaire	X									
Medical History (including concomitant medications)	X	X	X	X	X	X	X	X	X	X
General Physical Exam	X									X
Directed Physical Exam		X	X	X	X	X	X	X	X	
Adverse Events / Serious Adverse Events		X	X	X	X	X	X	X	X	X
Vital Signs (pre-and post vaccination)		X X*								X
Hep BsAg, Syphilis	X									
Hematology (+ plasma storage)	X	X		X				X	X	X
Clinical Chemistry (+ serum storage)	X	X		X				X	X	X
Immunology (CD4, CD8)	X	X								
Urine dipstick	X			X				X		
HIV test<br>Pre-/Post HIV-test Counseling	X	X					X	X	X	X
Family planning counselling	X	X					X	X	X	X
Pregnancy Test (all female volunteers)	X	X					X	X	X	X
PBMCs for Cellular immunogenicity assays (ELISPOT assay and ICS) + PBMC storage		X			X	X			X	X
Anti-rAd5 Antibody		X			X					X
HIV Antibody		X		X	X					X
HLA Typing**				X						
Total Blood Volume for Visit (mL)***	12/12	102/82		27/27	65/65	50/50	5/5	12/12	60/60	102/102
Cumulative Blood Volume (mL)***	12/12	114/100		141/127	206/192	256/242	261/247	273/259	333/319	435/421

ET = Early Termination

* Post vaccination assessment will be done at 30 minutes post vaccination ** To be drawn at either Week 2 or Week 6. ***Blood volumes expressed as volume collected at KAVI/volume collected at Kigali.

APPENDIX C - ADVERSE EVENT GRADING TOXICITY TABLE

DIVISION OF AIDS TABLE FOR GRADING THE SEVERITY OF ADULT AND PEDIATRIC ADVERSE EVENTS PUBLISH DATE: DECEMBER, 2004

Quick Reference

The Division of AIDS Table for Grading the Severity of Adult and Pediatric Adverse Events ("DAIDS AE grading table") is a descriptive terminology which can be utilized for Adverse Event (AE) reporting. A grading (severity) scale is provided for each AE term.

General Instructions

Estimating Severity Grade

If the need arises to grade a clinical AE that is not identified in the DAIDS AE grading table, use the category "Estimating Severity Grade" located at the top of Page 3. For AEs that are not listed in the table but will be collected systematically for a study/trial, protocol teams are highly encouraged to define study-specific severity scales within the protocol or an appendix to the protocol. (Please see "Template Wording for the Expedited Adverse Event Reporting Section of DAIDS-sponsored Protocols".) This is particularly important for laboratory values because the "Estimating Severity Grade" category only applies to clinical symptoms.

Grading Adult and Pediatric AEs

The DAIDS AE grading table includes parameters for grading both Adult and Pediatric AEs. When a single set of parameters is not appropriate for grading specific types of AEs for both Adult and Pediatric populations, separate sets of parameters for Adult and/or Pediatric populations (with specified respective age ranges) are given in the table. If there is no distinction in the table between Adult and Pediatric values for a type of AE, then the single set of parameters listed is to be used for grading the severity of both Adult and Pediatric events of that type.

Determining Severity Grade

If the severity of an AE could fall under either one of two grades (e.g., the severity of an AE could be either Grade 2 or Grade 3), select the higher of the two grades for the AE.

Definitions

Basic Self-care Functions	Adult<br>Activities such as bathing, dressing, toileting, transfer/movement, continence, and feeding.<br>Young Children<br>Activities that are age and culturally appropriate (e.g., feeding self with culturally appropriate eating implement).
LLN	Lower limit of normal
Medical Intervention	Use of pharmacologic or biologic agent(s) for treatment of an AE.
NA	Not Applicable
Operative Intervention	Surgical OR other invasive mechanical procedures.
ULN	Upper limit of normal
Usual Social & Functional Activities	Adult<br>Adaptive tasks and desirable activities, such as going to work, shopping, cooking, use of transportation, pursuing a hobby, etc.<br>Young Children<br>Activities that are age and culturally appropriate (e.g., social interactions, play activities, learning tasks, etc.).

DIVISION OF AIDS TABLE FOR GRADING THE SEVERITY OF ADULT AND PEDIATRIC ADVERSE EVENTS
PUBLISH DATE: DECEMBER, 2004

Contents

Clinical	Page
Estimating Severity Grade	3
Systemic	3
Infection	4
Injection Site Reactions	4
Skin – Dermatological	5
Cardiovascular	5
Gastrointestinal	7
Neurologic	9
Respiratory	12
Musculoskeletal	12
Genitourinary	13
Ocular/Visual	14
Endocrine/Metabolic	14

Laboratory	Page
Hematology	16
Chemistries	17
Urinalysis	20

DIVISION OF AIDS TABLE FOR GRADING THE SEVERITY OF ADULT AND PEDIATRIC ADVERSE EVENTS
PUBLISH DATE: DECEMBER, 2004

CLINICAL				
**PARAMETER**	**GRADE 1 MILD**	**GRADE 2 MODERATE**	**GRADE 3 SEVERE**	**GRADE 4 POTENTIALLY LIFE-THREATENING**
**ESTIMATING SEVERITY GRADE**				
Clinical adverse event NOT identified elsewhere in this DAIDS AE grading table	Symptoms causing no or minimal interference with usual social & functional activities	Symptoms causing greater than minimal interference with usual social & functional activities	Symptoms causing inability to perform usual social & functional activities	Symptoms causing inability to perform basic self-care functions OR Medical or operative intervention indicated to prevent permanent impairment, persistent disability, or death
**SYSTEMIC**				
Acute systemic allergic reaction	Localized urticaria (wheals) with no medical intervention indicated	Localized urticaria with medical intervention indicated OR Mild angioedema with no medical intervention indicated	Generalized urticaria OR Angioedema with medical intervention indicated OR Symptomatic mild bronchospasm	Acute anaphylaxis OR Life-threatening bronchospasm OR laryngeal edema
Chills	Symptoms causing no or minimal interference with usual social & functional activities	Symptoms causing greater than minimal interference with usual social & functional activities	Symptoms causing inability to perform usual social & functional activities	NA
Fatigue<br>Malaise	Symptoms causing no or minimal interference with usual social & functional activities	Symptoms causing greater than minimal interference with usual social & functional activities	Symptoms causing inability to perform usual social & functional activities	Incapacitating fatigue/ malaise symptoms causing inability to perform basic self-care functions
Fever (nonaxillary)	37.7 – 38.6°C	38.7 – 39.3°C	39.4 – 40.5°C	> 40.5°C
Pain (indicate body site)<br>DO NOT use for pain due to injection (See Injection Site Reactions: Injection site pain)<br>See also Headache, Arthralgia, and Myalgia	Pain causing no or minimal interference with usual social & functional activities	Pain causing greater than minimal interference with usual social & functional activities	Pain causing inability to perform usual social & functional activities	Disabling pain causing inability to perform basic self-care functions OR Hospitalization (other than emergency room visit) indicated

Basic Self-care Functions – Adult: Activities such as bathing, dressing, toileting, transfer/movement, continence, and feeding.

Basic Self-care Functions – Young Children: Activities that are age and culturally appropriate (e.g., feeding self with culturally appropriate eating implement).

Usual Social & Functional Activities – Adult: Adaptive tasks and desirable activities, such as going to work, shopping, cooking, use of transportation, pursuing a hobby, etc.

Usual Social & Functional Activities – Young Children: Activities that are age and culturally appropriate (e.g., social interactions, play activities, learning tasks, etc.).

DIVISION OF AIDS TABLE FOR GRADING THE SEVERITY OF ADULT AND PEDIATRIC ADVERSE EVENTS
PUBLISH DATE: DECEMBER, 2004

CLINICAL				
**PARAMETER**	**GRADE 1 MILD**	**GRADE 2 MODERATE**	**GRADE 3 SEVERE**	**GRADE 4 POTENTIALLY LIFE-THREATENING**
Unintentional weight loss	NA	5 – 9% loss in body weight from baseline	10 – 19% loss in body weight from baseline	≥ 20% loss in body weight from baseline OR Aggressive intervention indicated [e.g., tube feeding or total parenteral nutrition (TPN)]
**INFECTION**				
Infection (any other than HIV infection)	Localized, no systemic antimicrobial treatment indicated AND Symptoms causing no or minimal interference with usual social & functional activities	Systemic antimicrobial treatment indicated OR Symptoms causing greater than minimal interference with usual social & functional activities	Systemic antimicrobial treatment indicated AND Symptoms causing inability to perform usual social & functional activities OR Operative intervention (other than simple incision and drainage) indicated	Life-threatening consequences (e.g., septic shock)
**INJECTION SITE REACTIONS**				
Injection site pain (pain without touching)<br>Or<br>Tenderness (pain when area is touched)	Pain/tenderness causing no or minimal limitation of use of limb	Pain/tenderness limiting use of limb OR Pain/tenderness causing greater than minimal interference with usual social & functional activities	Pain/tenderness causing inability to perform usual social & functional activities	Pain/tenderness causing inability to perform basic self-care function OR Hospitalization (other than emergency room visit) indicated for management of pain/tenderness
Injection site reaction (localized)				
**Adult > 15 years**	Erythema OR Induration of 5x5 cm – 9x9 cm (or 25 cm^2 – 81 cm^2)	Erythema OR Induration OR Edema > 9 cm any diameter (or > 81 cm^2)	Ulceration OR Secondary infection OR Phlebitis OR Sterile abscess OR Drainage	Necrosis (involving dermis and deeper tissue)
**Pediatric ≤ 15 years**	Erythema OR Induration OR Edema present but ≤ 2.5 cm diameter	Erythema OR Induration OR Edema > 2.5 cm diameter but < 50% surface area of the extremity segment (e.g., upper arm/thigh)	Erythema OR Induration OR Edema involving ≥ 50% surface area of the extremity segment (e.g., upper arm/thigh) OR Ulceration OR Secondary infection OR Phlebitis OR Sterile abscess OR Drainage	Necrosis (involving dermis and deeper tissue)

Basic Self-care Functions – Adult: Activities such as bathing, dressing, toileting, transfer/movement, continence, and feeding.

Basic Self-care Functions – Young Children: Activities that are age and culturally appropriate (e.g., feeding self with culturally appropriate eating implement).

Usual Social & Functional Activities – Adult: Adaptive tasks and desirable activities, such as going to work, shopping, cooking, use of transportation, pursuing a hobby, etc.

Usual Social & Functional Activities – Young Children: Activities that are age and culturally appropriate (e.g., social interactions, play activities, learning tasks, etc.).

DIVISION OF AIDS TABLE FOR GRADING THE SEVERITY OF ADULT AND PEDIATRIC ADVERSE EVENTS PUBLISH DATE: DECEMBER, 2004

CLINICAL				
**PARAMETER**	**GRADE 1 MILD**	**GRADE 2 MODERATE**	**GRADE 3 SEVERE**	**GRADE 4 POTENTIALLY LIFE-THREATENING**
Pruritis associated with injection<br>See also Skin: Pruritis (itching - no skin lesions)	Itching localized to injection site AND Relieved spontaneously or with < 48 hours treatment	Itching beyond the injection site but not generalized OR Itching localized to injection site requiring ≥ 48 hours treatment	Generalized itching causing inability to perform usual social & functional activities	NA
**SKIN – DERMATOLOGICAL**				
Alopecia	Thinning detectable by study participant (or by caregiver for young children and disabled adults)	Thinning or patchy hair loss detectable by health care provider	Complete hair loss	NA
Cutaneous reaction – rash	Localized macular rash	Diffuse macular, maculopapular, or morbilliform rash OR Target lesions	Diffuse macular, maculopapular, or morbilliform rash with vesicles or limited number of bullae OR Superficial ulcerations of mucous membrane limited to one site	Extensive or generalized bullous lesions OR Stevens-Johnson syndrome OR Ulceration of mucous membrane involving two or more distinct mucosal sites OR Toxic epidermal necrolysis (TEN)
Hyperpigmentation	Slight or localized	Marked or generalized	NA	NA
Hypopigmentation	Slight or localized	Marked or generalized	NA	NA
Pruritis (itching – no skin lesions)<br>(See also Injection Site Reactions: Pruritis associated with injection)	Itching causing no or minimal interference with usual social & functional activities	Itching causing greater than minimal interference with usual social & functional activities	Itching causing inability to perform usual social & functional activities	NA
**CARDIOVASCULAR**				
Cardiac arrhythmia (general)<br>(By ECG or physical exam)	Asymptomatic AND No intervention indicated	Asymptomatic AND Non-urgent medical intervention indicated	Symptomatic, non-life-threatening AND Non-urgent medical intervention indicated	Life-threatening arrhythmia OR Urgent intervention indicated
Cardiac-ischemia/infarction	NA	NA	Symptomatic ischemia (stable angina) OR Testing consistent with ischemia	Unstable angina OR Acute myocardial infarction

Basic Self-care Functions – Adult: Activities such as bathing, dressing, toileting, transfer/movement, continence, and feeding.

Basic Self-care Functions – Young Children: Activities that are age and culturally appropriate (e.g., feeding self with culturally appropriate eating implement).

Usual Social & Functional Activities – Adult: Adaptive tasks and desirable activities, such as going to work, shopping, cooking, use of transportation, pursuing a hobby, etc.

Usual Social & Functional Activities – Young Children: Activities that are age and culturally appropriate (e.g., social interactions, play activities, learning tasks, etc.).

DIVISION OF AIDS TABLE FOR GRADING THE SEVERITY OF ADULT AND PEDIATRIC ADVERSE EVENTS PUBLISH DATE: DECEMBER, 2004

CLINICAL				
**PARAMETER**	**GRADE 1 MILD**	**GRADE 2 MODERATE**	**GRADE 3 SEVERE**	**GRADE 4 POTENTIALLY LIFE-THREATENING**
Hemorrhage (significant acute blood loss)	NA	Symptomatic AND No transfusion indicated	Symptomatic AND Transfusion of ≤ 2 units packed RBCs (for children ≤ 10 cc/kg) indicated	Life-threatening hypotension OR Transfusion of > 2 units packed RBCs (for children > 10 cc/kg) indicated
Hypertension				
**Adult > 17 years** (with repeat testing at same visit)	> 140 – 159 mmHg systolic OR > 90 – 99 mmHg diastolic	> 160 – 179 mmHg systolic OR > 100 – 109 mmHg diastolic	> 180 mmHg systolic OR > 110 mmHg diastolic	Life-threatening consequences (e.g., malignant hypertension) OR Hospitalization indicated (other than emergency room visit)
**Pediatric ≤ 17 years** (with repeat testing at same visit)	NA	91st – 94th percentile adjusted for age, height, and gender (systolic and/or diastolic)	≥ 95th percentile adjusted for age, height, and gender (systolic and/or diastolic)	Life-threatening consequences (e.g., malignant hypertension) OR Hospitalization indicated (other than emergency room visit)
Hypotension	NA	Symptomatic, corrected with oral fluid replacement	Symptomatic, IV fluids indicated	Shock requiring use of vasopressors or mechanical assistance to maintain blood pressure
Pericardial effusion	Asymptomatic, small effusion requiring no intervention	Asymptomatic, moderate or larger effusion requiring no intervention	Effusion with non-life threatening physiologic consequences OR Effusion with non-urgent intervention indicated	Life-threatening consequences (e.g., tamponade) OR Urgent intervention indicated
Prolonged PR interval				
**Adult > 16 years**	PR interval 0.21 – 0.25 sec	PR interval > 0.25 sec	Type II 2nd degree AV block OR Ventricular pause > 3.0 sec	Complete AV block
**Pediatric ≤ 16 years**	1st degree AV block (PR > normal for age and rate)	Type I 2nd degree AV block	Type II 2nd degree AV block	Complete AV block

Basic Self-care Functions – Adult: Activities such as bathing, dressing, toileting, transfer/movement, continence, and feeding.

Basic Self-care Functions – Young Children: Activities that are age and culturally appropriate (e.g., feeding self with culturally appropriate eating implement).

Usual Social & Functional Activities – Adult: Adaptive tasks and desirable activities, such as going to work, shopping, cooking, use of transportation, pursuing a hobby, etc.

Usual Social & Functional Activities – Young Children: Activities that are age and culturally appropriate (e.g., social interactions, play activities, learning tasks, etc.).

DIVISION OF AIDS TABLE FOR GRADING THE SEVERITY OF ADULT AND PEDIATRIC ADVERSE EVENTS PUBLISH DATE: DECEMBER, 2004

PARAMETER	GRADE 1 MILD	GRADE 2 MODERATE	GRADE 3 SEVERE	GRADE 4 POTENTIALLY LIFE-THREATENING
**CLINICAL**				
Prolonged QTc				
**Adult > 16 years**	Asymptomatic, QTc interval 0.45 – 0.47 sec OR Increase interval < 0.03 sec above baseline	Asymptomatic, QTc interval 0.48 – 0.49 sec OR Increase in interval 0.03 – 0.05 sec above baseline	Asymptomatic, QTc interval ≥ 0.50 sec OR Increase in interval ≥ 0.06 sec above baseline	Life-threatening consequences, e.g. Torsade de pointes or other associated serious ventricular dysrhythmia
**Pediatric ≤ 16 years**	Asymptomatic, QTc interval 0.450 – 0.464 sec	Asymptomatic, QTc interval 0.465 – 0.479 sec	Asymptomatic, QTc interval ≥ 0.480 sec	Life-threatening consequences, e.g. Torsade de pointes or other associated serious ventricular dysrhythmia
Thrombosis/embolism	NA	Deep vein thrombosis AND No intervention indicated (e.g., anticoagulation, lysis filter, invasive procedure)	Deep vein thrombosis AND Intervention indicated (e.g., anticoagulation, lysis filter, invasive procedure)	Embolic event (e.g., pulmonary embolism, life-threatening thrombus)
Vasovagal episode (associated with a procedure of any kind)	Present without loss of consciousness	Present with transient loss of consciousness	NA	NA
Ventricular dysfunction (congestive heart failure)	NA	Asymptomatic diagnostic finding AND intervention indicated	New onset with symptoms OR Worsening symptomatic congestive heart failure	Life-threatening congestive heart failure
**GASTROINTESTINAL**				
Anorexia	Loss of appetite without decreased oral intake	Loss of appetite associated with decreased oral intake without significant weight loss	Loss of appetite associated with significant weight loss	Life-threatening consequences OR Aggressive intervention indicated [e.g., tube feeding or total parenteral nutrition (TPN)]
Ascites	Asymptomatic	Symptomatic AND Intervention indicated (e.g., diuretics or therapeutic paracentesis)	Symptomatic despite intervention	Life-threatening consequences

Basic Self-care Functions – Adult: Activities such as bathing, dressing, toileting, transfer/movement, continence, and feeding.

Basic Self-care Functions – Young Children: Activities that are age and culturally appropriate (e.g., feeding self with culturally appropriate eating implement).

Usual Social & Functional Activities – Adult: Adaptive tasks and desirable activities, such as going to work, shopping, cooking, use of transportation, pursuing a hobby, etc.

Usual Social & Functional Activities – Young Children: Activities that are age and culturally appropriate (e.g., social interactions, play activities, learning tasks, etc.).

DIVISION OF AIDS TABLE FOR GRADING THE SEVERITY OF ADULT AND PEDIATRIC ADVERSE EVENTS PUBLISH DATE: DECEMBER, 2004

CLINICAL				
**PARAMETER**	**GRADE 1 MILD**	**GRADE 2 MODERATE**	**GRADE 3 SEVERE**	**GRADE 4 POTENTIALLY LIFE-THREATENING**
Cholecystitis	NA	Symptomatic AND Medical intervention indicated	Radiologic, endoscopic, or operative intervention indicated	Life-threatening consequences (e.g., sepsis or perforation)
Constipation	NA	Persistent constipation requiring regular use of dietary modifications, laxatives, or enemas	Obstipation with manual evacuation indicated	Life-threatening consequences (e.g., obstruction)
Diarrhea				
**Adult and Pediatric ≥ 1 year**	Transient or intermittent episodes of unformed stools OR Increase of ≤ 3 stools over baseline per 24-hour period	Persistent episodes of unformed to watery stools OR Increase of 4 – 6 stools over baseline per 24-hour period	Bloody diarrhea OR Increase of ≥ 7 stools per 24-hour period OR IV fluid replacement indicated	Life-threatening consequences (e.g., hypotensive shock)
**Pediatric < 1 year**	Liquid stools (more unformed than usual) but usual number of stools	Liquid stools with increased number of stools OR Mild dehydration	Liquid stools with moderate dehydration	Liquid stools resulting in severe dehydration with aggressive rehydration indicated OR Hypotensive shock
Dysphagia-Odynophagia	Symptomatic but able to eat usual diet	Symptoms causing altered dietary intake without medical intervention indicated	Symptoms causing severely altered dietary intake with medical intervention indicated	Life-threatening reduction in oral intake
Mucositis/stomatitis (clinical exam)<br>Indicate site (e.g., larynx, oral)<br>See Genitourinary for Vulvovaginitis<br>See also Dysphagia-Odynophagia and Proctitis	Erythema of the mucosa	Patchy pseudomembranes or ulcerations	Confluent pseudomembranes or ulcerations OR Mucosal bleeding with minor trauma	Tissue necrosis OR Diffuse spontaneous mucosal bleeding OR Life-threatening consequences (e.g., aspiration, choking)
Nausea	Transient (< 24 hours) or intermittent nausea with no or minimal interference with oral intake	Persistent nausea resulting in decreased oral intake for 24 – 48 hours	Persistent nausea resulting in minimal oral intake for > 48 hours OR Aggressive rehydration indicated (e.g., IV fluids)	Life-threatening consequences (e.g., hypotensive shock)

Basic Self-care Functions – Adult: Activities such as bathing, dressing, toileting, transfer/movement, continence, and feeding.

Basic Self-care Functions – Young Children: Activities that are age and culturally appropriate (e.g., feeding self with culturally appropriate eating implement).

Usual Social & Functional Activities – Adult: Adaptive tasks and desirable activities, such as going to work, shopping, cooking, use of transportation, pursuing a hobby, etc.

Usual Social & Functional Activities – Young Children: Activities that are age and culturally appropriate (e.g., social interactions, play activities, learning tasks, etc.).

DIVISION OF AIDS TABLE FOR GRADING THE SEVERITY OF ADULT AND PEDIATRIC ADVERSE EVENTS PUBLISH DATE: DECEMBER, 2004

CLINICAL				
**PARAMETER**	**GRADE 1 MILD**	**GRADE 2 MODERATE**	**GRADE 3 SEVERE**	**GRADE 4 POTENTIALLY LIFE-THREATENING**
Pancreatitis	NA	Symptomatic AND Hospitalization not indicated (other than emergency room visit)	Symptomatic AND Hospitalization indicated (other than emergency room visit)	Life-threatening consequences (e.g., circulatory failure, hemorrhage, sepsis)
Proctitis (functional-symptomatic) Also see Mucositis/stomatitis for clinical exam	Rectal discomfort AND No intervention indicated	Symptoms causing greater than minimal interference with usual social & functional activities OR Medical intervention indicated	Symptoms causing inability to perform usual social & functional activities OR Operative intervention indicated	Life-threatening consequences (e.g., perforation)
Vomiting	Transient or intermittent vomiting with no or minimal interference with oral intake	Frequent episodes of vomiting with no or mild dehydration	Persistent vomiting resulting in orthostatic hypotension OR Aggressive rehydration indicated (e.g., IV fluids)	Life-threatening consequences (e.g., hypotensive shock)
**NEUROLOGIC**				
Alteration in personality-behavior or in mood (e.g., agitation, anxiety, depression, mania, psychosis)	Alteration causing no or minimal interference with usual social & functional activities	Alteration causing greater than minimal interference with usual social & functional activities	Alteration causing inability to perform usual social & functional activities	Behavior potentially harmful to self or others (e.g., suicidal and homicidal ideation or attempt, acute psychosis) OR Causing inability to perform basic self-care functions
Altered Mental Status For Dementia, see Cognitive and behavioral/attentional disturbance (including dementia and attention deficit disorder)	Changes causing no or minimal interference with usual social & functional activities	Mild lethargy or somnolence causing greater than minimal interference with usual social & functional activities	Confusion, memory impairment, lethargy, or somnolence causing inability to perform usual social & functional activities	Delirium OR obtundation, OR coma
Ataxia	Asymptomatic ataxia detectable on exam OR Minimal ataxia causing no or minimal interference with usual social & functional activities	Symptomatic ataxia causing greater than minimal interference with usual social & functional activities	Symptomatic ataxia causing inability to perform usual social & functional activities	Disabling ataxia causing inability to perform basic self-care functions

Basic Self-care Functions – Adult: Activities such as bathing, dressing, toileting, transfer/movement, continence, and feeding.

Basic Self-care Functions – Young Children: Activities that are age and culturally appropriate (e.g., feeding self with culturally appropriate eating implement).

Usual Social & Functional Activities – Adult: Adaptive tasks and desirable activities, such as going to work, shopping, cooking, use of transportation, pursuing a hobby, etc.

Usual Social & Functional Activities – Young Children: Activities that are age and culturally appropriate (e.g., social interactions, play activities, learning tasks, etc.).

DIVISION OF AIDS TABLE FOR GRADING THE SEVERITY OF ADULT AND PEDIATRIC ADVERSE EVENTS PUBLISH DATE: DECEMBER, 2004

CLINICAL				
**PARAMETER**	**GRADE 1 MILD**	**GRADE 2 MODERATE**	**GRADE 3 SEVERE**	**GRADE 4 POTENTIALLY LIFE-THREATENING**
Cognitive and behavioral/attentional disturbance (including dementia and attention deficit disorder)	Disability causing no or minimal interference with usual social & functional activities OR Specialized resources not indicated	Disability causing greater than minimal interference with usual social & functional activities OR Specialized resources on part-time basis indicated	Disability causing inability to perform usual social & functional activities OR Specialized resources on a full-time basis indicated	Disability causing inability to perform basic self-care functions OR Institutionalization indicated
CNS ischemia (acute)	NA	NA	Transient ischemic attack	Cerebral vascular accident (CVA, stroke) with neurological deficit
Developmental delay – **Pediatric ≤ 16 years**	Mild developmental delay, either motor or cognitive, as determined by comparison with a developmental screening tool appropriate for the setting	Moderate developmental delay, either motor or cognitive, as determined by comparison with a developmental screening tool appropriate for the setting	Severe developmental delay, either motor or cognitive, as determined by comparison with a developmental screening tool appropriate for the setting	Developmental regression, either motor or cognitive, as determined by comparison with a developmental screening tool appropriate for the setting
Headache	Symptoms causing no or minimal interference with usual social & functional activities	Symptoms causing greater than minimal interference with usual social & functional activities	Symptoms causing inability to perform usual social & functional activities	Symptoms causing inability to perform basic self-care functions OR Hospitalization indicated (other than emergency room visit) OR Headache with significant impairment of alertness or other neurologic function
Insomnia	NA	Difficulty sleeping causing greater than minimal interference with usual social & functional activities	Difficulty sleeping causing inability to perform usual social & functional activities	Disabling insomnia causing inability to perform basic self-care functions
Neuromuscular weakness (including myopathy & neuropathy)	Asymptomatic with decreased strength on exam OR Minimal muscle weakness causing no or minimal interference with usual social & functional activities	Muscle weakness causing greater than minimal interference with usual social & functional activities	Muscle weakness causing inability to perform usual social & functional activities	Disabling muscle weakness causing inability to perform basic self-care functions OR Respiratory muscle weakness impairing ventilation

Basic Self-care Functions – Adult: Activities such as bathing, dressing, toileting, transfer/movement, continence, and feeding.

Basic Self-care Functions – Young Children: Activities that are age and culturally appropriate (e.g., feeding self with culturally appropriate eating implement).

Usual Social & Functional Activities – Adult: Adaptive tasks and desirable activities, such as going to work, shopping, cooking, use of transportation, pursuing a hobby, etc.

Usual Social & Functional Activities – Young Children: Activities that are age and culturally appropriate (e.g., social interactions, play activities, learning tasks, etc.).

DIVISION OF AIDS TABLE FOR GRADING THE SEVERITY OF ADULT AND PEDIATRIC ADVERSE EVENTS PUBLISH DATE: DECEMBER, 2004

CLINICAL				
**PARAMETER**	**GRADE 1 MILD**	**GRADE 2 MODERATE**	**GRADE 3 SEVERE**	**GRADE 4 POTENTIALLY LIFE-THREATENING**
Neurosensory alteration (including paresthesia and painful neuropathy)	Asymptomatic with sensory alteration on exam or minimal paresthesia causing no or minimal interference with usual social & functional activities	Sensory alteration or paresthesia causing greater than minimal interference with usual social & functional activities	Sensory alteration or paresthesia causing inability to perform usual social & functional activities	Disabling sensory alteration or paresthesia causing inability to perform basic self-care functions
Seizure: (new onset) **– Adult ≥ 18 years**<br>See also Seizure: (known pre-existing seizure disorder)	NA	1 seizure	2 – 4 seizures	Seizures of any kind which are prolonged, repetitive (e.g., status epilepticus), or difficult to control (e.g., refractory epilepsy)
Seizure: (known pre-existing seizure disorder) **– Adult ≥ 18 years**<br>For worsening of existing epilepsy the grades should be based on an increase from previous level of control to any of these levels.	NA	Increased frequency of pre-existing seizures (non-repetitive) without change in seizure character OR Infrequent break-through seizures while on stable medication in a previously controlled seizure disorder	Change in seizure character from baseline either in duration or quality (e.g., severity or focality)	Seizures of any kind which are prolonged, repetitive (e.g., status epilepticus), or difficult to control (e.g., refractory epilepsy)
Seizure **– Pediatric < 18 years**	Seizure, generalized onset with or without secondary generalization, lasting < 5 minutes with < 24 hours post ictal state	Seizure, generalized onset with or without secondary generalization, lasting 5 – 20 minutes with < 24 hours post ictal state	Seizure, generalized onset with or without secondary generalization, lasting > 20 minutes	Seizure, generalized onset with or without secondary generalization, requiring intubation and sedation
Syncope (not associated with a procedure)	NA	Present	NA	NA
Vertigo	Vertigo causing no or minimal interference with usual social & functional activities	Vertigo causing greater than minimal interference with usual social & functional activities	Vertigo causing inability to perform usual social & functional activities	Disabling vertigo causing inability to perform basic self-care functions

Basic Self-care Functions – Adult: Activities such as bathing, dressing, toileting, transfer/movement, continence, and feeding.

Basic Self-care Functions – Young Children: Activities that are age and culturally appropriate (e.g., feeding self with culturally appropriate eating implement).

Usual Social & Functional Activities – Adult: Adaptive tasks and desirable activities, such as going to work, shopping, cooking, use of transportation, pursuing a hobby, etc.

Usual Social & Functional Activities – Young Children: Activities that are age and culturally appropriate (e.g., social interactions, play activities, learning tasks, etc.).

DIVISION OF AIDS TABLE FOR GRADING THE SEVERITY OF ADULT AND PEDIATRIC ADVERSE EVENTS PUBLISH DATE: DECEMBER, 2004

CLINICAL				
**PARAMETER**	**GRADE 1 MILD**	**GRADE 2 MODERATE**	**GRADE 3 SEVERE**	**GRADE 4 POTENTIALLY LIFE-THREATENING**
**RESPIRATORY**				
Bronchospasm (acute)	FEV1 or peak flow reduced to 70 – 80%	FEV1 or peak flow 50 – 69%	FEV1 or peak flow 25 – 49%	Cyanosis OR FEV1 or peak flow < 25% OR Intubation
Dyspnea or respiratory distress				
**Adult ≥ 14 years**	Dyspnea on exertion with no or minimal interference with usual social & functional activities	Dyspnea on exertion causing greater than minimal interference with usual social & functional activities	Dyspnea at rest causing inability to perform usual social & functional activities	Respiratory failure with ventilatory support indicated
**Pediatric < 14 years**	Wheezing OR minimal increase in respiratory rate for age	Nasal flaring OR Intercostal retractions OR Pulse oximetry 90 – 95%	Dyspnea at rest causing inability to perform usual social & functional activities OR Pulse oximetry < 90%	Respiratory failure with ventilatory support indicated
**MUSCULOSKELETAL**				
Arthralgia<br>See also Arthritis	Joint pain causing no or minimal interference with usual social & functional activities	Joint pain causing greater than minimal interference with usual social & functional activities	Joint pain causing inability to perform usual social & functional activities	Disabling joint pain causing inability to perform basic self-care functions
Arthritis<br>See also Arthralgia	Stiffness or joint swelling causing no or minimal interference with usual social & functional activities	Stiffness or joint swelling causing greater than minimal interference with usual social & functional activities	Stiffness or joint swelling causing inability to perform usual social & functional activities	Disabling joint stiffness or swelling causing inability to perform basic self-care functions
Bone Mineral Loss				
**Adult ≥ 21 years**	BMD t-score -2.5 to -1.0	BMD t-score < -2.5	Pathological fracture (including loss of vertebral height)	Pathologic fracture causing life-threatening consequences
**Pediatric < 21 years**	BMD z-score -2.5 to -1.0	BMD z-score < -2.5	Pathological fracture (including loss of vertebral height)	Pathologic fracture causing life-threatening consequences

Basic Self-care Functions – Adult: Activities such as bathing, dressing, toileting, transfer/movement, continence, and feeding.

Basic Self-care Functions – Young Children: Activities that are age and culturally appropriate (e.g., feeding self with culturally appropriate eating implement).

Usual Social & Functional Activities – Adult: Adaptive tasks and desirable activities, such as going to work, shopping, cooking, use of transportation, pursuing a hobby, etc.

Usual Social & Functional Activities – Young Children: Activities that are age and culturally appropriate (e.g., social interactions, play activities, learning tasks, etc.).

DIVISION OF AIDS TABLE FOR GRADING THE SEVERITY OF ADULT AND PEDIATRIC ADVERSE EVENTS PUBLISH DATE: DECEMBER, 2004

CLINICAL				
**PARAMETER**	**GRADE 1 MILD**	**GRADE 2 MODERATE**	**GRADE 3 SEVERE**	**GRADE 4 POTENTIALLY LIFE-THREATENING**
Myalgia (non-injection site)	Muscle pain causing no or minimal interference with usual social & functional activities	Muscle pain causing greater than minimal interference with usual social & functional activities	Muscle pain causing inability to perform usual social & functional activities	Disabling muscle pain causing inability to perform basic self-care functions
Osteonecrosis	NA	Asymptomatic with radiographic findings AND No operative intervention indicated	Symptomatic bone pain with radiographic findings OR Operative intervention indicated	Disabling bone pain with radiographic findings causing inability to perform basic self-care functions
**GENITOURINARY**				
Cervicitis (symptoms)<br>(For use in studies evaluating topical study agents)<br>For other cervicitis see Infection: Infection (any other than HIV infection)	Symptoms causing no or minimal interference with usual social & functional activities	Symptoms causing greater than minimal interference with usual social & functional activities	Symptoms causing inability to perform usual social & functional activities	Symptoms causing inability to perform basic self-care functions
Cervicitis (clinical exam)<br>(For use in studies evaluating topical study agents)<br>For other cervicitis see Infection: Infection (any other than HIV infection)	Minimal cervical abnormalities on examination (erythema, mucopurulent discharge, or friability) OR Epithelial disruption < 25% of total surface	Moderate cervical abnormalities on examination (erythema, mucopurulent discharge, or friability) OR Epithelial disruption of 25 – 49% total surface	Severe cervical abnormalities on examination (erythema, mucopurulent discharge, or friability) OR Epithelial disruption 50 – 75% total surface	Epithelial disruption > 75% total surface
Inter-menstrual bleeding (IMB)	Spotting observed by participant OR Minimal blood observed during clinical or colposcopic examination	Inter-menstrual bleeding not greater in duration or amount than usual menstrual cycle	Inter-menstrual bleeding greater in duration or amount than usual menstrual cycle	Hemorrhage with life-threatening hypotension OR Operative intervention indicated
Urinary tract obstruction (e.g., stone)	NA	Signs or symptoms of urinary tract obstruction without hydronephrosis or renal dysfunction	Signs or symptoms of urinary tract obstruction with hydronephrosis or renal dysfunction	Obstruction causing life-threatening consequences

Basic Self-care Functions – Adult: Activities such as bathing, dressing, toileting, transfer/movement, continence, and feeding.

Basic Self-care Functions – Young Children: Activities that are age and culturally appropriate (e.g., feeding self with culturally appropriate eating implement).

Usual Social & Functional Activities – Adult: Adaptive tasks and desirable activities, such as going to work, shopping, cooking, use of transportation, pursuing a hobby, etc.

Usual Social & Functional Activities – Young Children: Activities that are age and culturally appropriate (e.g., social interactions, play activities, learning tasks, etc.).

DIVISION OF AIDS TABLE FOR GRADING THE SEVERITY OF ADULT AND PEDIATRIC ADVERSE EVENTS PUBLISH DATE: DECEMBER, 2004

CLINICAL				
**PARAMETER**	**GRADE 1 MILD**	**GRADE 2 MODERATE**	**GRADE 3 SEVERE**	**GRADE 4 POTENTIALLY LIFE-THREATENING**
Vulvovaginitis (symptoms) (Use in studies evaluating topical study agents) For other vulvovaginitis see Infection: Infection (any other than HIV infection)	Symptoms causing no or minimal interference with usual social & functional activities	Symptoms causing greater than minimal interference with usual social & functional activities	Symptoms causing inability to perform usual social & functional activities	Symptoms causing inability to perform basic self-care functions
Vulvovaginitis (clinical exam) (Use in studies evaluating topical study agents) For other vulvovaginitis see Infection: Infection (any other than HIV infection)	Minimal vaginal abnormalities on examination OR Epithelial disruption < 25% of total surface	Moderate vaginal abnormalities on examination OR Epithelial disruption of 25 - 49% total surface	Severe vaginal abnormalities on examination OR Epithelial disruption 50 - 75% total surface	Vaginal perforation OR Epithelial disruption > 75% total surface
**OCULAR/VISUAL**				
Uveitis	Asymptomatic but detectable on exam	Symptomatic anterior uveitis OR Medical intervention indicated	Posterior or pan-uveitis OR Operative intervention indicated	Disabling visual loss in affected eye(s)
Visual changes (from baseline)	Visual changes causing no or minimal interference with usual social & functional activities	Visual changes causing greater than minimal interference with usual social & functional activities	Visual changes causing inability to perform usual social & functional activities	Disabling visual loss in affected eye(s)
**ENDOCRINE/METABOLIC**				
Abnormal fat accumulation (e.g., back of neck, breasts, abdomen)	Detectable by study participant (or by caregiver for young children and disabled adults)	Detectable on physical exam by health care provider	Disfiguring OR Obvious changes on casual visual inspection	NA

Basic Self-care Functions – Adult: Activities such as bathing, dressing, toileting, transfer/movement, continence, and feeding.

Basic Self-care Functions – Young Children: Activities that are age and culturally appropriate (e.g., feeding self with culturally appropriate eating implement).

Usual Social & Functional Activities – Adult: Adaptive tasks and desirable activities, such as going to work, shopping, cooking, use of transportation, pursuing a hobby, etc.

Usual Social & Functional Activities – Young Children: Activities that are age and culturally appropriate (e.g., social interactions, play activities, learning tasks, etc.).

DIVISION OF AIDS TABLE FOR GRADING THE SEVERITY OF ADULT AND PEDIATRIC ADVERSE EVENTS PUBLISH DATE: DECEMBER, 2004

CLINICAL				
**PARAMETER**	**GRADE 1 MILD**	**GRADE 2 MODERATE**	**GRADE 3 SEVERE**	**GRADE 4 POTENTIALLY LIFE-THREATENING**
Diabetes mellitus	NA	New onset without need to initiate medication OR Modification of current medications to regain glucose control	New onset with initiation of medication indicated OR Diabetes uncontrolled despite treatment modification	Life-threatening consequences (e.g., ketoacidosis, hyperosmolar non-ketotic coma)
Gynecomastia	Detectable by study participant or caregiver (for young children and disabled adults)	Detectable on physical exam by health care provider	Disfiguring OR Obvious on casual visual inspection	NA
Hyperthyroidism	Asymptomatic	Symptomatic causing greater than minimal interference with usual social & functional activities OR Thyroid suppression therapy indicated	Symptoms causing inability to perform usual social & functional activities OR Uncontrolled despite treatment modification	Life-threatening consequences (e.g., thyroid storm)
Hypothyroidism	Asymptomatic	Symptomatic causing greater than minimal interference with usual social & functional activities OR Thyroid replacement therapy indicated	Symptoms causing inability to perform usual social & functional activities OR Uncontrolled despite treatment modification	Life-threatening consequences (e.g., myxedema coma)
Lipoatrophy (e.g., fat loss from the face, extremities, buttocks)	Detectable by study participant (or by caregiver for young children and disabled adults)	Detectable on physical exam by health care provider	Disfiguring OR Obvious on casual visual inspection	NA

Basic Self-care Functions – Adult: Activities such as bathing, dressing, toileting, transfer/movement, continence, and feeding.

Basic Self-care Functions – Young Children: Activities that are age and culturally appropriate (e.g., feeding self with culturally appropriate eating implement).

Usual Social & Functional Activities – Adult: Adaptive tasks and desirable activities, such as going to work, shopping, cooking, use of transportation, pursuing a hobby, etc.

Usual Social & Functional Activities – Young Children: Activities that are age and culturally appropriate (e.g., social interactions, play activities, learning tasks, etc.).

DIVISION OF AIDS TABLE FOR GRADING THE SEVERITY OF ADULT AND PEDIATRIC ADVERSE EVENTS PUBLISH DATE: DECEMBER, 2004

LABORATORY				
**PARAMETER**	**GRADE 1 MILD**	**GRADE 2 MODERATE**	**GRADE 3 SEVERE**	**GRADE 4 POTENTIALLY LIFE-THREATENING**
**HEMATOLOGY**	***Standard International Units are listed in italics***			
Absolute CD4+ count **– Adult and Pediatric > 13 years** (HIV NEGATIVE ONLY)	300 – 400/mm^3 *300 – 400/μL*	200 – 299/mm^3 *200 – 299/μL*	100 – 199/mm^3 *100 – 199/μL*	< 100/mm^3 *< 100/μL*
Absolute lymphocyte count **– Adult and Pediatric > 13 years** (HIV NEGATIVE ONLY)	600 – 650/mm^3 *0.600 x 10^9 – 0.650 x 10^9/L*	500 – 599/mm^3 *0.500 x 10^9 – 0.599 x 10^9/L*	350 – 499/mm^3 *0.350 x 10^9 – 0.499 x 10^9/L*	< 350/mm^3 *< 0.350 x 10^9/L*
Absolute neutrophil count (ANC)				
**Adult and Pediatric, > 7 days**	1,000 – 1,300/mm^3 *1.000 x 10^9 – 1.300 x 10^9/L*	750 – 999/mm^3 *0.750 x 10^9 – 0.999 x 10^9/L*	500 – 749/mm^3 *0.500 x 10^9 – 0.749 x 10^9/L*	< 500/mm^3 *< 0.500 x 10^9/L*
**Infant*†, 2 – ≤ 7 days**	1,250 – 1,500/mm^3 *1.250 x 10^9 – 1.500 x 10^9/L*	1,000 – 1,249/mm^3 *1.000 x 10^9 – 1.249 x 10^9/L*	750 – 999/mm^3 *0.750 x 10^9 – 0.999 x 10^9/L*	< 750/mm^3 *< 0.750 x 10^9/L*
**Infant*†, 1 day**	4,000 – 5,000/mm^3 *4.000 x 10^9 – 5.000 x 10^9/L*	3,000 – 3,999/mm^3 *3.000 x 10^9 – 3.999 x10^9/L*	1,500 – 2,999/mm^3 *1.500 x 10^9 – 2.999 x 10^9/L*	< 1,500/mm^3 *< 1.500 x 10^9/L*
Fibrinogen, decreased	100 – 200 mg/dL *1.00 – 2.00 g/L* OR 0.75 – 0.99 x LLN	75 – 99 mg/dL *0.75 – 0.99 g/L* OR 0.50 – 0.74 x LLN	50 – 74 mg/dL *0.50 – 0.74 g/L* OR 0.25 – 0.49 x LLN	< 50 mg/dL *< 0.50 g/L* OR < 0.25 x LLN OR Associated with gross bleeding
Hemoglobin (Hgb)				
**Adult and Pediatric ≥ 57 days** (HIV POSITIVE ONLY)	8.5 – 10.0 g/dL *1.32 – 1.55 mmol/L*	7.5 – 8.4 g/dL *1.16 – 1.31 mmol/L*	6.50 – 7.4 g/dL *1.01 – 1.15 mmol/L*	< 6.5 g/dL *< 1.01 mmol/L*
**Adult and Pediatric ≥ 57 days** (HIV NEGATIVE ONLY)	10.0 – 10.9 g/dL *1.55 – 1.69 mmol/L* OR Any decrease 2.5 – 3.4 g/dL *0.39 – 0.53 mmol/L*	9.0 – 9.9 g/dL *1.40 – 1.54 mmol/L* OR Any decrease 3.5 – 4.4 g/dL *0.54 – 0.68 mmol/L*	7.0 – 8.9 g/dL *1.09 – 1.39 mmol/L* OR Any decrease ≥ 4.5 g/dL *≥ 0.69 mmol/L*	< 7.0 g/dL *< 1.09 mmol/L*
**Infant*†, 36 – 56 days** (HIV POSITIVE OR NEGATIVE)	8.5 – 9.4 g/dL *1.32 – 1.46 mmol/L*	7.0 – 8.4 g/dL *1.09 – 1.31 mmol/L*	6.0 – 6.9 g/dL *0.93 – 1.08 mmol/L*	< 6.00 g/dL *< 0.93 mmol/L*

* Values are for term infants.

† Use age and sex appropriate values (e.g., bilirubin), including preterm infants.

DIVISION OF AIDS TABLE FOR GRADING THE SEVERITY OF ADULT AND PEDIATRIC ADVERSE EVENTS PUBLISH DATE: DECEMBER, 2004

LABORATORY				
**PARAMETER**	**GRADE 1 MILD**	**GRADE 2 MODERATE**	**GRADE 3 SEVERE**	**GRADE 4 POTENTIALLY LIFE-THREATENING**
**Infant*†, 22 – 35 days** (HIV POSITIVE OR NEGATIVE)	9.5 – 10.5 g/dL *1.47 – 1.63 mmol/L*	8.0 – 9.4 g/dL *1.24 – 1.46 mmol/L*	7.0 – 7.9 g/dL *1.09 – 1.23 mmol/L*	< 7.00 g/dL *< 1.09 mmol/L*
**Infant*†, 1 – 21 days** (HIV POSITIVE OR NEGATIVE)	12.0 – 13.0 g/dL *1.86 – 2.02 mmol/L*	10.0 – 11.9 g/dL *1.55 – 1.85 mmol/L*	9.0 – 9.9 g/dL *1.40 – 1.54 mmol/L*	< 9.0 g/dL *< 1.40 mmol/L*
International Normalized Ratio of prothrombin time (INR)	1.1 – 1.5 x ULN	1.6 – 2.0 x ULN	2.1 – 3.0 x ULN	> 3.0 x ULN
Methemoglobin	5.0 – 10.0%	10.1 – 15.0%	15.1 – 20.0%	> 20.0%
Prothrombin Time (PT)	1.1 – 1.25 x ULN	1.26 – 1.50 x ULN	1.51 – 3.00 x ULN	> 3.00 x ULN
Partial Thromboplastin Time (PTT)	1.1 – 1.66 x ULN	1.67 – 2.33 x ULN	2.34 – 3.00 x ULN	> 3.00 x ULN
Platelets, decreased	100,000 – 124,999/mm^3 *100.000 x 10^9 – 124.999 x 10^9/L*	50,000 – 99,999/mm^3 *50.000 x 10^9 – 99.999 x 10^9/L*	25,000 – 49,999/mm^3 *25.000 x 10^9 – 49.999 x 10^9/L*	< 25,000/mm^3 *< 25.000 x 10^9/L*
WBC, decreased	2,000 – 2,500/mm^3 *2.000 x 10^9 – 2.500 x 10^9/L*	1,500 – 1,999/mm^3 *1.500 x 10^9 – 1.999 x 10^9/L*	1,000 – 1,499/mm^3 *1.000 x 10^9 – 1.499 x 10^9/L*	< 1,000/mm^3 *< 1.000 x 10^9/L*
**CHEMISTRIES**	***Standard International Units are listed in italics***			
Acidosis	NA	pH < normal, but ≥ 7.3	pH < 7.3 without life-threatening consequences	pH < 7.3 with life-threatening consequences
Albumin, serum, low	3.0 g/dL – < LLN *30 g/L – < LLN*	2.0 – 2.9 g/dL *20 – 29 g/L*	< 2.0 g/dL *< 20 g/L*	NA
Alkaline Phosphatase	1.25 – 2.5 x ULN†	2.6 – 5.0 x ULN†	5.1 – 10.0 x ULN†	> 10.0 x ULN†
Alkalosis	NA	pH > normal, but ≤ 7.5	pH > 7.5 without life-threatening consequences	pH > 7.5 with life-threatening consequences
ALT (SGPT)	1.25 – 2.5 x ULN	2.6 – 5.0 x ULN	5.1 – 10.0 x ULN	> 10.0 x ULN
AST (SGOT)	1.25 – 2.5 x ULN	2.6 – 5.0 x ULN	5.1 – 10.0 x ULN	> 10.0 x ULN
Bicarbonate, serum, low	16.0 mEq/L – < LLN *16.0 mmol/L – < LLN*	11.0 – 15.9 mEq/L *11.0 – 15.9 mmol/L*	8.0 – 10.9 mEq/L *8.0 – 10.9 mmol/L*	< 8.0 mEq/L *< 8.0 mmol/L*
Bilirubin (Total)				
**Adult and Pediatric > 14 days**	1.1 – 1.5 x ULN	1.6 – 2.5 x ULN	2.6 – 5.0 x ULN	> 5.0 x ULN

* Values are for term infants.

† Use age and sex appropriate values (e.g., bilirubin), including preterm infants.

DIVISION OF AIDS TABLE FOR GRADING THE SEVERITY OF ADULT AND PEDIATRIC ADVERSE EVENTS PUBLISH DATE: DECEMBER, 2004

LABORATORY				
**PARAMETER**	**GRADE 1 MILD**	**GRADE 2 MODERATE**	**GRADE 3 SEVERE**	**GRADE 4 POTENTIALLY LIFE-THREATENING**
**Infant*†, ≤ 14 days** (non-hemolytic)	NA	20.0 – 25.0 mg/dL<br>*342 – 428 µmol/L*	25.1 – 30.0 mg/dL<br>*429 – 513 µmol/L*	> 30.0 mg/dL<br>*> 513.0 µmol/L*
**Infant*†, ≤ 14 days** (hemolytic)	NA	NA	20.0 – 25.0 mg/dL<br>*342 – 428 µmol/L*	> 25.0 mg/dL<br>*> 428 µmol/L*
Calcium, serum, high (corrected for albumin)				
**Adult and Pediatric ≥ 7 days**	10.6 – 11.5 mg/dL<br>*2.65 – 2.88 mmol/L*	11.6 – 12.5 mg/dL<br>*2.89 – 3.13 mmol/L*	12.6 – 13.5 mg/dL<br>*3.14 – 3.38 mmol/L*	> 13.5 mg/dL<br>*> 3.38 mmol/L*
**Infant*†, < 7 days**	11.5 – 12.4 mg/dL<br>*2.88 – 3.10 mmol/L*	12.5 – 12.9 mg/dL<br>*3.11 – 3.23 mmol/L*	13.0 – 13.5 mg/dL<br>*3.245 – 3.38 mmol/L*	> 13.5 mg/dL<br>*> 3.38 mmol/L*
Calcium, serum, low (corrected for albumin)				
**Adult and Pediatric ≥ 7 days**	7.8 – 8.4 mg/dL<br>*1.95 – 2.10 mmol/L*	7.0 – 7.7 mg/dL<br>*1.75 – 1.94 mmol/L*	6.1 – 6.9 mg/dL<br>*1.53 – 1.74 mmol/L*	< 6.1 mg/dL<br>*< 1.53 mmol/L*
**Infant*†, < 7 days**	6.5 – 7.5 mg/dL<br>*1.63 – 1.88 mmol/L*	6.0 – 6.4 mg/dL<br>*1.50 – 1.62 mmol/L*	5.50 – 5.90 mg/dL<br>*1.38 – 1.51 mmol/L*	< 5.50 mg/dL<br>*< 1.38 mmol/L*
Cardiac troponin I (cTnI)	NA	NA	NA	Levels consistent with myocardial infarction or unstable angina as defined by the manufacturer
Cardiac troponin T (cTnT)	NA	NA	NA	≥ 0.20 ng/mL<br>OR<br>Levels consistent with myocardial infarction or unstable angina as defined by the manufacturer
Cholesterol (fasting)				
**Adult ≥ 18 years**	200 – 239 mg/dL<br>*5.18 – 6.19 mmol/L*	240 – 300 mg/dL<br>*6.20 – 7.77 mmol/L*	> 300 mg/dL<br>*> 7.77 mmol/L*	NA
**Pediatric < 18 years**	170 – 199 mg/dL<br>*4.40 – 5.15 mmol/L*	200 – 300 mg/dL<br>*5.16 – 7.77 mmol/L*	> 300 mg/dL<br>*> 7.77 mmol/L*	NA
Creatine Kinase	3.0 – 5.9 x ULN†	6.0 – 9.9 x ULN†	10.0 – 19.9 x ULN†	≥ 20.0 x ULN†
Creatinine	1.1 – 1.3 x ULN†	1.4 – 1.8 x ULN†	1.9 – 3.4 x ULN†	≥ 3.5 x ULN†
Glucose, serum, high				
Nonfasting	116 – 160 mg/dL<br>*6.44 – 8.88 mmol/L*	161 – 250 mg/dL<br>*8.89 – 13.88 mmol/L*	251 – 500 mg/dL<br>*13.89 – 27.75 mmol/L*	> 500 mg/dL<br>*> 27.75 mmol/L*
Fasting	110 – 125 mg/dL<br>*6.11 – 6.94 mmol/L*	126 – 250 mg/dL<br>*6.95 – 13.88 mmol/L*	251 – 500 mg/dL<br>*13.89 – 27.75 mmol/L*	> 500 mg/dL<br>*> 27.75 mmol/L*

* Values are for term infants.

† Use age and sex appropriate values (e.g., bilirubin), including preterm infants.

DIVISION OF AIDS TABLE FOR GRADING THE SEVERITY OF ADULT AND PEDIATRIC ADVERSE EVENTS
PUBLISH DATE: DECEMBER, 2004

LABORATORY				
**PARAMETER**	**GRADE 1** **MILD**	**GRADE 2** **MODERATE**	**GRADE 3** **SEVERE**	**GRADE 4** **POTENTIALLY LIFE-THREATENING**
Glucose, serum, low				
**Adult and Pediatric ≥ 1 month**	55 – 64 mg/dL *3.05 – 3.55 mmol/L*	40 – 54 mg/dL *2.22 – 3.06 mmol/L*	30 – 39 mg/dL *1.67 – 2.23 mmol/L*	< 30 mg/dL *< 1.67 mmol/L*
**Infant*,†, < 1 month**	50 – 54 mg/dL *2.78 – 3.00 mmol/L*	40 – 49 mg/dL *2.22 – 2.77 mmol/L*	30 – 39 mg/dL *1.67 – 2.21 mmol/L*	< 30 mg/dL *< 1.67 mmol/L*
Lactate	< 2.0 x ULN without acidosis	≥ 2.0 x ULN without acidosis	Increased lactate with pH < 7.3 without life-threatening consequences	Increased lactate with pH < 7.3 with life-threatening consequences
LDL cholesterol (fasting)				
**Adult ≥ 18 years**	130 – 159 mg/dL *3.37 – 4.12 mmol/L*	160 – 190 mg/dL *4.13 – 4.90 mmol/L*	≥ 190 mg/dL *≥ 4.91 mmol/L*	NA
**Pediatric > 2 - < 18 years**	110 – 129 mg/dL *2.85 – 3.34 mmol/L*	130 – 189 mg/dL *3.35 – 4.90 mmol/L*	≥ 190 mg/dL *≥ 4.91 mmol/L*	NA
Lipase	1.1 – 1.5 x ULN	1.6 – 3.0 x ULN	3.1 – 5.0 x ULN	> 5.0 x ULN
Magnesium, serum, low	1.2 – 1.4 mEq/L *0.60 – 0.70 mmol/L*	0.9 – 1.1 mEq/L *0.45 – 0.59 mmol/L*	0.6 – 0.8 mEq/L *0.30 – 0.44 mmol/L*	< 0.60 mEq/L *< 0.30 mmol/L*
Pancreatic amylase	1.1 – 1.5 x ULN	1.6 – 2.0 x ULN	2.1 – 5.0 x ULN	> 5.0 x ULN
Phosphate, serum, low				
**Adult and Pediatric > 14 years**	2.5 mg/dL – < LLN *0.81 mmol/L – < LLN*	2.0 – 2.4 mg/dL *0.65 – 0.80 mmol/L*	1.0 – 1.9 mg/dL *0.32 – 0.64 mmol/L*	< 1.00 mg/dL *< 0.32 mmol/L*
**Pediatric 1 year – 14 years**	3.0 – 3.5 mg/dL *0.97 – 1.13 mmol/L*	2.5 – 2.9 mg/dL *0.81 – 0.96 mmol/L*	1.5 – 2.4 mg/dL *0.48 – 0.80 mmol/L*	< 1.50 mg/dL *< 0.48 mmol/L*
**Pediatric < 1 year**	3.5 – 4.5 mg/dL *1.13 – 1.45 mmol/L*	2.5 – 3.4 mg/dL *0.81 – 1.12 mmol/L*	1.5 – 2.4 mg/dL *0.48 – 0.80 mmol/L*	< 1.50 mg/dL *< 0.48 mmol/L*
Potassium, serum, high	5.6 – 6.0 mEq/L *5.6 – 6.0 mmol/L*	6.1 – 6.5 mEq/L *6.1 – 6.5 mmol/L*	6.6 – 7.0 mEq/L *6.6 – 7.0 mmol/L*	> 7.0 mEq/L *> 7.0 mmol/L*
Potassium, serum, low	3.0 – 3.4 mEq/L *3.0 – 3.4 mmol/L*	2.5 – 2.9 mEq/L *2.5 – 2.9 mmol/L*	2.0 – 2.4 mEq/L *2.0 – 2.4 mmol/L*	< 2.0 mEq/L *< 2.0 mmol/L*
Sodium, serum, high	146 – 150 mEq/L *146 – 150 mmol/L*	151 – 154 mEq/L *151 – 154 mmol/L*	155 – 159 mEq/L *155 – 159 mmol/L*	≥ 160 mEq/L *≥ 160 mmol/L*
Sodium, serum, low	130 – 135 mEq/L *130 – 135 mmol/L*	125 – 129 mEq/L *125 – 129 mmol/L*	121 – 124 mEq/L *121 – 124 mmol/L*	≤ 120 mEq/L *≤ 120 mmol/L*
Triglycerides (fasting)	NA	500 – 750 mg/dL *5.65 – 8.48 mmol/L*	751 – 1,200 mg/dL *8.49 – 13.56 mmol/L*	> 1,200 mg/dL *> 13.56 mmol/L*
Uric acid	7.5 – 10.0 mg/dL *0.45 – 0.59 mmol/L*	10.1 – 12.0 mg/dL *0.60 – 0.71 mmol/L*	12.1 – 15.0 mg/dL *0.72 – 0.89 mmol/L*	> 15.0 mg/dL *> 0.89 mmol/L*

* Values are for term infants.

† Use age and sex appropriate values (e.g., bilirubin), including preterm infants.

DIVISION OF AIDS TABLE FOR GRADING THE SEVERITY OF ADULT AND PEDIATRIC ADVERSE EVENTS PUBLISH DATE: DECEMBER, 2004

LABORATORY				
**PARAMETER**	**GRADE 1 MILD**	**GRADE 2 MODERATE**	**GRADE 3 SEVERE**	**GRADE 4 POTENTIALLY LIFE-THREATENING**
**URINALYSIS**	***Standard International Units are listed in italics***			
Hematuria (microscopic)	6 – 10 RBC/HPF	> 10 RBC/HPF	Gross, with or without clots OR with RBC casts	Transfusion indicated
Proteinuria, random collection	1 +	2 – 3 +	4 +	NA
Proteinuria, 24 hour collection				
**Adult and Pediatric ≥ 10 years**	200 – 999 mg/24 h<br>*0.200 – 0.999 g/d*	1,000 – 1,999 mg/24 h<br>*1.000 – 1.999 g/d*	2,000 – 3,500 mg/24 h<br>*2.000 – 3.500 g/d*	> 3,500 mg/24 h<br>*> 3.500 g/d*
**Pediatric > 3 mo - < 10 years**	201 – 499 mg/m^2/24 h<br>*0.201 – 0.499 g/d*	500 – 799 mg/m^2/24 h<br>*0.500 – 0.799 g/d*	800 – 1,000 mg/m^2/24 h<br>*0.800 – 1.000 g/d*	> 1,000 mg/ m^2/24 h<br>*> 1.000 g/d*

* Values are for term infants.

† Use age and sex appropriate values (e.g., bilirubin), including preterm infants.

12-28-04 Page 20 of 20 Version 1.0

APPENDIX D: IAVI V001 SAMPLE CONSENT INFORMATION SHEET

CONSENT TO PARTICIPATE IN A MEDICAL RESEARCH STUDY WITH AN EXPERIMENTAL HIV VACCINE

**Title of Study:**	A Phase I, Randomized, Placebo-Controlled, Double-Blind Trial to Evaluate the Safety and Immunogenicity of a Multiclade HIV-1 DNA Plasmid Vaccine Followed by Recombinant, Multiclade HIV-1 Adenoviral Vector Vaccine or the Multiclade HIV-1 Adenoviral Vector Vaccine Alone in Healthy Adult Volunteers not Infected with HIV
**Study Number:**	IAVI V001
**Sponsor:**	International AIDS Vaccine Initiative (IAVI)<br>110 William Street, 27th Floor<br>New York, New York 10038-3901<br>USA
**Study Protocol Date:**	06 June 2005
**Principal Investigator:**	
**Study Site:**	

Introduction

You are being asked to join a clinical research study to test a human immunodeficiency virus preventive vaccine. Human immunodeficiency virus is often called HIV. HIV is the virus that causes people to eventually become sick with the disease called acquired immunodeficiency syndrome (AIDS).

Over 40 million people in the world are currently infected with HIV. The number of people infected continues to rise quickly. Other infectious diseases such as smallpox and poliomyelitis have been controlled, or even eliminated, by vaccination programs. Many experts believe that an HIV vaccine offers hope in controlling the epidemic. Currently, there is no licensed and available HIV vaccine that can protect you against the HIV virus that causes the disease AIDS.

Many different types of HIV vaccines are now being developed and tested. This study is one of several studies that will test HIV vaccines in humans. More research studies will have to be carried out before the vaccines can be used in the general population. The United States National Institutes of Health (US NIH) developed the vaccines that are used in this study, and the International AIDS Vaccine Initiative (IAVI) is the organization that is doing the study at your site. The U.S. Food and Drug Administration (FDA) and the authorities in your country have given permission for this study.

Before you agree to join this research study, you need to understand the purpose of the study and know what will be done in the study. You should also know your rights as a participant in the study. Please ask the study nurse or study doctor to explain any words or information you do not understand.

Purpose
The goal of this study is to see if the study vaccines are safe and tolerable and to find out how the immune system responds to the study injections.

Requirements to Participate
You must be a healthy male or female who is not infected with HIV. You must not do anything that puts you at risk of HIV infection. You must be between 18 and 50 years of age (not yet reached your 51st birthday). If you are a woman, you must not be pregnant or breastfeeding and must agree to delay pregnancy for the duration of your participation in the study by using effective birth control. This is because the effect of the test vaccine on your unborn child is not yet known.

You must be well informed about this study and willing and able to provide written consent to participate in this study.

The Study Vaccines
There are two types of HIV vaccines being tested in this study. They do not contain material from live-HIV, from people who are infected with HIV, or from people who are resistant to HIV. The vaccines do not contain any blood or blood products. Both types of vaccine contain the same artificially made DNA (genetic information), which resembles a small part of the genes of HIV. When injected into your arm muscle, the vaccines may produce a response from your immune system.

The first type of vaccine, VRC-HIVDNA016-00-VP, is called a DNA vaccine and contains only a small part of artificially made HIV DNA. You can NOT get HIV infection or AIDS from this vaccine. This vaccine is given as an injection into the muscle of the upper part of your arm.

The second type of vaccine, VRC-HIVADV014-00-VP, a rAd5 recombinant adenovirus sub type 5 vaccine (referred to as the rAd5 vaccine) and contains only a small part of artificially made HIV DNA. The adenoviral vector vaccine is made from a virus called adenovirus. This virus is common in every day life and can cause colds and respiratory infections. Like the DNA vaccine, the adenoviral vector vaccine has been changed and cannot grow or cause infections because the DNA that instructs it to grow has been removed. It is called a “vector” because it is only a carrier of the HIV DNA needed to make HIV proteins. The adenovirus in the vaccine enters the muscle, where it delivers the DNA instructions to build proteins that imitate some of the proteins of the HIV-1 virus. In this study it will be used alone or after injections with the DNA vaccine in hopes of boosting the immune response caused by the DNA vaccine. Like the DNA vaccine, you can NOT get HIV infection or AIDS from this vaccine. This vaccine is given as a single injection into the muscle of the upper part of your arm.

The vaccines are being tested to find out which dose levels and which combinations of the two vaccines produce the best immune responses against HIV.

These study vaccines are not yet approved either by the US. Food and Drug Administration (FDA) or national regulatory agencies for treating or preventing HIV infection. They are experimental vaccines which the FDA allows to be used for research purposes only.

Length of Participation
This study will require that you make approximately 9 or 17 visits to the study clinic, depending on which group you are assigned to. If you do not feel well and need to return to the clinic more

often. The study nurse or doctor will tell you when these visits will be. This study will be approximately 12 months long. At some visits you will have a physical exam by a doctor and blood samples may be taken. You will be given the vaccine at 1 or 4 visits depending on the group you are in. Vaccination Visits will take approximately 1 to 2 hours and Follow-Up Visits will take approximately 1 hour to complete.

Study Procedures
About 64 volunteers are expected to participate in this study at two sites: Projet San Francisco in Kigali, Rwanda and KAVI in Nairobi, Kenya.

Screening Visit/s
This consent form tells you about the study and the study nurse or study doctor will talk to you about this form. If you are unable to read, it will be read to you in a language that you can understand. You will be asked questions to make sure that you understand this consent form. If you agree to participate, you will sign your name on 2 copies of this consent form which means that you agree to take part. You may keep one copy and a copy will be kept at the clinic. If you do not wish to keep a copy, the study clinic will keep it for you in a safe and secure place.

If you wish to have your spouse or partner or another person who is not part of the study team here to help you understand the study, you can. They will be asked to sign your consent as a witness. It is your choice whether you decide to be in this study or not. If you decide not to participate, none of your rights will be taken away and the care that you would normally have will not change. If you decide not to join, there is no licensed HIV vaccine now available. There might be another HIV vaccine study or a licensed vaccine in the future. It is not known if getting this vaccine would make you respond better or worse to a future vaccine.

In order to determine if you are able to join this study, these things will be done by your study doctor or nurse:

- You will be asked questions to see if you qualify for the study.
- You will be asked questions about your risk of exposure to HIV (e.g., sexual behavior and drug use).
- A complete medical history and physical examination will be done.
- Approximately 6 tubes (10 mL each) of blood will be taken from you. The blood will be used for an HIV test, Hepatitis B, syphilis and other tests to make sure you are healthy. You will be given pre- and post-HIV test counseling and risk reduction counseling.
- Urine samples will be taken to make sure your kidneys are healthy.
- If you are a woman, you will have a urine pregnancy test to make sure that you are not pregnant. This is being done because the safety of these vaccines for pregnant women and their babies is not yet known.
- If you are a woman, you will be given family planning counseling.

Injection Visits
You will be assigned by chance (like the flip of a coin) to one of four groups below:

- Group A: 12 volunteers will receive one injection of low dose rAd5 vaccine and 4 volunteers will receive one injection of placebo.
- Group B: 12 volunteers will receive one injection of high dose rAd5 vaccine and 4 volunteers will receive one injection of placebo

- Group C: 12 volunteers will receive 3 injections of DNA vaccine followed by 1 injection of low dose rAd5 vaccine and 4 volunteers will receive 4 injections of placebo.
- Group D: 12 volunteers will receive 3 injections of DNA vaccine followed by 1 injection of high dose rAd5 vaccine and 4 volunteers will receive 4 injections of placebo.

Depending on the group to which you are assigned, you will have 9 or 17 visits (not including screening) and you will be given either one or four injections of the vaccine or placebo in the upper arm muscle during the study. You will not be given more than one injection at a visit. For Groups A and B, there is only one injection of vaccine or placebo. For Groups C and D, the first, second and third injections will be given one month apart and the fourth injection will be given 6 months after the first injection. You have a 3 out of 4 chance that you will receive the test vaccine and a 1 out of 4 chance that you will receive the placebo. A placebo looks like the vaccine, but it does not contain the vaccine.

You and your study doctor and nurse will not know if you are getting vaccine or placebo until the end of the study. The Principal Investigator may request this information in the case of a medical emergency if it will help him/her to make decisions regarding your medical care. You will receive this information after the end of the entire study when all the volunteers have completed all their visits and all the results are available.

Before you are given an injection, these things will be done by your study doctor or nurse:
- A brief medical history and physical examination will be done.
- Up to 9 tubes (10 mL each) of blood may be taken. At some visits, the blood will be tested for HIV and results will be given to you at your next visit. You will be given pre- and post-HIV test counseling and risk reduction counseling. At some visits, your blood will be also be tested for safety and response to the vaccine.
- Urine samples will be taken at some visits to test your kidneys.
- If you are a woman, you will have a urine pregnancy test prior to receiving each vaccination to make sure you are not pregnant.
- If you are a woman, you will be given family planning counseling.

You will need to stay in the clinic for up to an hour after the injections to ensure you are not having any immediate health problems. The study nurse or study doctor will take your blood pressure before each injection and 30 minutes after each injection and at your request.

After each injection you will be given a diary card to record any symptoms, any visits to a health care provider or any use of medicine. You will be asked to record this information for 3 days after each injection. You will bring the diary card to the clinic 3 days after each injection. If you experience any unusual, alarming or unexpected symptoms or require hospitalization during the course of the study, please tell your study clinic right away.

Visit 3 Days after Each Injection
You will return to the study clinic 3 days after each injection visit with your completed diary card. The study nurse or study doctor will examine you for any reactions to study injections, collect a medical history, perform a physical examination and record the information on the study chart.

Visit 14 Days after Each Injection
You will return to the study clinic two weeks after each injection visit:
- A medical history and physical examination will be done.

- A blood sample will be collected to test for safety and response to the vaccine.
- A urine sample will be collected to test your kidneys at some visits.

Follow-Up Visits
If you are assigned in groups C or D, you will be asked to return to the study clinic at 6.5, 7, 7.5, 9 and 12 months after your first injection. If you are assigned in groups A and B you be asked to return at 1, 1.5, 3, 6, 9 and 12 months after the first injection.

The following things will be done by your study doctor or nurse:
- Brief medical history and physical examination will be done.
- A blood sample will be collected to test for safety and response to the vaccine.
- Blood will be collected for HIV testing at Months 3, 6, 9 and 12 (last study visit) for Groups A & B and at Months 2, 6, 9 and 12 (last study visit) for Groups C & D. There will be pre- and post- HIV test and risk reduction counseling. The HIV test results from the Month 12 visit will be available to you soon after the visit.

Results of routine laboratory tests will be discussed with you at your next visit or sooner, if required for your health.

You should inform the study site personnel before beginning any new medications, receiving any other vaccinations or entering any other studies.

Storage of Blood
After the tests needed for the study are done using the blood that was taken from you, the extra blood may be stored. A number, rather than your name, will be used to label this blood so it is confidential. The stored blood may be used in the future for quality control purposes and other tests related to approved HIV vaccine research and development. Your blood will be sent to external, national or international laboratories including the Core Laboratory of IAVI located in London and the Vaccine Research Center (VRC) at National Institutes of Health (NIH) in the US for additional testing. It is not known at this time to which laboratories (other than IAVI Core Lab and VRC, NIH) these samples will be sent or how long the samples will be stored. All of these tests will be for HIV vaccine research only.

Risks and Discomforts
Risks of blood drawing: Your blood will be taken at the screening and study visits by inserting a needle into your vein and this can cause temporary local pain, bruising, lightheadedness, anemia and, rarely, infection.

Risks from injections: An injection device called a Biojector will be used to administer the DNA vaccine or placebo to volunteers assigned to Groups C and D. This has been used routinely for some kinds of vaccinations. These vaccinations are usually well tolerated, but there may be a cut, bruise, swelling or tenderness. The rAd5 vaccine will be administered by a needle and syringe.

Risks of vaccination: The safety of the vaccine will be reviewed frequently during the study. With any new medicine or vaccine, there is a possibility of totally unexpected side effects, although previous testing indicates that this is unlikely. After you get the vaccine, you may experience pain, soreness, redness/discoloration bruising and swelling around the site of the injection. It does not happen often, but sometimes this causes a bacterial infection at the part of your body where you got the injection. As with all vaccines or drugs, you could have an allergic reaction – a rash, hives, or even difficulty breathing. Life threatening allergic reactions have not

been seen in previous studies, although they can occur; therefore, the clinic staff will watch you for 30 minutes after each injection. There may be other side effects, even serious ones that are not known. Therefore, it is important that you report any side effects to the clinic staff as soon as they occur.

Risks of the experimental DNA vaccine: The researchers expect the risks of this DNA vaccine to be similar to those of other DNA vaccines. Possible risks related to DNA vaccines include: muscle damage; antibodies to DNA leading to illness; and insertion of the vaccine DNA into the body's DNA (leading to cancer) or into the DNA of a bacteria or virus in your body. None of these possible risks of DNA vaccines have been seen in laboratory tests or in animals or humans so far, but you need to be aware of these possible risks. It is important to know that after nearly a thousand humans have received DNA vaccines, the tolerability and safety record has been very good so far. During this study, regular blood tests and check-ups will be performed to monitor these possible side effects. Some blood will be stored during the study in case additional safety tests are needed.

Not all the risks and side effects of these new vaccine candidates are known. This DNA vaccine has been given to 15 people in the first study in people. All of the risks and side effects are not known. One person was found to have a moderately low blood sugar 14 days after the third vaccination, but did not have any symptoms. A different person complained of moderate dizziness for a day on the 13th day after vaccination. It is not known if these problems were related to vaccination. An unexpected finding in the first study was that following four of the 44 injections a small red bump and then a scab formed at the injection site. The scab was less than ½ inch across. It was not deep and it was not infected. The scab came off after a few days. The skin healed without needing any treatment.

A similar DNA vaccine has been given to over 200 people in the U.S. and Uganda. In these other studies, people had frequent laboratory testing of blood and urine samples. Some changes in test results were seen for blood sugar, liver activity, red blood cell count, white blood cell count and urine protein. These changes did not cause symptoms, and test results returned to the usual values without treatment. It is not known if the temporary changes in lab test results were related to the study vaccine or happened for other reasons. In this study you will also have laboratory testing of blood and urine to check for changes. One person had hives that started 4 days after vaccination. It is not known if the hives were related to the vaccine, but it is possible.

Other DNA vaccines against HIV have been given to hundreds of people in clinical trials.

Risks of the experimental adenoviral vector vaccine: Different types of adenovirus have been given to people. The U.S. military has used adenovirus given by mouth to vaccinate more than 10 million people against adenovirus infections, with no serious reactions. In the past 10 years, about 1000 people have received experimental adenoviral vector vaccines. Most of these people were seriously ill before they got the vaccines. These people received injection of vaccine directly into the area of the body that had disease. People in these studies experienced fever, laboratory abnormalities, changes in chest x-rays (when vaccine was injected into the lungs or nose), and liver inflammation (when vaccine was injected into the liver).

These other adenoviral vector vaccines and products have been given to humans in other studies that tested treatment of cancer and inherited conditions. In one study a volunteer died from a reaction to the adenovirus after he was given a large dose directly into the main

liver blood vessel for the purpose of treating an inherited condition. In the study in which you will be participating much lower doses are used and the injection is into your arm muscle.

The adenoviral vector vaccine in this study has been given in the muscle in the arm to at least 90 people. Some people experienced headache, nausea, tiredness, pain and/or redness at the injection site. Other things have been seen in the other studies, but it is uncertain if they are caused by the study vaccine. One person had a seizure about two months after starting the study, but that person had a seizure three years before entering the study. It is unlikely that the vaccine had anything to do with it. Another person had a little change in liver blood test about one month after entering the study. In this case it is likely that the person had the liver abnormality before joining the study. The blood test returned to normal. One person had diarrhea lasting one day and another had a low white blood cell count. For both, the problem resolved without treatment. Other study participants also had small temporary changes in laboratory and urine tests while in the study, but it is not known whether these had anything to do with the study vaccine. However, all the possible risks or side effects are not known.

After an adenoviral vector vaccination some people have a flu-like condition with fever, headache, muscle aches, tired feeling and chills. It starts about 12-16 hours after vaccination and lasts a few hours. A few people have had nausea. Some people have injection site pain or discomfort in the first few days after a vaccination. The flu-like symptoms and injection site pain or discomfort may be treated with an over-the-counter medicine for pain and fever. Also, you may develop antibodies to adenovirus type 5 from the rAd5 vaccine. It is possible you would not be able to receive (or would have a reduced response to) future products that used an adenoviral vector. Currently there are no products approved by the FDA that use an adenoviral vector.

Risks of the combination of DNA and adenoviral vector vaccines: The DNA vaccine and adenoviral vector vaccines in this study have not been tested together before in people. The combination of a similar experimental DNA vaccine followed by the same experimental adenoviral vector vaccine used in this study has been given to approximately 60 people before, there were no serious safety concerns in that study. We do not know if this study will have similar results to the earlier study.

Possible risks related to HIV exposure: This study will show us how well the vaccine is tolerated (safety) and your body's ability to make a response to it. It is not looking to see if the vaccine can prevent HIV infection or disease. Until larger studies have been done we will not know whether this vaccine can prevent HIV infection or disease. HIV infection and AIDS can occur in a person who has received a study vaccine. If you are exposed to HIV after receiving these study injections, your risk of getting infected with HIV and developing ADIS is not known. If you do get infected, the research team also does not know what effect the vaccine may have on the disease. The time that it takes for you to become sick from HIV/AIDS may be the same, or longer, or even shorter than the time that it is expected without the vaccine. THEREFORE, YOU SHOULD NOT DO ANYTHING THAT MAY PUT YOU AT RISK OF GETTING HIV. YOU SHOULD NOT CONSIDER YOURSELF PROTECTED FROM HIV AFTER GETTING THE VACCINE (OR PLACEBO).

Pregnancy risks: We do not know what effect the vaccine would have on an unborn child if given to a pregnant woman. Women who may be able to bear children should use a reliable form of contraception until they have completed the study. Reliable forms of contraception

include oral contraceptive pills, Norplant implants, injectable contraceptives, the intrauterine device (IUD), condoms, and operations like tubal ligation or vasectomy. A pregnancy test will be done before the study begins and before each injection. You can get condoms for free at the trial site. If you become pregnant during the injection period, you will not receive any further injections. The study staff will stay in contact for with you, for safety information only, until the end of the study and delivery of the baby and.a doctor will check your baby.

<u>Possible social risks</u>: Following the injections, you may test positive on a routine HIV antibody test. This might happen even if you have not been infected with HIV. It could mean that your body has been exposed to the vaccine and has produced antibodies to it. If you test positive on a routine HIV test, additional tests will be done to find out if it's because of the vaccine or because you got infected with HIV due to exposure in the community. If your HIV test result is positive because you received the vaccine but you are not infected with HIV, you will not be told until the end of the study. The clinic will offer you the special tests even after the study ends until the routine HIV test is negative.

You will be tested for HIV six separate times during this study: at the screening visit, before the first and fourth injections (Months 0 and 6), at Month 2 and Month 9 and at your final study visit (Month 12). If you get naturally infected with HIV due to exposure in the community and have not had all your injections, you will not have more injections. The number of immune cells in your body and the amount of virus in your blood will be measured.

If you do get infected with HIV due to exposure in the community, it will be hard. Some people become sad or depressed when they learn they have HIV. The counselors at your site have been trained to assist and support you in understanding the meaning of your test result and in helping you to cope with a positive test result. You are welcome to return for further counseling at any time.

You will be referred for support and care and antiretroviral medications when clinically indicated according to the accepted guidelines for up to 5 years after treatment is initiated. If third party insurance or public funding is not available in your country, we will provide anti-retroviral therapy at no charge to you for 5 years after it is needed.

If you need an HIV test outside the study, please contact the study team first. We will offer HIV testing or suggest a lab that can tell the difference between a positive result from the vaccine or an HIV infection. ONLY with your permission will we give the results to a third party. To avoid any problems, you will be offered an identification card that shows that you joined this study. A contact number of the study doctor or counselor will be given to you in case of questions or medical emergencies.

It is unknown whether receiving this HIV vaccine will alter your immune response to any future HIV vaccine that you might receive.

You should not donate blood while you are participating in the study.

There is a possibility that participating in this study WILL put you at social/personal risk. Some participants in other HIV vaccine studies have had personal problems because of their participation. Spouses, other family members, or sexual partners have sometimes reacted by:

- becoming angry when a participant joined a study without consulting them
- worrying that the test vaccine would be harmful

- assuming that the participant was infected with HIV and shunning them
- assuming that the participant is engaging in certain sexual activities or drug use, and treating them unfairly

On rare occasions, a participant has lost a job because of being in an HIV study. This was either because the study took too much time from work, or because the employer thought the participant was HIV infected or at high risk for HIV.

Possible risks of genetic tests: Some of the blood drawn from you as part of this study will be used for genetic testing. The genetic testing is being done to see if different types of immune responses to the vaccine seem to be related to genetic differences in people. The testing will be done in a laboratory using your stored blood identified only by a sample number. Your name will not appear with the sample. The results of the genetic testing will appear only in confidential study-related documents; the information will not be in your medical record nor will you be told the results of the testing. New genetic research tests to help understand if a vaccine might work may be done on stored samples in the future

Benefits
There are no direct benefits for you in taking part in the study except that you will get information about your general health and your HIV status. You will receive HIV counseling and family planning counseling. The information that we collect from this study may help to develop an effective HIV vaccine.

Alternatives
There is no alternate licensed HIV vaccine available now. The only alternative is to not participate in this study.

Injuries
We do not expect you to suffer any injury as a result of participating in this study. However, in case you are injured as a result of being in this study, you will be given the necessary treatment for your injuries including emergency treatment without charge. If the study clinic cannot provide the type of treatment you need you will be referred to a clinic where treatment will be provided for you at no charge. The trial site and their funding agencies and collaborators including US NIH will not give you money if you have an injury resulting from this study.

If you have any symptoms or medical problems that you think are due to getting these vaccines, you should report them right away to Dr. ____________ at the following telephone number _________ or mobile number __________.

Circumstances for Discontinuation from Injections or Withdrawal from the Trial
Your participation in the trial is completely voluntary. You can withdraw from the study at any time without giving a reason. Withdrawal will NOT cause you to lose any rights you have or influence any current or future medical care you may need. If you decide not to participate, there might be another HIV vaccine trial or a licensed vaccine in the future.

The study injections may be stopped without your consent for the following reasons:

- If your doctor feels that study injections are harmful to your health
- If you have serious side effects from the injection

- If you become pregnant
- If you become HIV infected

If injections have been discontinued you will be asked to continue the follow up visits until the end of the study, if possible.

You may be removed from the study without your consent for the following reasons:
- If you don't keep appointments
- If the Data Monitoring and Ethics Committee feels that the study should be stopped
- If the study sponsor (IAVI or NIH) decide to stop the study
- If the US FDA or local regulatory authorities decide to stop the study

If you stop the study early for whatever reason, you may be asked to have a physical examination by a doctor and blood samples may be taken to test for safety and response to the vaccine. You may also receive HIV testing with pre- and post- test counseling, a pregnancy test and family counseling.

New Information
You will be told of any new information gained during the course of the study that might cause you to change your mind about staying in the study. You will be told about new information gained in this trial and trials elsewhere as well as information on any other HIV vaccines. You will also be informed if a safe and effective vaccine becomes available.

Supervision of the Study
The conduct of the study will be supervised by a group of experts called a Trial Steering Committee. All data collected will be regularly checked by independent monitors and a Data Monitoring and Ethics Committee.

Confidentiality
Your participation in the study, all information collected about you as well as all results of laboratory tests will be kept strictly confidential to the extent permitted by law. You will be identified by a unique identity number known only to you and the clinic staff. In addition to the clinic staff, people from national or international government regulatory agencies, members of the Ethics Committee, study monitors, auditors, inspectors and representatives of the IAVI (study sponsor) and the US NIH (vaccine provider) will check the records to make sure that the trial was conducted properly. They must respect your confidentiality. Your identity will not be disclosed in any publication or presentation of this study.

End of Study
At the end of the study, when all volunteers have completed their visits, the results of the study will be analyzed. This analysis may take about 6 months after the end of the study. At that time you will be told whether you received the vaccine or placebo, which is called unblinding, and you will be informed about the study results.

Reimbursement
You will be reimbursed X (the equivalent of $X) for each scheduled protocol visit. These reimbursements will continue for as long as you continue to participate in the study; the exact amount and nature of the reimbursements may be modified in light of changes in

bus fares, exchange rates, cost of living, or hospital coverage. Being in this study will not require you to pay for anything: vaccine, research clinic visits, examinations, laboratory tests and transportation costs that are related to this study will be covered.

Conflict of interest

The NIH, including some members of the Vaccine Research Center (VRC) scientific staff, developed the investigational vaccine being used in this research study. The results of this study could play a role in whether the US FDA will approve the vaccine for sale at some time in the future. If approved, the future sale of the vaccine could lead to payments to the NIH and to some NIH/VRC scientists. By U.S. law, government scientists are eligible to receive such payments for their inventions. You will not receive money or other compensation should this occur. Please discuss with your study doctor any questions you may have about these issues.

Contact Numbers
If you have any questions regarding the study or your participation in the study, you can call Dr.___________, the Principal Investigator, at ___________ or mobile:_________.
If you have a medical problem related to your study participation, please contact Dr. ________ at the ________________.
Nurse/Counselors are available at the ____________ at (Tel) _____________.
f you have a question about your rights as a research volunteer you should contact Dr.________, the Chairman of the Ethics Committee at ________, Tel:____________.

IAVI V001 ENROLMENT CONSENT FORM SIGNATURE PAGE
I, (name of volunteer) ____
Of (address)
____
____
____
____
____
____
agree to take part in the research project titled: A Phase I, Randomized, Placebo-Controlled, Double-Blind Trial to Evaluate the Safety and Immunogenicity of a Multiclade HIV-1 DNA Plasmid Vaccine Followed by Recombinant, Multiclade HIV-1 Adenoviral Vector Vaccine or the Multiclade HIV-1 Adenoviral Vector Vaccine Alone in Healthy Adult Volunteers not Infected with HIV

Volunteer:
I have been told about the study and what I will need to do and I understand and agree. I understand that it is my choice to be in the study and that I may stop being in the study at any time for any reason, and that this will not affect my legal rights.

Participant Signature__________________________

Name_____________________ Date ______________________

Witness:
I sign here as a witness to the consent process. I have participated in the discussion and witnessed the volunteer's consent to study participation:

Witness Signature _______________________

Name_______________ Date_________________

Person obtaining consent:
I have explained the nature, demands and foreseeable risks of the above research to the volunteer:

Signature of person obtaining consent_________________

Name_______________Date______

Principal Investigator:

Signature of Principal Investigator__________________

Name___________________ Date_______________

APPENDIX E: HIV TESTING ALGORITHM

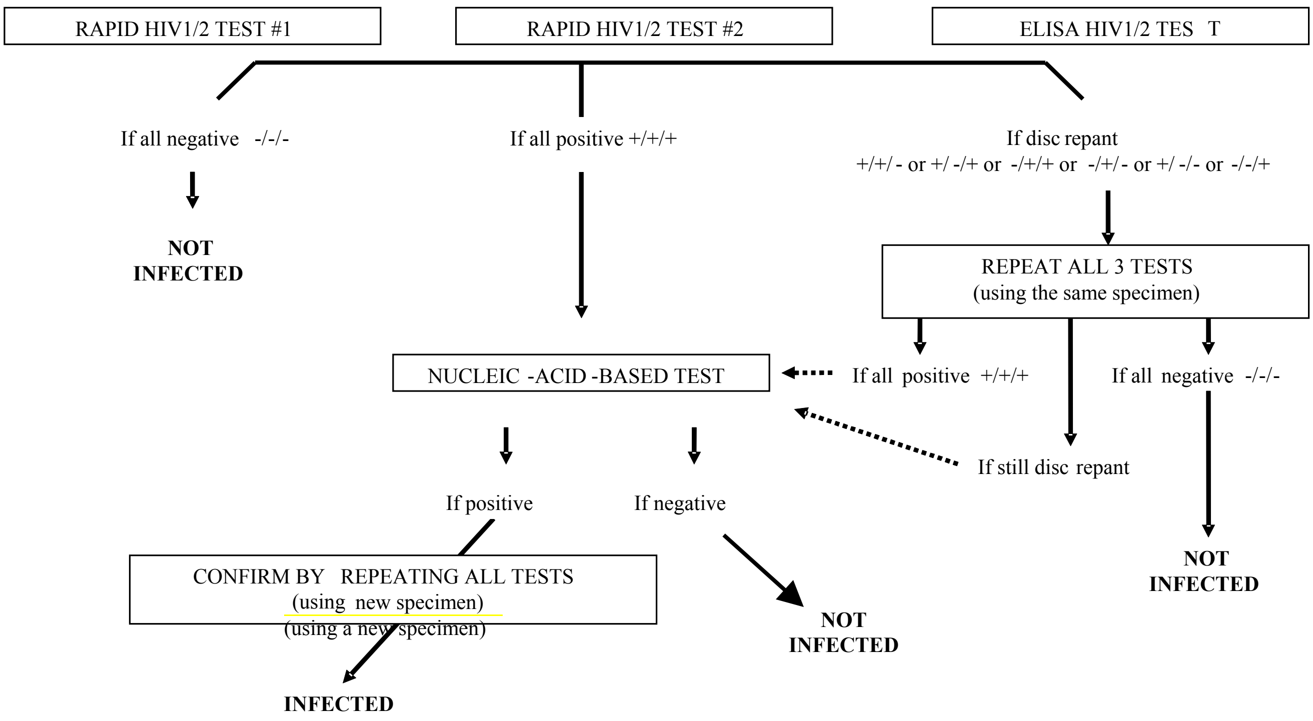

Supplement: Protocol S1 — A Phase I Randomized, Placebo-Controlled, Double-Blind Trial to Evaluate the Safety and Immunogenicity of a Multiclade HIV-1 DNA Plasmid Vaccine Followed by Recombinant, Multiclade HIV-1 Adenoviral Vector Vaccine or the Multiclade HIV-1 Adenoviral Vector Vaccine Alone in Healthy Adult Volunteers not Infected with HIV (5.64 MB PDF) [file pone.0012873.s005.pdf]
